# Photoredox Catalytic Synthesis of Indoles via Direct N–H Activation to Generate Putative Aminyl Radicals

**DOI:** 10.1021/acs.orglett.5c03410

**Published:** 2025-09-08

**Authors:** Juulia Talvitie, Andrés Mollar-Cuni, Jarkko Nyman, Juho Koivula, Lisa Hendrickx, Pedro Muñoz Rodríguez, Juho Helaja

**Affiliations:** Department of Chemistry, University of Helsinki, 00014 Helsinki, Finland

## Abstract

Visible-light-excited 3,6-bis­(trifluoromethyl)-9,10-phenanthrenequinone (PQ-CF_3_*) was used as a photocatalyst in the synthesis of 3-substituted *N*-pyridyl indoles via cyclization of 2-vinylarylamines, where the photocatalyst was catalytically regenerated with the chloro­(pyridine)­cobaloxime complex. The versatility of the reaction was shown with 22 substrates providing up to 83% yields. Based on the mechanistic studies, we propose that PQ-CF_3_ directly activates the N–H bond, generating a nitrogen-centered aminyl radical as the key intermediate.

## Graphical abstract

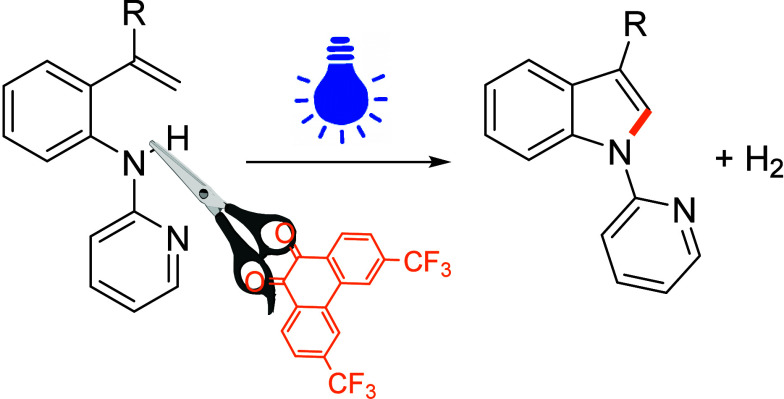

In photoredox catalysis, the generation of carbon-centered radicals by direct activation of C–H bonds and exploiting these in various synthetic manifolds has become increasingly popular in recent years. Analogously, it is known that nitrogen-centered radicals offer versatile reactivity in organic synthesis.^1^ However, aminyl radicals remain the least utilized N radicals^2^ as their direct generation is challenging due to their typically high bond dissociation energies (BDEs, e.g., >90 kcal/mol for anilines).^3^ Hitherto, Wu and co-workers have demonstrated that the generation of N radicals via direct N–H activation of arylamines can be achieved by visible light irradiation of quantum dots to be utilized in aryl coupling reactions.^4^

Our group has recently developed and applied 9,10-phenanthrenequinones (PQs) for photoredox catalysis to operate either via a single-electron transfer (SET) or hydrogen atom transfer (HAT) mechanism.^5,6^ Particularly, the 3,6-bis­(trifluoromethyl)-functionalized PQ derivative (PQ-CF_3_) provided a strong HAT catalyst for the C–H activation of secondary alcohols (BDEs ≈ 90 kcal/mol).^6^ This observation intrigued us to investigate whether visible-light-excited PQ-CF_3_ could activate N–H bonds in a similar fashion to allow for interesting synthetic applications.

Indole is a widely abundant alkaloid scaffold in both natural products and synthetic pharmaceuticals, exhibiting a wide range of valuable pharmacological properties.^7–12^ Therefore, the exploration of new methods for expedient indole synthesis has been active since the early days of synthetic chemistry. Photocatalysis provides an increasingly popular alternative for the task, and several types of starting materials have been utilized in intra- and intermolecular protocols for the construction of the indole ring.^13^

When indoles are synthesized via the cyclization of modified aromatic scaffolds, many of the existing photocatalytic strategies rely on SET catalysts to generate a radical cation intermediate of the substrate (Scheme 1). In 2012, Maity and Zheng reported a Ru-catalyzed synthesis of 2-substituted indoles via electrocyclization of 2-vinylarylamines.^14^ While indoles were afforded in good yields, the reaction proceeded only when nitrogen was substituted with an electron-rich 4-MeO-phenyl group. Shi and co-workers utilized a similar strategy to produce *N*-benzyl-protected 3-substituted indoles using an ultraviolet (UV)- or visible-light-excited acridinium photocatalyst.^15^ These examples inspired us to study whether analogous indole cyclizations could be accomplished via a neutral aniline N radical.

**Scheme 1:**
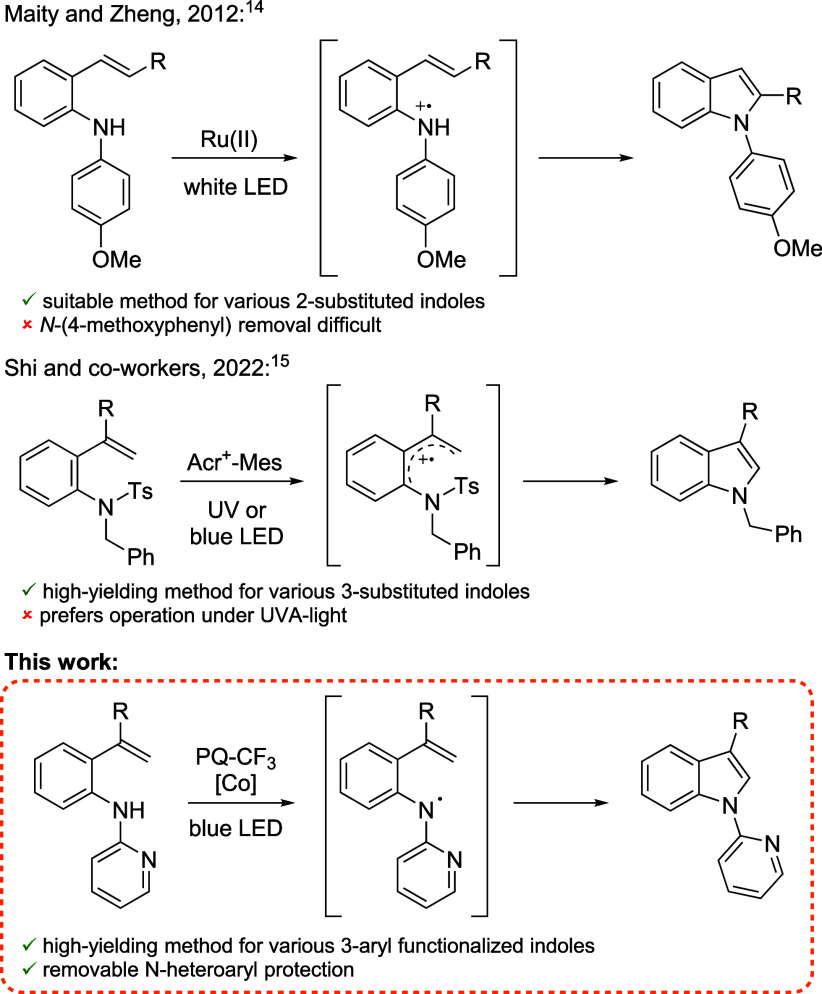
Photoredox Catalytic Strategies to Synthesize Indoles via Cyclization of 2-Vinylarylamines

We started investigating the photocatalytic synthesis of indoles by performing the cyclization of 2-vinylarylamine **1a** in MeCN with 10 mol % PQ-CF_3_ as the photocatalyst and O_2_ as the terminal oxidant (Table 1, entry 6). The reaction produced indole **2a** in 55% yield after 1 h of irradiation, but a prolonged reaction time led to the formation of unidentified side products and loss of indole. We reasoned that the presence of oxygen radicals and H_2_O_2_ could cause the overoxidation of **2a**,^16^ suggesting that an inert atmosphere would be preferable for the reaction. To avoid using stoichiometric oxidants, we tested a dual catalytic system in which PQ-CF_3_ was catalytically regenerated by a cobaloxime complex. When 10 mol % PQ-CF_3_ and 6 mol % Co­(dmgH)_2_(py)Cl were used as catalysts under an Ar atmosphere, the cyclization delivered **2a** in 51% yield after 1 h of irradiation and reached completion after 3 h with 85% yield of the product (Table 1, entry 1). A control experiment with both a Co catalyst and an O_2_ atmosphere (Table 1, entry 5) resulted in faster reactivity, but even though all starting material was consumed already after 1 h, **2a** was detected in a lower yield (77%) due to undesired side reactions. We prioritized cleaner reactivity over faster reaction rates and carried on with the reaction optimization with a Co catalyst under an Ar atmosphere.

**Table 1:**
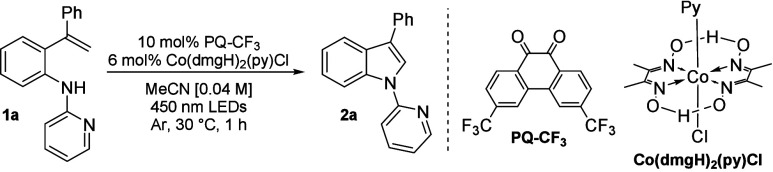
Effect of Deviation from the Standard Reaction Conditions^a^

entry	deviation from the standard conditions	yield^b^ (%)
1	none	51 (85)^c^
2	no light	0
3	no photocatalyst	0
4	no Co(dmgH)_2_(py)Cl	7
5	O_2_ atmosphere	77
6	O_2_ atmosphere, no Co(dmgH)_2_(py)Cl	55
7	at r.t.	32
8	EtOAc as the solvent	31^c^
9	DCE as the solvent	19^c^
10	0.02 M concentration	16
11	0.06 M concentration	35
12	5 mol % PQ-CF_3_	22
13	15 mol % PQ-CF_3_	30
14	10 mol % Co(dmgH)_2_(py)Cl	26
15	3 mol % Co(dmgH)_2_(py)Cl	7
16	5 mol % PQ-CF_3_ and 3 mol % Co(dmgH)_2_(py)Cl	30
17	PQ as the photocatalyst	37
18	anthraquinone as the photocatalyst	12
19	1 mmol scale, 112 h reaction time	75^d^

a Full reaction optimization in Tables S1–S5. The reactions were performed on a 0.10 mmol scale.

b NMR yield with 1,3,5-trimethoxybenzene as an internal standard.

c With a 3 h reaction time.

d Isolated yield after SiO_2_ chromatography, with a 440 nm Kessil PR 160L lamp as a light source.

Varying the solvent and the concentration did not have major effects on the reaction rate. However, the 5:3 ratio of the two catalysts proved to be important. Decreasing only the loading of the Co catalyst to 3 mol % dropped the yield of **2a** to 7%, but when the loading of PQ-CF_3_ was also decreased to 5 mol %, indole **2a** was achieved in 30% yield (Table 1, entries 15 and 16). Control experiments showed that the reaction did not proceed without light or without a photocatalyst, and the absence of a Co catalyst prevented the regeneration of PQ-CF_3_ (Table 1, entries 2–4). Furthermore, other tested photocatalysts did not provide improved yields (Table 1, entries 17 and 18). To our delight, the cyclization could be easily scaled up to 1 mmol, and **2a** was isolated in 75% yield after 112 h of irradiation with a 440 nm Kessil PR 160L lamp (Table 1, entry 19).

With the optimized reaction conditions in hand, we started studying the scope of the developed indole synthesis (Scheme 2). For the cyclization to reach completion with most of the substrates, the scale was increased to 0.20 mmol and the reaction time was prolonged to 48 h. First, we varied the aryl substituent on the amino group (R^1^) and obtained indoles **2a**–**2c** in very good yields (70–83%). However, N-methylated substrates **1z** and **1aa** did not provide indoles, but only partial demethylation was observed with **1z**. As the R^1^ aryl substituent did not have a dramatic effect on the outcome, we decided to continue with *N*-pyridyl-substituted compounds for easy access to free indoles.^17^

**Scheme 2:**
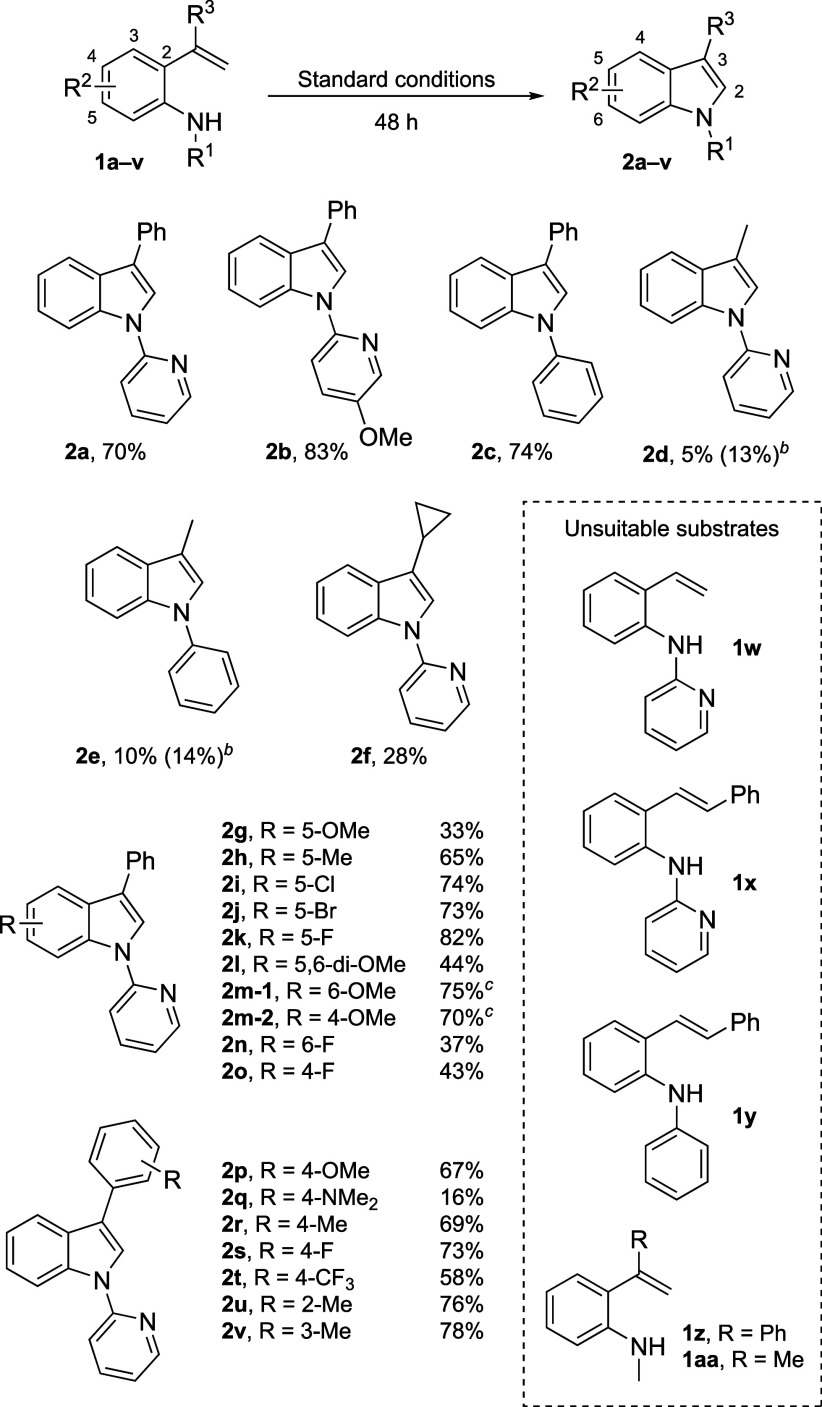
Reaction Scope Study^a^

a The reactions were performed on a 0.20 mmol scale, with isolated yields after SiO_2_ chromatography.
b NMR yield with 1,3,5-trimethoxybenzene as an internal standard.
c The starting material **1m** was an inseparable mixture of 5-MeO isomer **1m-1** and 3-MeO isomer **1m-2** in an 8:2 ratio. The yield is calculated based on the initial amount of the respective isomer in the mixture.

Different R^2^ substituents were tolerated under the reaction conditions. Indoles **2h**–**2k** with methyl or halide on the 5 position were received in good yields (65–82%). However, the yield of 5-MeO-substituted **2g** was only 33%, while 5,6-di-MeO-substituted indole **2l** was obtained in a slightly higher yield of 44%. In our earlier study, we observed that PQ catalyzed the oxidation of electron-rich benzylic alcohols more efficiently than PQ-CF_3_.^6^ Hence, we tested whether it would perform better in the cyclization of MeO-substituted **1g** and **1l**, but the received yields were nearly identical to those achieved with PQ-CF_3_.

Substrate **1m** contained an inseparable mixture of 5-MeO- and 3-MeO-substituted amines in an 8:2 ratio. Surprisingly, both 6-MeO-substituted indole **2m-1** and 4-MeO-substituted **2m-2** were achieved in 75 and 70% yields, respectively, when the yield was calculated from the amount of the respective isomer in the original starting material. Indoles **2n** and **2o** with 6-F and 4-F substituents showed completely opposite behavior, having significantly lower yields than 5-F-substituted **2k** (37 and 43% vs 82%, respectively).

Different R^3^ substituents led to diverse results. 3-Alkyl-substituted indoles **2d**–**2f** were obtained in poor yields (5–28%), whereas 3-aryl-substituted indoles **2p** and **2r**–**2v** containing either electron-donating or electron-withdrawing substituents were achieved in moderate to very good yields (58–78%). Only the reactivity of NMe_2_-substituted **1q** differed significantly from those of the other 3-aryl-substituted starting materials, delivering indole **2q** in 16% yield.

We attempted to widen the scope to 2-substituted indoles with substrates **1x** and **1y** but observed only *cis*–*trans* isomerization of the styryl group. The *cis* isomers of **1x** and **1y** were isolated in 30 and 29% yields, respectively, while 17 and 24% of the *trans* isomers were recovered. Moreover, 2-vinylamine **1w** produced no indole but underwent a [4 + 2] photocycloaddition with PQ-CF_3_, deactivating the catalyst. This kind of reactivity has been previously reported with PQ and several alkenes,^18^ and we confirmed the observation by performing a control experiment with PQ-CF_3_ and 1,1-diphenylethylene under standard conditions, which also led to the formation of a cycloadduct.

As an additional advantage, our method provided a simple route to free indoles by removing the pyridine group with base treatment according to previous literature.^17^ The deprotection of **2a** with NaOMe produced free indole **3a** in an 83% yield (Supporting Information). 3-Phenylindole **3a** exhibits antimicrobial properties as itself,^19^ but it can also be used in further transformations to access pyrazinoindole derivatives with useful pharmacological activities.^15,20,21^

We confirmed the radical nature of the developed reaction by performing the cyclization of **1a** under the standard conditions with 2 equiv of (2,2,6,6-tetramethylpiperidin-1-yl)­oxyl (TEMPO) or 2.9 equiv of 5,5-dimethyl-1-pyrroline-*N*-oxide (DMPO) as a radical scavenger. After 48 h of irradiation, **2a** was isolated in 46 and 2% yields, respectively, while the reaction with DMPO also produced adduct **S1** in 13% yield (Supporting Information). The adduct was also observed when the reaction was performed in the dark or in the absence of both catalysts, suggesting that it formed a thermal (3 + 2) cycloaddition between DMPO and **1a**.^22,23^

We carried out a Hammett σ_p_ correlation experiment to explore the reaction rates of electronically diverse 4-substituted amines **1a** and **1g**–**1k**. As the received nonlinear fit suggested that the substituents have a notable resonance effect to the mechanism,^24–26^ we used Hammett σ_p_^+^ constants to obtain a linear fit with a good coefficient of determination (*R* ^2^ = 0.93; Figure 1A). With resemblance to our previous study,^6^ the reaction rate was slowest with the most electron-rich substrate **1b** increasing toward more electron-deficient substrates (ρ = 0.72). The computed oxidation potentials of substrates **1a** and **1g**–**1k** showed a positive upward trend (Figure 1B); the most electron-rich substrates possessed the lowest oxidation potentials. Hence, the rate-determining step of the cyclization is unlikely to be related to single-electron oxidation of the substrate.

**Figure 1:**
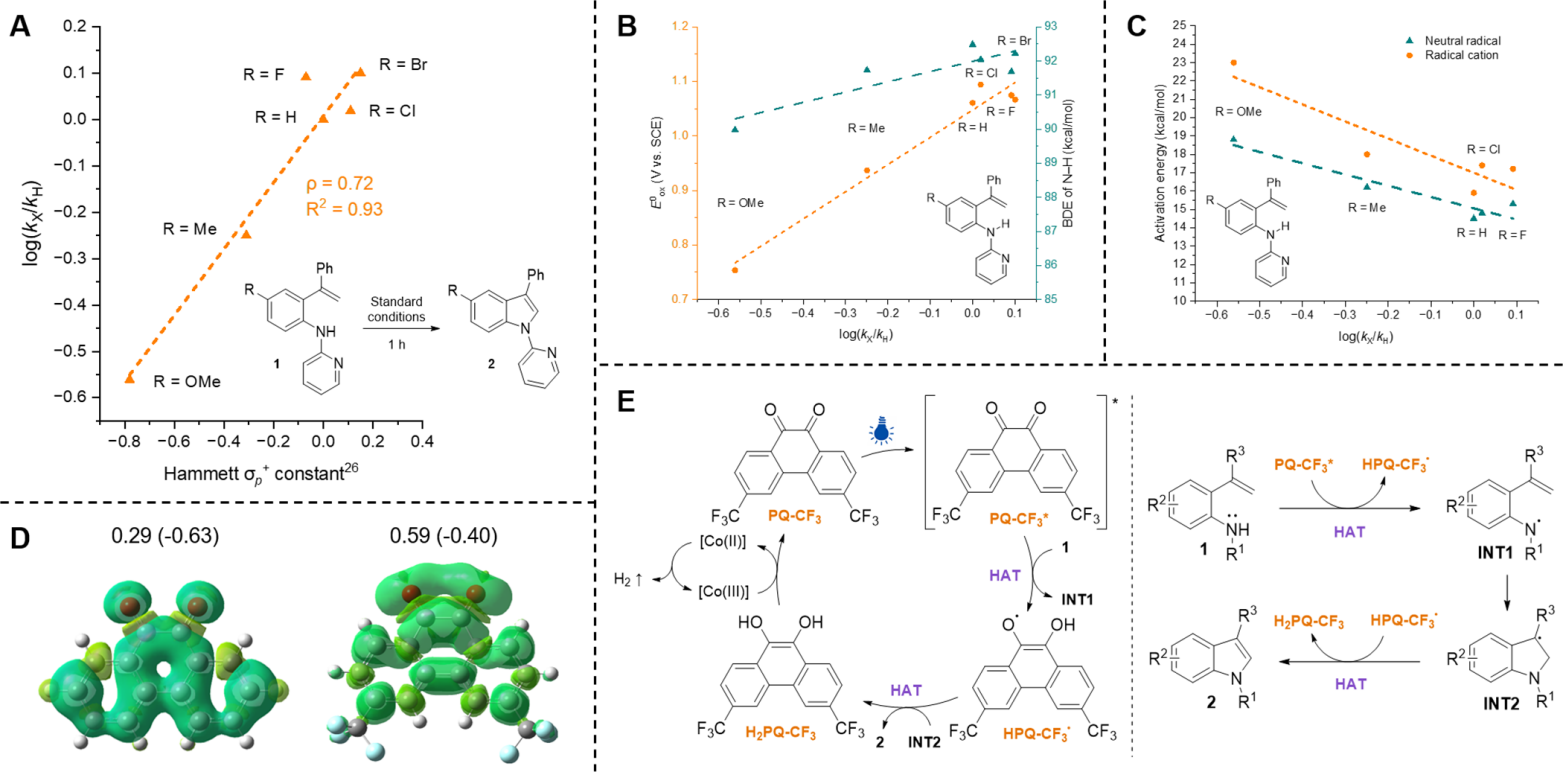
Mechanistic investigations. (A) Observed Hammett σ_p_^+^ correlations with substrates **1a** and **1g**–**1k** after 1 h of irradiation under standard conditions on a 0.10 mmol scale. NMR yields were measured with 1,3,5-trimethoxybenzene as an internal standard. (B) Correlation of the computed oxidation potential (V vs SCE) and the BDE of the N–H bond (kcal/mol) to the reaction rate with substrates **1a** and **1g**–**1k** (Tables S7 and S8 of the Supporting Information). (C) Correlation of the computed activation energy barrier to the reaction rate for the cyclization via neutral radical and radical cation pathways with substrates **1a**, **1g**–**1i**, and **1k** (Table S9 of the Supporting Information). (D) Computed Mulliken spin densities (charges) for the oxygen atoms of PQ (left) and PQ-CF_3_ (right). (E) Proposed reaction mechanism.

As the styryl substrates **1x** and **1y** resulted in only the *cis*–*trans* isomerization of the alkene under our reaction conditions, we rationalized that the isomerization could proceed either via a triplet state formed by energy transfer from the catalyst or by the catalyst-induced formation of the N radical via HAT. This result also contrasts with the Maity and Zheng report, in which they showed that similar substrates react readily under Ru photoredox catalysis, forming radical cations via SET (Scheme 1).^14^

For theoretical insight, we performed density functional theory (DFT) calculations to explore possible reaction routes (Supporting Information). We first computed the BDEs for the N–H bond of substrates **1a** and **1g**–**1k** (Figure 1B). The variation of BDEs was rather small with the substrates excluding **1g** (91.7–92.5 kcal/mol), exhibiting a very weak correlation to the reaction rates. Next, we computed the reaction profiles for the cyclization of both the neutral aminyl radical (HAT pathway) and the radical cation (SET pathway) intermediates of **1a** and **1g**–**1k** to examine the likelihood of both cyclization routes (Figure 1C). The HAT pathway offered regularly lower activation energy barriers in comparison to the corresponding SET route. For instance, the neutral radical cyclization barriers of MeO-, Me-, and Cl-substituted **1g**–**1i** ranged from 18.8 to 14.8 kcal/mol (Δ*G* ^⧧^), whereas the corresponding radical cation barriers had higher Δ*G* ^⧧^ (23.0–17.4 kcal/mol), suggesting that the cyclization via the latter route would be sluggish, particularly for electron-rich substrates.

In our previous photophysical study, we measured triplet emissions for PQ-CF_3_*.^6^ Herein, the triplet-state DFT computations of **1a** and the PQ-CF_3_* complex showed a very strong H-bonding character between N–H and quinone oxygens (Supporting Information). The reaction energy for the hydrogen transfer is slightly endergonic (Δ*G* = 2.8 kcal/mol) with a low reaction barrier (Δ*G* ^⧧^ = 2.1 kcal/mol), making the HAT route favorable for the N-radical formation (Figure S10 of the Supporting Information). Similar HAT abstraction energetics were also calculated for PQ (Table S10 of the Supporting Information). Notably, in the case of PQ-CF_3_, CF_3_ groups polarize the spin density on carbonyl oxygens bringing increased charge densities on the oxygen atoms (Figure 1D). We presume that this factor affects favorably on PQ-CF_3_’s affinity for hydrogen atoms.

Based on the experimental and theoretical evidence, we propose that the reaction starts with PQ-CF_3_*-induced HAT from the amino group of **1**, leading to the formation of HPQ-CF_3_^•^ and **INT1**, which subsequently cyclizes to the neutral radical intermediate **INT2** (Figure 1E). The presence of HPQ-CF_3_^•^ was supported by UV–vis spectroscopic studies (Supporting Information). A second HAT yields indole **2** as the product. The reduced photocatalyst H_2_PQ-CF_3_ is regenerated to PQ-CF_3_ by the Co catalytic cycle producing H_2_ as a side product (Supporting Information).

In conclusion, the developed cooperative photoredox and Co catalytic pathway produces 3-aryl-*N*-pyridyl-indoles generally in very good yields. The removal of the pyridyl group with base treatment affords free indoles in high yields. The mechanistic investigations indicated that PQ-CF_3_ induces a rarely utilized direct N–H activation via HAT, making it an interesting alternative for other C–N bond formation reactions.

## Supplementary Material





## Data Availability

The data underlying this study are available in the published article and its Supporting Information.

## Notes

Experimental procedures, full reaction optimization, mechanistic studies, computational procedures and details, and NMR spectra for all compounds (PDF)
PQ-CF_3_ structure (XYZ)

## †

Instituto de Química Médica, Consejo Superior de Investigaciones Científicas (CSIC), 28006 Madrid, Spain
