# Supplementary material for: Photoredox Catalytic Synthesis of Indoles via Direct N–H Activation to Generate Putative Aminyl Radicals
Source: Org Lett. 2025 Sep 8;27(37):10537–41. doi: 10.1021/acs.orglett.5c03410 (PMC12455651; doi:10.1021/acs.orglett.5c03410)

Supporting information

Photoredox Catalytic Synthesis of Indoles via Direct N–H Activation to Generate Putative Aminyl Radicals

Juulia Talvitie, Andrés Mollar-Cuni, Jarkko Nyman, Juho Koivula, Lisa Hendrickx, Pedro Muñoz Rodríguez, and Juho Helaja*

Department of Chemistry, University of Helsinki, A. I. Virtasen aukio 1, 00014 Helsinki, Finland

*Corresponding author

- E-mail: juho.helaja@helsinki.fi

Contents

General Information **3**

Synthesis and Characterization of the Starting Materials **5**

General Procedure A 5

General Procedure B 5

Optimization of Reaction Conditions **17**

Synthesis and Characterization of Indoles 2a–2v and 3a **19**

General Procedure C 19

Unsuitable Substrates 28

Mechanistic Studies **30**

Radical Trapping with TEMPO and DMPO 30

Reaction Rate Measurements for Hammett Correlation Experiment 31

H_2 Detection Experiment 31

UV-Vis Spectroscopic Measurements 32

Computational Studies **36**

Bibliography **41**

Copies of NMR Spectra **43**

General Information

All reagents and solvents were purchased from commercial sources (Acros Organics, Alfa Aesar, BLDpharm, Fluorochem, Merck, Sigma-Aldrich, TCI and VWR) and used without further purification. PQ-CF_3[1] and 2-(arylvinyl)anilines[2,3] for the preparation of the substrates were synthesized according to literature procedures. Anhydrous DCM and DMSO were dried over 4Å sieves. Glassware was stored in ambient conditions prior to use. NMR spectra were recorded at 25 °C on Bruker Avance Neo 400 MHz and 500 MHz spectrometers. Acetone-d_6 and TMS-containing $CDCl_3$ were used as deuterated solvents with solvent signals (2.05, 29.84 ppm and 0.00 (from TMS), 77.16 ppm, respectively) as references to chemical shifts. NMR yields were calculated with 1,3,5-trimethoxybenzene as an internal standard. Structural assignments were made with additional information from gHSQC experiments. Thin-layer chromatography (TLC) was conducted using silica gel GF254 with fluorescence indicator (254 nm). Chromatographic separations were performed with VWR silica gel (230-300 mesh). UV-Vis spectra were measured with Varian Cary 50 UV-Visible spectrophotometer using standard 10 mm quartz cuvettes. High-resolution mass spectra (HRMS) were obtained on a Jeol MStation JMS-700 (EI) instrument with a quadrupole mass analyzer. The photoreaction set up is shown in Figure S1. The light source was 3 x 3W ProLight Opto royal blue (λ_{max} 450 nm) LEDs which were positioned on the bottom of the reaction vial at a 4 mm distance. The UV-Vis emission spectrum of the LEDs is shown in Figure S2. No filters were used. The measured light power of 3 LEDs was 1.03 W. The reaction stand was a custom-made aluminum block with build-in water cooling.

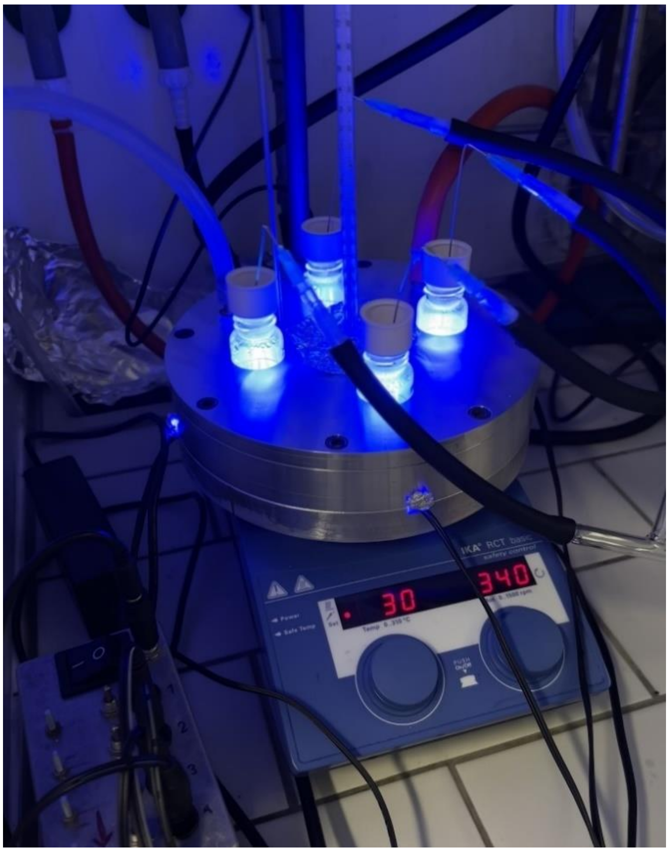

Figure S1. The experimental set-up of the photoreactions.

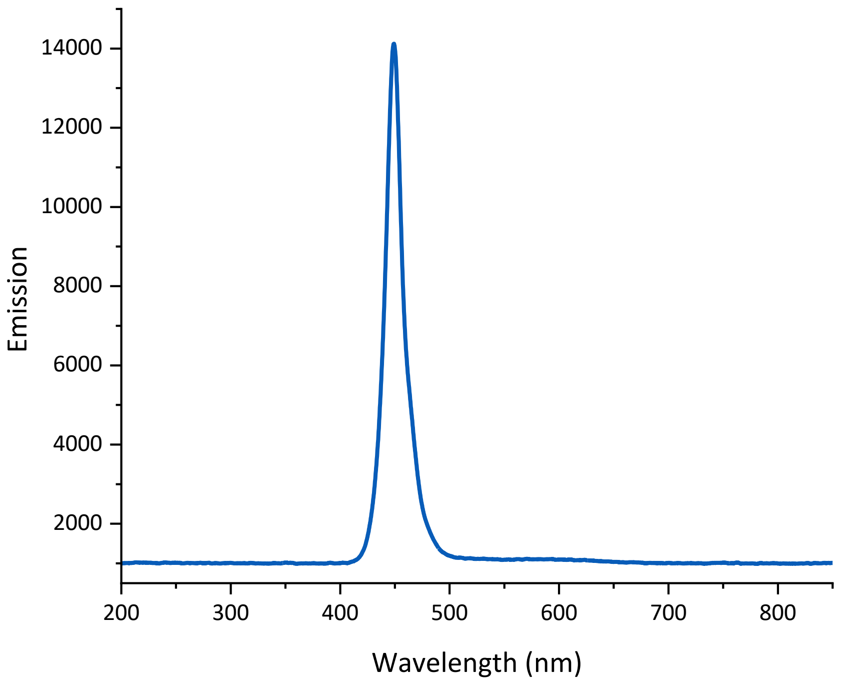

Figure S2. UV-Vis emission spectrum of the used LEDs.

Synthesis and Characterization of the Starting Materials

General Procedure A

The synthesis of amines **1a**–**b**, **1d**, and **1f**–***trans*-1x** was performed following a modified literature procedure.[4] A two-neck flask equipped with a stirrer bar and a condenser was loaded with $Pd(OAc)_2$ (6 mol%) and 1,1′-bis(diphenylphosphino)ferrocene (dppf, 18 mol%). The flask was evacuated and backfilled with argon three times. Water (0.4 equiv) and *t*-BuOH (0.4 M) were added to the flask and the mixture was bubbled with argon for 30 min. The catalyst was activated by stirring the reaction mixture at 110 °C in an oil bath for 30 min. The flask was lifted from the oil bath, and Cs_2CO_3 (1.44 equiv), respective aniline (1 equiv), and 2-chloropyridine (1.3 equiv) were added to the mixture. If the aniline was an oil, it was dissolved in a small amount of *t*-BuOH before addition. The mixture was bubbled with argon for further 30 min and stirred at 110 °C in an oil bath for 2–21 h until the reaction had finished according to ^1H NMR. The mixture was cooled down, filtered through Celite and washed with EtOAc. The solvents were evaporated on a rotary evaporator and the crude product was purified with flash chromatography on silica gel.

General Procedure B

The synthesis of amines **1c** and ***trans*-1y** was performed following a modified literature procedure.[5] A mixture of phenylboronic acid (2 equiv), copper acetate monohydrate (1.5 equiv), 4 Å molecular sieves (1.15 g/mmol) and anhydrous DCM (0.1 M) were stirred at r.t. for 5 minutes, after which it was added with 2-styrylaniline derivative (1 equiv), and Et_3N (2 equiv). The reaction mixture was stirred overnight at r.t. and filtered through Celite and silica with DCM. The solvents were evaporated on a rotary evaporator and the crude product was purified with flash chromatography on silica gel.

***N*-(2-(1-Phenylvinyl)phenyl)pyridin-2-amine (1a)**

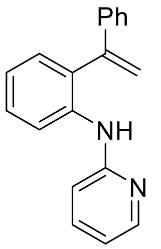

The product was prepared from 2-(1-phenylvinyl)aniline (943 mg, 4.83 mmol) and 2-chloropyridine (0.59 mL, 6.28 mmol) according to the general procedure A. The reaction time was 2 h. The product was isolated with flash chromatography (silica gel, 5:1 n-Hex/EtOAc) as a yellow oil (1.11 g, 4.08 mmol, 85%).

^1H NMR (400 MHz, $CDCl_3$) δ 8.09 (ddd, *J* = 5.0, 2.0, 0.9 Hz, 1H), 7.83 (dd, *J* = 8.2, 1.2 Hz, 1H), 7.40 – 7.19 (m, 8H), 7.08 (td, *J* = 7.4, 1.2 Hz, 1H), 6.64 (ddd, *J* = 7.2, 5.0, 1.0 Hz, 1H), 6.59 (d, *J* = 8.5 Hz, 1H), 6.25 (s, 1H), 5.84 (d, *J* = 1.3 Hz, 1H), 5.36 (d, *J* = 1.3 Hz, 1H).

^{13}C NMR (101 MHz, $CDCl_3$) δ 155.9 (C), 148.3 (CH), 147.0 (C), 139.8 (C), 138.2 (C), 137.5 (CH), 133.2 (C), 131.0 (CH), 128.70 (2×CH), 128.65 (CH), 128.3 (CH), 126.6 (2×CH), 122.8 (CH), 120.8 (CH), 116.8 (CH_2), 115.1 (CH), 108.8 (CH).

HRMS (EI) *m/z*: [M^+] calcd for $C_{19}H_{16}N_2$ 272.1313, found: 272.1320.

5-Methoxy-*N*-(2-(1-phenylvinyl)phenyl)pyridin-2-amine (1b)

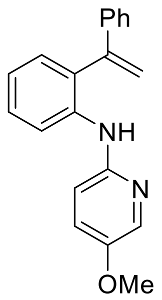

The product was prepared from 2-(1-phenylvinyl)aniline (323 mg, 1.65 mmol) and 2-chloro-5-methoxypyridine (0.25 mL, 2.62 mmol) according to the general procedure A. The reaction time was 2 h. The product was isolated with flash chromatography (silica gel, 5:1 n-Hex/EtOAc) as a yellow oil (337 g, 1.11 mmol, 67%).

^{1}H NMR (400 MHz, $CDCl_3$) δ 7.83 (d, *J* = 3.0 Hz, 1H), 7.77 (d, *J* = 8.3 Hz, 1H), 7.37 – 7.20 (m, 7H), 7.07 – 6.98 (m, 2H), 6.60 (d, *J* = 9.0 Hz, 1H), 6.13 (s, 1H), 5.85 (d, *J* = 1.3 Hz, 1H), 5.37 (d, *J* = 1.3 Hz, 1H), 3.77 (s, 3H).

^{13}C NMR (101 MHz, $CDCl_3$) δ 150.2 (C), 150.1 (C), 147.0 (C), 139.7 (C), 139.2 (C), 133.7 (CH), 131.9 (C), 131.0 (CH), 128.70 (2×CH), 128.66 (CH), 128.3 (CH), 126.6 (2×CH), 124.9 (CH), 121.7 (CH), 118.9 (CH), 116.7 (CH_2), 110.2 (CH), 56.3 (CH_3).

HRMS (EI) *m/z*: [M^+] calcd for $C_{20}H_{18}N_2O$ 302.1419, found: 302.1425.

***N*-Phenyl-2-(1-phenylvinyl)aniline (1c)**

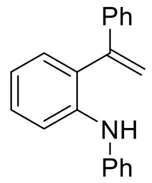

The product was prepared from 2-(1-phenylvinyl)aniline (300 mg, 1.54 mmol) and phenylboronic acid (391 mg, 3.2 mmol) according to the general procedure B. The reaction time was 24 h. The product was isolated with flash chromatography (silica gel, 10:1 n-Hex/EtOAc) as a pale yellow solid (135 mg, 0.50 mmol, 30%).

^{1}H NMR (400 MHz, acetone-d_6) δ 7.38 – 7.19 (m, 8H), 7.13 (dd, *J* = 8.6, 7.4 Hz, 2H), 6.99 (ddd, *J* = 7.6, 6.6, 1.9 Hz, 1H), 6.89 (dd, *J* = 8.7, 1.1 Hz, 2H), 6.79 (tt, *J* = 7.3, 1.1 Hz, 1H), 6.15 (s, 1H), 5.83 (d, *J* = 1.4 Hz, 1H), 5.34 (d, *J* = 1.3 Hz, 1H).

^{13}C NMR (101 MHz, acetone-d_6) δ 148.2, 144.7, 141.9, 140.9, 133.0, 131.9, 129.8, 129.3, 129.2, 128.7, 127.3, 121.8, 121.2, 118.8, 118.5, 116.7.

The characterization data was in agreement with the previous literature.[6]

***N*-(2-Isopropenylphenyl)pyridin-2-amine (1d)**

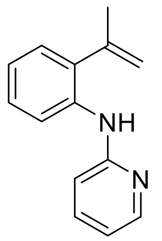

The product was prepared from 2-isopropenylaniline (0.27 mL, 2.00 mmol) and 2-chloropyridine (0.25 mL, 2.64 mmol) according to the general procedure A. The reaction time was 2 h. The product was isolated with flash chromatography (silica gel, 5:1 n-Hex/EtOAc) as an off-white solid (303 mg, 1.44 mmol, 72%).

^{1}H NMR (400 MHz, $CDCl_3$) δ 8.20 (ddd, *J* = 5.0, 2.0, 0.9 Hz, 1H), 7.69 (dd, *J* = 8.1, 1.2 Hz, 1H), 7.48 (ddd, *J* = 8.8, 7.2, 2.0 Hz, 1H), 7.28 – 7.23 (m, 1H), 7.19 (dd, *J* = 7.6, 1.5 Hz, 1H), 7.03 (td, *J* = 7.5, 1.2 Hz, 1H), 6.85 (dt, *J* = 8.4, 1.0 Hz, 1H), 6.73 (ddd, *J* = 7.2, 5.0, 0.9 Hz, 1H), 6.67 (s, 1H), 5.30 (p, *J* = 1.6 Hz, 1H), 5.04 (dq, *J* = 1.9, 0.9 Hz, 1H), 2.04 (dd, *J* = 1.5, 0.9 Hz, 3H).

^{13}C NMR (101 MHz, $CDCl_3$) δ 156.2 (C), 148.4 (CH), 143.8 (C), 137.9 (CH), 136.8 (C), 135.7 (C), 128.9 (CH), 127.9 (CH), 122.9 (CH), 120.7 (CH), 116.7 (CH_2), 115.1 (CH), 108.5 (CH), 24.1 (CH_3).

HRMS (EI) *m/z*: [M^+] calcd for $C_{14}H_{14}N_2$ 210.1157, found: 210.1155.

N-Phenyl-2-isopropenylaniline (1e)

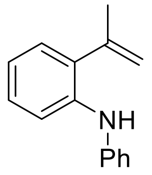

The product was prepared according to a modified literature procedure.[7] A mixture of phenylboronic acid (277 mg, 2.27 mmol, 1.2 equiv), myristic acid (172 mg, 0.75 mmol, 40 mol%), copper acetate monohydrate (70.6 mg, 0.35 mmol, 19 mol%), 4 Å molecular sieves (2.0 g), and toluene (3.8 mL) were stirred at r.t. for 15 minutes, after which it was added with 2-isopropenylaniline (0.253 mL, 1.86 mmol, 1 equiv), and 2,6-lutidine (0.216 mL, 1.86 mmol, 1 equiv). The reaction mixture was stirred for 5 days at r.t. and filtered through Celite and silica with DCM. The solvents were evaporated on a rotary evaporator. The product was isolated with flash chromatography (silica gel, 80:1 n-Hex/EtOAc) as a yellow oil (290 mg, 1.39 mmol, 74%).

^{1}H NMR (400 MHz, $CDCl_3$) δ 7.31 (d, *J* = 7.8 Hz, 1H), 7.28 – 7.23 (m, 2H), 7.19 – 7.13 (m, 2H), 7.06 (dd, *J* = 8.6, 1.1 Hz, 2H), 6.95 – 6.88 (m, 2H), 5.85 (s, 1H), 5.29 (dt, *J* = 3.1, 1.5 Hz, 1H), 5.06 (dd, *J* = 2.1, 1.0 Hz, 1H), 2.06 (dd, *J* = 1.6, 0.9 Hz, 3H).

^{13}C NMR (101 MHz, $CDCl_3$) δ 144.1, 143.6, 139.5, 133.7, 129.5, 128.9, 127.8, 121.1, 120.8, 118.2, 117.3, 116.3, 24.1.

The characterization data was in agreement with the previous literature.[6]

N-(2-(1-Cyclopropylvinyl)phenyl)pyridin-2-amine (1f)

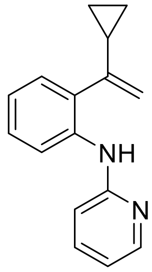

The product was prepared from 2-(1-cyclopropylvinyl)aniline (300 mg, 1.88 mmol) and 2-chloropyridine (0.23 mL, 2.44 mmol) according to the general procedure A. The reaction time was 2 h. The product was isolated with flash chromatography (silica gel, 10:1 n-Hex/EtOAc) as a yellow oil (345 mg, 1.46 mmol, 78%).

^{1}H NMR (400 MHz, $CDCl_3$) δ 8.21 (ddd, *J* = 5.0, 1.9, 0.9 Hz, 1H), 7.77 (dd, *J* = 8.2, 1.1 Hz, 1H), 7.48 (ddd, *J* = 8.8, 7.2, 2.0 Hz, 1H), 7.30 – 7.22 (m, 1H), 7.10 (dd, *J* = 7.5, 1.7 Hz, 1H), 6.98 (td, *J* = 7.4, 1.2 Hz, 1H), 6.88 (d, *J* = 8.4 Hz, 1H), 6.73 (ddd, *J* = 7.2, 5.0, 0.9 Hz, 1H), 6.70 (s, 1H), 5.25 (dd, *J* = 1.7, 0.7 Hz, 1H), 4.96 (d, *J* = 1.6 Hz, 1H), 1.63 (tt, *J* = 8.3, 5.2 Hz, 1H), 0.72 – 0.64 (m, 2H), 0.47 – 0.40 (m, 2H).

^{13}C NMR (101 MHz, $CDCl_3$) δ 156.0 (C), 149.2 (C), 148.6 (CH), 137.73 (CH), 137.68 (C), 132.3 (C), 130.0 (CH), 128.0 (CH), 122.0 (CH), 119.6 (CH), 115.2 (CH), 113.1 (CH_2), 108.7 (CH), 17.1 (CH), 6.7 ($2xCH_2$).

HRMS (EI) *m/z*: [M^+] calcd for $C_{16}H_{16}N_2$ 236.1313, found: 236.1316.

N-(4-Methoxy-2-(1-phenylvinyl)phenyl)pyridin-2-amine (1g)

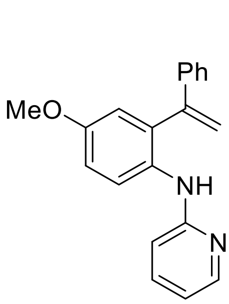

The product was prepared from 4-methoxy-2-(1-phenylvinyl)aniline (1.13 g, 5.00 mmol) and 2-chloropyridine (0.62 mL, 6.55 mmol) according to the general procedure A. The reaction time was 17 h. The product was isolated with flash chromatography (silica gel, 10:1→1:1 n-Hex/EtOAc) as a yellow solid (1.36 g, 4.49 mmol, 90%).

^{1}H NMR (400 MHz, $CDCl_3$) δ 8.01 (ddd, *J* = 5.0, 1.9, 0.9 Hz, 1H), 7.55 (d, *J* = 8.7 Hz, 1H), 7.33 – 7.16 (m, 6H), 6.92 (dd, *J* = 8.7, 3.0 Hz, 1H), 6.89 (d, *J* = 2.9 Hz, 1H),

6.56 (ddd, J = 7.2, 5.0, 0.9 Hz, 1H), 6.42 (dt, J = 8.5, 0.9 Hz, 1H), 5.92 (s, 1H), 5.75 (d, J = 1.3 Hz, 1H), 5.31 (d, J = 1.2 Hz, 1H), 3.82 (s, 3H).

^{13}C NMR (101 MHz, $CDCl_3$) δ 157.0 (C), 156.2 (C), 148.2 (CH), 147.2 (C), 139.7 (C), 137.3 (CH), 136.9 (C), 131.0 (C), 128.6 (2×CH), 128.1 (CH), 126.5 (2×CH), 125.1 (CH), 116.4 (CH_2), 116.3 (CH), 114.2 (CH), 114.0 (CH), 107.5 (CH), 55.7 (CH_3).

HRMS (EI) *m/z*: [M^+] calcd for $C_{20}H_{18}N_2O$ 302.1419, found: 302.1426.

N-(4-Methyl-2-(1-phenylvinyl)phenyl)pyridin-2-amine (1h)

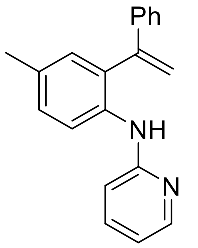

The product was prepared from 4-methyl-2-(1-phenylvinyl)aniline (419 mg, 2.00 mmol) and 2-chloropyridine (0.25 mL, 2.64 mmol) according to the general procedure A. The reaction time was 17 h. The product was isolated with flash chromatography (silica gel, 5:1 n-Hex/EtOAc) as an off-white solid (549 mg, 1.92 mmol, 96%).

^{1}H NMR (400 MHz, $CDCl_3$) δ 8.05 (dd, J = 5.0, 1.2 Hz, 1H), 7.61 (d, J = 8.1 Hz, 1H), 7.34 (ddd, J = 8.8, 7.2, 2.0 Hz, 1H), 7.31 – 7.20 (m, 5H), 7.16 (dd, J = 8.2, 2.2 Hz, 1H), 7.11 (d, J = 2.1 Hz, 1H), 6.60 (dd, J = 7.3, 5.0 Hz, 1H), 6.55 (d, J = 8.4 Hz, 1H), 6.11 (s, 1H), 5.79 (d, J = 1.3 Hz, 1H), 5.32 (d, J = 1.3 Hz, 1H), 2.35 (s, 3H).

^{13}C NMR (101 MHz, $CDCl_3$) δ 156.3 (C), 148.3 (CH), 147.2 (C), 139.9 (C), 137.4 (CH), 135.5 (C), 134.0 (C), 132.7 (C), 131.5 (CH), 129.2 (CH), 128.6 (2×CH), 128.1 (CH), 126.5 (2×CH), 121.9 (CH), 116.4 (CH_2), 114.6 (CH), 108.1 (CH), 20.9 (CH_3).

HRMS (EI) *m/z*: [M^+] calcd for $C_{20}H_{18}N_2$ 286.1470, found: 286.1467.

N-(4-Chloro-2-(1-phenylvinyl)phenyl)pyridin-2-amine (1i)

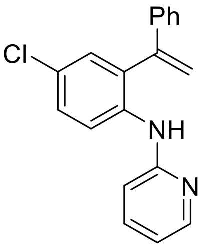

The product was prepared from 4-chloro-2-(1-phenylvinyl)aniline (232 mg, 1.01 mmol) and 2-chloropyridine (0.12 mL, 1.27 mmol) according to the general procedure A. The reaction time was 17 h. The product was isolated with flash chromatography (silica gel, 10:1→5:1 n-Hex/EtOAc) as a yellow solid (249 mg, 0.81 mmol, 80%).

^{1}H NMR (400 MHz, $CDCl_3$) δ 8.11 (ddd, J = 5.0, 1.9, 0.9 Hz, 1H), 7.90 (s, 1H), 7.38 (ddd, J = 8.4, 7.2, 1.9 Hz, 1H), 7.35 – 7.20 (m, 7H), 6.67 (ddd, J = 7.2, 5.0, 0.9 Hz, 1H), 6.47 (dt, J = 8.4, 0.9 Hz, 1H), 6.18 (s, 1H), 5.87 (d, J = 1.1 Hz, 1H), 5.38 (d, J = 1.1 Hz, 1H).

^{13}C NMR (101 MHz, $CDCl_3$) δ 155.5 (C), 148.2 (CH), 146.0 (C), 139.0 (C), 137.6 (CH), 137.0 (C), 134.1 (C), 130.5 (CH), 128.9 (2×CH), 128.57 (CH), 128.55 (CH), 127.2 (C), 126.6 (2×CH), 121.7 (CH), 117.5 (CH_2), 115.5 (CH), 109.3 (CH).

HRMS (EI) *m/z*: [M^+] calcd for $C_{19}H_{15}ClN_2$ 306.0924, found: 306.0911.

N-(4-Bromo-2-(1-phenylvinyl)phenyl)pyridin-2-amine (1j)

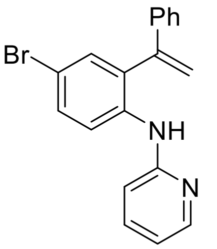

The product was prepared from 4-bromo-2-(1-phenylvinyl)aniline (1.20 g, 4.36 mmol) and 2-chloropyridine (0.54 mL, 5.71 mmol) according to the general procedure A. The reaction time was 17 h. The product was isolated with flash chromatography (silica gel, 10:1→5:1 n-Hex/EtOAc) as a pale orange solid (784 mg, 2.23 mmol, 51%). The reaction produced some **1a** as a side product which could not be chromatographically separated from **1j**. The final product contained 6 wt% **1a**.

^{1}H NMR (400 MHz, $CDCl_3$) δ 8.11 (dd, *J* = 5.0, 1.1 Hz, 1H), 7.88 (d, *J* = 8.7 Hz, 1H), 7.45 (dd, *J* = 8.7, 2.5 Hz, 1H), 7.41 – 7.22 (m, 7H), 6.68 (ddd, *J* = 7.2, 5.0, 0.9 Hz, 1H), 6.48 (d, *J* = 8.4 Hz, 1H), 6.19 (s, 1H), 5.87 (d, *J* = 1.1 Hz, 1H), 5.38 (d, *J* = 1.1 Hz, 1H).

^{13}C NMR (101 MHz, $CDCl_3$) δ 155.3 (C), 148.2 (CH), 145.8 (C), 139.0 (C), 137.6 (CH), 137.5 (C), 134.3 (C), 133.3 (CH), 131.5 (CH), 128.9 (2×CH), 128.6 (CH), 126.5 (2×CH), 121.8 (CH), 117.6 (CH), 115.5 (CH_2), 114.6 (CH), 109.4 (CH).

HRMS (EI) *m/z*: [M^+] calcd for $C_{19}H_{15}BrN_2$ 350.0419, found: 350.0413.

N-(4-Fluoro-2-(1-phenylvinyl)phenyl)pyridin-2-amine (1k)

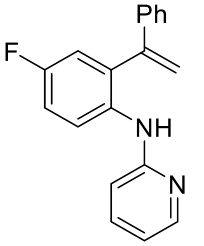

The product was prepared from 4-fluoro-2-(1-phenylvinyl)aniline (405 mg, 1.90 mmol) and 2-chloropyridine (0.23 mL, 2.43 mmol) according to the general procedure A. The reaction time was 17 h. The product was isolated with flash chromatography (silica gel, 10:1→5:1 n-Hex/EtOAc) as a brown solid (472 mg, 1.63 mmol, 86%).

^{1}H NMR (400 MHz, $CDCl_3$) δ 8.06 (ddd, *J* = 5.0, 1.9, 0.9 Hz, 1H), 7.77 (dd, *J* = 8.8, 5.2 Hz, 1H), 7.35 (ddd, *J* = 8.4, 7.2, 1.9 Hz, 1H), 7.30 – 7.22 (m, 5H), 7.10 – 7.01 (m, 2H), 6.63 (ddd, *J* = 7.2, 5.0, 0.9 Hz, 1H), 6.43 (dt, *J* = 8.4, 0.9 Hz, 1H), 6.03 (s, 1H), 5.82 (d, *J* = 1.1 Hz, 1H), 5.35 (d, *J* = 1.1 Hz, 1H).

^{13}C NMR (101 MHz, $CDCl_3$) δ 158.7 (d, *J* = 242.7 Hz, CF), 156.2 (C), 148.2 (CH), 146.3 (d, *J* = 1.4 Hz, C), 139.2 (C), 137.5 (CH), 135.7 (d, *J* = 7.6 Hz, C), 134.2 (d, *J* = 2.7 Hz, C), 128.8 (2×CH), 128.4 (CH), 126.5 (2×CH), 123.6 (d, *J* = 8.0 Hz, CH), 117.5 (d, *J* = 22.7 Hz, CH), 117.2 (CH_2), 115.3 (d, *J* = 22.1 Hz, CH), 115.0 (CH), 108.4 (CH).

^{19}F NMR (376 MHz, $CDCl_3$) δ -120.2.

HRMS (EI) *m/z*: [M^+] calcd for $C_{19}H_{15}FN_2$ 290.1219, found: 290.1212.

_N_-(4,5-Dimethoxy-2-(1-phenylvinyl)phenyl)pyridin-2-amine (1l)

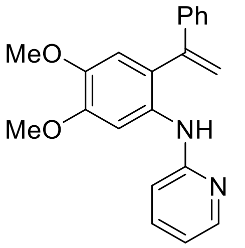

The product was prepared from 4,5-dimethoxy-2-(1-phenylvinyl)aniline (696 mg, 2.72 mmol) and 2-chloropyridine (0.35 mL, 3.70 mmol) according to the general procedure A. The reaction time was 19 h. The product was isolated with flash chromatography (silica gel, 10:1 → 5:1 n-Hex/EtOAc) as a pale yellow solid (719 g, 2.16 mmol, 79%).

^{1}H NMR (400 MHz, $CDCl_3$) δ 8.05 (ddd, *J* = 5.0, 1.9, 0.9 Hz, 1H), 7.38 – 7.18 (m, 7H), 6.82 (s, 1H), 6.60 (ddd, *J* = 7.2, 5.0, 0.9 Hz, 1H), 6.43 (d, *J* = 8.4 Hz, 1H), 5.99 (s, 1H), 5.75 (d, *J* = 1.4 Hz, 1H), 5.31 (d, *J* = 1.3 Hz, 1H), 3.90 (s, 3H), 3.87 (s, 3H).

^{13}C NMR (101 MHz, $CDCl_3$) δ 156.8 (C), 149.0 (C), 148.3 (CH), 147.0 (C), 145.4 (C), 140.2 (C), 137.4 (CH), 131.2 (C), 128.6 (2×CH), 128.1 (CH), 126.9 (C), 126.6 (2×CH), 116.4 (CH), 114.4 (CH), 113.9 (CH), 108.0 (CH), 107.5 (CH), 56.4 (CH_3), 56.2 (CH_3).

HRMS (EI) *m/z*: [M^+] calcd for $C_{21}H_{20}N_2O_2$ 332.1525, found: 332.1520.

_N_-(5-Methoxy-2-(1-phenylvinyl)phenyl)pyridin-2-amine (1m-1)

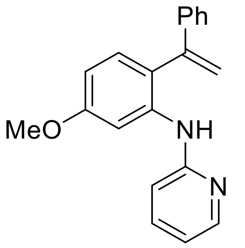

The product was prepared from 5-methoxy-2-(1-phenylvinyl)aniline (140 mg, 0.62 mmol, contained 20 mol% inseparable 3-methoxy-isomer) and 2-chloropyridine (0.08 mL, 0.87 mmol) according to the general procedure A. The reaction time was 22 h. The product was isolated with flash chromatography (silica gel, 10:1 → 5:1 n-Hex/EtOAc) as a light brown oil (143 g, 0.47 mmol, 76%) which contained 20 mol% inseparable *N*-(3-methoxy-2-(1-phenylvinyl)phenyl)pyridin-2-amine isomer **1m-2**.

^{1}H NMR (400 MHz, $CDCl_3$) δ 8.15 (dd, *J* = 5.0, 1.1 Hz, 1H), 7.59 – 7.53 (m, 1H), 7.43 (ddd, *J* = 8.7, 7.2, 1.9 Hz, 1H), 7.37 – 7.21 (m, 6H), 6.80 (d, *J* = 8.4 Hz, 1H), 6.69 (ddd, *J* = 7.2, 5.0, 1.0 Hz, 1H), 6.68 – 6.61 (m, 2H), 6.08 (d, *J* = 1.3 Hz, 1H), 5.32 (d, *J* = 1.2 Hz, 1H), 3.70 (s, 3H).

^{13}C NMR (101 MHz, $CDCl_3$) δ 157.9 (C), 155.8 (C), 148.4 (CH), 142.3 (C), 139.5 (C), 139.3 (C), 137.6 (CH), 128.8 (CH), 128.5 (2×CH), 128.0 (CH), 126.0 (2×CH), 121.0 (C), 117.2 (CH_2), 115.3 (CH), 112.0 (CH), 109.4 (CH), 105.1 (CH), 56.1 (CH_3).

HRMS (EI) *m/z*: [M^+] calcd for $C_{20}H_{18}N_2O$ 302.1419, found: 302.1432.

_N_-(5-Fluoro-2-(1-phenylvinyl)phenyl)pyridin-2-amine (1n)

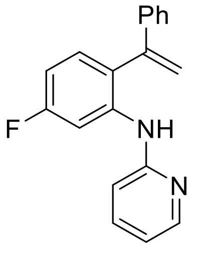

The product was prepared from 3-fluoro-2-(1-phenylvinyl)aniline (157 mg, 1.17 mmol) and 2-chloropyridine (0.09 mL, 0.96 mmol) according to the general procedure A. The reaction time was 17 h. The product was isolated with flash chromatography (silica gel, 10:1 n-Hex/EtOAc) as a brown solid (172 mg, 0.59 mmol, 80%).

^{1}H NMR (400 MHz, $CDCl_3$) δ 8.17 (ddd, *J* = 5.0, 1.9, 0.9 Hz, 1H), 7.83 (d, *J* = 8.3 Hz, 1H), 7.45 (ddd, *J* = 8.3, 7.2, 2.0 Hz, 1H), 7.40 – 7.33 (m, 2H), 7.33 – 7.23 (m, 4H), 6.78 (ddd, *J* = 9.2, 8.3, 1.0 Hz, 1H), 6.75 – 6.68 (m, 2H), 6.56 (s, 1H), 6.13 (d, *J* = 1.0 Hz, 1H), 5.43 (d, *J* = 1.0 Hz, 1H).

^{13}C NMR (101 MHz, $CDCl_3$) δ 160.6 (d, *J* = 244.2 Hz, CF), 155.3 (CH), 148.3 (C), 140.2 (d, *J* = 5.5 Hz, C), 139.7 (d, *J* = 1.4 Hz, C), 138.6 (C), 137.7 (CH), 129.2 (d, *J* = 9.9 Hz, CH), 128.8 (2×CH), 128.5 (CH), 126.1 (2×CH), 119.4 (d, *J* = 19.6 Hz, C), 118.8 (CH_2), 115.8 (CH), 114.4 (d, *J* = 3.0 Hz, CH), 109.9 (CH), 108.9 (d, *J* = 22.5 Hz, CH).

^{19}F NMR (376 MHz, $CDCl_3$) δ -113.3.

HRMS (EI) *m/z*: [M^+] calcd for $C_{19}H_{15}FN_2$ 290.1219, found: 290.1218.

N-(3-Fluoro-2-(1-phenylvinyl)phenyl)pyridin-2-amine (1o)

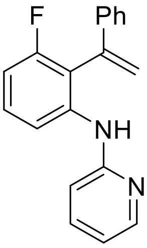

The product was prepared from 3-fluoro-2-(1-phenylvinyl)aniline (249 mg, 0.74 mmol) and 2-chloropyridine (0.14 mL, 1.52 mmol) according to the general procedure A. The reaction time was 17 h. The product was isolated with flash chromatography (silica gel, 10:1 n-Hex/EtOAc) as an orange oil (285 mg, 0.98 mmol, 84%). The product contained a small amount of inseparable impurity which remained from the aniline starting material.

^{1}H NMR (400 MHz, $CDCl_3$) δ 8.17 (ddd, *J* = 5.0, 2.0, 0.9 Hz, 1H), 7.94 (dd, *J* = 11.7, 2.6 Hz, 1H), 7.41 (ddd, *J* = 8.3, 7.2, 1.9 Hz, 1H), 7.37 – 7.26 (m, 5H), 7.18 (dd, *J* = 8.4, 6.6 Hz, 1H), 6.76 – 6.69 (m, 2H), 6.50 (dt, *J* = 8.4, 0.9 Hz, 1H), 6.38 (s, 1H), 5.88 (d, *J* = 1.3 Hz, 1H), 5.37 (d, *J* = 1.3 Hz, 1H).

^{13}C NMR (101 MHz, $CDCl_3$) δ 163.0 (d, *J* = 244.0 Hz, CF), 155.0 (C), 148.2 (CH), 146.1 (CH), 139.9 (d, *J* = 11.6 Hz, C), 139.5 (C), 137.6 (CH), 131.7 (d, *J* = 9.6 Hz, CH), 128.8 (2×CH), 128.5 (CH), 127.2 (d, *J* = 3.1 Hz, C), 126.6 (2×CH), 117.4 (CH_2), 115.8 (CH), 110.1 (CH), 108.4 (d, *J* = 21.8 Hz, CH), 106.2 (d, *J* = 26.9 Hz, CH).

^{19}F NMR (376 MHz, $CDCl_3$) δ -112.4.

HRMS (EI) *m/z*: [M^+] calcd for $C_{19}H_{15}FN_2$ 290.1219, found: 290.1226.

N-(2-(1-(4-Methoxyphenyl)vinyl)phenyl)pyridin-2-amine (1p)

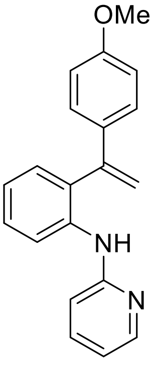

The product was prepared from 2-(1-(4-methoxyphenyl)vinyl)aniline (500 mg, 2.22 mmol) and 2-chloropyridine (0.30 mL, 3.17 mmol) according to the general procedure A. The reaction time was 18 h. The product was isolated with flash chromatography (silica gel, 10:1 → 5:1 n-Hex/EtOAc) as a yellow oil (477 mg, 1.58 mmol, 71%).

^{1}H NMR (400 MHz, $CDCl_3$) δ 8.11 (ddd, *J* = 5.0, 2.0, 0.9 Hz, 1H), 7.85 (dd, *J* = 8.2, 1.2 Hz, 1H), 7.43 – 7.30 (m, 2H), 7.30 – 7.22 (m, 3H), 7.07 (td, *J* = 7.5, 1.2 Hz, 1H), 6.82 – 6.74 (m, 2H), 6.65 (ddd, *J* = 7.2, 5.0, 1.0 Hz, 1H), 6.62 (d, *J* = 8.4 Hz, 1H), 6.31 (s, 1H), 5.75 (d, *J* = 1.3 Hz, 1H), 5.24 (d, *J* = 1.3 Hz, 1H), 3.76 (s, 3H).

^{13}C NMR (101 MHz, $CDCl_3$) δ 159.8 (C), 155.9 (C), 148.3 (CH), 146.3 (C), 138.2 (C), 137.5 (CH), 133.2 (C), 132.2 (C), 131.0 (CH), 128.5 (CH), 127.8 (2×CH), 122.6 (CH), 120.4 (CH), 115.1 (CH), 114.8 (CH_2), 114.1 (2×CH), 108.9 (CH), 55.4 (CH_3).

HRMS (EI) *m/z*: [M^+] calcd for $C_{20}H_{18}N_2O$ 302.1419, found: 302.1409.

N-(2-(1-(4-(Dimethylamino)phenyl)vinyl)phenyl)pyridin-2-amine (1q)

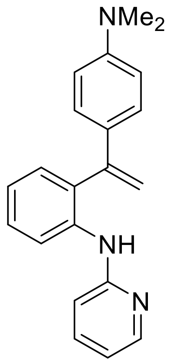

The product was prepared from 4-(1-(2-aminophenyl)vinyl)-*N,N*-dimethylaniline (1.12 g, 4.72 mmol) and 2-chloropyridine (0.60 mL, 6.34 mmol) according to the general procedure A. The reaction time was 18 h. The product was isolated with flash chromatography (silica gel, 10:1 → 5:1 n-Hex/EtOAc) as a pale yellow oil (1.03 g, 3.26 mmol, 76%) which contained 4 wt% of the corresponding indole **2q** as an impurity.

^{1}H NMR (400 MHz, $CDCl_3$) δ 8.12 (ddd, *J* = 5.0, 2.0, 0.9 Hz, 1H), 7.91 (dd, *J* = 8.3, 1.2 Hz, 1H), 7.38 (ddd, *J* = 8.4, 7.2, 1.9 Hz, 1H), 7.37 – 7.28 (m, 1H), 7.25 (dd, *J* = 7.4, 1.7 Hz, 1H), 7.21 (d, *J* = 8.9 Hz, 2H), 7.04 (td, *J* = 7.5, 1.2 Hz, 1H), 6.67 – 6.57 (m, 4H), 6.44 (s, 1H), 5.71 (d, *J* = 1.5 Hz, 1H), 5.13 (d, *J* = 1.4 Hz, 1H), 2.92 (s, 6H).

^{13}C NMR (101 MHz, $CDCl_3$) δ 156.0 (C), 150.6 (C), 148.3 (CH), 146.4 (C), 138.3 (C), 137.4 (CH), 133.3 (C), 130.9 (CH), 128.3 (CH), 127.5 (2×CH), 127.4 (C), 122.2 (CH), 119.8 (CH), 115.0 (CH), 112.7 (CH_2), 112.4 (2×CH), 109.2 (CH), 40.5 (2×CH_3).

HRMS (EI) *m/z*: [M^+] calcd for $C_{21}H_{21}N_3$ 315.1735, found: 315.1732.

N-(2-(1-(p-Tolyl)vinyl)phenyl)pyridin-2-amine (1r)

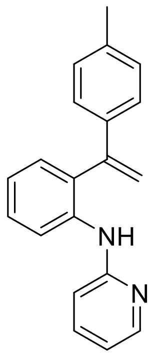

The product was prepared from 2-(1-(4-methylphenyl)vinyl)aniline (905 mg, 4.32 mmol) and 2-chloropyridine (0.49 mL, 5.18 mmol) according to the general procedure A. The reaction time was 18 h. The product was isolated with flash chromatography (silica gel, 10:1 → 5:1 → 3:1 n-Hex/EtOAc) as a yellow oil (879 mg, 3.07 mmol, 71%).

^{1}H NMR (400 MHz, $CDCl_3$) δ 8.10 (ddd, *J* = 5.0, 1.9, 0.9 Hz, 1H), 7.84 (dd, *J* = 8.2, 1.2 Hz, 1H), 7.42 – 7.30 (m, 2H), 7.26 (dd, *J* = 7.5, 1.8 Hz, 1H), 7.21 (d, *J* = 8.2 Hz, 2H), 7.09 – 7.04 (m, 3H), 6.65 (ddd, *J* = 7.2, 5.0, 1.0 Hz, 1H), 6.61 (dt, *J* = 8.5, 0.9 Hz, 1H), 6.29 (s, 1H), 5.81 (d, *J* = 1.3 Hz, 1H), 5.29 (d, *J* = 1.4 Hz, 1H), 2.29 (s, 3H).

^{13}C NMR (101 MHz, $CDCl_3$) δ 155.9 (C), 148.3 (CH), 146.7 (C), 138.21 (C), 138.16 (C), 137.5 (CH), 136.8 (C), 133.2 (C), 131.0 (CH), 129.4 (2×CH), 128.5 (CH), 126.5 (2×CH), 122.6 (CH), 120.5 (CH), 115.8 (CH_2), 115.0 (CH), 108.9 (CH), 21.3 (CH_3).

HRMS (EI) *m/z*: [M^+] calcd for $C_{20}H_{18}N_2$ 286.1470, found: 286.1465.

N-(2-(1-(4-Fluorophenyl)vinyl)phenyl)pyridin-2-amine (1s)

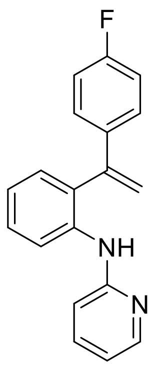

The product was prepared from 2-(1-(4-fluorophenyl)vinyl)aniline (489 mg, 2.29 mmol) and 2-chloropyridine (0.28 mL, 2.98 mmol) according to the general procedure A. The reaction time was 21 h. The product was isolated with flash chromatography (silica gel, 10:1 → 5:1 n-Hex/EtOAc) as a pale orange solid (567 mg, 1.95 mmol, 85%).

^{1}H NMR (400 MHz, $CDCl_3$) δ 8.09 (dd, *J* = 5.0, 1.0 Hz, 1H), 7.79 (d, *J* = 8.1 Hz, 1H), 7.43 – 7.31 (m, 2H), 7.31 – 7.23 (m, 3H), 7.09 (td, *J* = 7.4, 1.2 Hz, 1H), 6.91 (t, *J* = 8.7 Hz, 2H), 6.66 (ddd, *J* = 7.2, 5.0, 0.9 Hz, 1H), 6.62 (d, *J* = 8.4 Hz, 1H), 6.21 (s, 1H), 5.78 (d, *J* = 1.1 Hz, 1H), 5.34 (d, *J* = 1.1 Hz, 1H).

^{13}C NMR (101 MHz, $CDCl_3$) δ 162.8 (d, *J* = 247.6 Hz, CF), 155.8 (C), 148.4 (CH), 146.0 (C), 138.1 (C), 137.6 (CH), 135.9 (d, *J* = 3.3 Hz, C), 133.1 (C), 131.0 (CH), 128.8 (CH), 128.3 (d, *J* = 8.1 Hz, 2×CH), 122.9 (CH), 121.0 (CH), 116.5 (d, *J* = 1.7 Hz, CH_2), 115.5 (d, *J* = 21.5 Hz, 2×CH), 115.2 (CH), 108.6 (CH).

^{19}F NMR (376 MHz, $CDCl_3$) δ -114.0.

HRMS (EI) *m/z*: [M^+] calcd for $C_{19}H_{15}FN_2$ 290.1219, found: 290.1219.

***N*-(2-(1-(4-(Trifluoromethyl)phenyl)vinyl)phenyl)pyridin-2-amine (1t)**

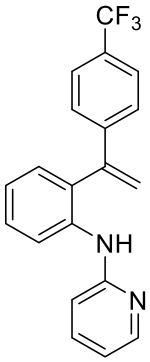

The product was prepared from 2-(1-(4-(trifluoromethyl)phenyl)vinyl)aniline (771 mg, 2.93 mmol) and 2-chloropyridine (0.40 mL, 4.23 mmol) according to the general procedure A. The reaction time was 19 h. The product was isolated with flash chromatography (silica gel, 10:1 → 5:1 n-Hex/EtOAc) as a pale yellow solid (840 mg, 2.47 mmol, 84%).

^{1}H NMR (400 MHz, $CDCl_3$) δ 8.06 (ddd, *J* = 5.0, 1.9, 0.9 Hz, 1H), 7.74 (dd, *J* = 8.1, 1.2 Hz, 1H), 7.45 (d, *J* = 8.1 Hz, 2H), 7.43 – 7.33 (m, 4H), 7.29 (dd, *J* = 7.6, 1.6 Hz, 1H), 7.13 (td, *J* = 7.5, 1.2 Hz, 1H), 6.65 (ddd, *J* = 7.2, 5.0, 1.0 Hz, 1H), 6.60 (dt, *J* = 8.3, 0.9 Hz, 1H), 6.13 (s, 1H), 5.90 (d, *J* = 1.0 Hz, 1H), 5.49 (d, *J* = 1.0 Hz, 1H).

^{13}C NMR (101 MHz, $CDCl_3$) δ 155.8 (C), 148.4 (CH), 146.1 (C), 143.4 (d, *J* = 1.7 Hz, C), 138.1 (C), 137.6 (CH), 133.1 (C), 131.1 (CH), 130.0 (q, *J* = 32.5 Hz, C), 129.1 (CH), 126.8 (2×CH), 125.6 (q, *J* = 3.7 Hz, 2×CH), 124.2 (q, *J* = 272.2 Hz, CF_3), 123.4 (CH), 122.8 (CH), 118.7 (CH), 115.3 (CH), 108.3 (CH).

^{19}F NMR (376 MHz, $CDCl_3$) δ -62.6.

HRMS (EI) *m/z*: [M^+] calcd for $C_{20}H_{15}F_3N_2$ 340.1187, found: 340.1175.

***N*-(2-(1-(*o*-Tolyl)vinyl)phenyl)pyridin-2-amine (1u)**

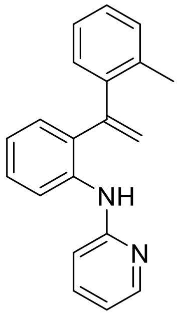

The product was prepared from 2-(1-(*o*-tolyl)vinyl)aniline (559 mg, 2.67 mmol) and 2-chloropyridine (0.33 mL, 3.47 mmol) according to the general procedure A. The reaction time was 21 h. The product was isolated with flash chromatography (silica gel, 10:1 → 5:1 n-Hex/EtOAc) as a pale yellow solid (552 mg, 1.93 mmol, 72%).

^{1}H NMR (400 MHz, $CDCl_3$) δ 8.12 (ddd, *J* = 5.0, 2.0, 0.9 Hz, 1H), 7.76 (dd, *J* = 8.1, 1.2 Hz, 1H), 7.38 (ddd, *J* = 8.5, 7.2, 1.9 Hz, 1H), 7.31 – 7.22 (m, 3H), 7.20 – 7.14 (m, 2H), 7.12 – 7.08 (m, 1H), 7.03 (td, *J* = 7.5, 1.2 Hz, 1H), 6.66 (ddd, *J* = 7.2, 5.0, 0.9 Hz, 1H), 6.51 (dt, *J* = 8.4, 0.9 Hz, 1H), 6.38 (s, 1H), 5.60 (d, *J* = 1.7 Hz, 1H), 5.48 (d, *J* = 1.7 Hz, 1H), 2.09 (s, 3H).

^{13}C NMR (101 MHz, $CDCl_3$) δ 156.1 (C), 148.4 (CH), 148.0 (C), 141.5 (C), 137.7 (C), 137.5 (CH), 135.7 (C), 133.9 (C), 130.9 (CH), 130.5 (CH), 129.6 (CH), 128.4 (CH), 127.9 (CH), 126.2 (CH), 123.1 (CH), 121.7 (CH), 120.0 (CH), 115.0 (CH), 108.5 (CH), 20.7 (CH_3).

HRMS (EI) *m/z*: [M^+] calcd for $C_{20}H_{18}N_2$ 286.1470, found: 286.1483.

N-(2-(1-(m-Tolyl)vinyl)phenyl)pyridin-2-amine (1v)

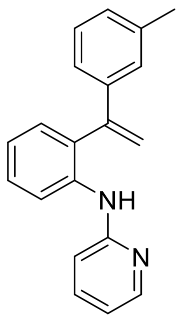

The product was prepared from 2-(1-(*m*-tolyl)vinyl)aniline (471 mg, 2.25 mmol) and 2-chloropyridine (0.28 mL, 2.92 mmol) according to the general procedure A. The reaction time was 21 h. The product was isolated with flash chromatography (silica gel, 10:1 → 5:1 n-Hex/EtOAc) as a yellow oil (463 mg, 1.62 mmol, 72%).

^{1}H NMR (400 MHz, $CDCl_3$) δ 8.09 (ddd, *J* = 5.0, 1.9, 0.9 Hz, 1H), 7.82 (dd, *J* = 8.2, 1.2 Hz, 1H), 7.42 – 7.31 (m, 2H), 7.28 (dd, *J* = 7.5, 1.6 Hz, 1H), 7.18 – 7.06 (m, 4H), 7.05 – 7.02 (m, 1H), 6.64 (ddd, *J* = 7.2, 5.0, 0.9 Hz, 1H), 6.59 (dt, *J* = 8.4, 1.0 Hz, 1H), 6.25 (s, 1H), 5.82 (d, *J* = 1.3 Hz, 1H), 5.34 (d, *J* = 1.3 Hz, 1H), 2.24 (s, 3H).

^{13}C NMR (126 MHz, $CDCl_3$) δ 156.0 (C), 148.3 (CH), 147.1 (C), 139.7 (C), 138.3 (C), 138.2 (C), 137.4 (CH), 133.4 (C), 131.0 (CH), 129.1 (CH), 128.62 (CH), 128.59 (CH), 127.3 (CH), 123.8 (CH), 122.8 (CH), 120.9 (CH), 116.6 (CH_2), 115.0 (CH), 108.7 (CH), 21.5 (CH_3).

HRMS (EI) *m/z*: [M^+] calcd for $C_{20}H_{18}N_2$ 286.1470, found: 286.1476.

N-(2-Vinylphenyl)pyridin-2-amine (1w)

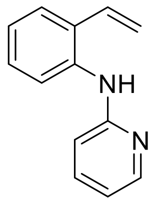

The product was prepared from 2-vinylaniline (0.50mL, 4.27 mmol) and 2-chloropyridine (0.50 mL, 5.28 mmol) according to the general procedure A. The reaction time was 19 h. The product was isolated with flash chromatography (silica gel, 10:1 → 5:1 n-Hex/EtOAc) as a pale pink solid (602 mg, 3.07 mmol, 72%).

^{1}H NMR (400 MHz, $CDCl_3$) δ 8.18 (ddd, *J* = 5.0, 1.9, 0.9 Hz, 1H), 7.55 (dd, *J* = 7.8, 1.6 Hz, 1H), 7.48 – 7.40 (m, 2H), 7.32 – 7.23 (m, 1H), 7.19 – 7.13 (m, 1H), 6.92 (dd, *J* = 17.5, 11.0 Hz, 1H), 6.70 (ddd, *J* = 7.2, 5.0, 1.0 Hz, 1H), 6.62 (dt, *J* = 8.4, 0.9 Hz, 1H), 6.50 (s, 1H), 5.72 (dd, *J* = 17.5, 1.4 Hz, 1H), 5.31 (dd, *J* = 11.0, 1.4 Hz, 1H).

^{13}C NMR (101 MHz, $CDCl_3$) δ 157.1, 148.6, 137.8, 137.5, 132.8, 132.5, 128.8, 127.0, 124.9, 123.9, 116.6, 114.9, 107.8.

The characterization data was in agreement with the previous literature.[8]

(E)-N-(2-Styrylphenyl)pyridin-2-amine (*trans*-1x)

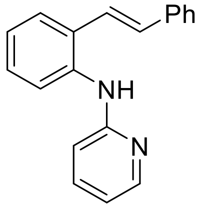

The product was prepared from 2-styrylaniline (200 mg, 1.02 mmol) and 2-chloropyridine (0.10 mL, 1.02 mmol) according to the slightly modified general procedure A with 9 mol% $Pd(OAc)_2$, 25 mol% of dppf, 2.03 equiv of Cs_2CO_3, 0.5 equiv of H_2O, and 1 equiv of 2-chloropyridine. The reaction time was 2 h. The product was isolated with flash chromatography (silica gel, 10:1→5:1 n-Hex/EtOAc) as an off-white solid (198 mg, 0.73 mmol, 71%).

^{1}H NMR (400 MHz, $CDCl_3$) δ 8.19 (ddd, *J* = 5.0, 2.0, 0.9 Hz, 1H), 7.68 (dd, *J* = 7.8, 1.6 Hz, 1H), 7.49 – 7.40 (m, 4H), 7.35 – 7.21 (m, 5H), 7.19 (td, *J* = 7.5, 1.3 Hz, 1H), 7.07 (d, *J* = 16.2 Hz, 1H), 6.70 (ddd, *J* = 7.3, 5.0, 0.9 Hz, 1H), 6.66 (d, *J* = 8.4 Hz, 1H), 6.61 (s, 1H).

^{13}C NMR (101 MHz, $CDCl_3$) δ 157.1, 148.7, 137.90, 137.88, 137.4, 132.2, 131.1, 128.8, 128.6, 128.0, 126.9, 126.8, 125.0, 124.2, 124.1, 115.0, 108.0.

The characterization data was in agreement with the previous literature.[8]

(E)-*N*-Phenyl-2-styrylaniline (*trans*-1y)

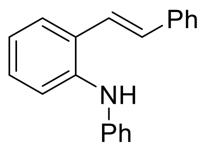

The product was prepared from 2-styrylaniline (490 mg, 2.00 mmol) and phenylboronic acid (620 mg, 4.1 mmol) according to the general procedure B. The reaction time was 18 h. The product was isolated with flash chromatography (silica gel, 10:1 n-Hex/EtOAc) as a brown solid (412 mg, 1.52 mmol, 76%).

^{1}H NMR (400 MHz, $CDCl_3$) δ 7.59 (dd, *J* = 7.8, 1.5 Hz, 1H), 7.48 (d, *J* = 7.0 Hz, 2H), 7.34 (t, *J* = 7.4 Hz, 2H), 7.32 – 7.19 (m, 6H), 7.08 – 7.01 (m, 2H), 7.01 – 6.96 (m, 2H), 6.91 (tt, *J* = 7.2, 1.1 Hz, 1H), 5.57 (s, 1H).

^{13}C NMR (101 MHz, $CDCl_3$) δ 144.1 (C), 140.7 (C), 137.6 (C), 131.1 (CH), 129.8 (C), 129.5 (2×CH), 128.9 (2×CH), 128.6 (CH), 127.9 (CH), 127.3 (CH), 126.7 (2×CH), 124.5 (CH), 122.7 (CH), 120.7 (CH), 120.1 (CH), 117.5 (2×CH).

HRMS (EI) *m/z*: [M^+] calcd for $C_{20}H_{17}N$ 271.1361, found: 271.1360.

***N*-Methyl-2-(1-phenylvinyl)aniline (1z)**

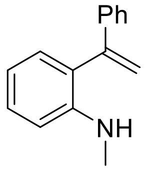

The product was prepared according to a literature procedure.[9] 2-(1-Phenylvinyl)aniline (244 mg, 1.25 mmol) was dissolved in THF (2.5 mL) and cooled to –78 °C. n-BuLi (1.6 M solution in hexane, 0.88 mL, 1.38 mmol) was added and the resulting mixture was allowed to warm to –40 °C. Then, the mixture was cooled to –78 °C, and iodomethane (0.12 mL, 1.9 mmol) was added dropwise. The reaction mixture was slowly warmed to room temperature and stirred for 18 h. The reaction was quenched by addition of water and the organics were extracted with EtOAc, washed with brine, dried over anhydrous $MgSO_4$, and concentrated by a rotary evaporator. The product was isolated with flash chromatography (silica gel, 100:1 n-Hex/EtOAc) as a pale-yellow oil (172 mg, 0.82 mmol, 66%).

^{1}H NMR (400 MHz, $CDCl_3$) δ 7.37 – 7.35 (m, 2H), 7.32 – 7.25 (m, 4H), 7.08 (d, *J* = 7.4 Hz, 1H), 6.74 (d, *J* = 7.4 Hz, 1H), 6.65 (d, *J* = 8.1 Hz, 1H), 5.82 (s, 1H), 5.33 (s, 1H), 3.71 (s, 1H), 2.72 (s, 3H).

^{13}C NMR (101 MHz, $CDCl_3$) δ 147.1, 146.8, 139.7, 130.1, 129.1, 128.7, 128.2, 127.3, 126.6, 116.7, 116.4, 110.0, 30.9.

The characterization data was in agreement with the previous literature.[9]

***N*-Methyl-2-(prop-1-en-2-yl)aniline (1aa)**

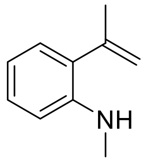

The product was prepared according to a literature procedure.[9] 2-(Prop-1-en-2-yl)aniline (347.2 mg, 3 mmol) was dissolved in THF (6 mL) and cooled to –78 °C. n-BuLi (1.6 M solution in hexane, 2.10 mL, 3.30 mmol) was added and the resulting mixture was allowed to warm to –40 °C. Then, the mixture was cooled to –78 °C, and iodomethane (0.28 mL, 4.5 mmol) was added dropwise. The reaction mixture was slowly warmed to room temperature and stirred for 18 h. The reaction was quenched by addition of water and the organics were extracted with EtOAc, washed with brine, dried over anhydrous $MgSO_4$, and

concentrated by a rotary evaporator. The product was isolated with flash chromatography (silica gel, 100:1 n-Hex/EtOAc) as a pale-yellow oil (182 mg, 1.43 mmol, 47%).

^{1}H NMR (400 MHz, $CDCl_3$) δ 7.17 (t, *J* = 7.8 Hz, 1H), 7.00 (dd, *J* = 7.4, 1.8 Hz, 1H), 6.68 (t, *J* = 7.4 Hz, 1H), 6.62 (d, *J* = 8.1 Hz, 1H), 5.28 (s, 1H), 5.02 (s, 1H), 4.15 (s, 1H), 2.83 (s, 3H), 2.04 (s, 3H).

^{13}C NMR (101 MHz, $CDCl_3$) δ 145.6, 143.7, 129.4, 128.3, 127.8, 116.5, 115.7, 109.9, 31.0, 24.2.

The characterization data was in agreement with the previous literature.[10]

Optimization of Reaction Conditions

General Screening Procedure

Amine **1a**, photocatalyst (varying amount), Co(dmgH)$_2$(py)Cl (varying amount), and solvent(s) were added in a vial which was sealed with a septum. The reaction mixture was bubbled through a needle with argon for 30 min and irradiated with blue LEDs (450 nm) while stirring at 30 °C for the specified time. A stock solution of 1,3,5-trimethoxybenzene (TMB) in DMSO was prepared in a volumetric flask and used as an internal standard for the reaction condition screening.

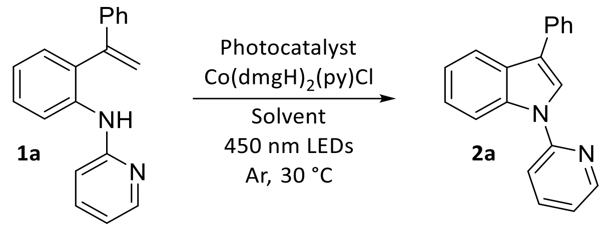

Table S1. Optimization of solvent. Conditions: Amine **1a** (27.2 mg, 0.1 mmol), PQ-CF$_3$ (3.4 mg, 10 mol%), Co(dmgH)$_2$(py)Cl (2.4 mg, 6 mol%), argon atmosphere, blue LEDs (450 nm), 30 °C, concentration 0.04 M in respective solvent. Reaction time 3 h.

Entry	Variation: Solvent	Unreacted **1a** (%)[a]	Yield of **2a** (%)[a]
**S1**	**MeCN**	**0**	**85**
S2	EtOAc	51	31
S3	DCE	57	19
S4	DMF	85	5
S5	DMSO	79	14
S6	acetone	75	16
S7	MeCN/H_2O (5:1)	70	24

[a]NMR yield; TMB as internal standard.

Table S2. Optimization of concentration. Conditions: Amine **1a** (27.2 mg, 0.1 mmol), PQ-CF$_3$ (3.4 mg, 10 mol%), Co(dmgH)$_2$(py)Cl (2.4 mg, 6 mol%), argon atmosphere, blue LEDs (450 nm), 30 °C, varying concentration in MeCN. Reaction time 1 h.

Entry	Variation: Concentration	Unreacted **1a** (%)[a]	Yield of **2a** (%)[a]
S1	0.02 M	77	16
**S2**	**0.04 M**	**43**	**51**
S3	0.06 M	52	35
S4	0.08 M	79	17

[a]NMR yield; TMB as internal standard.

Table S3. Optimization of photocatalyst. Conditions: Amine **1a** (27.2 mg, 0.1 mmol), varying photocatalyst (10 mol%), Co(dmgH)$_2$(py)Cl (2.4 mg, 6 mol%), argon atmosphere, blue LEDs (450 nm), 30 °C, concentration 0.04 M in MeCN. Reaction time 1 h.

Entry	Variation: Photocatalyst	Unreacted **1a** (%)[a]	Yield of **2a** (%)[a]
**S1**	**PQ-CF$_3$**	**43**	**51**
S2	PQ	50	37
S3	Eosin Y	95	4
S4	Anthraquinone	80	12

[a]NMR yield; TMB as internal standard.

Table S4. Optimization of catalyst loadings. Amine **1a** (27.2 mg, 0.1 mmol), PQ-CF_3 (varying amount), Co(dmgH)$_2$(py)Cl (varying amount), argon atmosphere, blue LEDs (450 nm), 30 °C, concentration 0.04 M in MeCN. Reaction time 1 h.

Entry	Variation: Catalyst loadings	Unreacted **1a** (%)[a]	Yield of **2a** (%)[a]
S1	PQ-CF_3 (5 mol%), [Co] (6 mol%)	65	22
**S2**	**PQ-CF_3 (10 mol%), [Co] (6 mol%)**	**43**	**51**
S3	PQ-CF_3 (15 mol%), [Co] (6 mol%)	56	30
S4	PQ-CF_3 (10 mol%), [Co] (3 mol%)	83	7
S5	PQ-CF_3 (10 mol%), [Co] (10 mol%)	69	26
S6	PQ-CF_3 (5 mol%), [Co] (3 mol%)	67	30

[a]NMR yield; TMB as internal standard.

Table S5. Optimization of other conditions. Standard conditions: Amine **1a** (27.2 mg, 0.1 mmol), PQ-CF_3 (3.4 mg, 10 mol%), Co(dmgH)$_2$(py)Cl (2.4 mg, 6 mol%), argon atmosphere, blue LEDs (450 nm), 30 °C, concentration 0.04 M in MeCN. Reaction time 1 h.

Entry	Variation: Miscellaneous	Unreacted **1a** (%)[a]	Yield of **2a** (%)[a]
S1	no light	100	0
S2	no photocatalyst	100	0
S3	no Co(dmgH)$_2$(py)Cl	89	7
S4	O_2 atmosphere	0	77
S5	O_2 atmosphere, no Co(dmgH)$_2$(py)Cl	41	55
S6	purple LEDs (420 nm)	69	20
S7	at r.t.	60	32

[a]NMR yield; TMB as internal standard.

Synthesis and Characterization of Indoles 2a–2v and 3a

General Procedure C

Amine **1a**–**1v** (0.2 mmol), PQ-CF_3 (10 mol%), Co(dmgH)$_2$(py)Cl (6 mol%), and MeCN (5 mL) were added in a vial which was sealed with a septum. Loose-fitting septa were secured with Parafilm. The mixture was bubbled through a needle with argon for 30 min and irradiated with blue LEDs (450 nm) while stirring at 30 °C for 48 h. The solvent was evaporated on a rotary evaporator and the crude product was purified with flash chromatography on silica gel using mixtures of n-Hex and EtOAc.

3-Phenyl-1-(pyridin-2-yl)-1*H*-indole (2a)

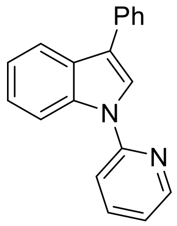

The product was prepared from **1a** according to the general procedure C and isolated with flash chromatography (silica gel, n-Hex:EtOAc 40:1 → 20:1) as a white solid (38.2 mg, 0.14 mmol, 70%).

^{1}H NMR (400 MHz, $CDCl_3$) δ 8.58 (dd, *J* = 4.9, 1.1 Hz, 1H), 8.25 (d, *J* = 8.4 Hz, 1H), 7.95 (d, *J* = 8.0 Hz, 1H), 7.87 (s, 1H), 7.87 – 7.78 (m, 1H), 7.72 (dd, *J* = 8.2, 1.3 Hz, 2H), 7.54 (d, *J* = 8.2 Hz, 1H), 7.47 (t, *J* = 7.7 Hz, 2H), 7.38 – 7.30 (m, 2H), 7.32 – 7.23 (m, 1H), 7.17 (dd, *J* = 7.4, 4.9 Hz, 1H).

^{13}C NMR (101 MHz, $CDCl_3$) δ 152.4, 149.2, 138.6, 136.0, 134.9, 128.9, 128.6, 128.0, 126.7, 123.7, 123.6, 121.9, 120.9, 120.4, 120.2, 114.9, 113.3.

The characterization data was in agreement with the previous literature.[11]

1-(5-Methoxypyridin-2-yl)-3-phenyl-1*H*-indole (2b)

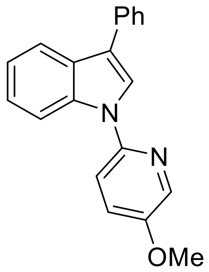

The product was prepared from **1b** according to the general procedure C and isolated with flash chromatography (silica gel, n-Hex:EtOAc 40:1 → 20:1) as a white solid (49.5 mg, 0.16 mmol, 83%).

^{1}H NMR (400 MHz, $CDCl_3$) δ 8.25 (d, *J* = 3.1 Hz, 1H), 8.04 (d, *J* = 8.3 Hz, 1H), 7.95 (d, *J* = 7.6 Hz, 1H), 7.76 (s, 1H), 7.71 (dd, *J* = 8.2, 1.3 Hz, 2H), 7.49 – 7.39 (m, 3H), 7.35 – 7.20 (m, 4H), 3.87 (s, 3H).

^{13}C NMR (101 MHz, $CDCl_3$) δ 153.6 (C), 145.8 (C), 136.0 (C), 135.6 (CH), 135.1 (C), 128.9 (2×CH), 128.0 (C), 127.8 (2×CH), 126.5 (CH), 124.0 (CH), 123.9 (CH), 123.4 (CH), 121.4 (CH), 120.2 (CH), 120.0 (C), 115.9 (CH), 112.5 (CH), 56.1 (CH_3).

HRMS (EI) *m/z*: [M^+] calcd for $C_{20}H_{16}N_2O$ 300.1263, found: 300.1256.

1,3-Diphenyl-1*H*-indole (2c)

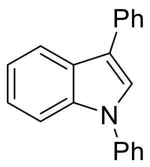

The product was prepared from **1c** according to the general procedure C and isolated with flash chromatography (silica gel, n-Hex:EtOAc[2% AcOH] 200:1 → 80:1) as a white solid (40.3 mg, 0.15 mmol, 74%). The purified product contained a trace of unreacted **1c**.

^{1}H NMR (400 MHz, $CDCl_3$) δ 8.02 – 7.95 (m, 1H), 7.71 (dd, *J* = 8.2, 1.3 Hz, 2H), 7.59 (dd, *J* = 6.6, 1.6 Hz, 1H), 7.55 – 7.48 (m, 5H), 7.46 (t, *J* = 7.8 Hz, 2H), 7.35 (tt, *J* = 6.1, 2.2 Hz, 1H), 7.33 – 7.19 (m, 3H).

^{13}C NMR (126 MHz, $CDCl_3$) δ 139.7, 136.8, 135.3, 129.8, 129.0, 127.8, 127.3, 126.8, 126.4, 125.7, 124.7, 123.0, 121.0, 120.3, 119.3, 111.0.

The characterization data was in agreement with the previous literature.[12]

3-Methyl-1-(pyridin-2-yl)-1*H*-indole (2d)

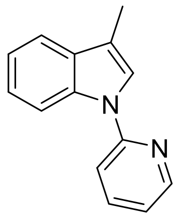

The product was prepared from **1d** according to the general procedure C and isolated with flash chromatography (silica gel, n-Hex:EtOAc 40:1) as a colorless oil (2.0 mg, 0.010 mmol, 5%).

^{1}H NMR (400 MHz, $CDCl_3$) δ 8.54 (dd, *J* = 5.5, 1.6 Hz, 1H), 8.22 (d, *J* = 8.3 Hz, 1H), 7.79 (ddd, *J* = 8.3, 7.3, 2.0 Hz, 1H), 7.60 (d, *J* = 7.9 Hz, 1H), 7.52 (s, 1H), 7.46 (d, *J* = 8.3 Hz, 1H), 7.30 (t, *J* = 7.7 Hz, 1H), 7.22 (t, *J* = 7.4 Hz, 1H), 7.11 (dd, *J* = 7.4, 4.9 Hz, 1H), 2.38 (d, *J* = 1.2 Hz, 3H).

^{13}C NMR (101 MHz, $CDCl_3$) δ 152.8, 149.0, 138.4, 135.5, 131.2, 123.4, 123.3, 120.9, 119.6, 119.2, 114.9, 114.2, 113.2, 9.8.

The characterization data was in agreement with the previous literature.[13]

3-Methyl-1-phenyl-1*H*-indole (2e)

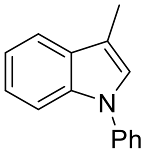

The product was prepared from **1e** (35.2 mg, 0.17 mmol) according to the general procedure C and isolated with flash chromatography (silica gel, n-Hex:EtOAc 100:0 → 80:1) as a colorless oil (3.6 mg, 0.017 mmol, 10%).

^{1}H NMR (400 MHz, $CDCl_3$) δ 7.66 – 7.59 (m, 1H), 7.56 (d, *J* = 8.0 Hz, 1H), 7.5 – 7.47 (m, 4H), 7.36 – 7.27 (m, 1H), 7.22 (td, *J* = 7.6, 1.4 Hz, 1H), 7.17 (td, *J* = 7.4, 1.2 Hz, 1H), 7.14 (d, *J* = 1.1 Hz, 1H), 2.39 (d, *J* = 1.0 Hz, 3H).

^{13}C NMR (101 MHz, $CDCl_3$) δ 140.1, 136.1, 129.9, 129.7, 126.1, 125.6, 124.1, 122.5, 119.9, 119.3, 113.0, 110.5, 9.7.

The characterization data was in agreement with the previous literature.[14]

3-Cyclopropyl-1-(pyridin-2-yl)-1*H*-indole (2f)

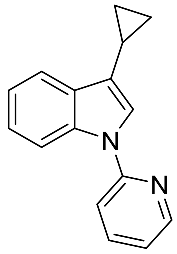

The product was prepared from **1f** according to the general procedure C and isolated with flash chromatography (silica gel, n-Hex:EtOAc 40:1) as a colorless oil (13.0 mg, 0.055 mmol, 28%).

^{1}H NMR (400 MHz, $CDCl_3$) δ 8.53 (dd, *J* = 5.0, 1.9 Hz, 1H), 8.17 (d, *J* = 8.2 Hz, 1H), 7.81 – 7.73 (m, 2H), 7.44 (d, *J* = 8.3 Hz, 1H), 7.42 (s, 1H), 7.29 (t, *J* = 7.7 Hz, 1H), 7.22 (t, *J* = 6.8 Hz, 1H), 7.10 (dd, *J* = 8.3, 4.9 Hz, 1H), 2.04 – 1.93 (m, 1H), 0.98 – 0.89 (m, 2H), 0.74 – 0.67 (m, 2H).

^{13}C NMR (101 MHz, $CDCl_3$) δ 152.6 (C), 149.1 (CH), 138.4 (CH), 135.5 (C), 131.1 (C), 123.5 (CH), 122.3 (C), 122.0 (CH), 121.0 (CH), 120.0 (CH), 119.5 (CH), 114.3 (CH), 113.1 (CH), 6.3 (2xCH_2), 6.2 (CH).

HRMS (EI) *m/z*: [M^+] calcd for $C_{16}H_{14}N_2$ 234.1157, found: 234.1155.

5-Methoxy-3-phenyl-1-(pyridin-2-yl)-1*H*-indole (2g)

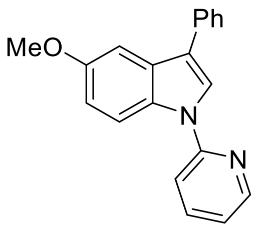

The product was prepared from **1g** according to the general procedure C and isolated with flash chromatography (silica gel, n-Hex:EtOAc 40:1 → 20:1) as a white solid (20.0 mg, 0.067 mmol, 33%).

^1H NMR (400 MHz, $CDCl_3$) δ 8.56 (dd, *J* = 4.9, 2.6 Hz, 1H), 8.22 (d, *J* = 9.1 Hz, 1H), 7.87 – 7.78 (m, 2H), 7.70 (d, *J* = 6.8 Hz, 2H), 7.53 – 7.44 (m, 3H), 7.38 (d, *J* = 2.6 Hz, 1H), 7.34 (t, *J* = 7.4 Hz, 1H), 7.16 (dd, *J* = 7.4, 4.9 Hz, 1H), 6.99 (dd, *J* = 9.1, 2.6 Hz, 1H), 3.88 (s, 3H).

^{13}C NMR (101 MHz, $CDCl_3$) δ 155.6 (C), 152.5 (C), 149.1 (CH), 138.6 (CH), 135.0 (C), 131.1 (C), 129.3 (C), 129.0 (2×CH), 127.9 (2×CH), 126.7 (CH), 124.0 (CH), 120.7 (C), 120.1 (CH), 114.6 (CH), 114.2 (CH), 113.2 (CH), 102.2 (CH), 56.0 (CH_3).

HRMS (EI) *m/z*: [M^+] calcd for $C_{20}H_{16}N_2O$ 300.1263, found: 300.1258.

5-Methyl-3-phenyl-1-(pyridin-2-yl)-1*H*-indole (2h)

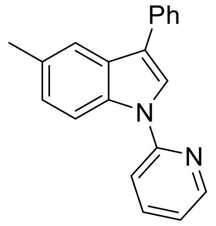

The product was prepared from **1h** according to the general procedure C and isolated with flash chromatography (silica gel, n-Hex:EtOAc 40:1 → 20:1) as a white solid (36.8 mg, 0.13 mmol, 65%).

^1H NMR (400 MHz, $CDCl_3$) δ 8.56 (dd, *J* = 4.9, 1.1 Hz, 1H), 8.13 (d, *J* = 8.5 Hz, 1H), 7.84 (s, 1H), 7.78 (ddd, *J* = 8.4, 7.3, 2.0 Hz, 1H), 7.74 – 7.68 (m, 3H), 7.50 (d, *J* = 8.2 Hz, 1H), 7.47 (t, *J* = 7.7 Hz, 2H), 7.32 (t, *J* = 7.4 Hz, 1H), 7.18 – 7.10 (m, 2H), 2.49 (s, 3H).

^{13}C NMR (101 MHz, $CDCl_3$) δ 152.5 (C), 149.1 (CH), 138.5 (CH), 135.0 (C), 134.3 (C), 131.2 (C), 128.9 (2×CH), 128.0 (2×CH), 126.6 (CH), 125.1 (CH), 123.6 (CH), 120.6 (C), 120.1 (CH), 119.9 (CH), 114.5 (CH), 113.1 (CH), 21.7 (CH_3).

HRMS (EI) *m/z*: [M^+] calcd for $C_{20}H_{16}N_2$ 284.1313, found: 284.1315.

5-Chloro-3-phenyl-1-(pyridin-2-yl)-1*H*-indole (2i)

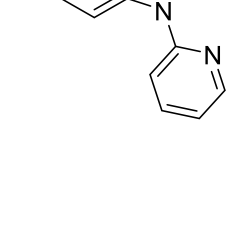

The product was prepared from **1i** according to the general procedure C and isolated with flash chromatography (silica gel, n-Hex:EtOAc 40:1 → 20:1) as a pale yellow solid (45.7 mg, 0.15 mmol, 74%).

^1H NMR (400 MHz, $CDCl_3$) δ 8.54 (ddd, *J* = 4.9, 2.0, 0.9 Hz, 1H), 8.21 (d, *J* = 8.9 Hz, 1H), 7.86 (d, *J* = 2.1 Hz, 1H), 7.80 – 7.73 (m, 2H), 7.66 – 7.58 (m, 2H), 7.46 (t, *J* = 7.7 Hz, 2H), 7.42 (d, *J* = 8.2 Hz, 1H), 7.37 – 7.30 (m, 1H), 7.26 (dd, *J* = 8.9, 2.1 Hz, 1H), 7.15 (ddd, *J* = 7.4, 4.9, 0.9 Hz, 1H).

^{13}C NMR (101 MHz, $CDCl_3$) δ 152.1 (C), 149.1 (CH), 138.7 (CH), 134.3 (C), 134.1 (C), 129.7 (C), 129.0 (2×CH), 127.8 (2×CH), 127.5 (C), 127.0 (CH), 124.4 (CH), 123.8 (CH), 120.6 (CH), 120.5 (C), 119.6 (CH), 114.9 (CH), 114.5 (CH).

HRMS (EI) *m/z*: [M$^+$] calcd for $C_{19}H_{13}ClN_2$ 304.0767, found: 304.0761.

5-Bromo-3-phenyl-1-(pyridin-2-yl)-1*H*-indole (2j)

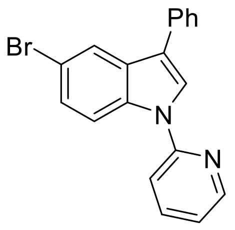

The product was prepared from **1j** according to the general procedure C and isolated with flash chromatography (silica gel, n-Hex:EtOAc 40:1 → 20:1) as a white solid (50.8 mg, 0.15 mmol, 73%).

^{1}H NMR (400 MHz, $CDCl_3$) δ 8.58 (ddd, *J* = 4.9, 2.0, 0.9 Hz, 1H), 8.19 (d, *J* = 8.9 Hz, 1H), 8.04 (d, *J* = 2.0 Hz, 1H), 7.89 – 7.80 (m, 2H), 7.66 (dd, *J* = 8.2, 1.3 Hz, 2H), 7.52 – 7.45 (m, 3H), 7.42 (dd, *J* = 8.9, 2.0 Hz, 1H), 7.38 – 7.32 (m, 1H), 7.20 (ddd, *J* = 7.4, 4.9, 0.9 Hz, 1H).

^{13}C NMR (126 MHz, $CDCl_3$) δ 152.2 (C), 149.2 (CH), 138.8 (CH), 134.7 (C), 134.1 (C), 130.3 (C), 129.1 (2×CH), 128.0 (2×CH), 127.1 (CH), 126.5 (CH), 124.3 (CH), 122.8 (CH), 120.7 (CH), 120.5 (C), 115.23 (CH), 115.21 (C), 114.6 (CH).

HRMS (EI) *m/z*: [M$^+$] calcd for $C_{19}H_{13}BrN_2$ 348.0262, found: 348.0268.

5-Fluoro-3-phenyl-1-(pyridin-2-yl)-1*H*-indole (2k)

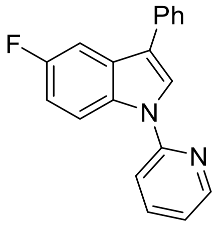

The product was prepared from **1k** according to the general procedure C and isolated with flash chromatography (silica gel, n-Hex:EtOAc 40:1 → 20:1) as a white solid (47.2 mg, 0.16 mmol, 82%).

^{1}H NMR (400 MHz, $CDCl_3$) δ 8.58 (ddd, *J* = 4.9, 1.9, 0.9 Hz, 1H), 8.28 (dd, *J* = 9.1, 4.6 Hz, 1H), 7.86 (s, 1H), 7.83 (ddd, *J* = 8.3, 7.4, 2.0 Hz, 1H), 7.67 (dd, *J* = 8.2, 1.3 Hz, 2H), 7.57 (dd, *J* = 9.6, 2.6 Hz, 1H), 7.52 – 7.43 (m, 3H), 7.34 (t, *J* = 7.4 Hz, 1H), 7.19 (ddd, *J* = 7.4, 4.9, 1.0 Hz, 1H), 7.08 (td, *J* = 9.0, 2.6 Hz, 1H).

^{13}C NMR (126 MHz, $CDCl_3$) δ 159.1 (d, *J* = 237.2 Hz, CF), 152.3 (C), 149.1 (CH), 138.7 (CH), 134.4 (C), 132.6 (C), 129.2 (d, *J* = 9.7 Hz, C), 129.1 (2×CH), 127.8 (2×CH), 126.9 (CH), 124.7 (CH), 120.8 (d, *J* = 4.4 Hz, C), 120.5 (CH), 114.8 (d, *J* = 9.1 Hz, CH), 114.4 (CH), 111.8 (d, *J* = 25.5 Hz, CH), 105.3 (d, *J* = 24.1 Hz, CH).

^{19}F NMR (376 MHz, $CDCl_3$) δ -121.9.

HRMS (EI) *m/z*: [M$^+$] calcd for $C_{19}H_{13}FN_2$ 288.1063, found: 288.1064.

5,6-Dimethoxy-3-phenyl-1-(pyridin-2-yl)-1*H*-indole (2l)

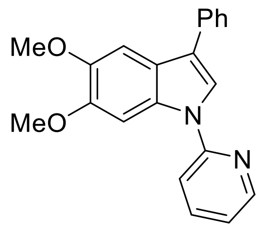

The product was prepared from **1l** according to the general procedure C and isolated with flash chromatography (silica gel, n-Hex:EtOAc 5:1 → 1:1) as a yellow solid (29.2 mg, 0.088 mmol, 44%).

^{1}H NMR (400 MHz, $CDCl_3$) δ 8.57 (dd, *J* = 5.0, 1.3 Hz, 1H), 8.03 (s, 1H), 7.85 – 7.76 (m, 1H), 7.72 – 7.65 (m, 2H), 7.66 (s, 1H), 7.52 – 7.42 (m, 3H), 7.36 – 7.30 (m, 2H), 7.15 (dd, *J* = 7.4, 4.9 Hz, 1H), 3.99 (s, 3H), 3.95 (s, 3H).

^{13}C NMR (101 MHz, $CDCl_3$) δ 152.9 (C), 148.9 (CH), 147.7 (C), 146.4 (C), 138.6 (CH), 135.1 (C), 130.5 (C), 129.0 (2×CH), 127.8 (2×CH), 126.6 (CH), 121.51 (CH), 121.46 (C), 121.0 (C), 119.9 (CH), 114.0 (CH), 101.5 (CH), 97.9 (CH), 56.43 (CH_3), 56.42 (CH_3).

HRMS (EI) *m/z*: [M^+] calcd for $C_{21}H_{18}N_2O_2$ 330.1368, found: 330.1363.

6-Methoxy-3-phenyl-1-(pyridin-2-yl)-1*H*-indole (2m-1) and 4-Methoxy-3-phenyl-1-(pyridin-2-yl)-1*H*-indole (2m-2)

The products were prepared from the mixture containing 80:20 **1m-1** and **1m-2** according to the general procedure C. Both isomers were isolated with flash chromatography (silica gel, n-Hex:EtOAc 40:1 → 10:1) as colorless oils, **2m-1** in 60% yield (36.2 mg, 0.12 mmol) and **2m-2** in 14% yield (8.3 mg, 0.028 mmol). Considering the amounts of **1m-1** and **1m-2** in the initial mixture, the yields of **2m-1** and **2m-2** were 75% and 70%, respectively.

6-Methoxy-3-phenyl-1-(pyridin-2-yl)-1*H*-indole (2m-1)

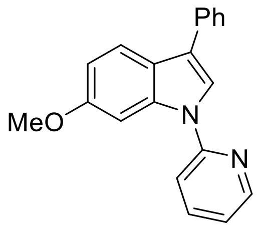

^{1}H NMR (400 MHz, $CDCl_3$) δ 8.56 (dd, *J* = 4.9, 1.1 Hz, 1H), 7.82 (d, *J* = 8.5 Hz, 1H), 7.83 – 7.74 (m, 1H), 7.70 – 7.63 (m, 2H), 7.61 (s, 1H), 7.50 (d, *J* = 8.1 Hz, 1H), 7.39 (t, *J* = 7.4 Hz, 2H), 7.30 (t, *J* = 7.4 Hz, 1H), 7.23 (d, *J* = 8.2 Hz, 1H), 7.15 (ddd, *J* = 7.4, 4.9, 1.0 Hz, 1H), 6.66 (d, *J* = 7.9 Hz, 1H), 3.81 (s, 3H).

^{13}C NMR (101 MHz, $CDCl_3$) δ 154.7 (C), 152.4 (C), 149.2 (CH), 138.5 (CH), 137.4 (C), 135.6 (C), 129.9 (2×CH), 127.6 (2×CH), 126.3 (CH), 124.3 (CH), 124.0 (CH), 121.3 (C), 120.5 (CH), 118.2 (C), 115.3 (CH), 106.2 (CH), 102.4 (CH), 55.3 (CH_3).

HRMS (EI) *m/z*: [M^+] calcd for $C_{20}H_{16}N_2O$ 300.1263, found: 300.1268.

4-Methoxy-3-phenyl-1-(pyridin-2-yl)-1*H*-indole (2m-2)

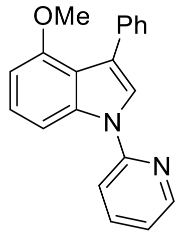

^{1}H NMR (400 MHz, $CDCl_3$) δ 8.59 (dd, *J* = 4.9, 1.1 Hz, 1H), 7.90 (d, *J* = 2.3 Hz, 1H), 7.88 – 7.77 (m, 2H), 7.73 (s, 1H), 7.70 (d, *J* = 7.0 Hz, 2H), 7.51 (d, *J* = 8.3 Hz, 1H), 7.46 (t, *J* = 7.8 Hz, 2H), 7.32 (t, *J* = 7.4 Hz, 1H), 7.18 (ddd, *J* = 7.4, 4.9, 0.9 Hz, 1H), 6.94 (dd, *J* = 8.7, 2.3 Hz, 1H), 3.91 (s, 3H).

^{13}C NMR (101 MHz, $CDCl_3$) δ 157.5 (C), 152.7 (C), 149.1 (CH), 138.6 (CH), 136.9 (C), 134.9 (C), 128.9 (2×CH), 127.8 (2×CH), 126.7 (CH), 122.8 (C), 122.2 (CH), 121.0 (C), 120.7 (CH), 120.2 (CH), 114.6 (CH), 111.1 (CH), 97.9 (CH), 56.0 (CH_3).

HRMS (EI) *m/z*: [M^+] calcd for $C_{20}H_{16}N_2O$ 300.1263, found: 300.1272.

6-Fluoro-3-phenyl-1-(pyridin-2-yl)-1*H*-indole (2n)

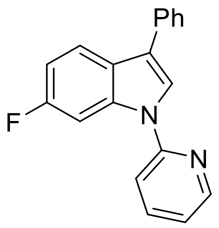

The product was prepared from **1n** (43.9 mg, 0.15 mmol) according to the general procedure C and isolated with flash chromatography (silica gel, n-Hex:EtOAc 40:1 → 10:1) as a colorless oil (16.2 mg, 0.056 mmol, 37%).

^{1}H NMR (500 MHz, $CDCl_3$) δ 8.62 – 8.57 (m, 1H), 8.03 (d, *J* = 8.4 Hz, 1H), 7.85 (td, *J* = 7.8, 1.9 Hz, 1H), 7.72 (s, 1H), 7.71 – 7.65 (m, 2H), 7.53 (d, *J* = 8.2 Hz, 1H), 7.44 (t, *J* = 7.7 Hz, 2H), 7.34 (t, *J* = 7.4 Hz, 1H), 7.29 – 7.19 (m, 2H), 6.92 (dd, *J* = 11.2, 7.9 Hz, 1H).

^{13}C NMR (101 MHz, $CDCl_3$) δ 156.9 (d, *J* = 248.7 Hz, CF), 152.2 (C), 149.3 (CH), 138.7 (CH), 138.3 (d, *J* = 10.3 Hz, C), 134.4 (C), 129.1 (d, *J* = 4.0 Hz, 2×CH), 128.3 (2×CH), 126.9 (CH), 124.6 (CH), 124.2 (d, *J* = 8.1 Hz, CH), 120.9 (CH), 119.8 (d, *J* = 3.0 Hz, C), 117.0 (d, *J* = 18.9 Hz, C), 115.2 (CH), 109.3 (d, *J* = 3.8 Hz, CH), 107.4 (d, *J* = 20.3 Hz, CH).

^{19}F NMR (376 MHz, $CDCl_3$) δ -116.1.

HRMS (EI) *m/z*: [M^+] calcd for $C_{19}H_{13}FN_2$ 288.1063, found: 288.1059.

4-Fluoro-3-phenyl-1-(pyridin-2-yl)-1*H*-indole (2o)

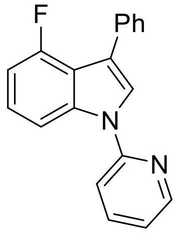

The product was prepared from **1o** according to the general procedure C and isolated with flash chromatography (silica gel, n-Hex:EtOAc 40:1 → 20:1) as a colorless oil with the purity of 94% (24.1 mg, 0.083 mmol, 43%).

^{1}H NMR (400 MHz, $CDCl_3$) δ 8.57 (ddd, *J* = 4.9, 2.0, 0.9 Hz, 1H), 8.10 (dd, *J* = 10.6, 2.4 Hz, 1H), 7.86 – 7.77 (m, 2H), 7.78 (s, 1H), 7.67 (dd, *J* = 8.2, 1.3 Hz, 2H), 7.46 (t, *J* = 7.8 Hz, 3H), 7.34 (t, *J* = 7.4 Hz, 1H), 7.17 (ddd, *J* = 7.4, 4.9, 0.9 Hz, 1H), 7.02 (td, *J* = 8.9, 2.4 Hz, 1H).

^{13}C NMR (101 MHz, $CDCl_3$) δ 160.8 (d, *J* = 239.0 Hz, CF), 152.3 (C), 149.1 (CH), 138.7 (CH), 136.1 (d, *J* = 12.7 Hz, C), 134.5 (C), 129.0 (2×CH), 127.9 (2×CH), 126.9 (CH), 125.0 (C), 123.3 (d, *J* = 3.7 Hz, CH), 121.1 (C), 120.8 (d, *J* = 10.0 Hz, CH), 120.4 (CH), 114.3 (CH), 110.3 (d, *J* = 24.4 Hz, CH), 100.9 (d, *J* = 28.3 Hz, CH).

^{19}F NMR (376 MHz, $CDCl_3$) δ -118.6.

HRMS (EI) *m/z*: [M^+] calcd for $C_{19}H_{13}FN_2$ 288.1063, found: 288.1067.

1-(Pyridin-2-yl)-3-(*p*-tolyl)-1*H*-indole (2p)

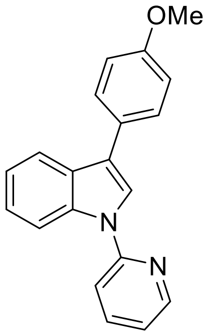

The product was prepared from **1p** according to the general procedure C and isolated with flash chromatography (silica gel, n-Hex:EtOAc 20:1 → 5:1) as a colorless oil (40.4 mg, 0.13 mmol, 67%).

^{1}H NMR (400 MHz, $CDCl_3$) δ 8.56 (dd, *J* = 5.1, 1.8 Hz, 1H), 8.25 (d, *J* = 8.3 Hz, 1H), 7.89 (d, *J* = 7.9 Hz, 1H), 7.82 – 7.73 (m, 2H), 7.63 (d, *J* = 8.7 Hz, 2H), 7.50 (d, *J* = 8.2 Hz, 1H), 7.33 (t, *J* = 7.7 Hz, 1H), 7.29 – 7.23 (m, 1H), 7.13 (dd, *J* = 7.4, 4.9 Hz, 1H), 7.01 (d, *J* = 8.7 Hz, 2H), 3.85 (s, 3H).

^{13}C NMR (101 MHz, $CDCl_3$) δ 158.6 (C), 152.5 (C), 149.1 (CH), 138.5 (CH), 135.9 (C), 129.0 (2×CH), 128.8 (C), 127.3 (C), 123.6 (CH), 122.9 (CH), 121.7 (CH), 120.6 (C), 120.2 (2×CH), 114.7 (CH), 114.4 (2×CH), 113.3 (CH), 55.5 (CH_3).

HRMS (EI) *m/z*: [M^+] calcd $C_{20}H_{16}N_2O$ 300.1263, found: 300.1266.

***N,N*-Dimethyl-4-(1-(pyridin-2-yl)-1*H*-indol-3-yl)aniline (2q)**

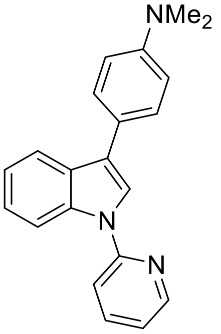

The product was prepared from **1q** according to the general procedure C and isolated with flash chromatography (silica gel, n-Hex:EtOAc 20:1 → 3:1) as a colorless oil (9.8 mg, 0.031 mmol, 16%). The starting material **1q** contained 4 wt% of **2q** as an impurity, which was taken into account when calculating the yield.

^{1}H NMR (400 MHz, $CDCl_3$) δ 8.58 (dd, *J* = 4.9, 1.1 Hz, 1H), 8.27 (d, *J* = 8.5 Hz, 1H), 7.93 (d, *J* = 7.6 Hz, 1H), 7.82 (td, *J* = 8.3, 2.0 Hz, 1H), 7.78 (s, 1H), 7.61 (d, *J* = 8.8 Hz, 2H), 7.53 (d, *J* = 8.3 Hz, 1H), 7.37 – 7.28 (m, 1H), 7.28 – 7.22 (m, 1H), 7.15 (dd, *J* = 7.4, 4.9 Hz, 1H), 6.87 (d, *J* = 8.8 Hz, 2H), 3.01 (s, 6H).

^{13}C NMR (101 MHz, $CDCl_3$) δ 152.6 (C), 149.7 (C), 149.1 (CH), 138.5 (CH), 135.9 (C), 129.1 (C), 128.8 (2×CH), 123.5 (CH), 123.1 (C), 122.4 (CH), 121.5 (CH), 121.2 (C), 120.4 (CH), 120.0 (CH), 114.7 (CH), 113.3 (CH), 113.2 (2×CH), 40.9 (2×CH_3).

HRMS (EI) *m/z*: [M^+] calcd for $C_{21}H_{19}N_3$ 313.1579, found: 313.1580.

1-(Pyridin-2-yl)-3-(*p*-tolyl)-1*H*-indole (2r)

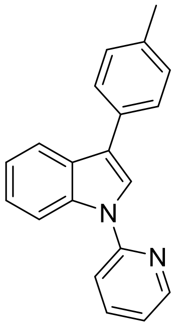

The product was prepared from **1r** according to the general procedure C and isolated with flash chromatography (silica gel, n-Hex:EtOAc 40:1 → 20:1) as a colorless oil (39.3 mg, 0.14 mmol, 69%).

^{1}H NMR (400 MHz, $CDCl_3$) δ 8.57 (dd, *J* = 5.0, 1.9 Hz, 1H), 8.25 (d, *J* = 8.3 Hz, 1H), 7.93 (d, *J* = 7.9 Hz, 1H), 7.84 (s, 1H), 7.80 (td, *J* = 7.9, 2.0 Hz, 1H), 7.61 (d, *J* = 7.8 Hz, 2H), 7.52 (d, *J* = 8.2 Hz, 1H), 7.33 (t, *J* = 7.8 Hz, 1H), 7.30 – 7.21 (m, 3H), 7.15 (dd, *J* = 7.4, 4.9 Hz, 1H), 2.41 (s, 3H).

^{13}C NMR (101 MHz, $CDCl_3$) δ 152.5 (C), 149.2 (CH), 138.5 (CH), 136.4 (C), 135.9 (C), 131.9 (C), 129.6 (2×CH), 128.7 (C), 127.8 (2×CH), 123.6 (CH), 123.3 (CH), 121.8 (CH), 120.9 (C), 120.3 (CH), 120.2 (CH), 114.8 (CH), 113.3 (CH), 21.4 (CH_3).

HRMS (EI) *m/z*: [M^+] calcd $C_{20}H_{16}N_2$ 284.1313, found: 284.1313.

3-(4-Fluorophenyl)-1-(pyridin-2-yl)-1*H*-indole (2s)

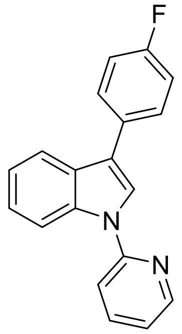

The product was prepared from **1s** according to the general procedure C and isolated with flash chromatography (silica gel, n-Hex:EtOAc 40:1 → 20:1) as a colorless oil (42.0 mg, 0.15 mmol, 73%).

^{1}H NMR (400 MHz, $CDCl_3$) δ 8.56 (dd, *J* = 5.0, 1.8 Hz, 1H), 8.22 (d, *J* = 8.3 Hz, 1H), 7.86 (d, *J* = 7.9 Hz, 1H), 7.82 – 7.74 (m, 2H), 7.64 (dd, *J* = 8.5, 5.5 Hz, 2H), 7.50 (d, *J* = 8.2 Hz, 1H), 7.33 (t, *J* = 7.8 Hz, 1H), 7.26 (t, *J* = 7.5 Hz, 1H), 7.19 – 7.10 (m, 3H).

^{13}C NMR (101 MHz, $CDCl_3$) δ 161.9 (d, *J* = 245.4 Hz, CF), 152.3 (C), 149.2 (CH), 138.6 (CH), 135.8 (C), 130.8 (d, *J* = 3.2 Hz, C), 129.4 (d, *J* = 7.8 Hz, 2×CH), 128.5 (C), 123.7 (CH), 123.5 (CH), 121.9 (CH), 120.4 (CH), 119.93 (CH), 119.88 (C), 115.8 (d, *J* = 21.3 Hz, 2×CH), 114.8 (CH), 113.3 (CH).

^{19}F NMR (376 MHz, $CDCl_3$) δ -116.0.

HRMS (EI) *m/z*: [M$^+$] calcd for $C_{19}H_{13}FN_2$ 288.1063, found: 288.1064.

1-(Pyridin-2-yl)- 3-(4-(trifluoromethyl)phenyl)-1*H*-indole (2t)

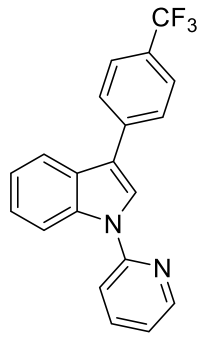

The product was prepared from **1t** according to the general procedure C and isolated with flash chromatography (silica gel, n-Hex:EtOAc 40:1 → 20:1) as a colorless oil (39.6 mg, 0.12 mmol, 58%).

^{1}H NMR (400 MHz, $CDCl_3$) δ 8.58 (ddd, *J* = 4.9, 2.0, 0.9 Hz, 1H), 8.20 (d, *J* = 8.3 Hz, 1H), 7.96 – 7.88 (m, 2H), 7.87 – 7.77 (m, 3H), 7.70 (d, *J* = 8.1 Hz, 2H), 7.54 (dt, *J* = 8.3, 0.9 Hz, 1H), 7.36 (ddd, *J* = 8.4, 7.1, 1.3 Hz, 1H), 7.29 (ddd, *J* = 8.1, 7.1, 1.1 Hz, 1H), 7.19 (ddd, *J* = 7.4, 4.9, 1.0 Hz, 1H).

^{13}C NMR (101 MHz, $CDCl_3$) δ 152.1 (C), 149.3 (CH), 138.68 (CH), 138.65 (C), 134.0 (C), 128.5 (q, *J* = 32.4 Hz, CCF_3), 128.1 (C), 127.8 (2×CH), 125.9 (q, *J* = 3.7 Hz, 2×CH), 124.6 (q, *J* = 271.8 Hz, CF_3), 124.5 (CH), 124.0 (CH), 122.2 (CH), 120.8 (CH), 119.9 (CH), 119.4 (C), 115.1 (CH), 113.3 (CH).

^{19}F NMR (376 MHz, $CDCl_3$) δ -62.2.

HRMS (EI) *m/z*: [M$^+$] calcd for $C_{20}H_{13}F_3N_2$ 338.1031, found: 338.1037.

1-(Pyridin-2-yl)-3-(*o*-tolyl)-1H-indole (2u)

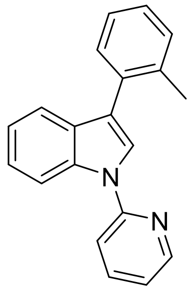

The product was prepared from **1u** according to the general procedure C and isolated with flash chromatography (silica gel, n-Hex:EtOAc 40:1 → 20:1) as a colorless oil (43.8 mg, 0.15 mmol, 76%).

^{1}H NMR (400 MHz, $CDCl_3$) δ 8.58 (ddd, *J* = 4.9, 2.0, 0.9 Hz, 1H), 8.28 (d, *J* = 8.3 Hz, 1H), 7.83 (ddd, *J* = 8.2, 7.4, 2.0 Hz, 1H), 7.70 (s, 1H), 7.51 (dd, *J* = 12.6, 8.1 Hz, 2H), 7.48 – 7.42 (m, 1H), 7.37 – 7.26 (m, 3H), 7.25 – 7.17 (m, 1H), 7.17 (ddd, *J* = 7.4, 5.1, 1.1 Hz, 1H), 2.36 (s, 3H).

^{13}C NMR (101 MHz, $CDCl_3$) δ 152.6 (C), 149.2 (CH), 138.6 (CH), 137.2 (C), 135.2 (C), 133.8 (C), 131.0 (CH), 130.5 (CH), 130.0 (C), 127.4 (CH), 125.8 (CH), 124.4 (CH), 123.5 (CH), 121.5 (CH), 120.6 (CH), 120.3 (C), 120.2 (CH), 114.7 (CH), 113.3 (CH), 20.9 (CH_3).

HRMS (EI) *m/z*: [M^+] calcd for $C_{20}H_{16}N_2$ 284.1313, found: 284.1311.

1-(Pyridin-2-yl)-3-(*m*-tolyl)-1*H*-indole (2v)

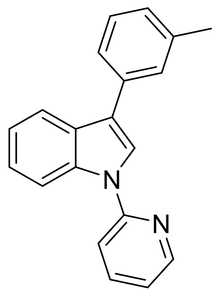

The product was prepared from **1v** according to the general procedure C and isolated with flash chromatography (silica gel, n-Hex:EtOAc 40:1 → 20:1) as a colorless oil (44.7 mg, 0.16 mmol, 78%).

^{1}H NMR (400 MHz, $CDCl_3$) δ 8.57 (ddd, *J* = 4.9, 2.0, 0.9 Hz, 1H), 8.25 (d, *J* = 8.3 Hz, 1H), 7.95 (d, *J* = 8.0 Hz, 1H), 7.86 (s, 1H), 7.80 (ddd, *J* = 8.3, 7.3, 2.0 Hz, 1H), 7.57 – 7.49 (m, 3H), 7.40 – 7.29 (m, 2H), 7.27 (ddd, *J* = 8.1, 7.1, 1.1 Hz, 1H), 7.19 – 7.11 (m, 2H), 2.44 (s, 3H).

^{13}C NMR (101 MHz, $CDCl_3$) δ 152.4 (C), 149.2 (CH), 138.54 (CH), 138.51 (C), 136.0 (C), 134.8 (C), 128.8 (CH), 128.7 (CH), 127.5 (CH), 125.0 (CH), 123.6 (CH), 123.5 (CH), 121.8 (CH), 121.0 (C), 120.30 (CH), 120.28 (CH), 114.8 (CH), 113.3 (CH), 21.7 (CH_3).

HRMS (EI) *m/z*: [M^+] calcd for $C_{20}H_{16}N_2$ 284.1313, found: 284.1315.

1 mmol Scale Synthesis of 2a

The product was prepared from **1a** according to the general procedure C in 1.0 mmol scale. The reaction was performed in a flask, and a 440 nm Kessil PR 160L lamp at 75% intensity and 4 cm distance was used as the light source. The temperature of the flask was approximately 40 °C. The set-up is shown in Figure S3. The conversion was complete after 112 h, and **2a** was isolated with flash chromatography (silica gel, n-Hex:EtOAc 40:1 → 20:1) as an off-white solid (204 mg, 0.75 mmol, 75%).

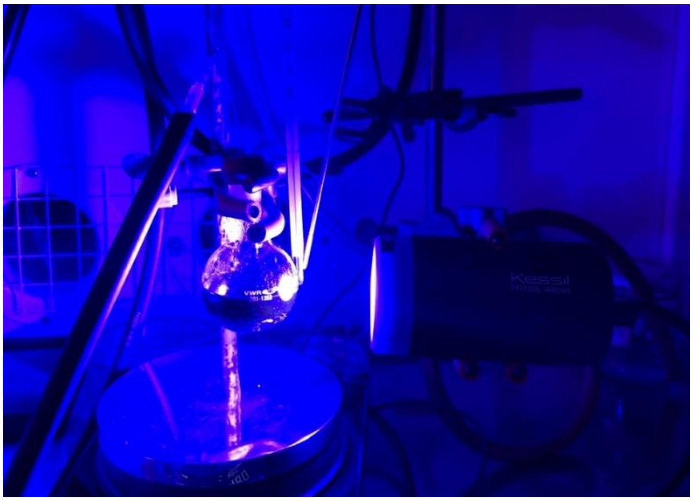

Figure S3. The reaction set-up of the 1 mmol scale reaction.

3-Phenylindole (3a)

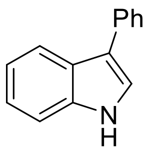

The synthesis was performed following a literature procedure.[15] A pressure vial was charged with **2a** (108 mg, 0.40 mmol), anhydrous DMSO (4 mL), and 5 M NaOMe in MeOH (0.16 mL, 0.80 mmol, 2 equiv). The vial was sealed with a septum, and the solution was bubbled with Ar for 30 min. The reaction mixture was stirred at 120 °C in an oil bath for 16 h, cooled down to r.t., added with water (10 mL) and extracted with EtOAc (3 x 10 mL). The combined organics were dried over Na_2SO_4, and the solvents were evaporated on a rotary evaporator. The crude product was dissolved in DCM, washed with brine (2 x 10 mL), dried over Na_2SO_4, and the solvent was evaporated on a rotary evaporator. The crude product was purified with flash chromatography on silica gel (n-Hex:EtOAc 10:1) to yield 3-phenylindole **3a** as a white solid (64.0 mg, 0.33 mmol, 83%).

^{1}H NMR (400 MHz, $CDCl_3$) δ 8.23 (s, 1H), 7.95 (ddt, *J* = 7.9, 1.5, 0.8 Hz, 1H), 7.71 – 7.64 (m, 2H), 7.50 – 7.40 (m, 3H), 7.37 (d, *J* = 2.5 Hz, 1H), 7.32 – 7.16 (m, 3H).

^{13}C NMR (101 MHz, $CDCl_3$) δ 136.8, 135.7, 128.9, 127.7, 126.13, 125.9, 122.6, 121.9, 120.5, 120.0, 118.6, 111.5.

The characterization data was in agreement with the previous literature.[16]

Unsuitable Substrates

N-(2-Vinylphenyl)pyridin-2-amine **1w**, 2-styrylaniline derivatives ***trans*-1x** and ***trans*-1y**, and *N*-methyl derivatives **1z** and **1aa** were also tested in the photocatalytic cyclization reaction. Amine **1w** did not produce any indole but a [4+2] cycloadduct between PQ-CF_3 and **1w** which was not separated from other side products. With ***trans*-1x** and ***trans*-1y**, no indole formation occurred but *cis–trans* isomerization of the double bond was observed. Substrates **1z** and **1aa** did not produce any indole, but partial demethylation was observed with **1z**.

Reaction with *trans*-1x

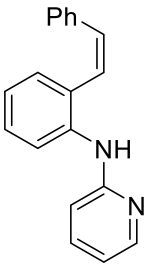

The cyclization was attempted with ***trans*-1x** as a starting material according to the general procedure C. The crude was purified with flash chromatography (silica gel, n-Hex:EtOAc 40:1 → 5:1) to yield 17% unreacted ***trans*-1x** and isomerized ***cis*-1x** as a colorless oil (16.4 mg, 0.06 mmol, 30%).

^{1}H NMR (400 MHz, $CDCl_3$) δ 8.16 (dd, *J* = 5.7, 1.9 Hz, 1H), 7.71 (d, *J* = 8.3 Hz, 1H), 7.41 (ddd, *J* = 8.3, 7.2, 1.9 Hz, 1H), 7.29 – 7.22 (m, 2H), 7.21 – 7.11 (m, 5H), 6.95 (td, *J* = 7.5, 1.2 Hz, 1H), 6.75 – 6.65 (m, 3H), 6.57 (d, *J* = 12.2 Hz, 1H), 6.46 (s, 1H).

^{13}C NMR (101 MHz, $CDCl_3$) δ 155.9 (C), 148.4 (CH), 138.0 (C), 137.6 (CH), 136.5 (C), 132.7 (CH), 130.1 (CH), 129.2 (C), 128.9 (2×CH), 128.4 (2×CH), 128.3 (CH), 127.7 (CH), 126.3 (CH), 122.9 (CH), 120.6 (CH), 115.2 (CH), 108.7 (CH).

HRMS (EI) *m/z*: [M$^+$] calcd for $C_{19}H_{16}N_2$ 272.1313, found: 272.1323.

Reaction with *trans*-1y

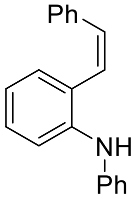

The cyclization was attempted with ***trans*-1y** (41.7 mg, 0.15 mmol) as a starting material according to the general procedure C. The crude was purified with flash chromatography (silica gel, n-Hex:EtOAc 100:0 → 40:1) to yield 24% unreacted ***trans*-1y** and isomerized ***cis*-1y** as a colorless oil (12.1 mg, 0.04 mmol, 29%).

^{1}H NMR (400 MHz, $CDCl_3$) δ 7.29 (d, *J* = 8.1 Hz, 1H), 7.26 – 7.12 (m, 9H), 6.93 – 6.88 (m, 3H), 6.83 (t, *J* = 7.7 Hz, 1H), 6.70 (d, *J* = 12.1 Hz, 1H), 6.57 (d, *J* = 12.1 Hz, 1H), 5.65 (s, 1H).

^{13}C NMR (101 MHz, $CDCl_3$) δ 143.0 (C), 140.7 (C), 136.7 (C), 132.4 (CH), 130.2 (CH), 129.4 (2×CH), 128.8 (2×CH), 128.5 (2×CH), 128.3 (CH), 127.8 (CH), 127.0 (C), 126.6 (CH), 121.3 (CH), 120.8 (CH), 118.6 (2×CH), 116.8 (CH).

HRMS (EI) *m/z*: [M^+] calcd for $C_{20}H_{17}N$ 271.1361, found: 271.1360.

Mechanistic Studies

Radical Trapping with TEMPO and DMPO

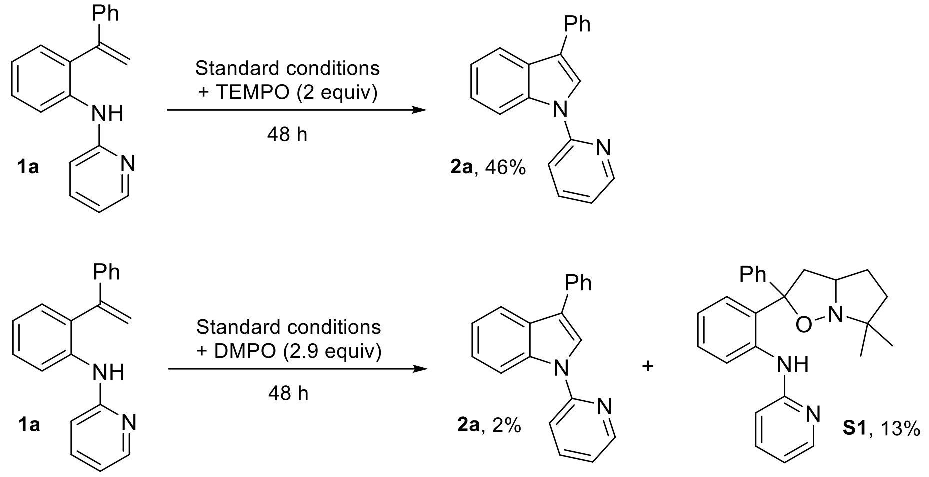

The reaction was performed according to the general procedure C using **1a** as a starting material with (2,2,6,6-tetramethylpiperidin-1-yl)oxyl (TEMPO, 63.0 mg, 0.40 mmol, 2 equiv) added in the reaction mixture. The crude was purified with flash chromatography (silica gel, n-Hex:EtOAc 40:1 → 5:1). The yield of **2a** was 46% (25.0 mg, 0.092 mmol). Unreacted **1a** (19.4 mg, 0.071 mmol, 36%) was also isolated.

Another reaction was performed according to the general procedure C using **1a** as a starting material with (5,5-dimethyl-1-pyrroline-N-oxide (DMPO, 65.7 mg, 0.58 mmol, 2.9 equiv) added in the reaction mixture. The crude was purified with flash chromatography (silica gel, n-Hex:EtOAc 40:1 → 1:1). The yield of **2a** was 2% (1.0 mg, 0.004 mmol). Unreacted **1a** (22.0 mg, 0.081 mmol, 41%) was also isolated. The reaction produced also a DMPO adduct **S1** which was isolated as a pale-yellow solid (10.1 mg, 0.026 mmol, 13%). The adduct **S1** contained two stereoisomers in 85:15 ratio. The NMR data is reported for the major isomer.

***N*-(2-(6,6-Dimethyl-2-phenylhexahydropyrrolo[1,2-b]isoxazol-2-yl)phenyl)pyridin-2-amine (S1)**

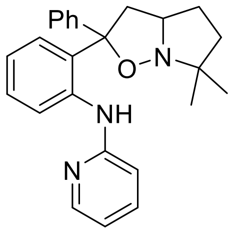

^{1}H NMR (400 MHz, $CDCl_3$) δ 8.07 (ddd, *J* = 5.0, 2.0, 0.9 Hz, 1H), 7.78 (dd, *J* = 8.1, 1.3 Hz, 1H), 7.73 (s, 1H), 7.53 (dd, *J* = 7.9, 1.6 Hz, 1H), 7.42 (dd, *J* = 8.4, 1.3 Hz, 2H), 7.32 – 7.21 (m, 2H), 7.11 (t, *J* = 7.8 Hz, 2H), 7.05 (td, *J* = 7.6, 1.3 Hz, 1H), 7.00 (t, *J* = 7.3 Hz, 1H), 6.57 (ddd, *J* = 7.2, 5.0, 1.0 Hz, 1H), 6.41 (d, *J* = 8.3 Hz, 1H), 3.84 (dddd, *J* = 9.4, 7.6, 5.5, 3.9 Hz, 1H), 3.09 (dd, *J* = 12.2, 7.6 Hz, 1H), 2.84 (dd, *J* = 12.3, 5.6 Hz, 1H), 2.10 – 2.01 (m, 1H), 1.88 (dt, *J* = 12.1, 9.1 Hz, 1H), 1.71 – 1.61 (m, 1H), 1.61 – 1.52 (m, 1H), 1.44 (s, 3H), 1.10 (s, 3H).

^{13}C NMR (101 MHz, $CDCl_3$) δ 156.2 (NH*C*N), 148.1 (CH), 145.5 (C), 139.3 (C), 137.2 (CH), 134.5 (C), 128.4 (CH), 127.8 (2×CH), 126.8 (CH), 126.4 (CH), 126.0 (2×CH), 122.8 (CH), 122.0 (CH), 114.5 (CH), 108.9 (CH), 88.0 (CO), 68.0 ($\mathit{C}(CH_3)_2$), 63.9 (*CH*NO), 49.6 ($C\mathit{CH_2}CH$), 35.9 ($CHCH_2\mathit{CH_2}$), 31.3 ($CH\mathit{CH_2}CH_2$), 27.4 (CH_3), 24.6 (CH_3).

HRMS (EI) *m/z*: [M^+] calcd for $C_{25}H_{27}N_3O$ 385.2154, found: 385.2145.

Reaction Rate Measurements for Hammett Correlation Experiment

The reactions were performed according to the general procedure C with **1a** and **1g**–**k** in a 0.10 mmol scale. The NMR yields were measured after 1 h irradiation using 1,3,5-trimethoxybenzene as an internal standard, and the corresponding reaction rates were calculated from the observed yields.

Table S6. Calculated reaction rates of substrates **1a** and **1g**–**k**.

Substrate	NMR yield after 1 h (%)	Reaction rate k_X (s^{-1})	$\log(k_X/k_H)$
**1a**	13.9	0.00386	-0.56114
**1g**	28.5	0.00792	-0.24931
**1h**	50.6	0.01406	0
**1i**	62.5	0.01736	0.09173
**1j**	52.9	0.01469	0.01931
**1k**	63.8	0.01772	0.10067

H_2 Detection Experiment

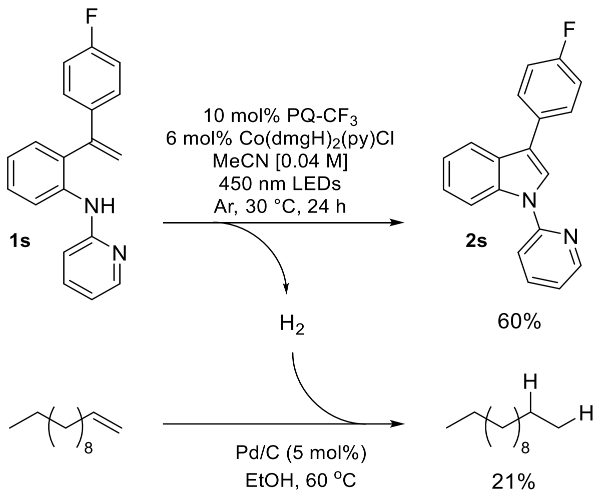

Coupling reaction: The reaction was performed according to the general procedure C with **1s** in a 0.10 mmol scale. The ^1H NMR yield was measured after 24 h irradiation using 1,3,5-trimethoxybenzene as an internal standard. The yield of **2s** was 60%.

Hydrogenation reaction: 5 mol% of Pd/C (5 wt% Pd and 50% wetted powder) was added to a Schlenk flask which was evacuated and backfilled with argon three times. 1-Dodecene (22.2 µL, 0.10 mmol) and degassed EtOH (2 mL) were added under argon flow. The Schlenk flask was connected to the coupling reaction with a silicon tube, and the mixture was heated in an oil bath at 60 °C for 24 h. 1,3,5-Trimethoxybenzene was added as an internal standard, the solvent was evaporated on a rotary evaporator, and the crude product was redissolved in $CDCl_3$. The NMR yield of dodecane was determined to be 21%.

UV-Vis Spectroscopic Measurements

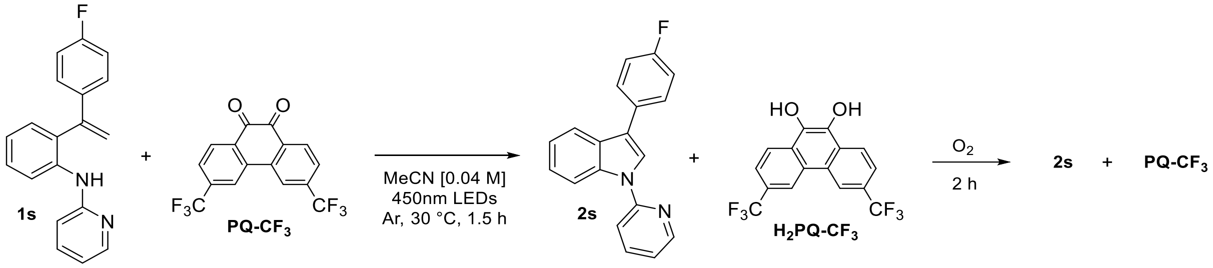

Substrate **1s** (2.9 mg, 0.01 mmol) and PQ-CF_3 (3.4 mg, 0.01 mmol, 1 equiv) were measured in a Schlenk flask which was evacuated and backfilled with argon five times. Degassed MeCN (2.5 mL) was added under argon atmosphere. The mixture was stirred for 30 min at r.t. without irradiation. UV-Vis samples were prepared in 10 × 10 mm light path quartz cuvettes by diluting an aliquot (0.25 mL) of the reaction mixture with MeCN (2.25 mL) under argon atmosphere. No new peaks were observed in the UV-Vis spectra of the reaction mixture, indicating that PQ-$CF_3^{\bullet-}$ was not present in the samples (Figure S4).

Next, the reaction mixture was irradiated with blue LEDs (450 nm) for 1.5 h while stirring at 30 °C. UV-Vis spectra of the mixture were measured after 15 min, 30 min, and 1.5 h irradiation. The spectra indicated the disappearance of PQ-CF_3 and the appearance of H_2PQ-CF_3, but no additional peaks were detected. After the irradiation, the Schlenk flask was exposed to air for 2 h. The UV-Vis spectrum showed the recovery of PQ-CF_3 (Figure S4).

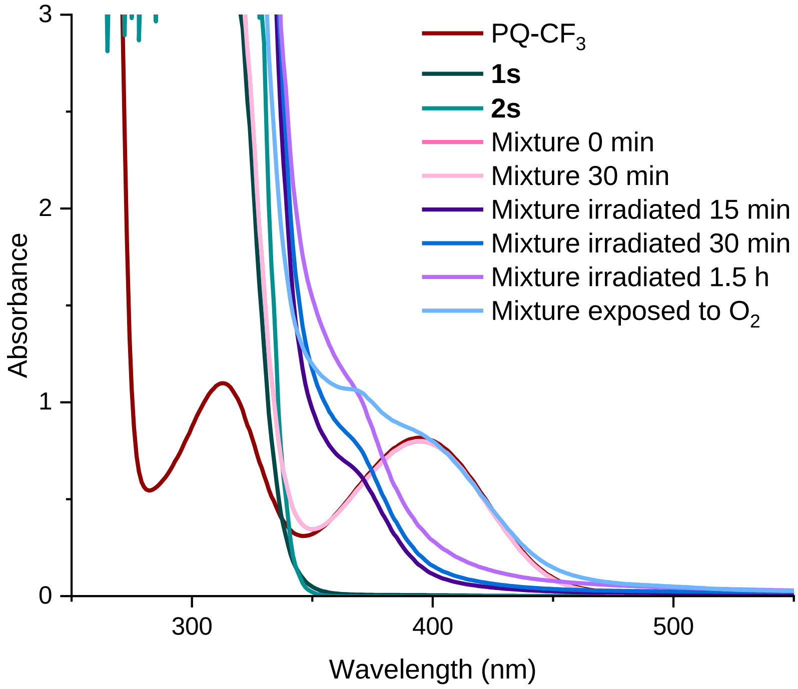

Figure S4. The UV-Vis spectra of **1s**, **2s**, PQ-CF_3, and their mixture before and after irradiation, and after air exposure.

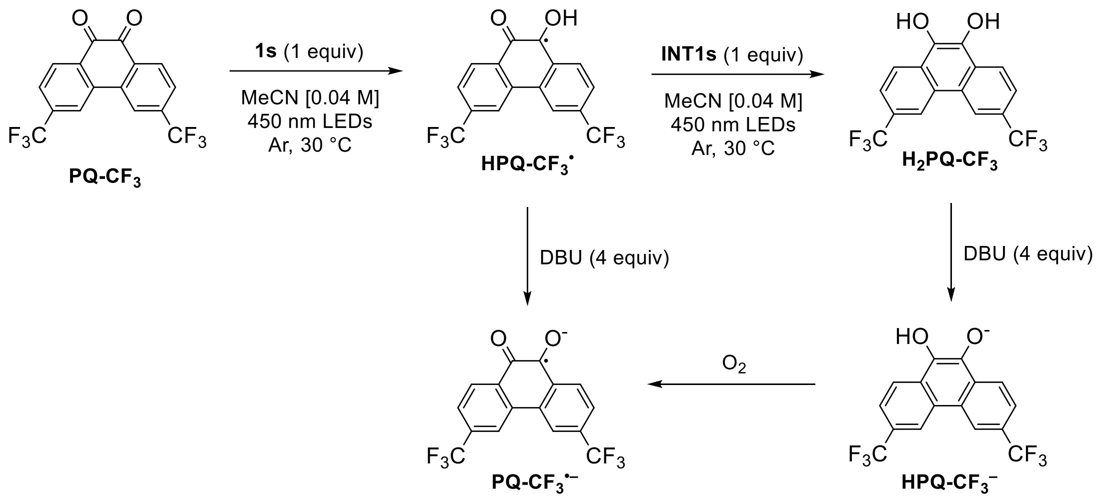

For further mechanistic evidence, another reaction was performed with **1s** and PQ-CF_3 and irradiated for 15 min, after which DBU (4 equiv) was added to the reaction mixture. Two new signals appeared in the UV-Vis spectrum after the base addition (Figure S5). The signal around 400 nm resulted from HPQ-CF_3^- and the signal at 570 nm resulted from PQ-$CF_3^{\bullet-}$,[17] which indicates that both H_2PQ-CF_3 and HPQ-$CF_3^\bullet$ are present under neutral reaction conditions. After exposing the DBU-containing reaction mixture to air, the signal of HPQ-CF_3^- disappeared while the signal of PQ-$CF_3^{\bullet-}$ grew stronger due to oxidation by O_2 (Figure S5).

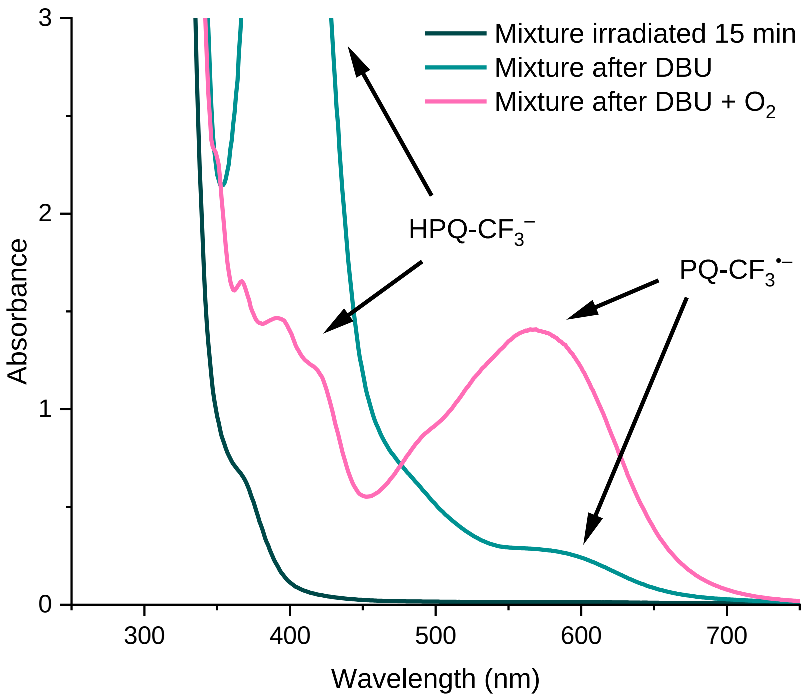

Figure S5. The UV-Vis spectra of the mixture of **1s** and PQ-CF_3 after irradiation, after the base addition and after air exposure.

Stock solutions were prepared of the substrate **1a** (2.7 mg, 0.01 mmol in 5.0 mL MeCN), PQ-CF_3 (3.4 mg, 0.01 mmol in 5.0 mL MeCN), and Co(dmgH)$_2$(py)Cl (4.0 mg, 0.01 mmol in 5.0 mL MeCN). These solutions were diluted with MeCN so that the final concentrations of **1a**, PQ-CF_3, and Co(dmgH)$_2$(py)Cl in each sample were 0.1 mM, 0.1 mM, and 0.06 mM, respectively. UV-Vis spectra were measured for each compound individually and for their mixtures (Figure S6). No clear spectral evidence of interactions between the compounds was observed.

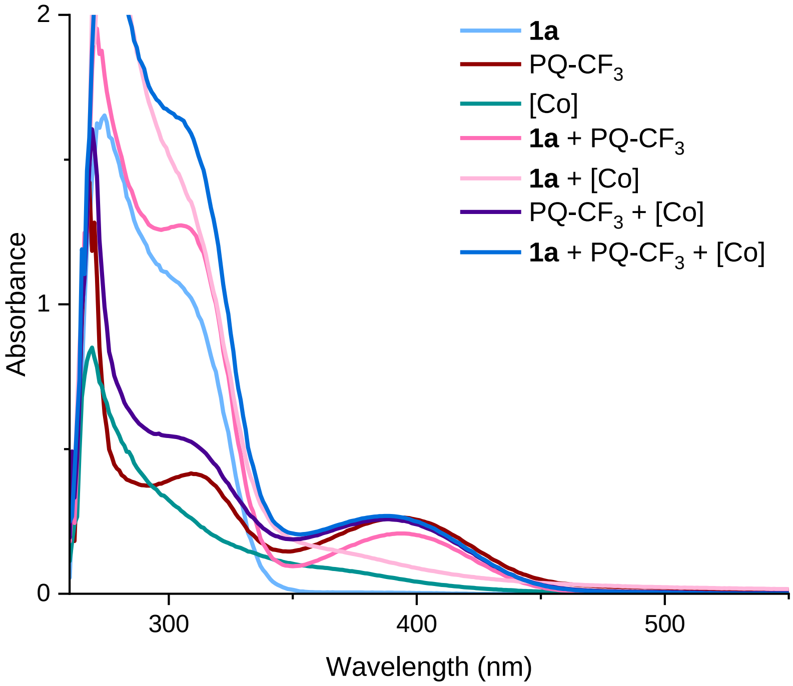

Figure S6. The UV-Vis spectra of **1a**, PQ-CF_3, Co(dmgH)$_2$(py)Cl (marked as [Co]), and their mixtures.

To mimic the reaction conditions, a sample was prepared from the same stock solutions so that the concentrations of **1a**, PQ-CF_3, and Co(dmgH)$_2$(py)Cl were 0.1 mM, 0.01 mM, and 0.006 mM, respectively. The sample was fitted with a septum, sealed with parafilm, and the mixture was bubbled through a needle with argon for 30 min and irradiated with blue LEDs (450 nm) at 30 °C for 30 min while bubbling with argon. The UV-Vis spectrum of the mixture was measured before and after irradiation. The only new peaks observed corresponded to the product **2a** (Figure S7).

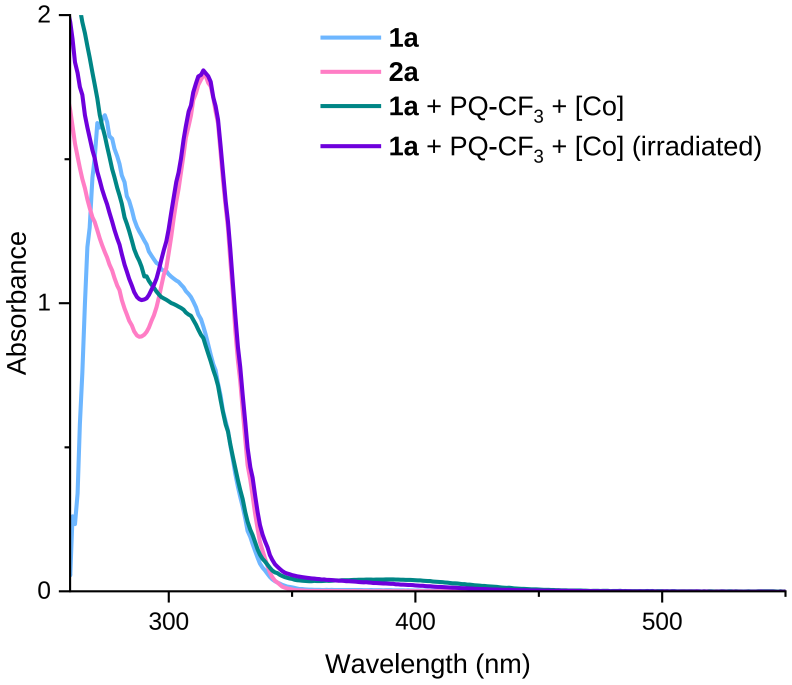

Figure S7. The UV-Vis spectra of **1a**, **2a**, and the mixture of **1a** [0.1 mM], PQ-CF_3 [0.01 mM], and $Co(dmgH)_2(py)Cl$ [0.006 mM] (marked as [Co]) before and after irradiation.

Computational Studies

The geometry optimizations were performed either with UB3LYP[18] density functional utilizing a 6-31G+(d,p) basis set using Grimme's empirical dispersion (GD3BJ)[19] for dipolar interactions or with UM06-2X[20] functional using def2-tzvp basis set and CPCM[21] solvation model for acetonitrile included in the Gaussian 16 program package.[22] The initial geometries for transition state (TS) search (Berny algorithm – calculated force constants) were found by saddle point crossing distance scans. Intrinsic reaction coordinate (IRC) calculations were done for TS structures (one imaginary frequency) to check their relevance to each reaction step.

Cartesian coordinates (XYZ) of geometry-optimized structures are listed in a separate file (cartesian.xyz) together with their free energies and optional imaginary frequencies (for TSs).

Oxidation potentials (Table S6) are calculated with the method B3LYP 6-31G+(d,p) CPCM(MeCN) developed by Nicewicz and co-workers[23] including additionally Grimme's dispersion interactions (GD3BJ). Manual conformation search was performed for the computed structures.

Table S7. Calculated oxidation potentials of **1a**–**d** and **1g**–**x**. Computed with B3LYP 6-31G+(d,p) CPCM(MeCN) including dispersions (GD3BJ).

	**Structure**							
	**1a**	**1g**	**1h**	**1i**	**1j**	**1k**	**1l**	**1m-1**
radical cation (a.u.)[a]	-842.848514	-957.36697	-882.15465	-1302.457414	-3413.994435	-942.097898	-1071.873128	-957.361931
neutral (a.u.)[b]	-843.05035	-957.557174	-882.35159	-1302.660138	-3414.196136	-942.299902	-1072.058307	-957.559059
ΔG	126.654028	119.354836	123.58237	127.2112562	126.5693138	126.7594492	116.2016002	123.6997124
ΔG/F	5.49213077	5.175614065	5.3589336	5.516294009	5.488457301	5.496702192	5.038879503	5.364022047
$E^{o}_{1/2}$= Norm-SHE	1.21113077	0.894614065	1.0779336	1.235294009	1.207457301	1.215702192	0.757879503	1.083022047
$E^{o}_{1/2}$= Norm-SCE	1.07013077	0.753614065	0.9369336	1.094294009	1.066457301	1.074702192	0.616879503	0.942022047
$E^{o}_{1/2}$= Norm-Fc(0.64)	0.57113077	0.254614065	0.4379336	0.595294009	0.567457301	0.575702192	0.117879503	0.443022047

	**Structure**							
	**1n**	**1o**	**1p**	**1q**	**1r**	**1s**	**1t**	**1u**
radical cation (a.u.)[a]	-942.095428	-942.093	-957.358032	-976.793	-882.151803	-942.098338	-1179.915436	-882.146677
neutral (a.u.)[b]	-942.300998	-942.298	-957.558738	-976.977	-882.351715	-942.300688	-1180.118712	-882.347172
ΔG	128.9971485	128.8371	125.9449418	115.7059	125.4466992	126.9765676	127.5576414	125.8125373
ΔG/F	5.593736112	5.586797	5.461382498	5.017383	5.439777076	5.506117148	5.531314403	5.455641007
$E^{o}_{1/2}$= Norm-SHE	1.312736112	1.305797	1.180382498	0.736383	1.158777076	1.225117148	1.250314403	1.174641007
$E^{o}_{1/2}$= Norm-SCE	1.171736112	1.164797	1.039382498	0.595383	1.017777076	1.084117148	1.109314403	1.033641007
$E^{o}_{1/2}$= Norm-Fc(0.64)	0.672736112	0.665797	0.540382498	0.096383	0.518777076	0.585117148	0.610314403	0.534641007

	**Structure**					
	**1v**	**1b**	**1c**	**1x**	**1y**	**1d**
radical cation (a.u.)[a]	-882.151548	-957.362	-826.800938	-842.854997	-826.806338	-651.124945
neutral (a.u.)[b]	-882.352446	-957.554	-826.994872	-843.052523	-826.997079	-651.322955
ΔG	126.0654236	120.7216	121.6954468	123.9494612	119.6918086	124.2531759
ΔG/F	5.466606982	5.234879	5.277110566	5.374851969	5.190226296	5.388022024
$E^{o}_{1/2}$= Norm-SHE	1.185606982	0.953879	0.996110566	1.093851969	0.909226296	1.107022024
$E^{o}_{1/2}$= Norm-SCE	1.044606982	0.812879	0.855110566	0.952851969	0.768226296	0.966022024
$E^{o}_{1/2}$= Norm-Fc(0.64)	0.545606982	0.313879	0.356110566	0.453851969	0.269226296	0.467022024

[a]XYZ coordinates are stored with code **1**"**a**-**z**"-rc; [b]XYZ coordinates are stored with code **1**"**a**-**z**"-N.

Table S8. Calculated bond-dissociation energies (BDEs) and bond-dissociation free energies (BDFEs) of **1a**–**b** and **1g**–**x**. Computed with M062-2X/def2-tzvp (N = neutral compound, rN = neutral N-radical).

**Structure**[a]	ΔH	ΔG	**Structure**[a]	ΔH	ΔG
**1a**-N (a.u.)	-842.784812	-842.847169	**1p**-N (a.u.)	-957.275837	-957.343682
**1a**-rN (a.u.)	-842.141652	-842.206225	**1p**-rN (a.u.)	-956.631924	-956.702629
BD(F)E (kcal/mol)	92.48362105	82.92602221	BD(F)E (kcal/mol)	92.95613578	82.99442075
**1g**-N (a.u.)	-957.274414	-957.342413	**1q**-N (a.u.)	-976.665237	-976.736266
**1g**-rN (a.u.)	-956.635246	-956.705924	**1q**-rN (a.u.)	-976.019738	-976.092764
BD(F)E (kcal/mol)	89.97860269	80.1304669	BD(F)E (kcal/mol)	93.95136602	84.53119178
**1h**-N (a.u.)	-882.06586	-882.133619	**1r**-N (a.u.)	-882.066908	-882.133072
**1h**-rN (a.u.)	-881.4239	-881.493599	**1r**-rN (a.u.)	-881.423662	-881.490953
BD(F)E (kcal/mol)	91.73060952	82.34620333	BD(F)E (kcal/mol)	92.53758687	83.663346
**1i**-N (a.u.)	-1302.397411	-1302.463604	**1s**-N (a.u.)	-942.04676	-942.111321
**1i**-rN (a.u.)	-1301.75495	-1301.82321	**1s**-rN (a.u.)	-941.403241	-941.470014
BD(F)E (kcal/mol)	92.04499183	82.58089192	BD(F)E (kcal/mol)	92.708897	83.15380819
**1j**-N (a.u.)	-3416.391199	-3416.458741	**1t**-N (a.u.)	-1179.871401	-1179.942923
**1j**-rN (a.u.)	-3415.748462	-3415.817908	**1t**-rN (a.u.)	-1179.227356	-1179.301006
BD(F)E (kcal/mol)	92.21818448	82.85636864	BD(F)E (kcal/mol)	93.03896705	83.53658906
**1k**-N (a.u.)	-942.045558	-942.109913	**1u**-N (a.u.)	-882.062806	-882.128104
**1k**-rN (a.u.)	-941.403661	-941.470867	**1u**-rN (a.u.)	-881.421413	-881.489174
BD(F)E (kcal/mol)	91.69107641	81.73500897	BD(F)E (kcal/mol)	91.37481157	81.66221786
**1l**-N (a.u.)	-1071.756072	-1071.831862	**1v**-N (a.u.)	-882.066215	-882.133941
**1l**-rN (a.u.)	-1071.115445	-1071.19294	**1v**-rN (a.u.)	-881.422659	-881.492738
BD(F)E (kcal/mol)	90.89413921	81.65719778	BD(F)E (kcal/mol)	92.73211485	83.0885472
**1m-1**-N (a.u.)	-957.27611	-957.345004	**1b**-N (a.u.)	-957.268159	-957.338784
**1m-1**-rN (a.u.)	-956.63326	-956.703642	**1b**-rN (a.u.)	-956.63282	-956.703195
BD(F)E (kcal/mol)	92.28909307	83.18832122	BD(F)E (kcal/mol)	87.5758684	79.56570826
**1n**-N (a.u.)	-942.046254	-942.111335	**1x**-N (a.u.)	-842.784225	-842.85024
**1n**-rN (a.u.)	-941.402444	-941.47	**1x**-rN (a.u.)	-842.141871	-842.207642
BD(F)E (kcal/mol)	92.89150229	83.17137846	BD(F)E (kcal/mol)	91.9778483	83.9639231
**1o**-N (a.u.)	-942.044041	-942.108406	**1y**-N (a.u.)	-826.72196	-826.787126
**1o**-rN (a.u.)	-941.399919	-941.466809	**1y**-rN (a.u.)	-826.088699	-826.153889
BD(F)E (kcal/mol)	93.08728529	83.33578598	BD(F)E (kcal/mol)	86.27190344	78.08980566
Hydrogen (a.u.)	-0.495778	-0.508793			

[a]XYZ coordinates are stored with the same names.

Table S9. Calculated reaction barriers (ΔTS) for the radical cation (rc) and the neutral radical (rN) of **1a**, **1g**–**l**, **1k**, and **1m-1**. Computed with UM06-2X/def2-tzvp/CPCM(MeCN).

**Structure**[a]	ΔH	ΔG	ifreq	**Structure**[a]	ΔH	ΔG	ifreq
**1a**-rN (a.u.)	-842.150798	-842.150798		**1g**-rN (a.u.)	-956.647608	-956.718169	
**1a**-rN-TS (a.u.)	-842.130109	-842.191954	-514.35	**1g**-rN-TS (a.u.)	-956.620360	-956.688200	-542.56
ΔTS (kcal/mol)	13.0	**14.5**		ΔTS (kcal/mol)	17.0	**18.8**	
**1a**-rc (a.u.)	-842.580189	-842.644674		**1g**-rc (a.u.)	-957.082908	-957.153880	
**1a**-rc-TS (a.u.)	-842.557583	-842.619216	-349.84	**1g**-rc-TS (a.u.)	-957.049710	-957.117208	-378.74
Δ TS (kcal/mol)	14.2	**15.9**		Δ TS (kcal/mol)	20.8	**23.0**	
**1h**-rN (a.u.)	-881.43433	-881,503839		**1i**-rN (a.u.)	-1301.763380	-1301.831147	
**1h**-rN-TS (a.u.)	-881.411962	-881,478017	-502.62	**1i**-rN-TS (a.u.)	-1301.742216	-1301.807548	-519.82
ΔTS (kcal/mol)	14.03613473	**16.2**		ΔTS (kcal/mol)	13.2	**14.8**	
**1h**-rc (a.u.)	-881.86545	-881,935645		**1i**-rc (a.u.)	-1302.191359	-1302.259606	
**1h**-rc-TS (a.u.)	-881.840414	-881,906986	-351.22	**1i**-rc-TS (a.u.)	-1302.166862	-1302.231851	-358.93

Δ TS (kcal/mol)	15.7	**18.0**		Δ TS (kcal/mol)	15.4	**17.4**	
**1k**-rN (a.u.)	-941.412352	-941.478707		**1m-1**-rN (a.u.)	-956.643710	-956.713880	
**1k**-rN-TS (a.u.)	-941.390420	-941.454260	-523.20	**1m-1**-rN-TS (a.u.)	-956.622735	-956.690404	-469.59
ΔTS (kcal/mol)	13.8	**15.3**		ΔTS (kcal/mol)	13.2	**14.7**	
**1k**-rc (a.u.)	-941.841009	-941.907458		**1m-1**-rc (a.u.)	-957.072513	-957.143481	
**1k**-rc-TS (a.u.)	-941.816310	-941.880039	-360.90	**1m-1**-rc-TS (a.u.)	-957.052069	-957.119501	-340.04
Δ TS (kcal/mol)	15.5	**17.2**		Δ TS (kcal/mol)	12.8	**15.0**	

[a]XYZ coordinates are stored with the same names.

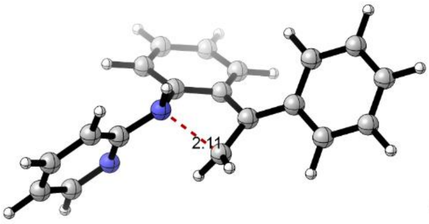

1a radical cation TS

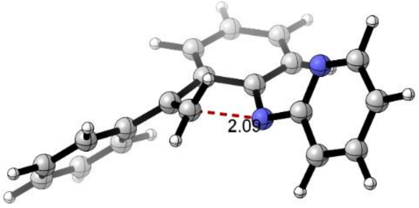

1a neutral radical TS

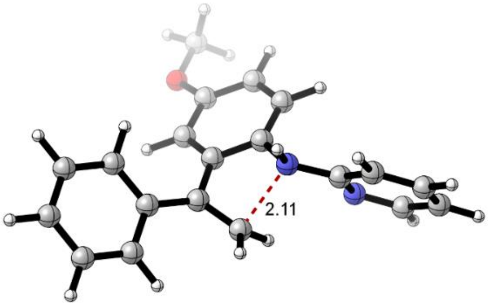

1g radical cation TS

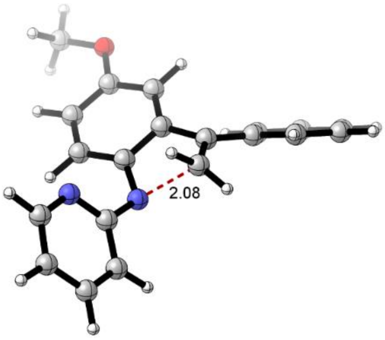

1g neutral radical TS

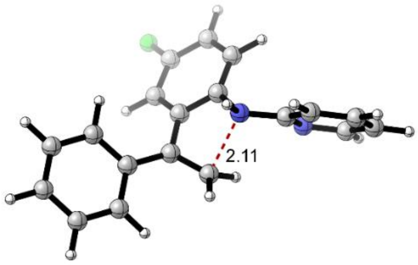

1k radical cation TS

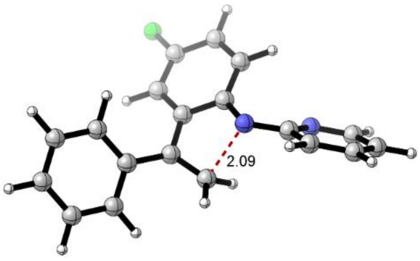

1k neutral radical TS

Figure S8. Computed radical cation and neutral radical transition states for selected substrates.

Hydrogen abstraction energetics by triplet state PQs

Our previous photophysical studies revealed long 6 ms lifetimes for the excited triplet states of both PQ and PQ-CF_3 measured at low T (100 K).[1] As PQs showed no fluorescence emission, we have herein focused on studying the triplet state interactions of catalysts (^{3}PQ/^{3}PQ-CF_3) and substrate **1a**.

We computed energy profiles (ΔG, ΔH) with UB3LYP/6-31+G(d,p) empiricaldispersion=GD3BJ CPCM(MeCN) for N–H abstraction by the triplet state ^{3}PQ/^{3}PQ-CF_3 (Figure S7). The ground state

optimization of **1a** + PQ/PQ-CF_3 complexes showed only very weak H-bonding associated with minor changes in the free energies of the complexes: ΔG of -0.3 kcal/mol (2.09 Å) and +0.8 kcal/mol (2.11 Å) for PQ and PQ-CF_3, respectively. However, the corresponding triplet state complexes showed strong hydrogen bonding as N–H bond lengths to the carbonyl oxygen were 1.56 Å and 1.60 Å for PQ and PQ-CF_3, respectively. We were also able to locate very low free energy barrier transition states ($\Delta G^{\ddagger}$) of 0.5 and 2.1 kcal/mol for hydrogen atom abstraction by ^{3}PQ and ^{3}PQ-CF_3, respectively (Figures S7 and S8). The triplet state reaction energy for the formation of the radical intermediate **INT1a** is slightly exergonic (ΔG = -1.0 kcal/mol) for PQ, while it is endergonic with 3.9 kcal/mol for PQ-CF_3. The energy landscape indicates that the H abstractions by both ^{3}PQ* and ^{3}PQ-CF_3* are diffusion-controlled processes.

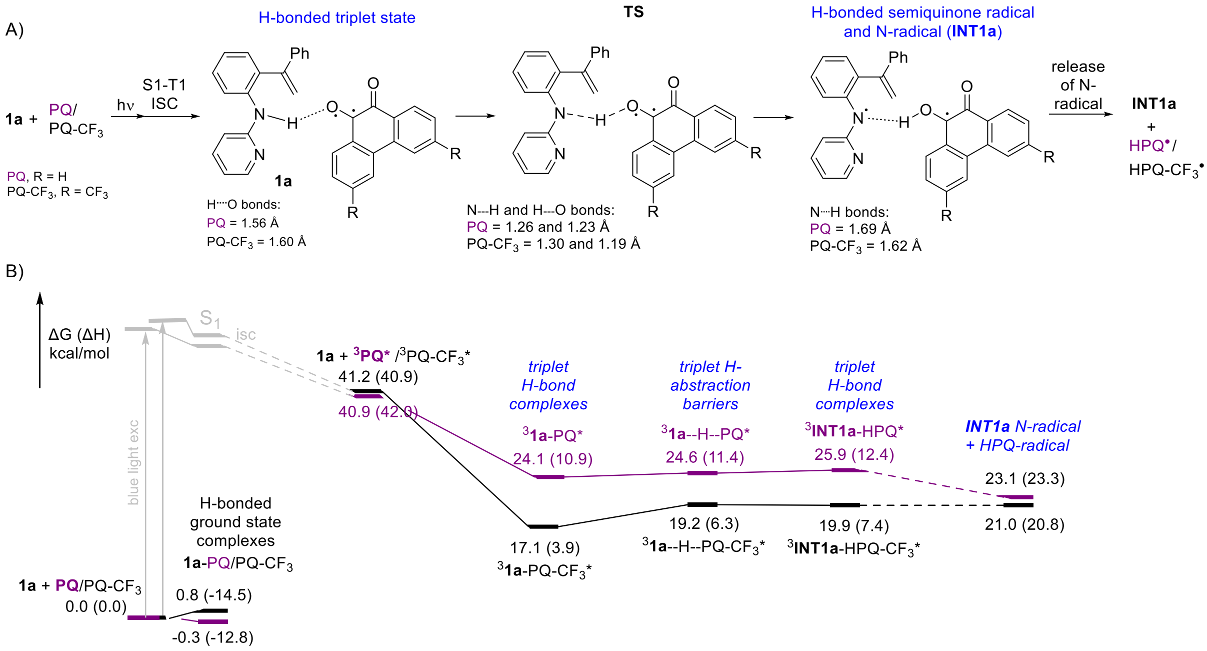

Figure S9. A) Mechanistic proposal for ^{3}PQ* and ^{3}PQ-CF_3 mediated N-H abstraction. B) Computed energy profiles for N–H abstraction by ^{3}PQ* and ^{3}PQ-CF_3.

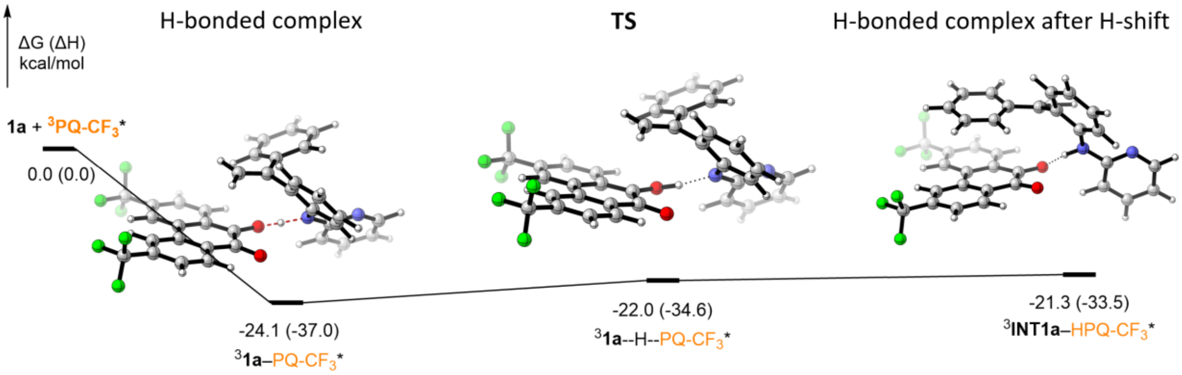

Figure S10. DFT energy profile for the formation of N-radical **INT1** and semiquinone HPQ-$CF_3^{\bullet}$ via HAT from **1a** to PQ-CF_3 normalized to **1a** + ^{3}PQCF_3*.

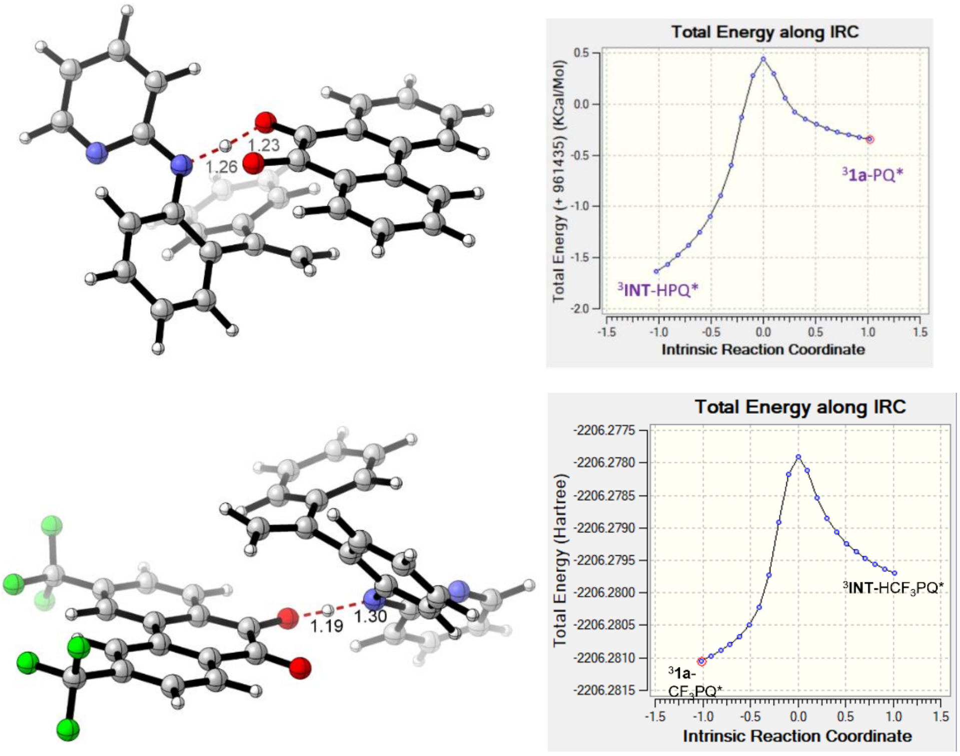

Figure S11. IRC profiles for the found triplet TS structures for the H-shift between **1a** and PQ (above) or PQ-CF_3 (below).

Table S10. Triplet state calculations for the N–H abstraction by ^{3}PQ* and ^{3}PQ-CF_3*. Computed with UB3LYP/6-31+G(d,p) GD3BJ CPCM(MeCN).

**Structure**	ΔG	ΔH	ifreq	xyz-name
H-bonded **1a**-PQ (ground state)	-1531.773849	-1531.678427	-	1a-PQ
H-bonded **1a**-PQ (triplet)	-1531.734977	-1531.640616	-	1a-PQ-T
**1a**-PQ-TS H-shift (triplet)	-1531.734177	-1531.639760	-1186.24	1a-PQ-TS-T
**INT1a**-HPQ (triplet)	-1531.732087	-1531.638172	-	INT-HPQ-T
**INT1a** (N-radical)	-842.413453	-842.348772	-	INT1a
HPQ (semiquinone radical)	-689.322666	-689.272085	-	HPQ
PQ-triplet	-688.660051	-688.610347	-	PQ-T
H-bonded **1a**-PQ-CF_3 (ground state)	-2205.901560	-2205.790100	-	1a-PQCF_3
H-bonded **1a**-PQ-CF_3 (triplet)	-2205.875623	-2205.760809	-	1a-PQ CF_3-T
**1a**-PQ-CF_3-TS H-shift (triplet)	-2205.872128	-2205.757002	-1109.53	1a-PQCF_3-TS-T
**INT1a**-HPQ-CF_3 (triplet)	-2205. 871108	-2205.757002	-	INT-HPQ CF_3-T
HCF_3PQ (semiquinone radical)	-1363.455909	-1363.385019	-	HPQCF_3
PQ-CF_3-triplet	-1362.789906	-1362.789906	-	PQCF_3-T
PQ	-688.725688	-688.675570	-	PQ
PQ-CF_3	-1362.855061	-1362.784592	-	PQ-CF_3
**1a**	-843.047743	-842.982401	-	1a

Bibliography

(1) Talvitie, J.; Alanko, I.; Lenarda, A.; Durandin, N.; Tkachenko, N.; Nieger, M.; Helaja, J. Electron-Deficient Phenanthrenequinone Derivative for Photoactivated Hydrogen Atom Transfer Mediated Oxidation of Secondary Alcohols. *ChemPhotoChem* **2023**, *7* (10), e202300107. https://doi.org/10.1002/cptc.202300107.

(2) Chatupheeraphat, A.; Rueping, M.; Magre, M. Chemo- And Regioselective Magnesium-Catalyzed Ortho-Alkenylation of Anilines. *Org. Lett.* **2019**, *21* (22), 9153–9157. https://doi.org/10.1021/acs.orglett.9b03526.

(3) Mäkelä, M. K.; Bulatov, E.; Malinen, K.; Talvitie, J.; Nieger, M.; Melchionna, M.; Lenarda, A.; Hu, T.; Wirtanen, T.; Helaja, J. Carbocatalytic Cascade Synthesis of Polysubstituted Quinolines from Aldehydes and 2-Vinyl Anilines. *Adv. Synth. Catal.* **2021**, *363* (15), 3775–3782. https://doi.org/10.1002/adsc.202100711.

(4) Fors, B. P.; Krattiger, P.; Strieter, E.; Buchwald, S. L. Water-Mediated Catalyst Preactivation: An Efficient Protocol for C-N Cross-Coupling Reactions. *Org. Lett.* **2008**, *10* (16), 3505–3508. https://doi.org/10.1021/acs.orglett.2c02510.

(5) Zhang, R.; Zhao, Y.; Li, G.; Yang, D.; Ni, Z. A New Series of Pyrenyl-Based Triarylamines: Syntheses, Structures, Optical Properties, Electrochemistry and Electroluminescence. *RSC Adv.* **2016**, *6* (11), 9037–9048. https://doi.org/10.1039/c5ra26017e.

(6) Kobayashi, K.; Miyamoto, K.; Yamase, T.; Nakamura, D.; Morikawa, O.; Konishi, H. Synthesis of 1-Aryl-1H-Indole Derivatives by Iodine-Mediated Cyclization of 2-(Arylamino)Styrene Derivatives. *Bull. Chem. Soc. Jpn.* **2006**, *79* (10), 1580–1584. https://doi.org/10.1246/bcsj.79.1580.

(7) Antilla, J. C.; Buchwald, S. L. Copper-Catalyzed Coupling of Arylboronic Acids and Amines. *Org. Lett.* **2001**, *3* (13), 2077–2079. https://doi.org/10.1021/ol0160396.

(8) Hao, W.; Li, K.; Ye, C.; Yu, W.; Chang, J. Iodine-Mediated C=C Double Bond Cleavage toward Pyrido[2,1- b]Quinazolinones. *Org. Lett.* **2022**, *24* (17), 3286–3290. https://doi.org/10.1021/acs.orglett.2c01183.

(9) Torigoe, T.; Ohmura, T.; Suginome, M. Asymmetric Cycloisomerization of o-Alkenyl-N-Methylanilines to Indolines by Iridium-Catalyzed C(sp3)−H Addition to Carbon–Carbon Double Bonds, *Angew. Chem. Int. Ed.* **2017**, *56* (45), 14272–14276. https://doi.org/10.1002/anie.201708578.

(10) Ru, T.; Ning, Y.; Liu, D.; Tao, Y.; Wang, J.; Chen, F-E. Hydrogen-Free Palladium-Catalyzed Intramolecular Anti-Markovnikov Hydroaminocarbonylation of 2-(1-Methylvinyl)anilines, *Chem. Commun.*, **2023**, *59* (25), 3755-3758. https://doi.org/10.1039/d2cc06836b.

(11) Hu, X.; Tan, Z.; Xiong, L.; Yang, C.; Jiang, H.; Zeng, W. Rh(III)-Catalyzed Csp2-Csp3σ-Bond Enolation of α-Indolyl Alcohols. *Org. Lett.* **2021**, *23* (10), 3965–3969. https://doi.org/10.1021/acs.orglett.1c01132.

(12) Chen, J.; Chang, D.; Xiao, F.; Deng, G.-J. Three-Component Ordered Annulation of Amines, Ketones, and Nitrovinylarenes: Access to Fused Pyrroles and Substituted Indoles under Metal-Free Conditions. *J. Org. Chem.* **2019**, *84* (2), 568–578. https://doi.org/10.1021/acs.joc.8b02410.

(13) Whyte, A.; Torelli, A.; Mirabi, B.; Prieto, L.; Rodríguez, J. F.; Lautens, M. Cobalt-Catalyzed

Enantioselective Hydroarylation of 1,6-Enynes. *J. Am. Chem. Soc.* **2020**, *142* (20), 9510–9517. https://doi.org/10.1021/jacs.0c03246.

(14) Zheng, C. H. M.; Balatsky, D. A.; Dipucchio, R. C.; Schafer, L. L. The Catalytic Synthesis of N-Aryl Indoles Featuring an Alternative Disconnection. Hydroaminoalkylation for a Telescoped Reaction Sequence. *Org. Lett.* **2022**, *24* (36), 6571–6575. https://doi.org/10.1021/acs.orglett.2c02510.

(15) Xu, W.; Hei, Y.-Y.; Song, J.-L.; Zhan, X.-C.; Zhang, X.-G.; Deng, C.-L. Copper(I)-Catalyzed Thiolation of C–H Bonds for the Synthesis of Sulfenyl Pyrroles and Indoles. *Synthesis (Stuttg).* **2019**, *51* (02), 545–551. https://doi.org/10.1055/s-0037-1610295.

(16) Lan, H.; Huo, X.; Jia, Y.; Wang, D. Silyl Radical Generation from Silylboronic Pinacol Esters through Substitution with Aminyl Radicals. *Org. Lett.* **2024**, *26* (5), 1011–1016. https://doi.org/10.1021/acs.orglett.3c04085.

(17) Mizen, M. B.; Wrighton, M. S. Reductive Addition of CO_2 to 9,10-Phenanthrenequinone. *J. Electrochem. Soc.* **1989**, *136* (4), 941–946. https://doi.org/10.1149/1.2096891.

(18) Becke, A. D. A New Mixing of Hartree–Fock and Local Density-functional Theories. *J. Chem. Phys.* **1993**, *98* (2), 1372–1377. https://doi.org/10.1063/1.464304.

(19) Grimme, S.; Antony, J.; Ehrlich, S.; Krieg, H. A Consistent and Accurate Ab Initio Parametrization of Density Functional Dispersion Correction (DFT-D) for the 94 Elements H-Pu. *J. Chem. Phys.* **2010**, *132* (15), 154104. https://doi.org/10.1063/1.3382344.

(20) Zhao, Y.; Truhlar, D. G. The M06 Suite of Density Functionals for Main Group Thermochemistry, Thermochemical Kinetics, Noncovalent Interactions, Excited States, and Transition Elements: Two New Functionals and Systematic Testing of Four M06-Class Functionals and 12 Other Function. *Theor. Chem. Acc.* **2008**, *120* (1–3), 215–241. https://doi.org/10.1007/s00214-007-0310-x.

(21) Barone, V.; Cossi, M. Quantum Calculation of Molecular Energies and Energy Gradients in Solution by a Conductor Solvent Model. *J. Phys. Chem. A* **1998**, *102* (11), 1995–2001. https://doi.org/10.1021/jp9716997.

(22) Frisch, M. J.; Trucks, G. W.; Schlegel, H. B.; Scuseria, G. E.; Robb, M. A.; Cheeseman, J. R.; Scalmani, G.; Barone, V.; Petersson, G. A.; Nakatsuji, H.; Li, X.; Caricato, M.; Marenich, A. V.; Bloino, J.; Janesko, B. G.; Gomperts, R.; Mennucci, B.; Hratch, D. J. Gaussian 16. Gaussian, Inc., Wallingford CT 2016.

(23) Roth, H. G.; Romero, N. A.; Nicewicz, D. A. Experimental and Calculated Electrochemical Potentials of Common Organic Molecules for Applications to Single-Electron Redox Chemistry. *Synlett* **2016**, *27* (5), 714–723. https://doi.org/10.1055/s-0035-1561297.

Copies of NMR Spectra

^{1}H NMR (400 MHz, $CDCl_3$) of **1a**

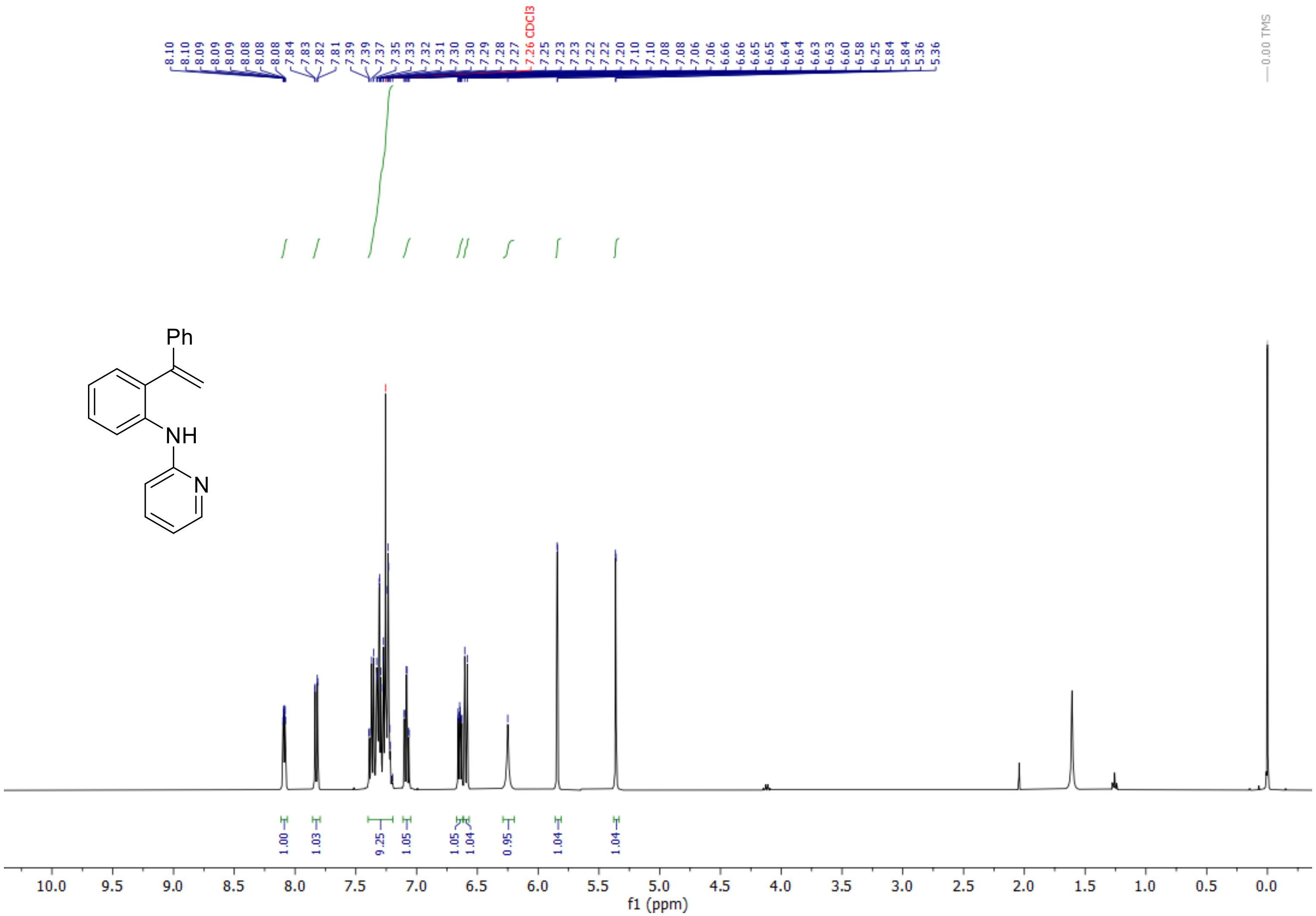

^{13}C NMR (101 MHz, $CDCl_3$) of **1a**

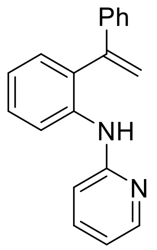

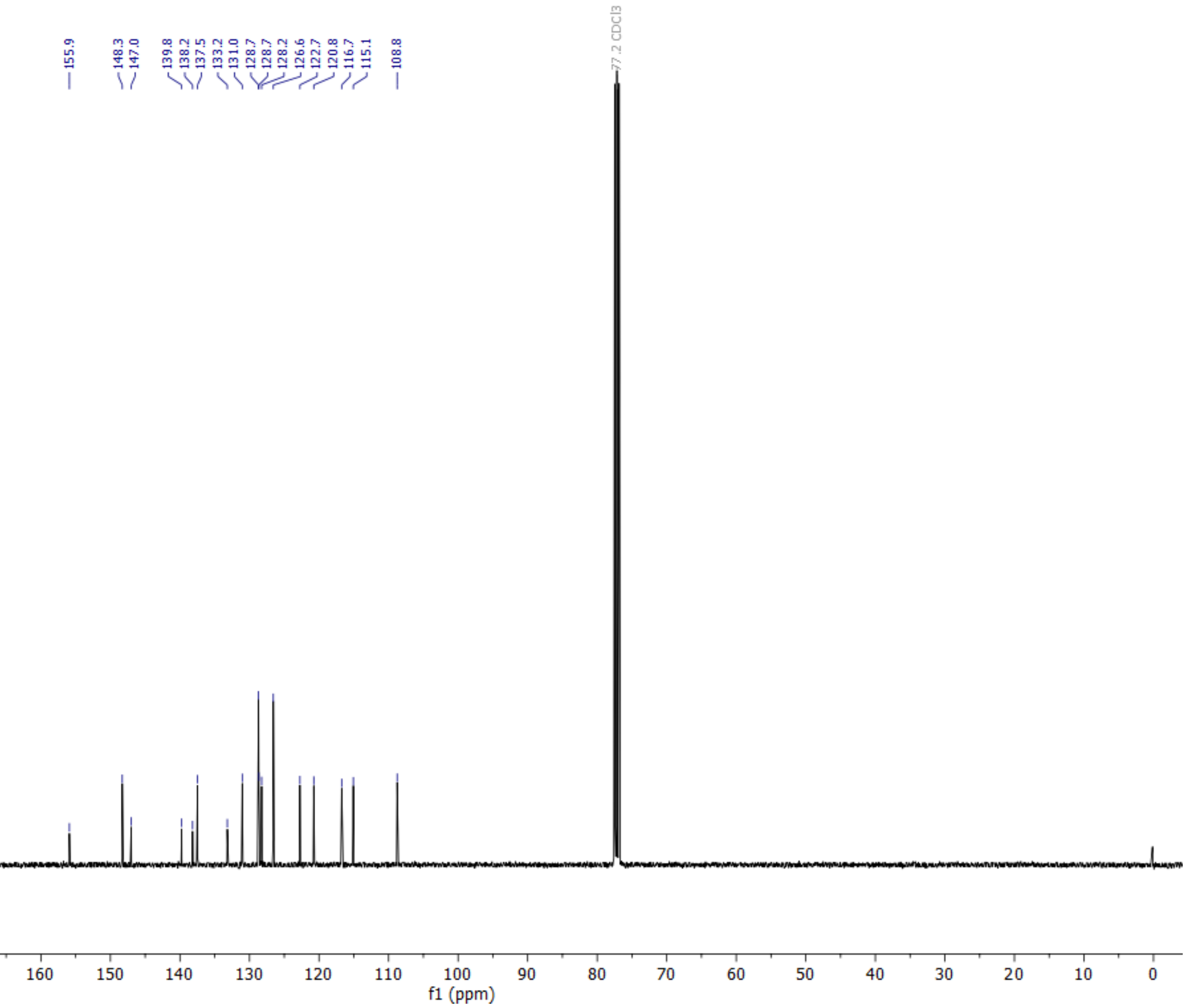

^{1}H–^{13}C HSQC-DEPT NMR (400 MHz, $CDCl_3$) of **1a**

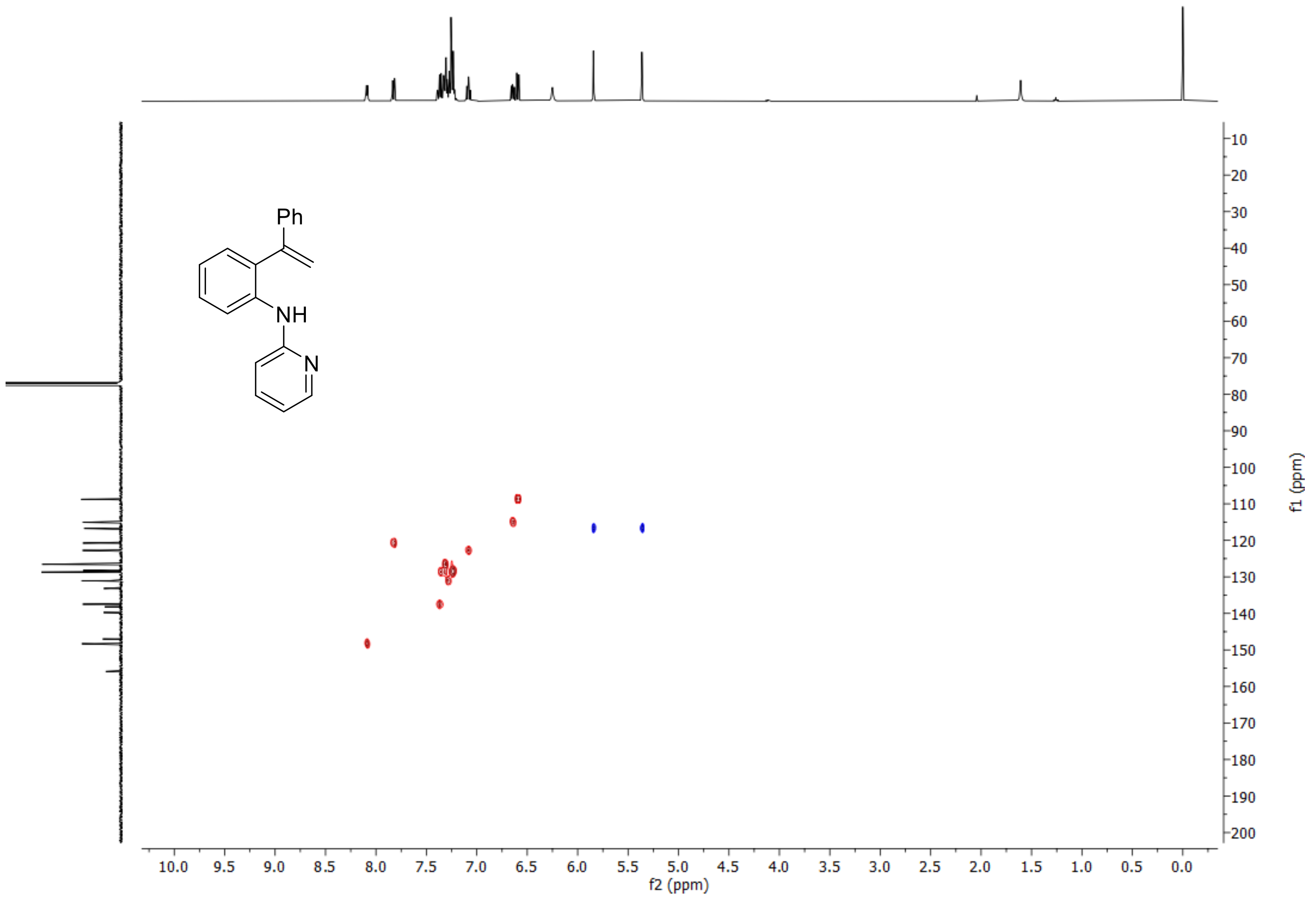

^{1}H NMR (400 MHz, $CDCl_3$) of **1b**

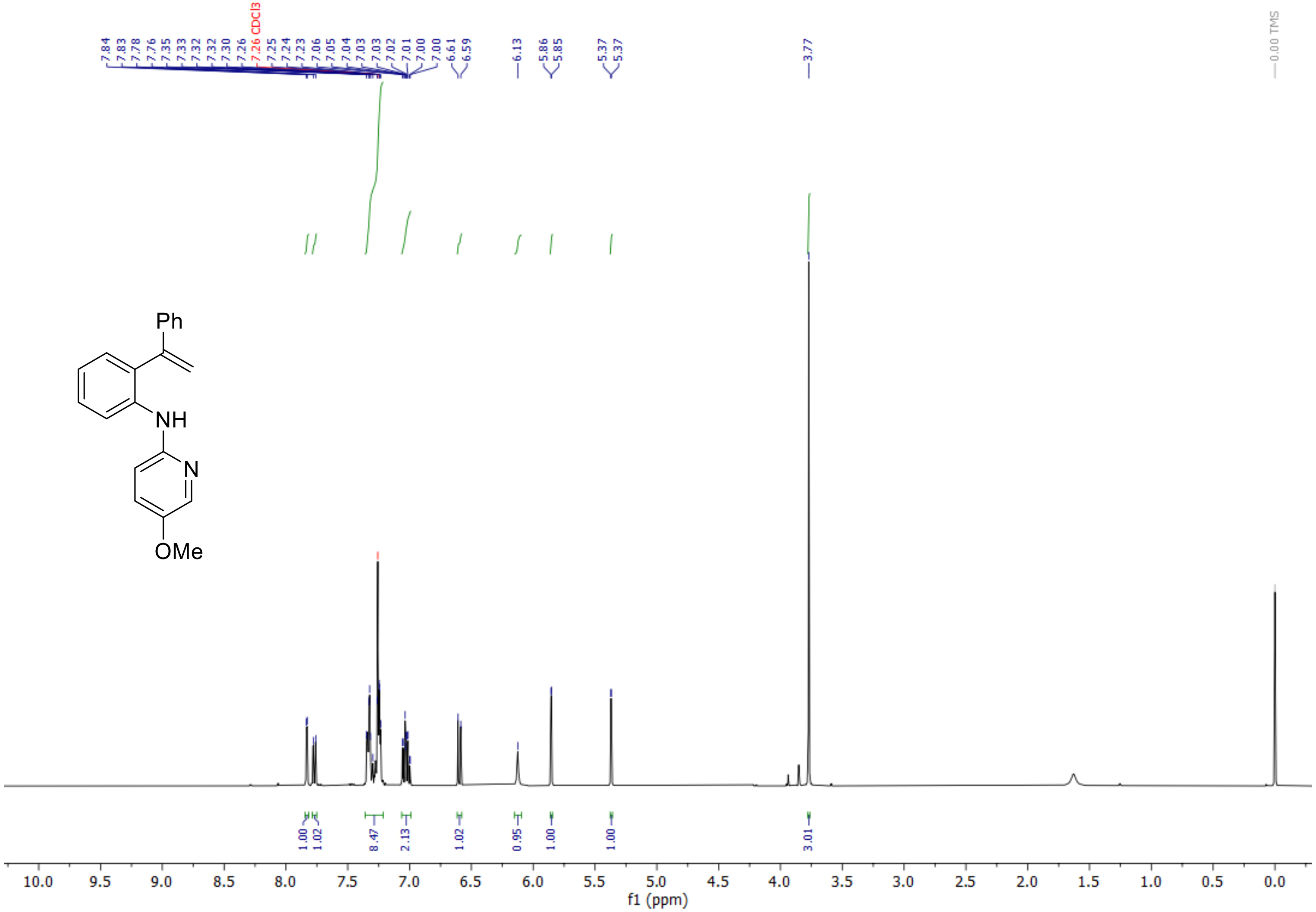

^{13}C NMR (101 MHz, $CDCl_3$) of **1b**

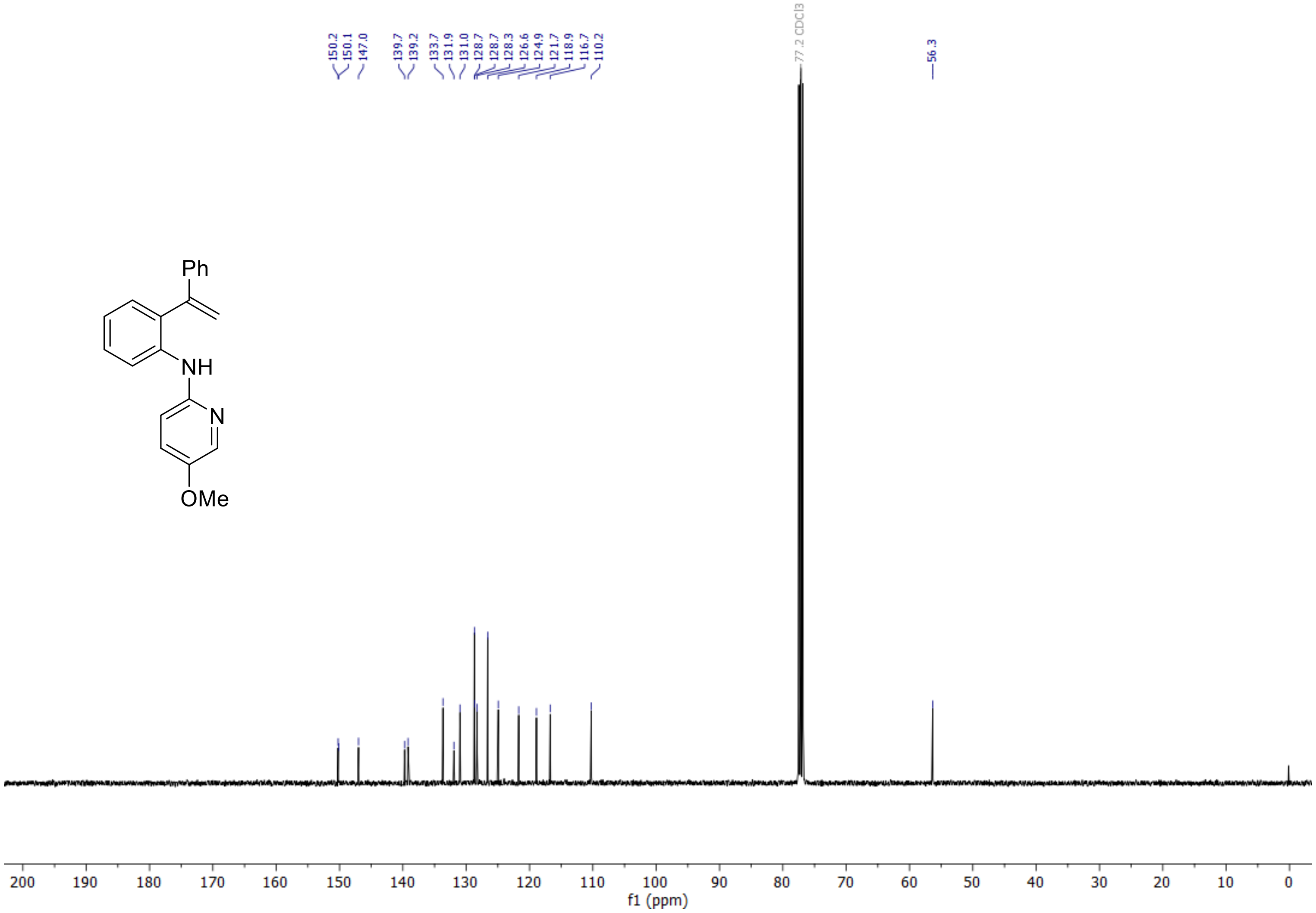

^{1}H–^{13}C HSQC-DEPT NMR (400 MHz, $CDCl_3$) of **1b**

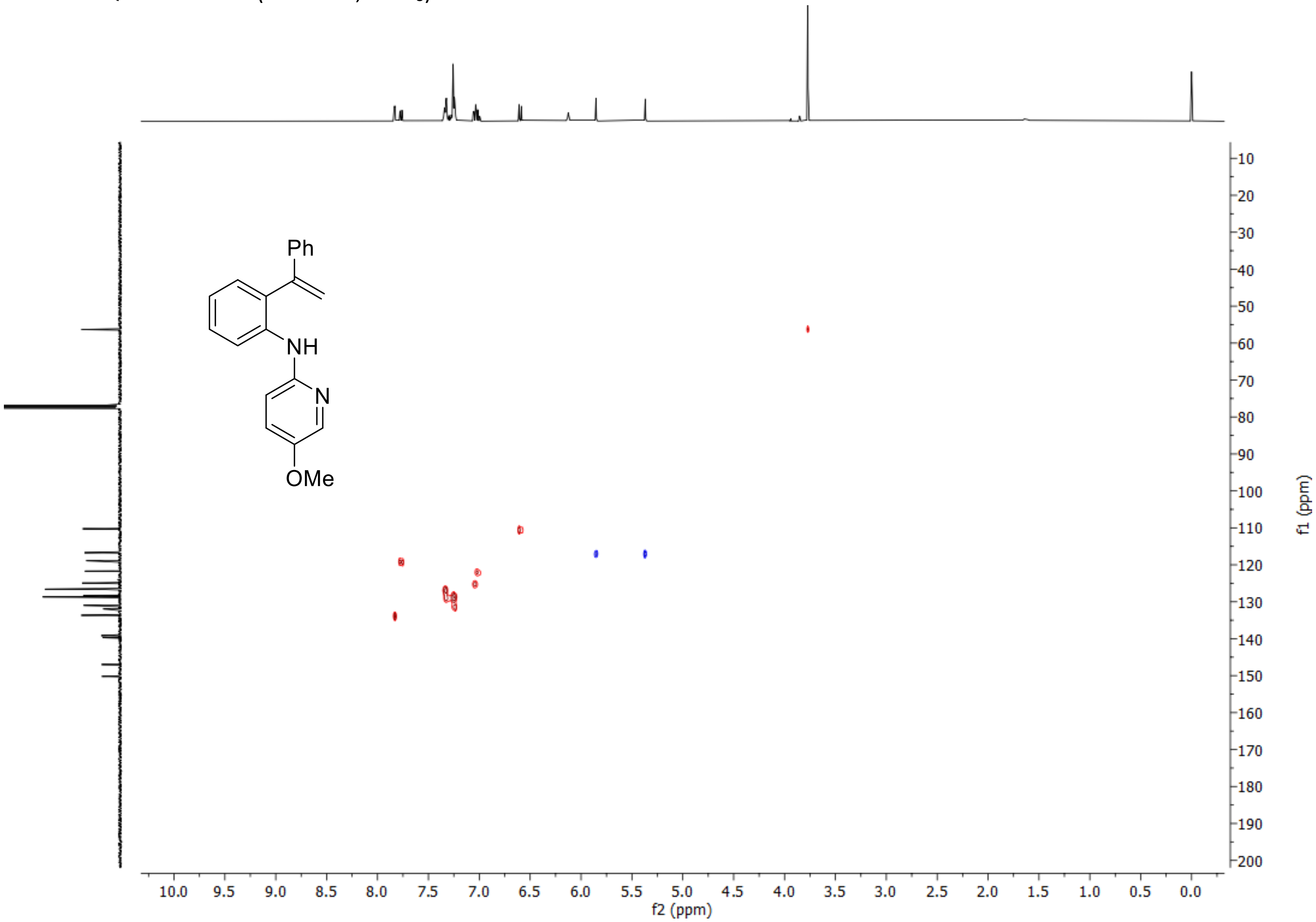

^1H NMR (400 MHz, $CDCl_3$) of **1d**

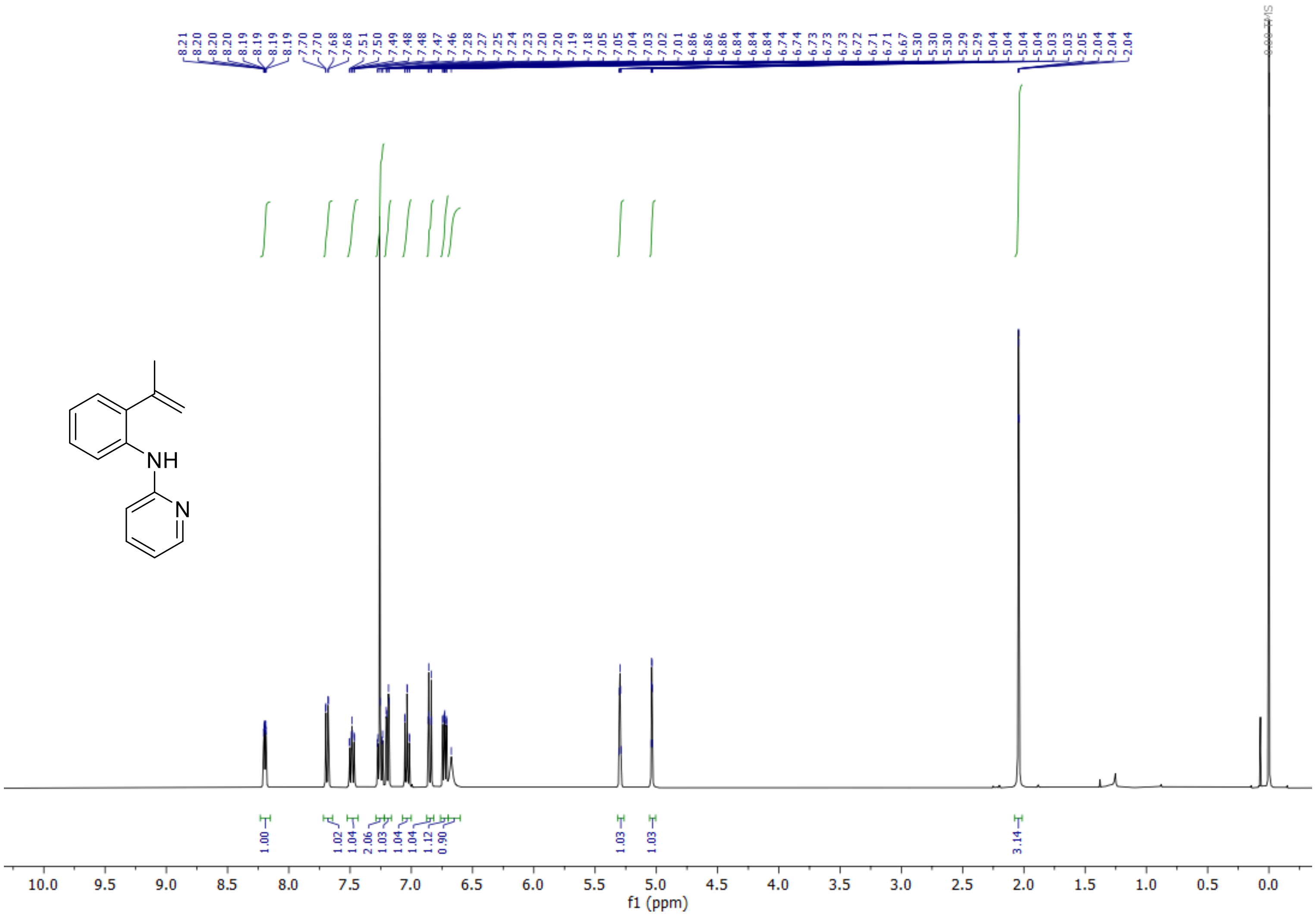

^{13}C NMR (101 MHz, $CDCl_3$) of **1d**

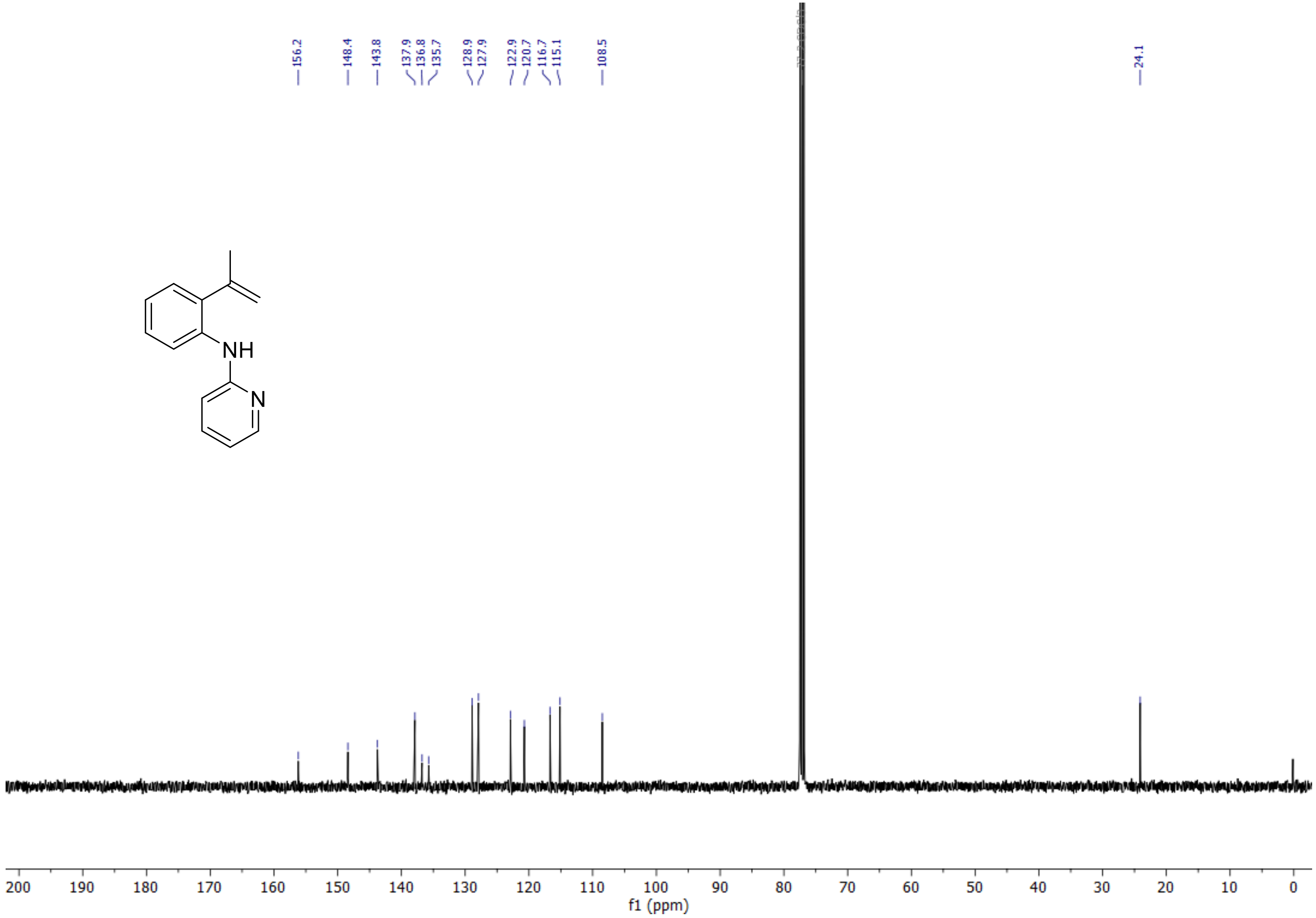

^{1}H–^{13}C HSQC-DEPT NMR (400 MHz, $CDCl_3$) of **1d**

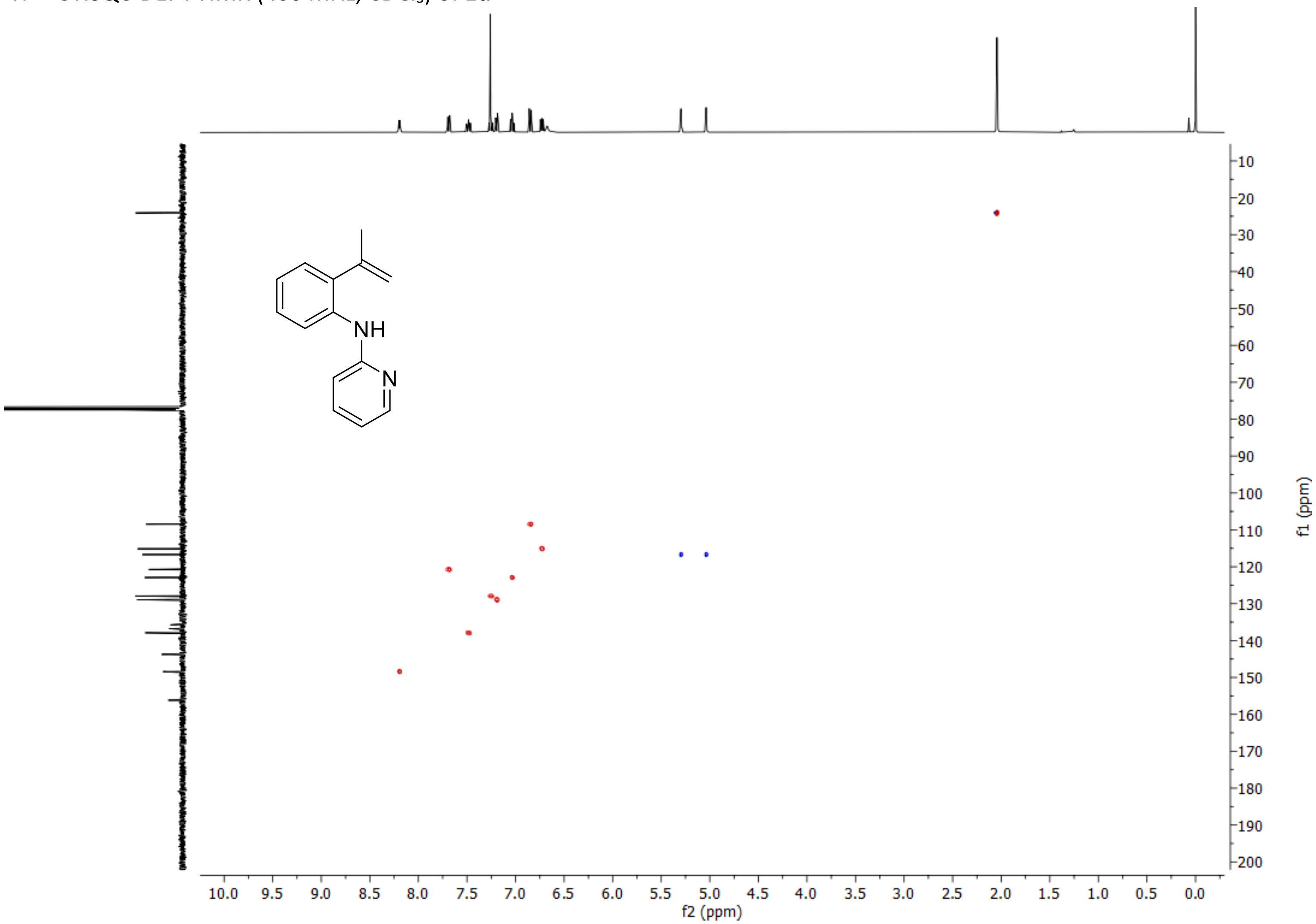

^{1}H NMR (400 MHz, $CDCl_3$) of **1f**

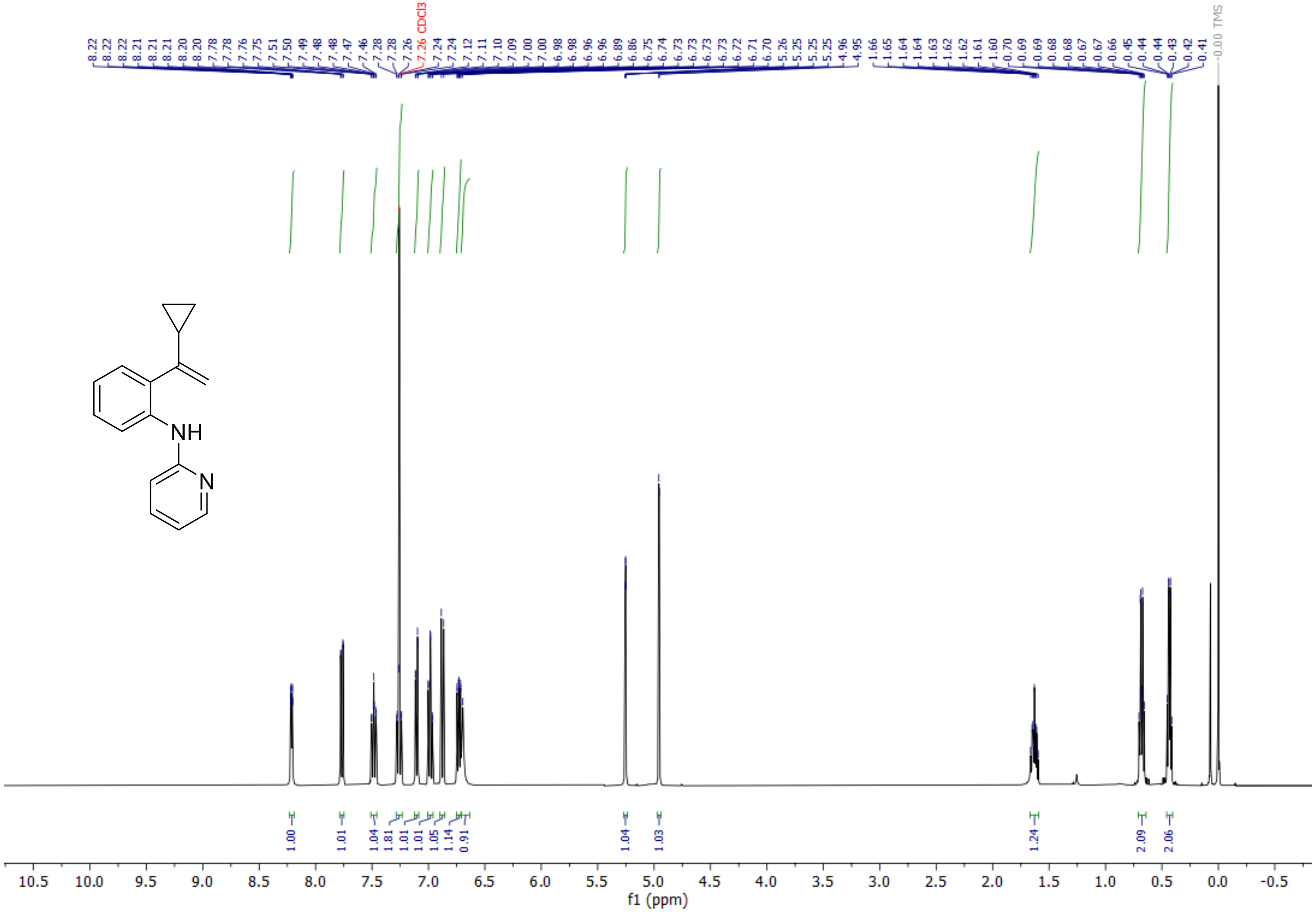

^{13}C NMR (101 MHz, $CDCl_3$) of **1f**

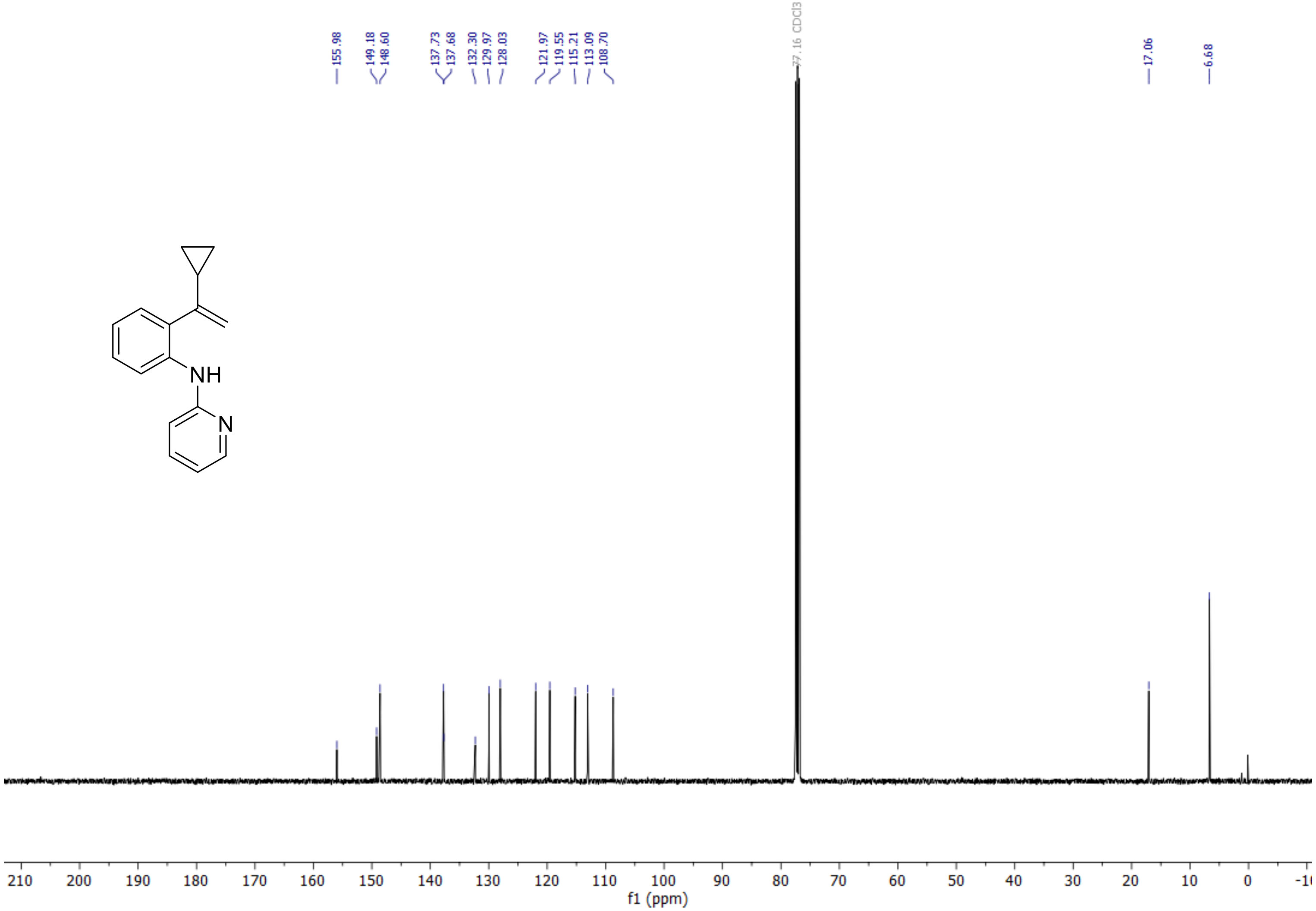

^{1}H–^{13}C HSQC-DEPT NMR (400 MHz, $CDCl_3$) of **1f**

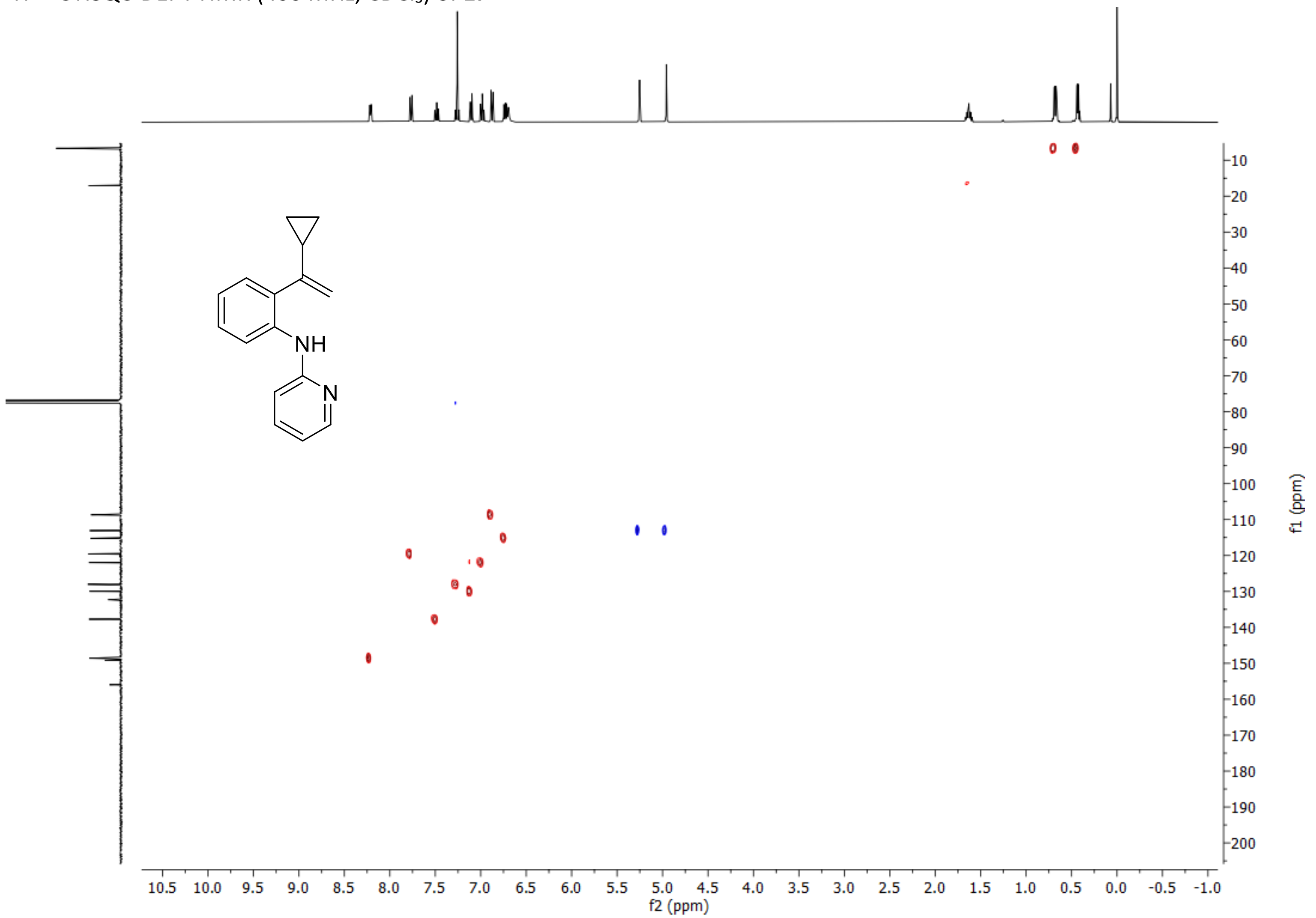

^{1}H NMR (400 MHz, $CDCl_3$) of **1g**

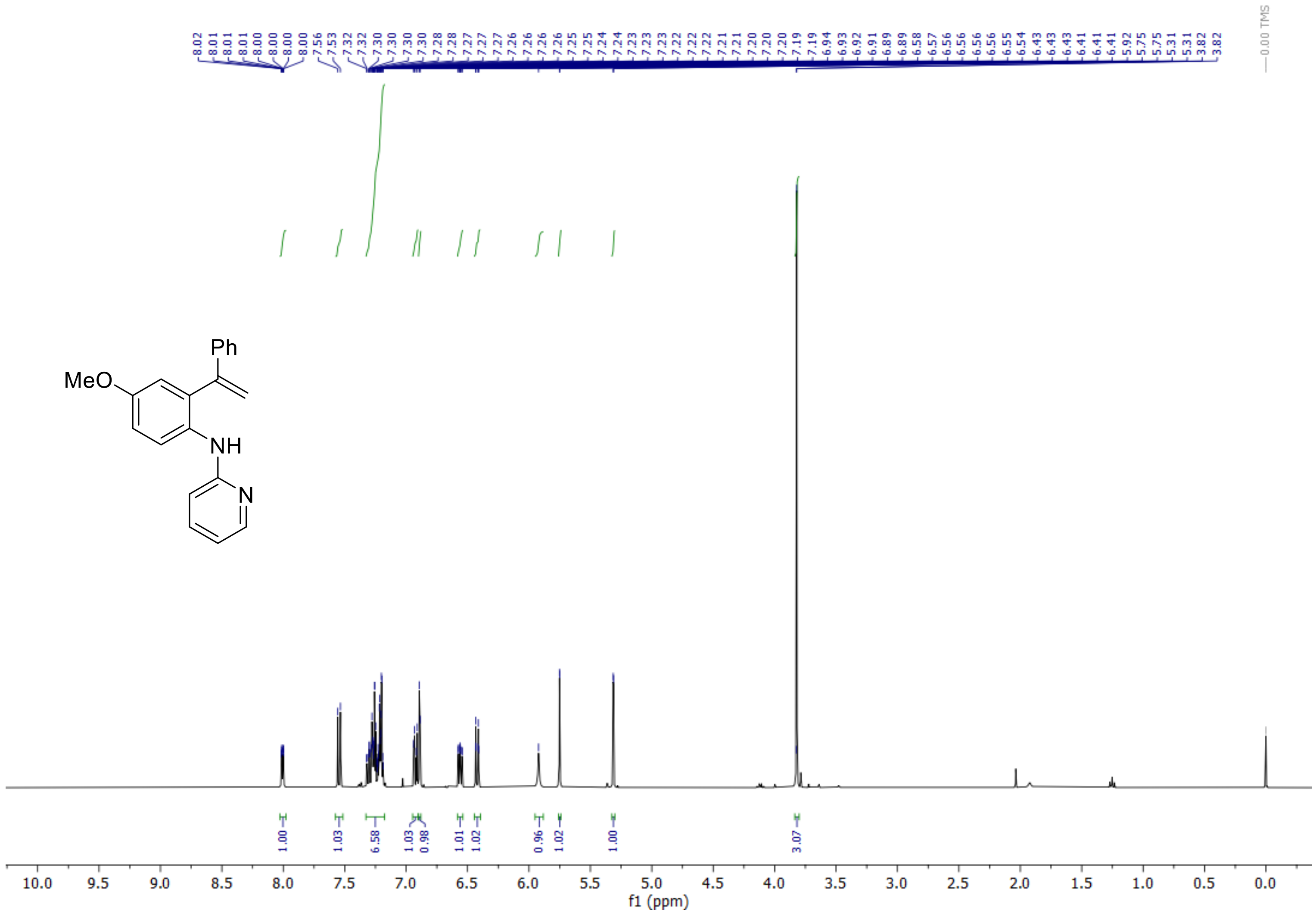

^{13}C NMR (101 MHz, $CDCl_3$) of **1g**

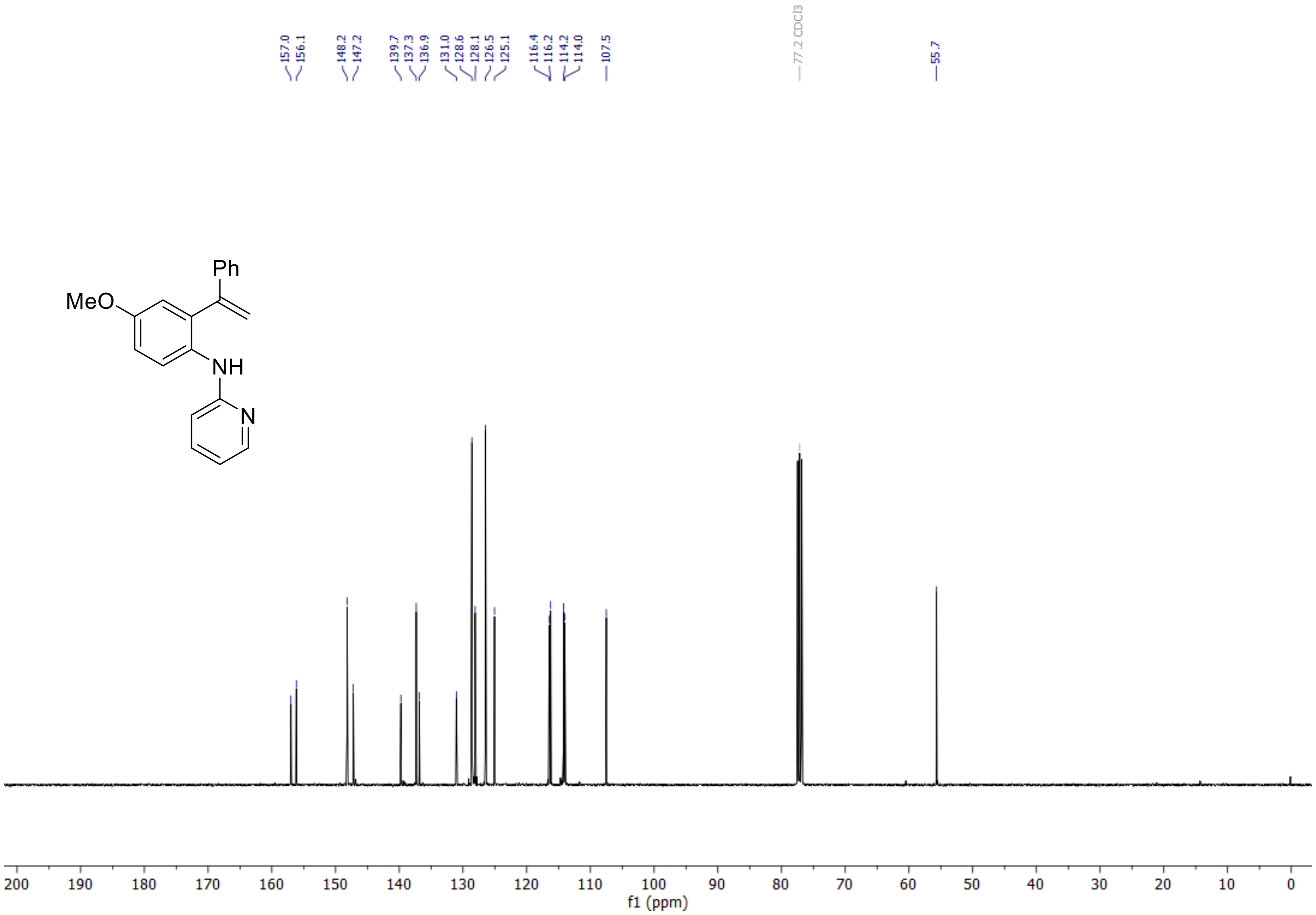

^{1}H–^{13}C HSQC-DEPT NMR (400 MHz, $CDCl_3$) of **1g**

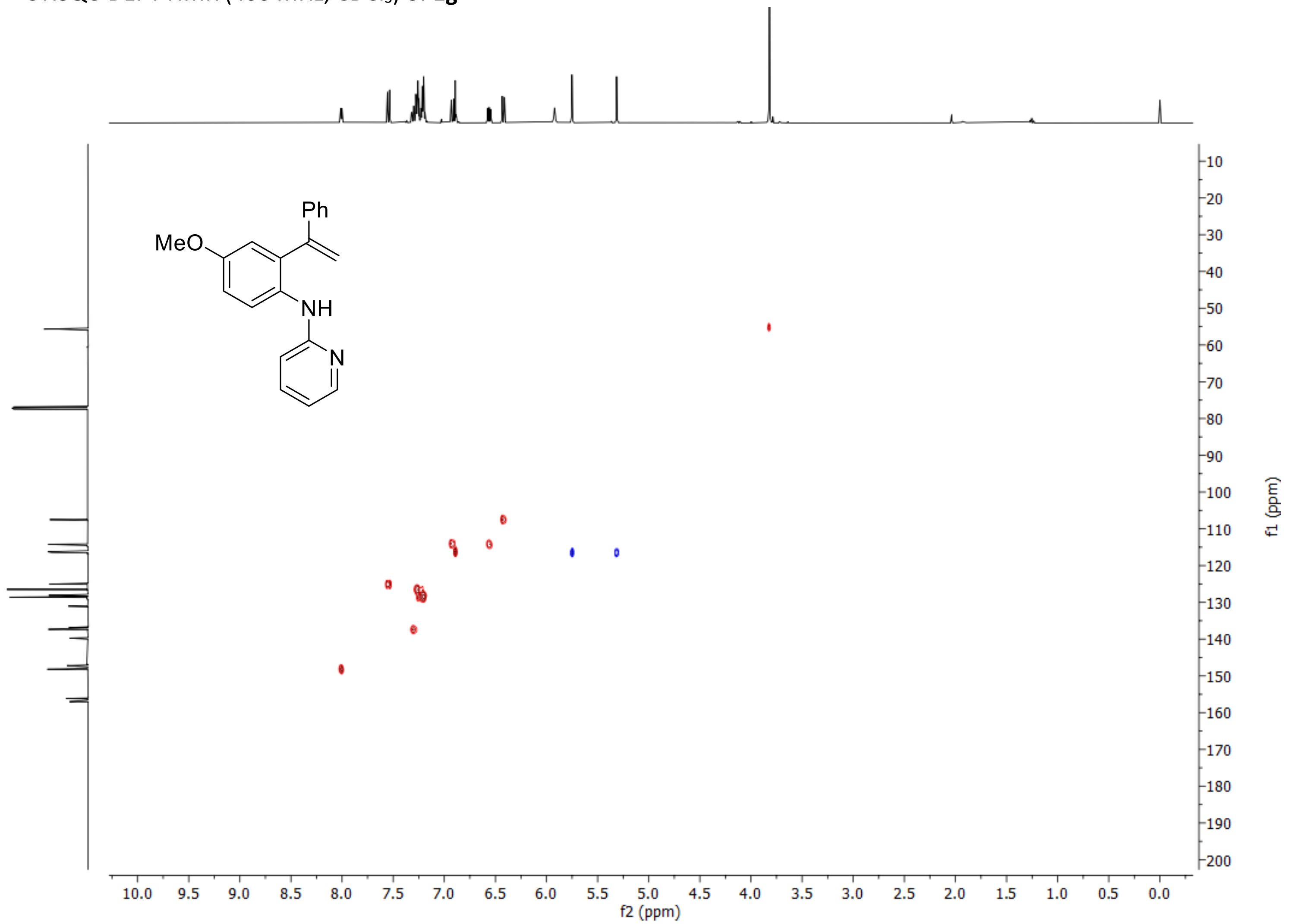

^{1}H NMR (400 MHz, $CDCl_3$) of **1h**

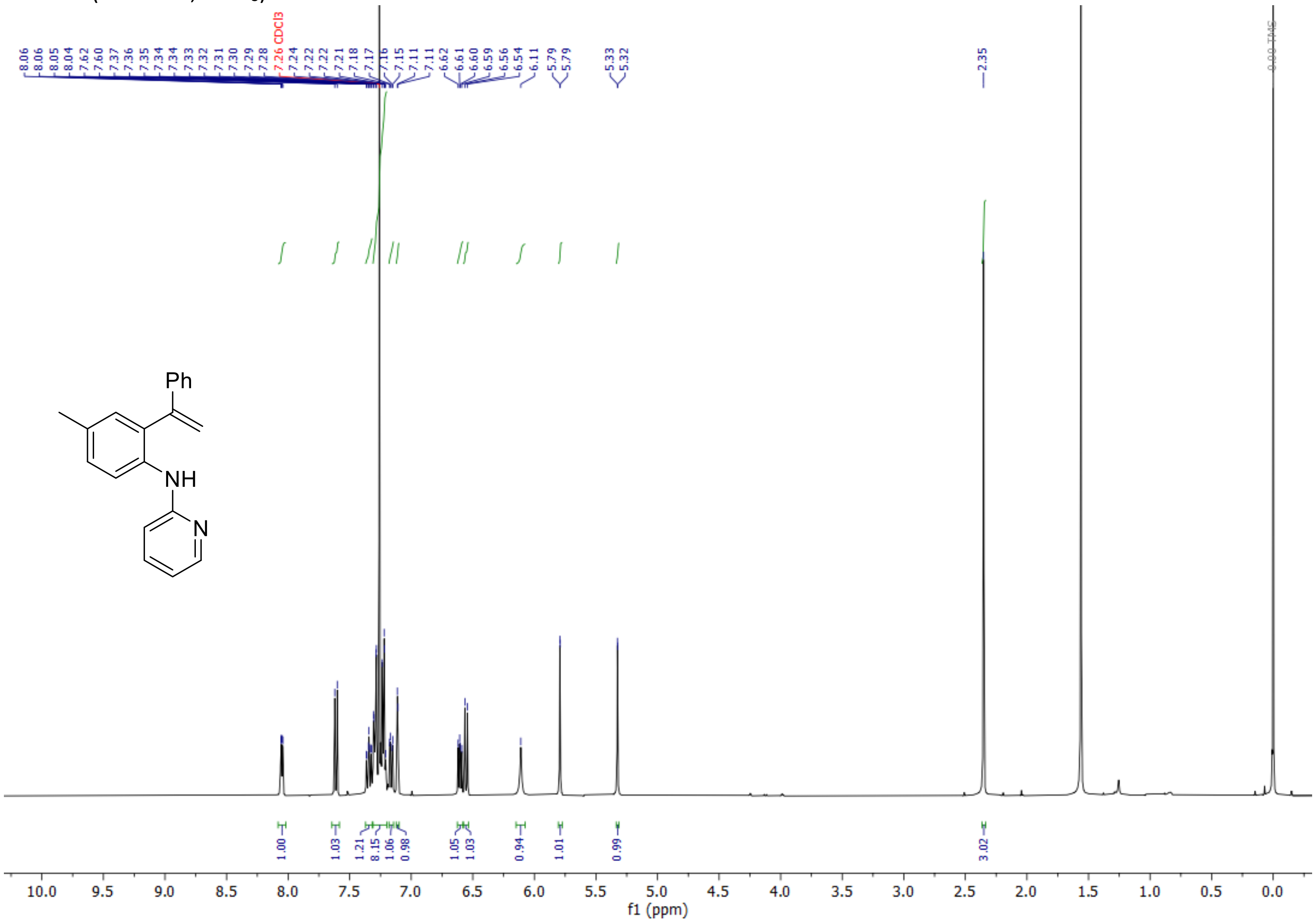

^{13}C NMR (101 MHz, $CDCl_3$) of **1h**

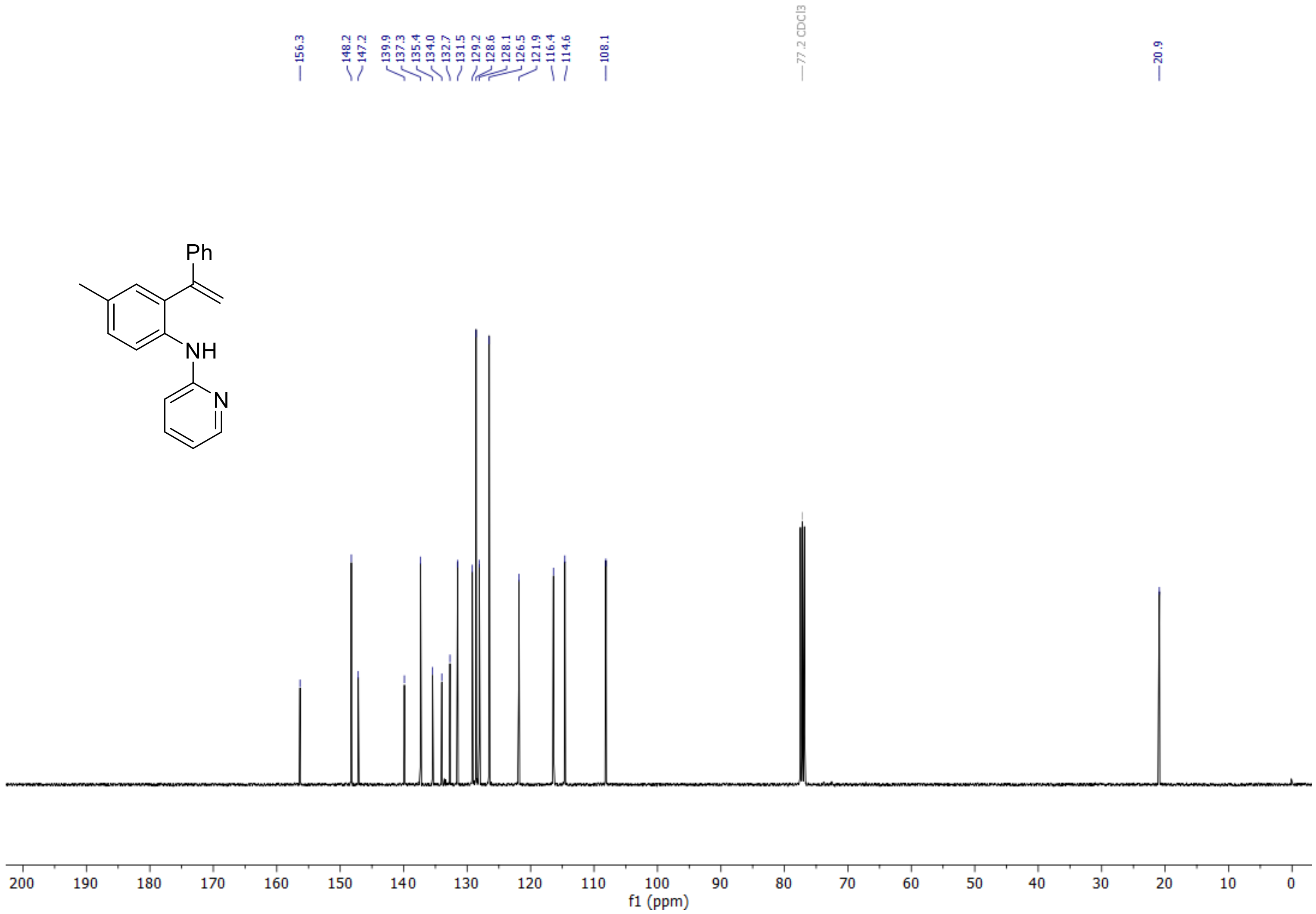

^1H–^{13}C HSQC-DEPT NMR (400 MHz, $CDCl_3$) of **1h**

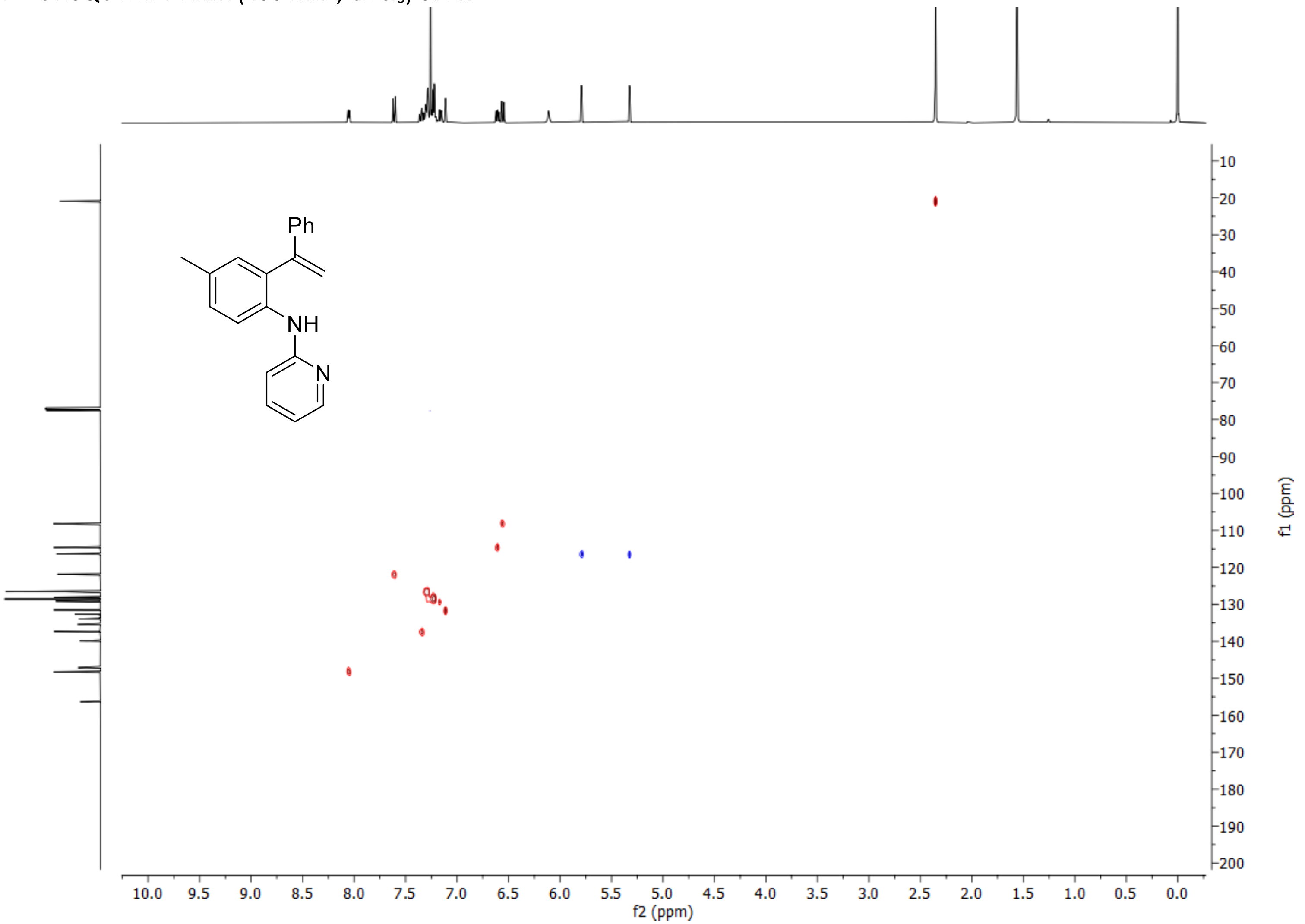

^{1}H NMR (400 MHz, $CDCl_3$) of **1i**

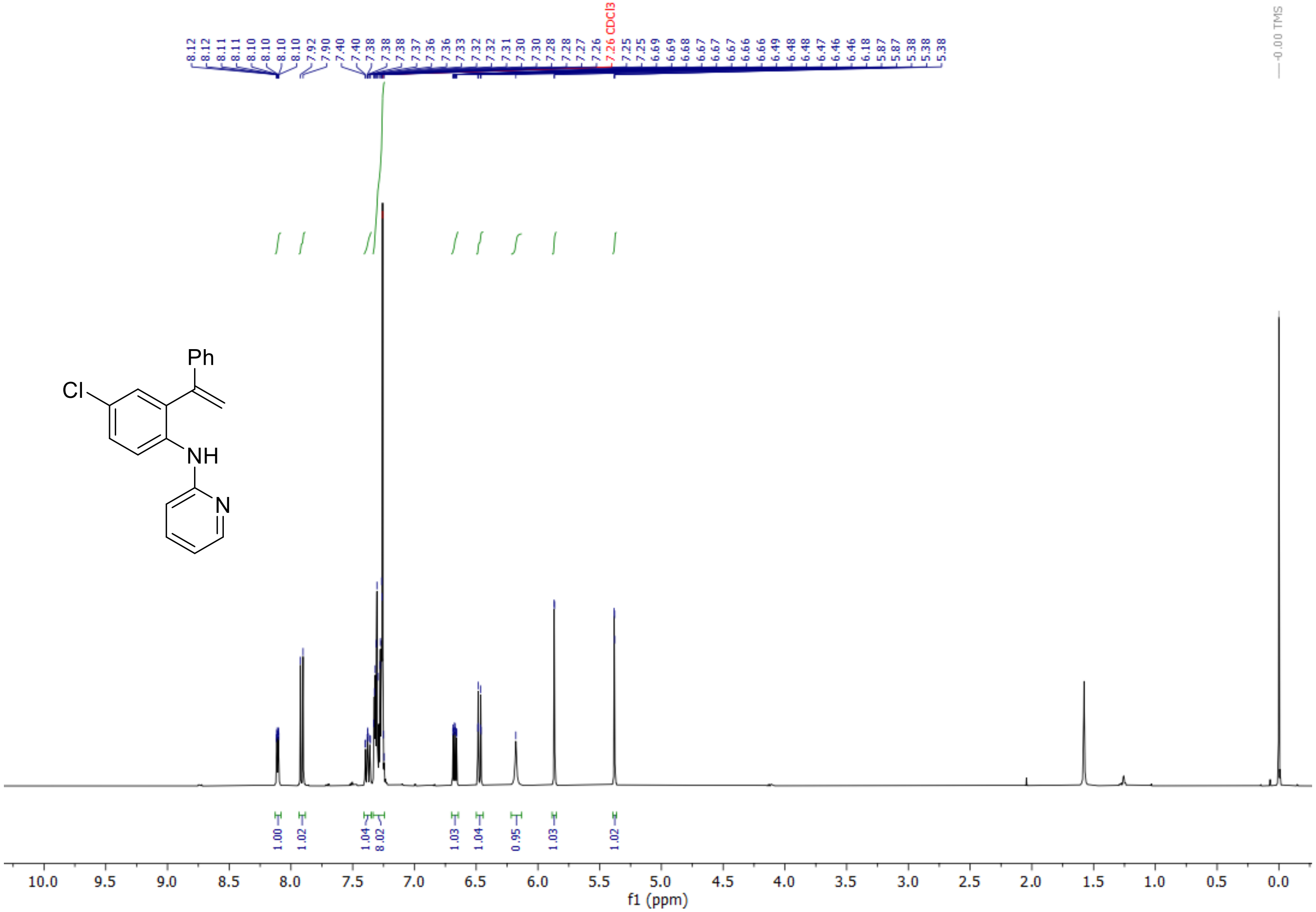

^{13}C NMR (101 MHz, $CDCl_3$) of **1i**

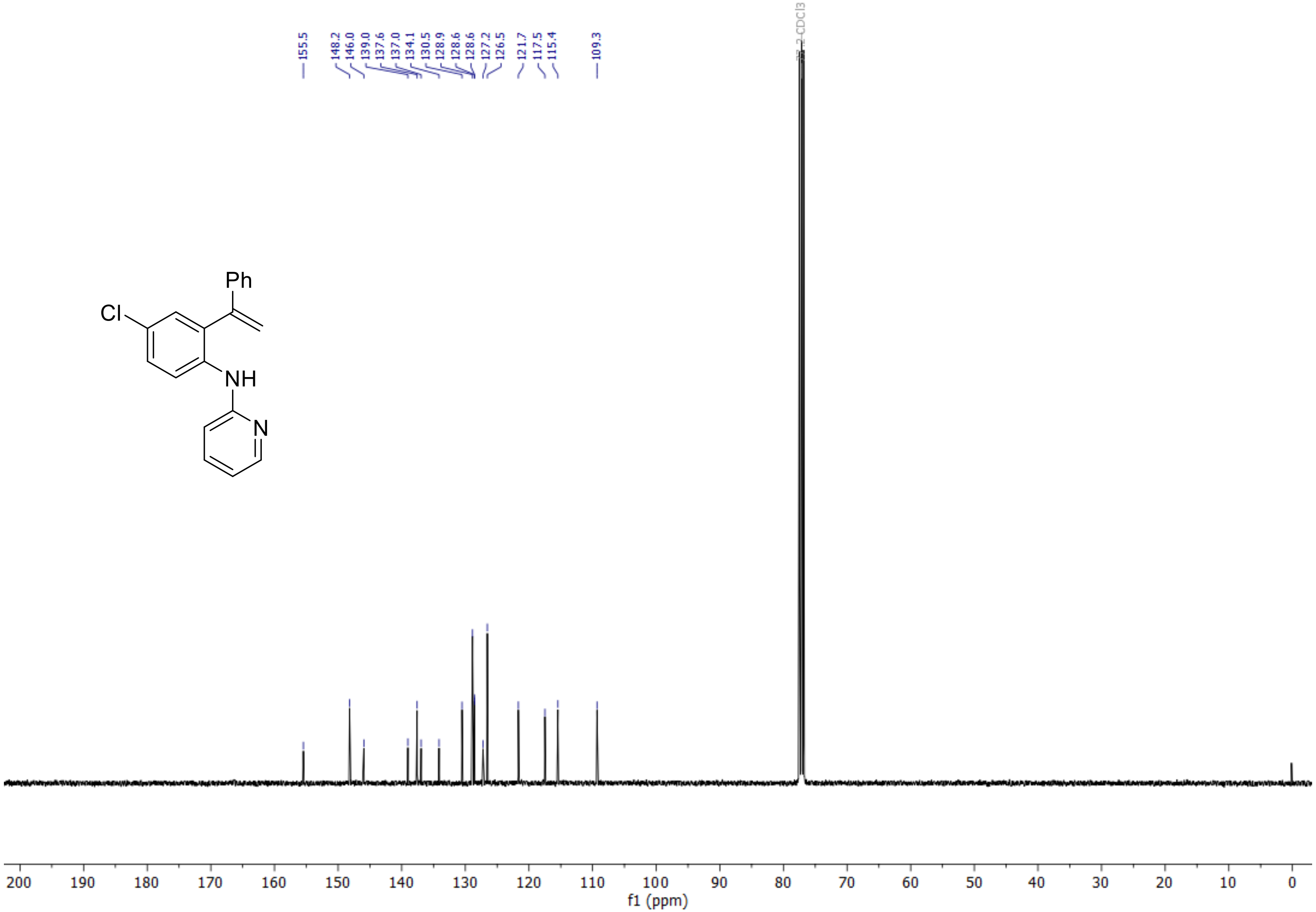

^{1}H–^{13}C HSQC-DEPT NMR (400 MHz, $CDCl_3$) of **1i**

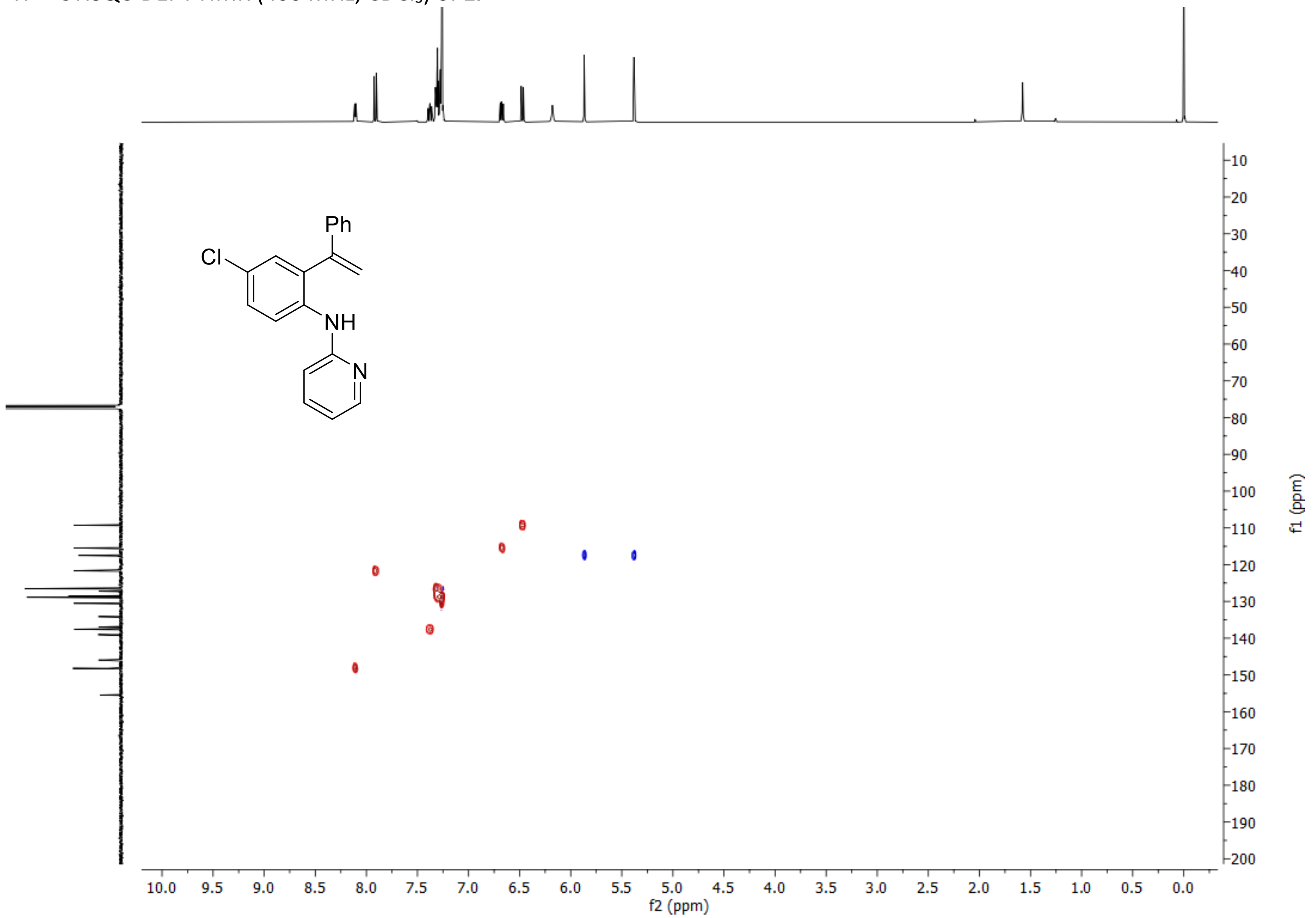

^{1}H NMR (400 MHz, $CDCl_3$) of **1j**

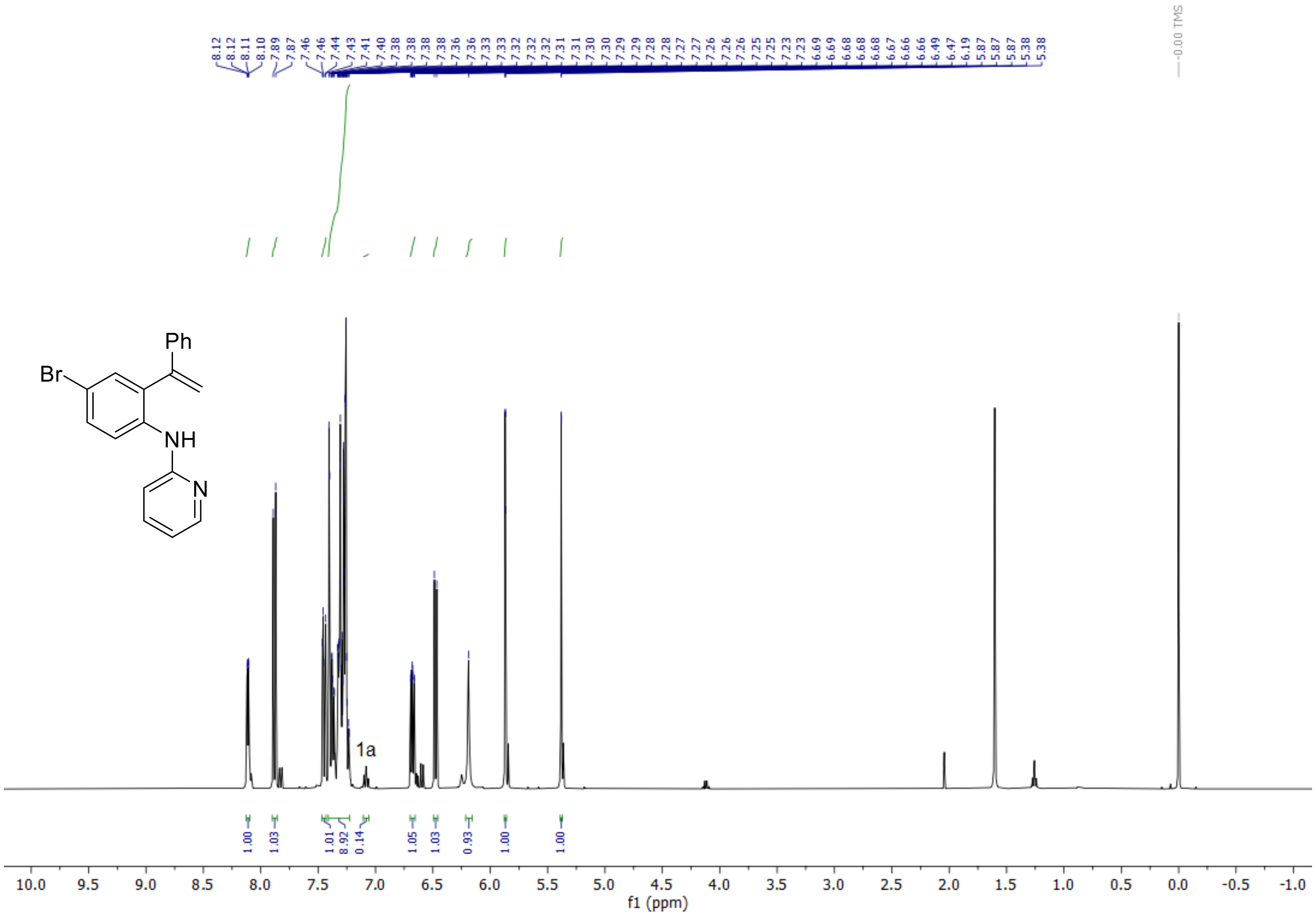

^{13}C NMR (101 MHz, $CDCl_3$) of **1j**

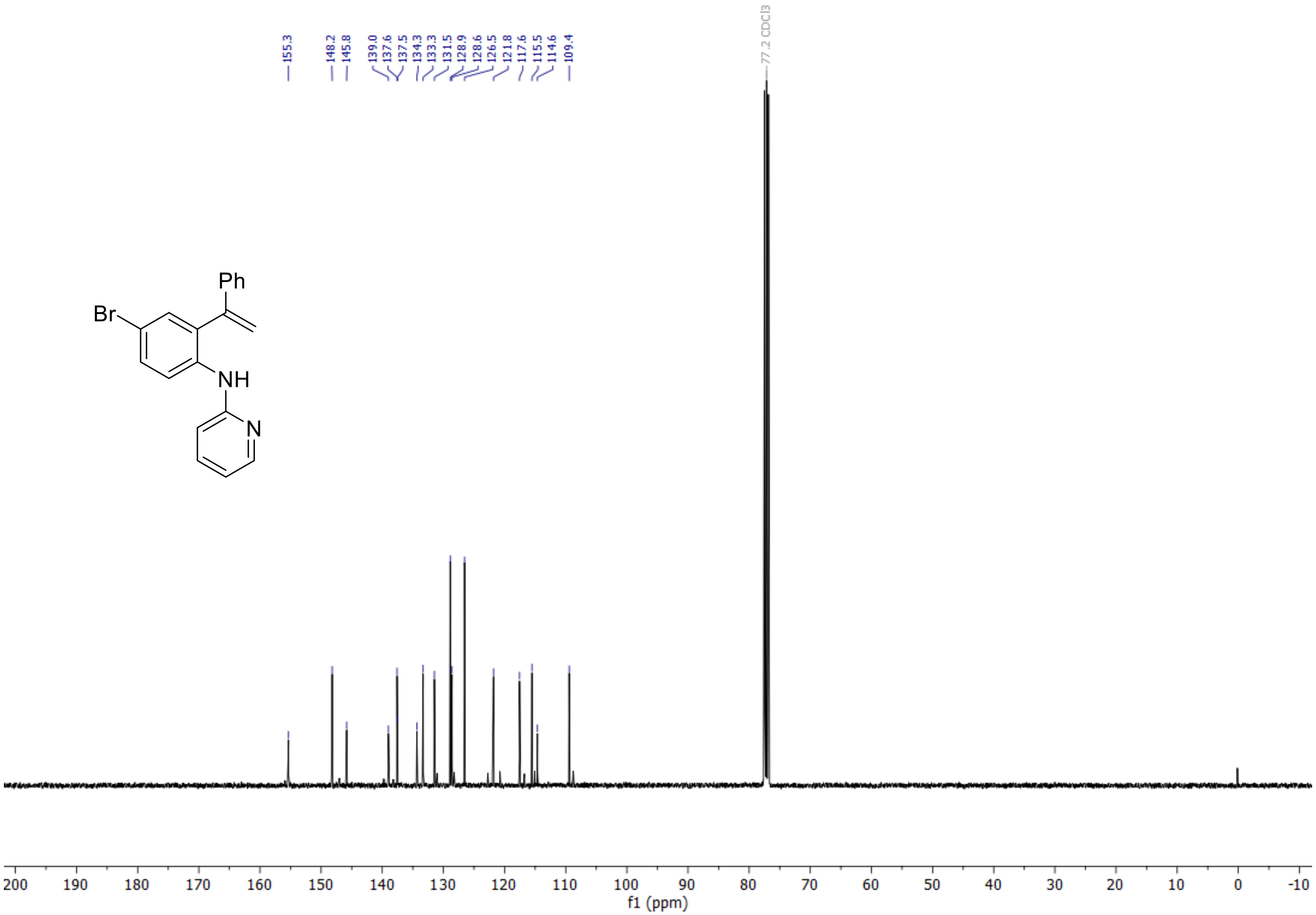

^{1}H–^{13}C HSQC-DEPT NMR (400 MHz, $CDCl_3$) of **1j**

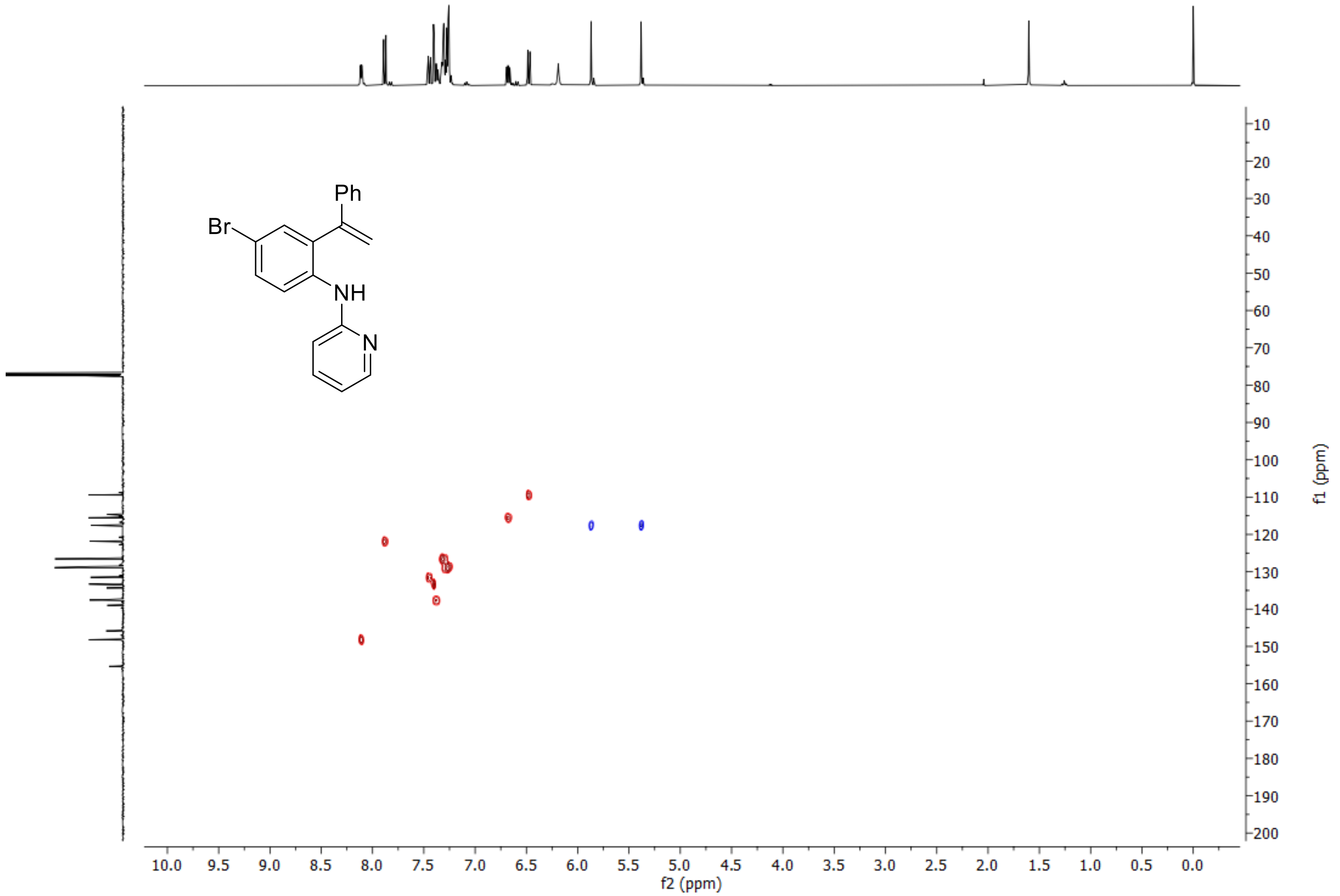

^1H NMR (400 MHz, $CDCl_3$) of **1k**

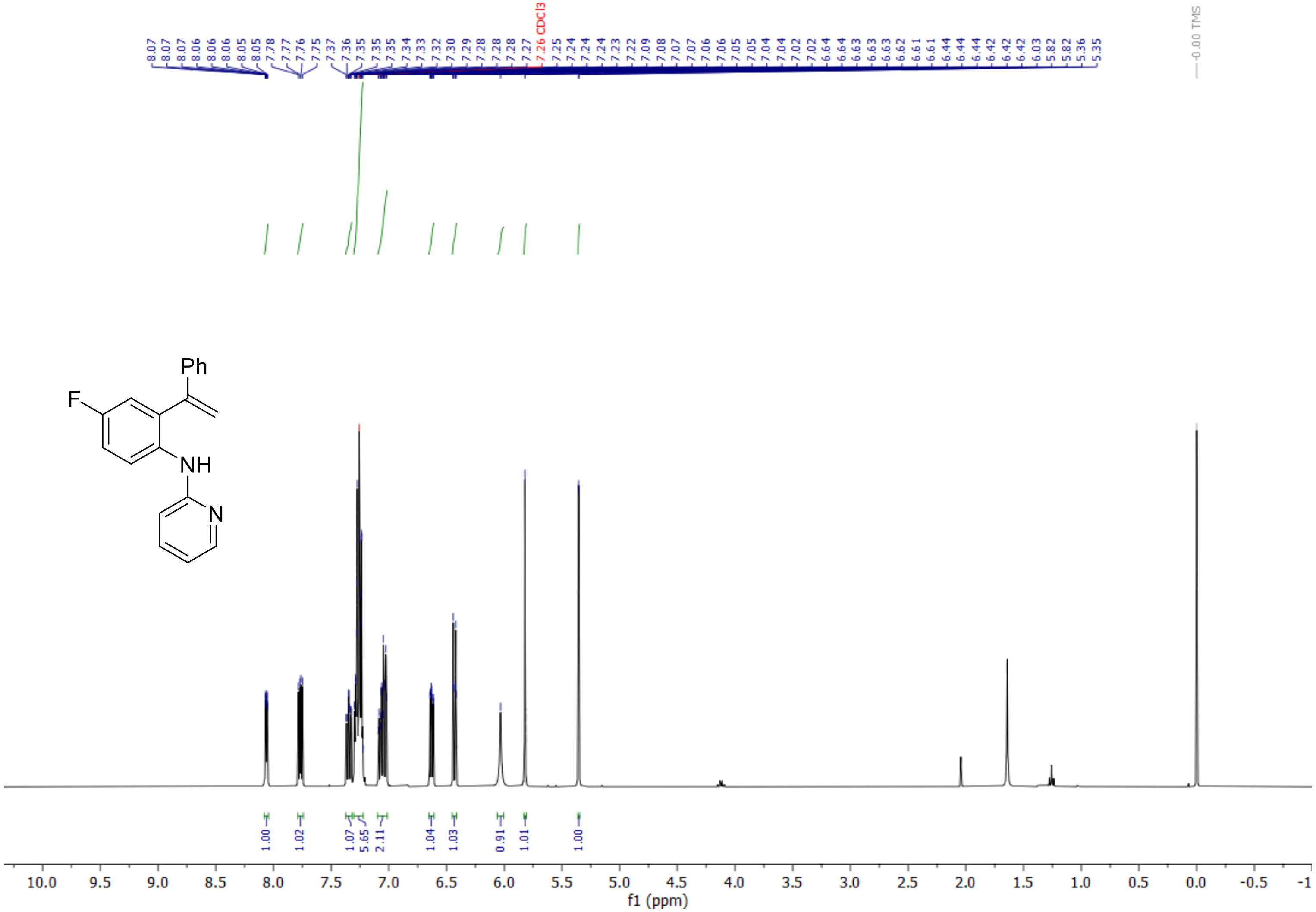

^{13}C NMR (101 MHz, $CDCl_3$) of **1k**

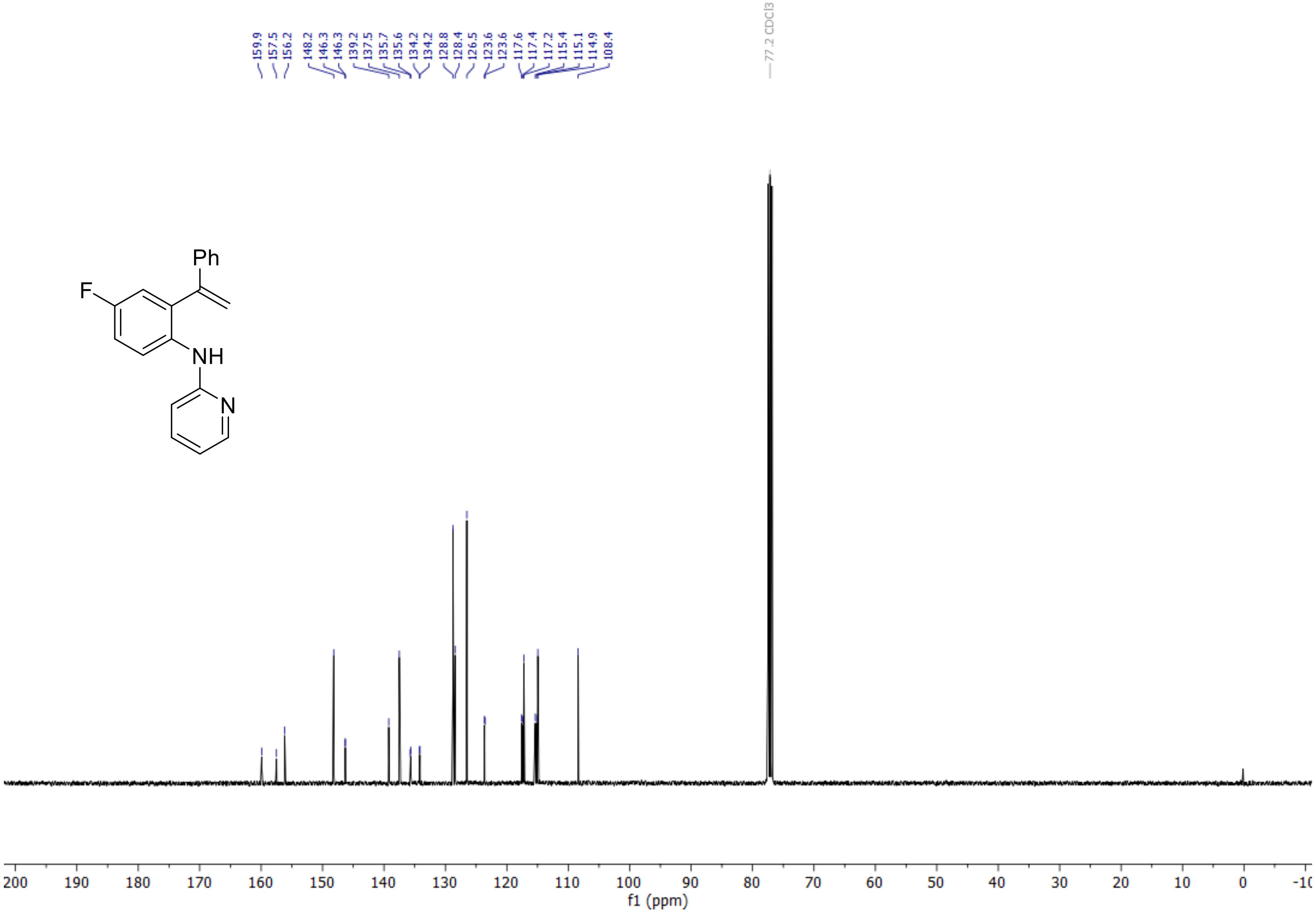

^{1}H–^{13}C HSQC-DEPT NMR (400 MHz, $CDCl_3$) of **1k**

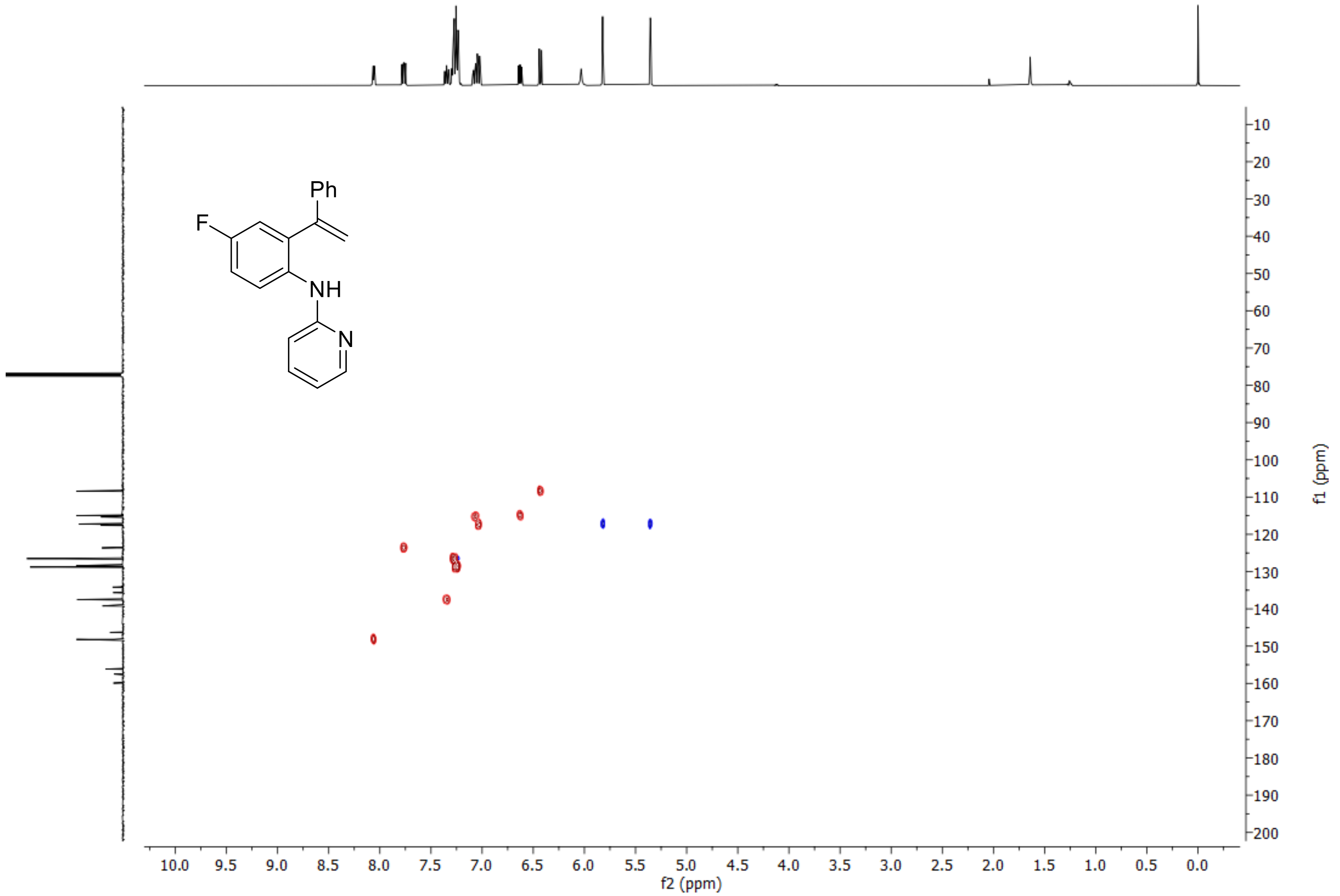

^{19}F NMR (376 MHz, $CDCl_3$) of **1k**

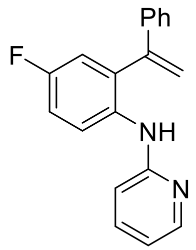

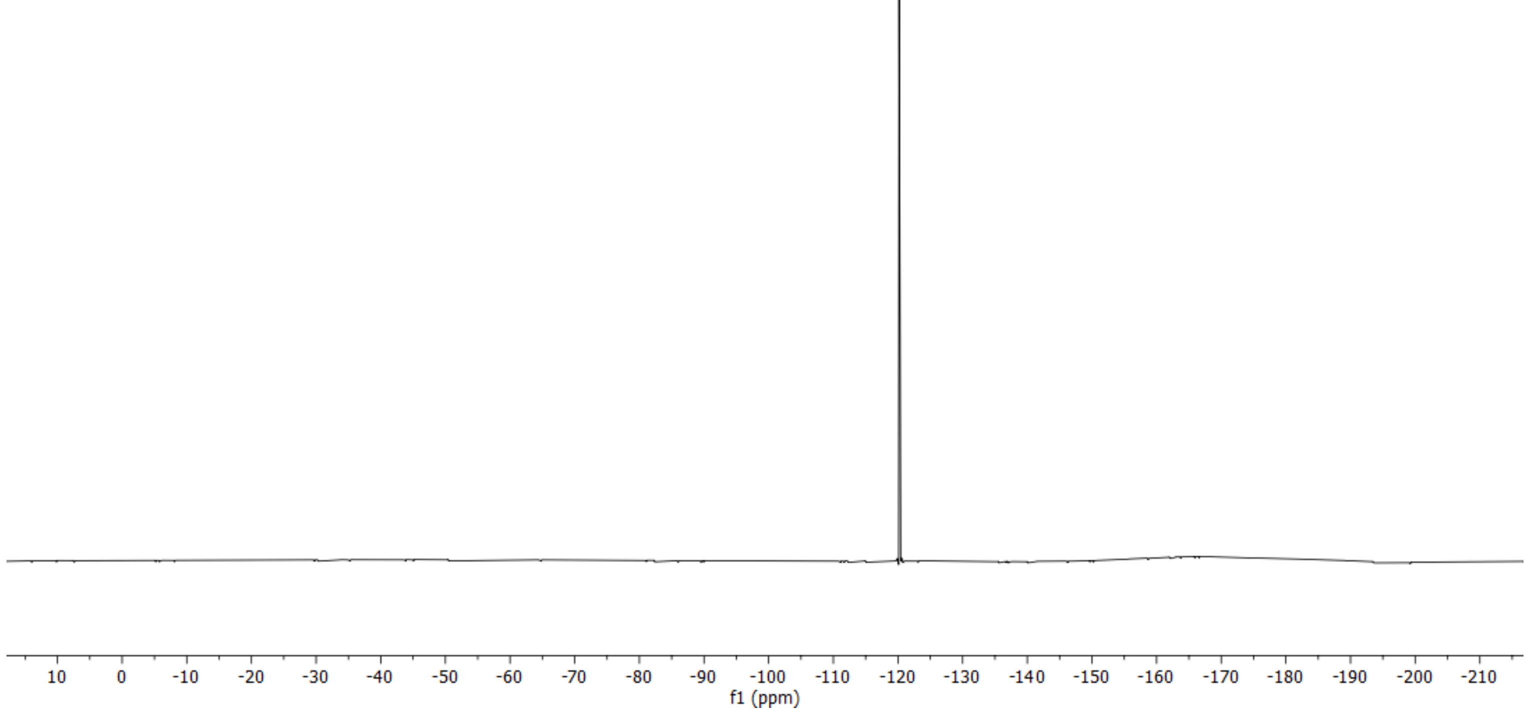

^1H NMR (400 MHz, $CDCl_3$) of **1l**

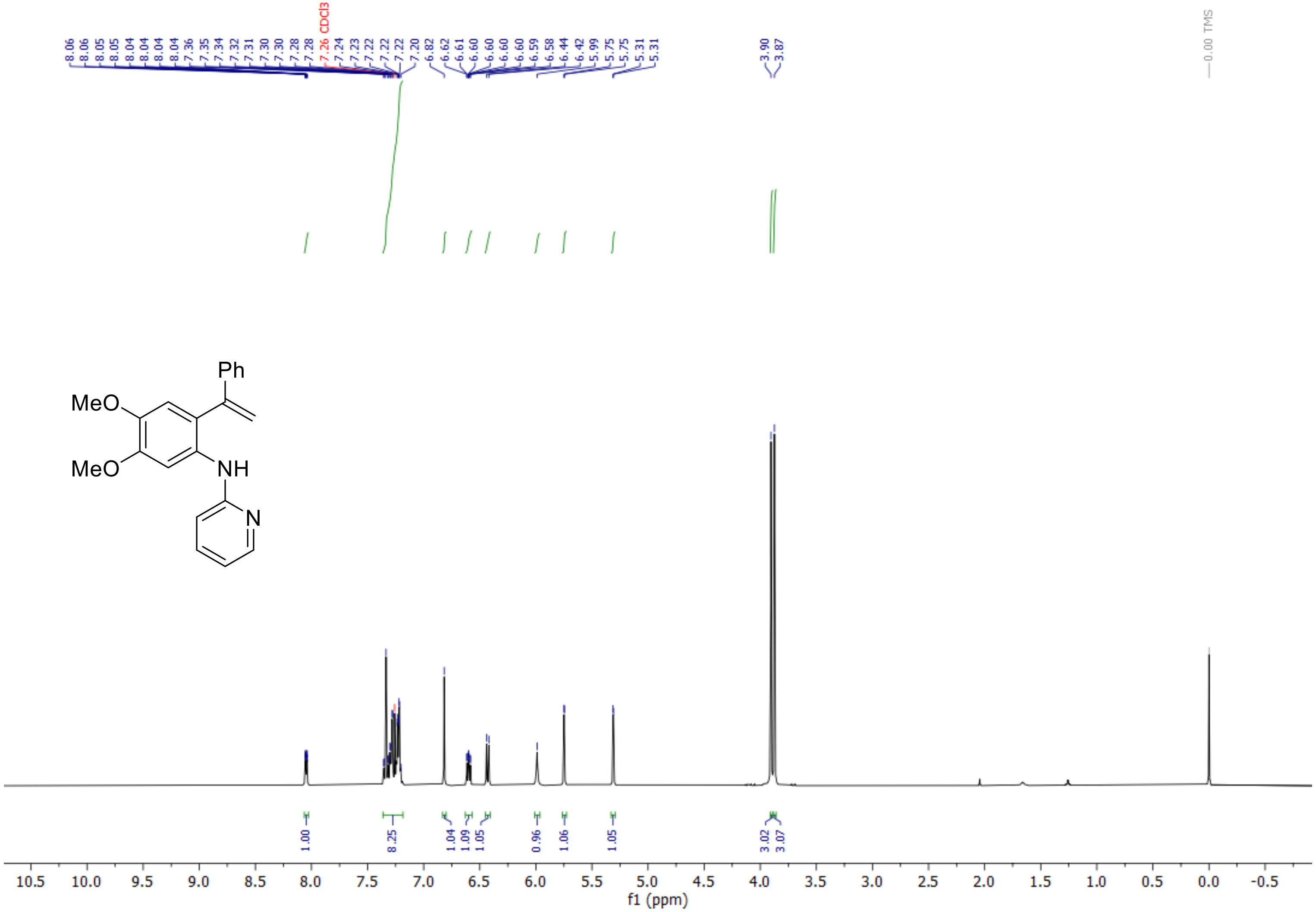

^{13}C NMR (101 MHz, $CDCl_3$) of **1l**

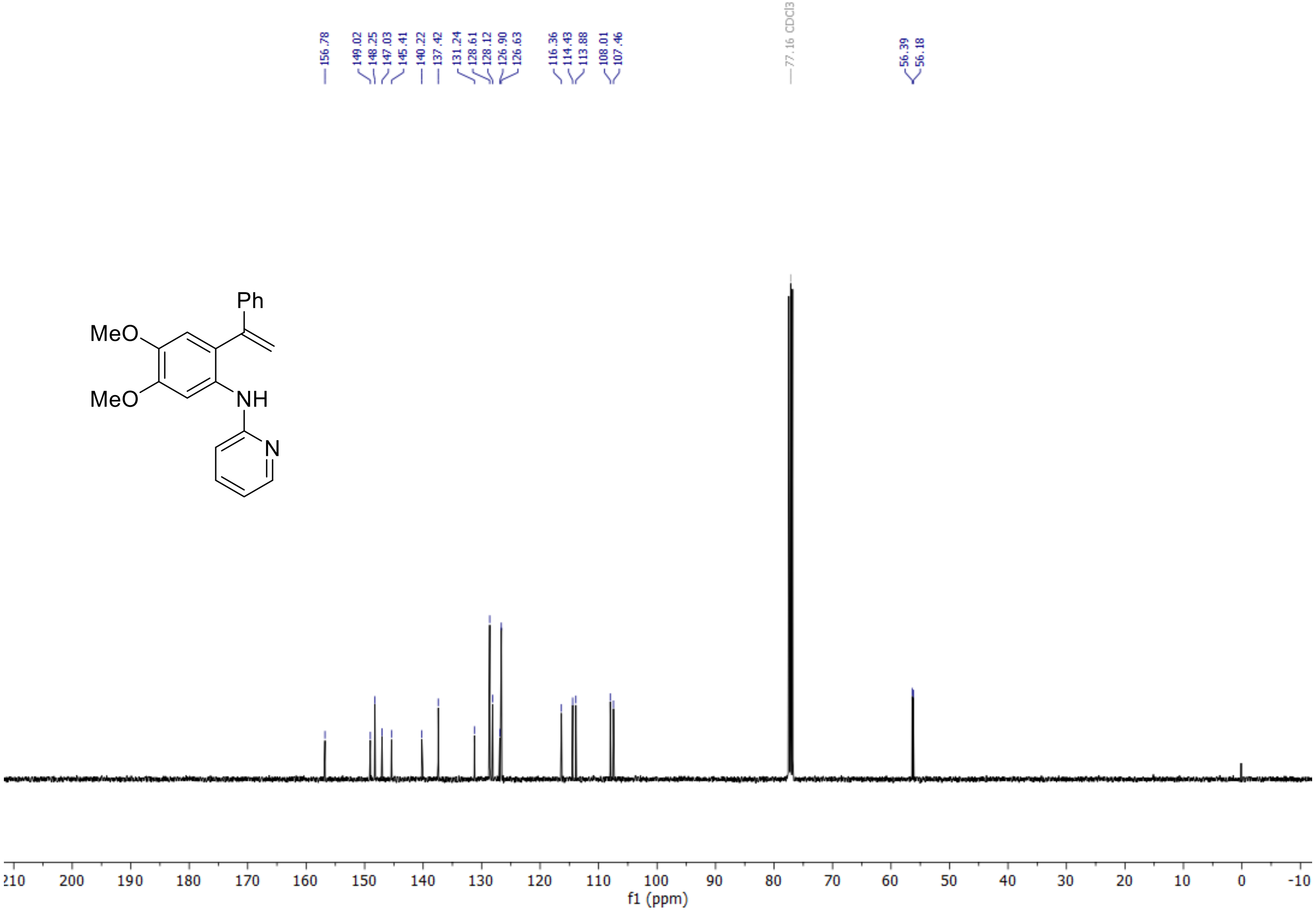

^{1}H–^{13}C HSQC-DEPT NMR (400 MHz, $CDCl_3$) of **1l**

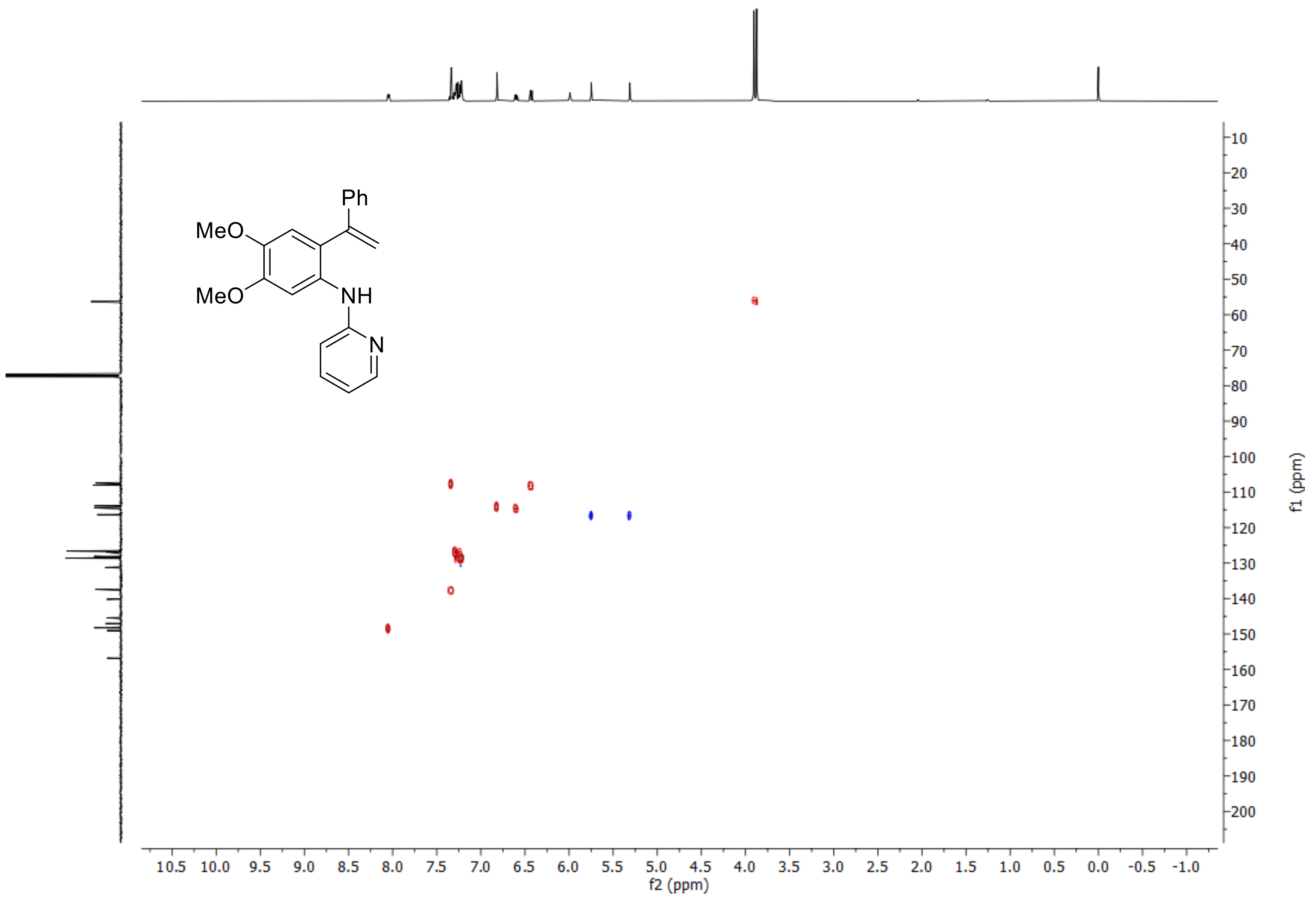

^{1}H NMR (400 MHz, $CDCl_3$) of **1m**

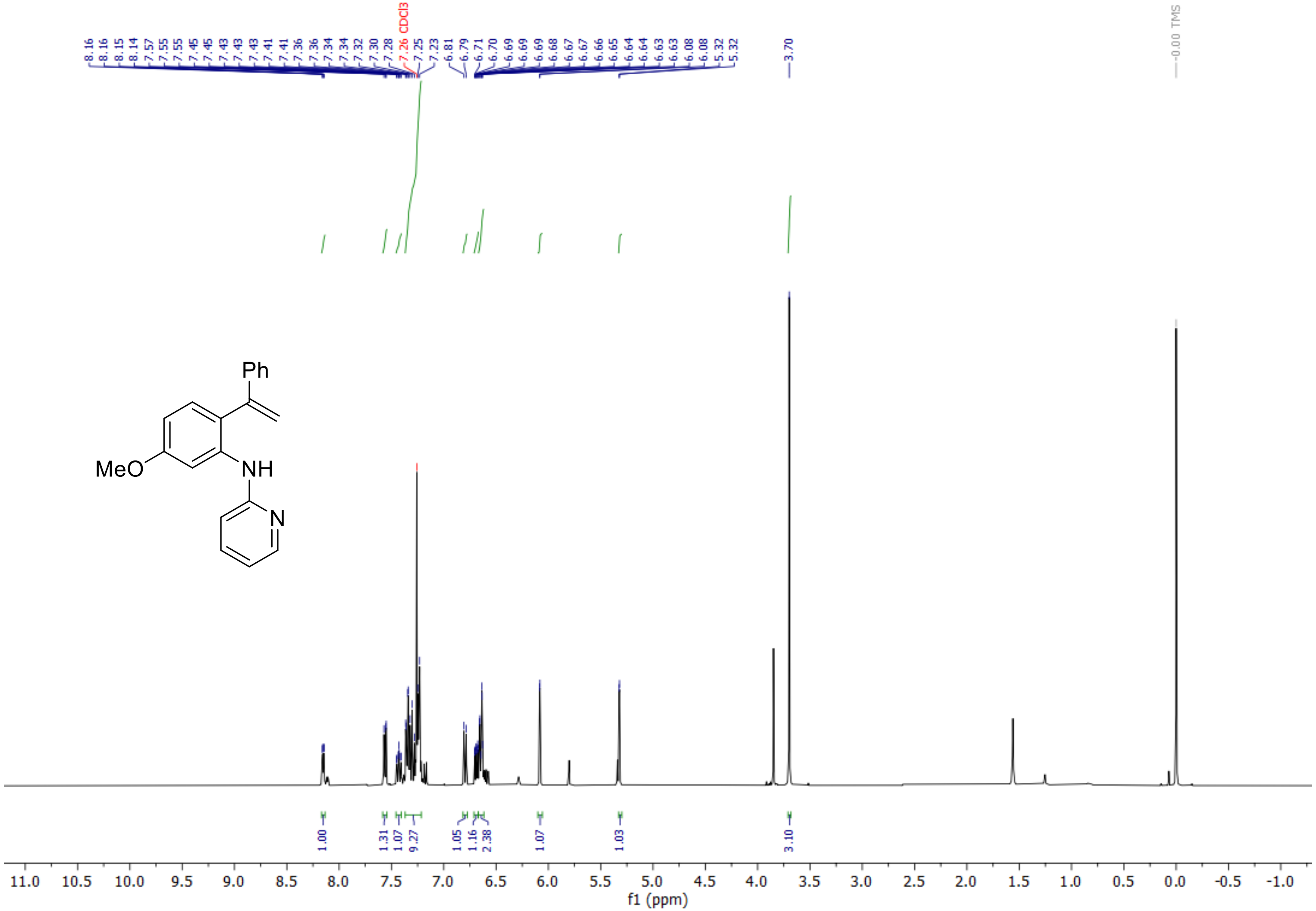

^{13}C NMR (101 MHz, $CDCl_3$) of **1m**

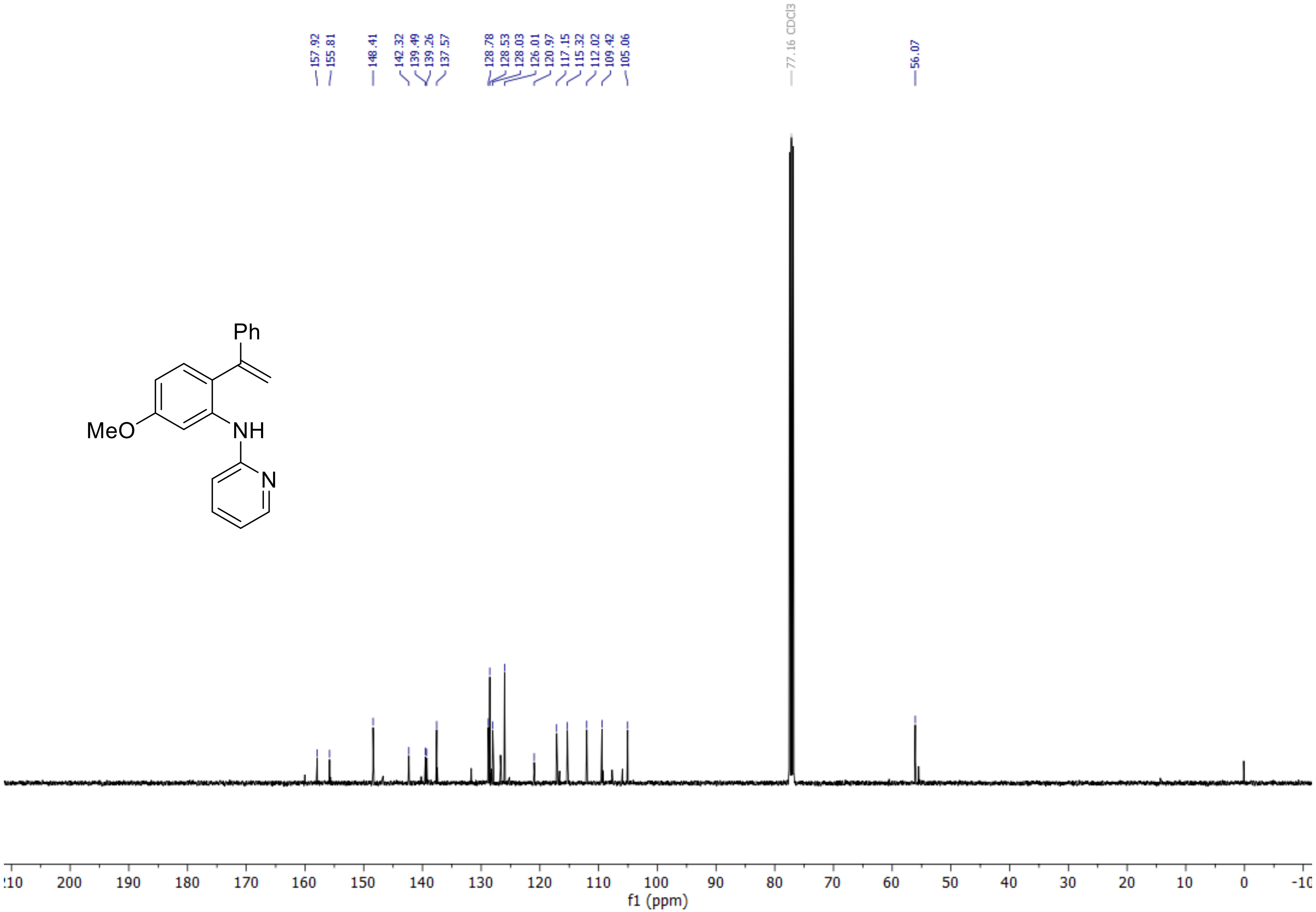

^{1}H–^{13}C HSQC-DEPT NMR (400 MHz, $CDCl_3$) of **1m**

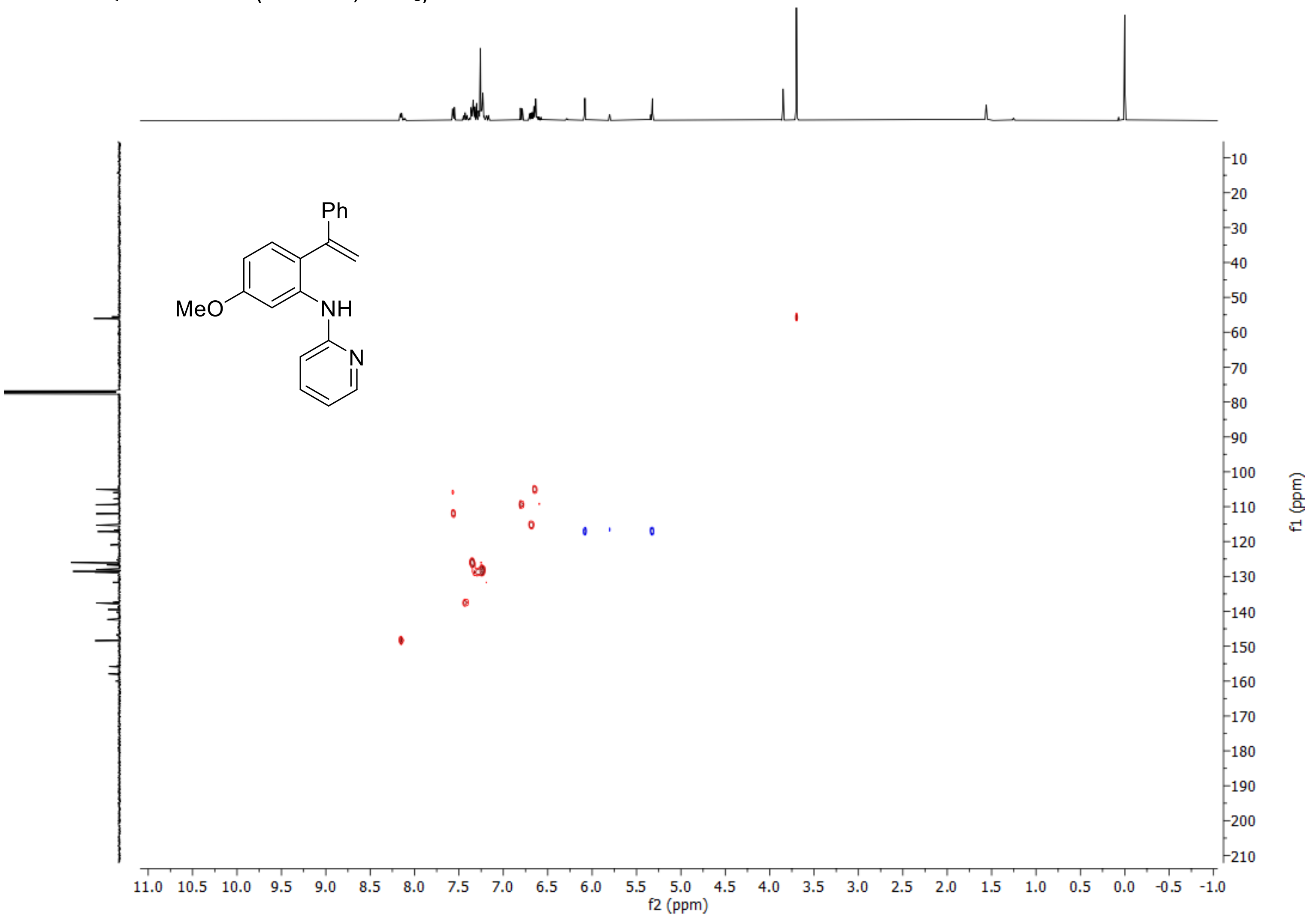

^1H NMR (400 MHz, $CDCl_3$) of **1n**

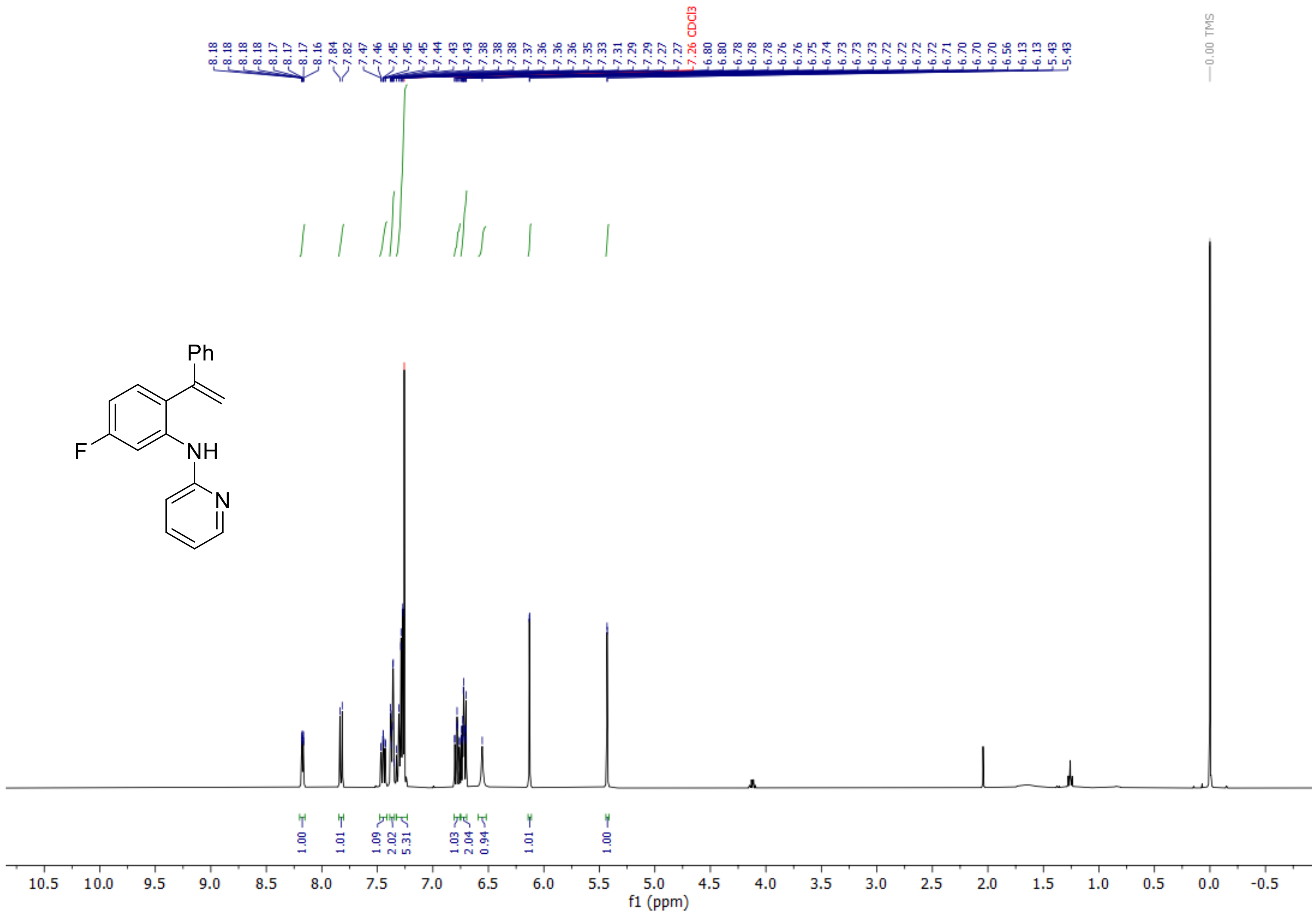

^{13}C NMR (101 MHz, CDCl₃) of **1n**

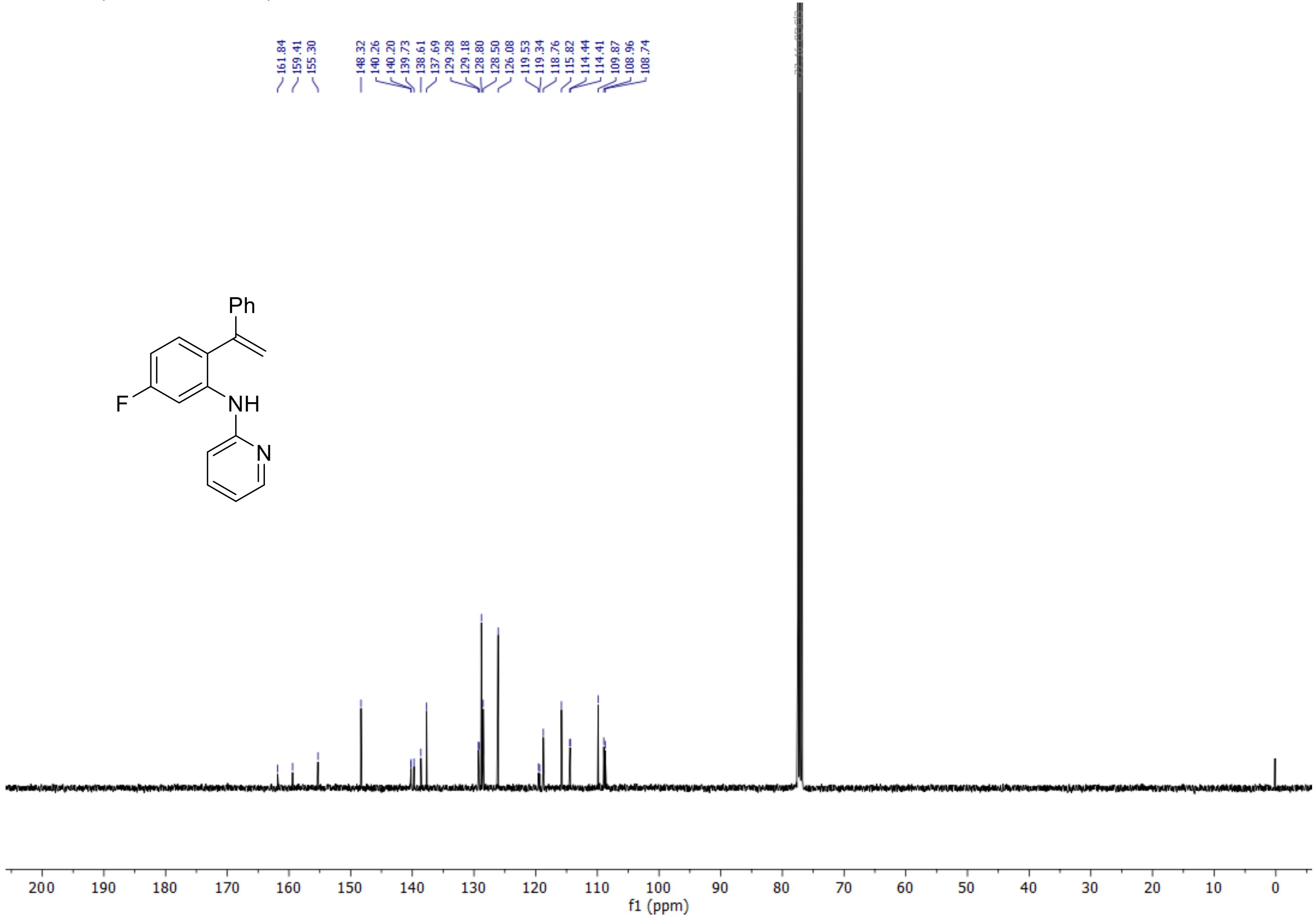

^{1}H–^{13}C HSQC-DEPT NMR (400 MHz, $CDCl_3$) of **1n**

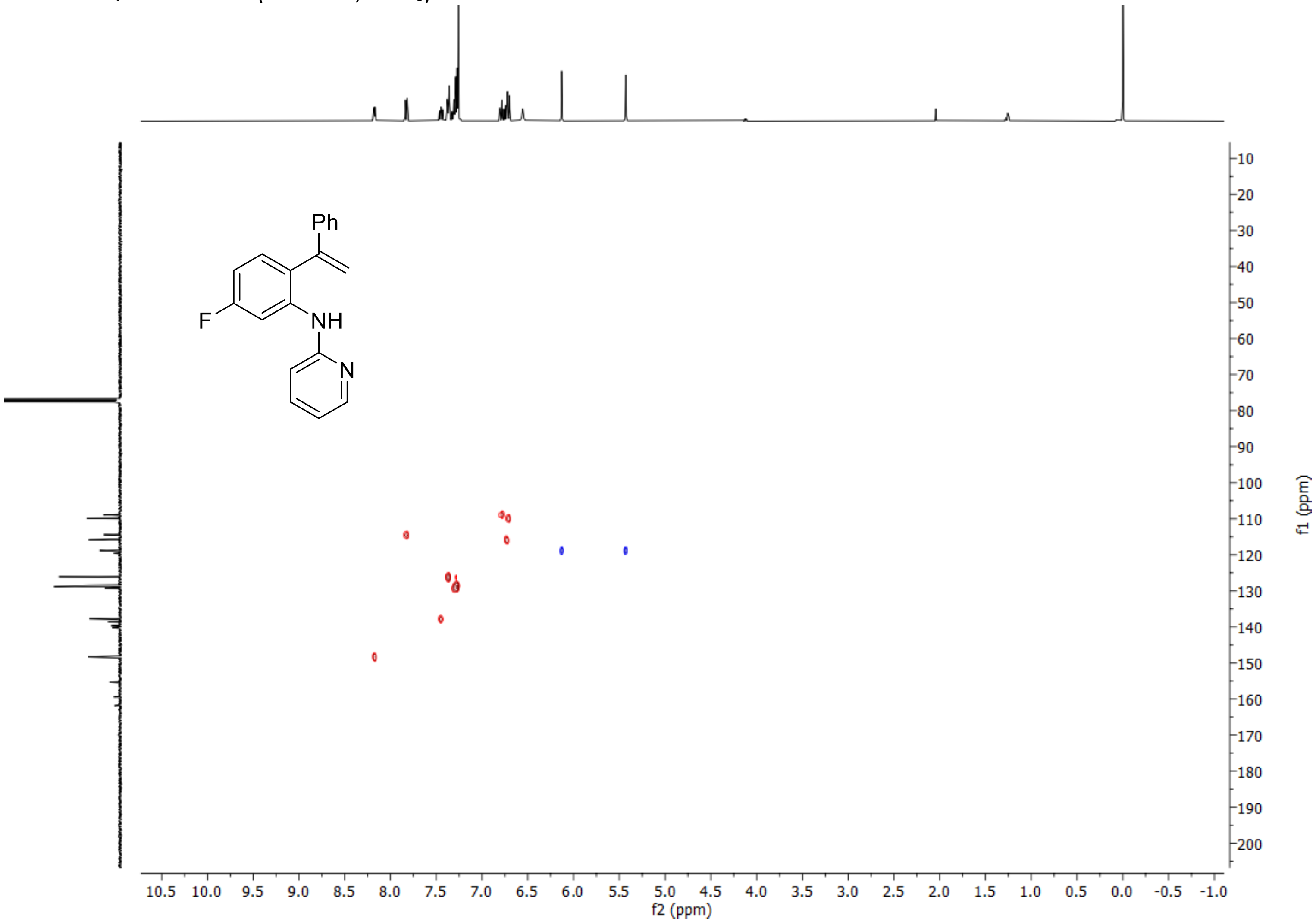

^{19}F NMR (376 MHz, $CDCl_3$) of **1n**

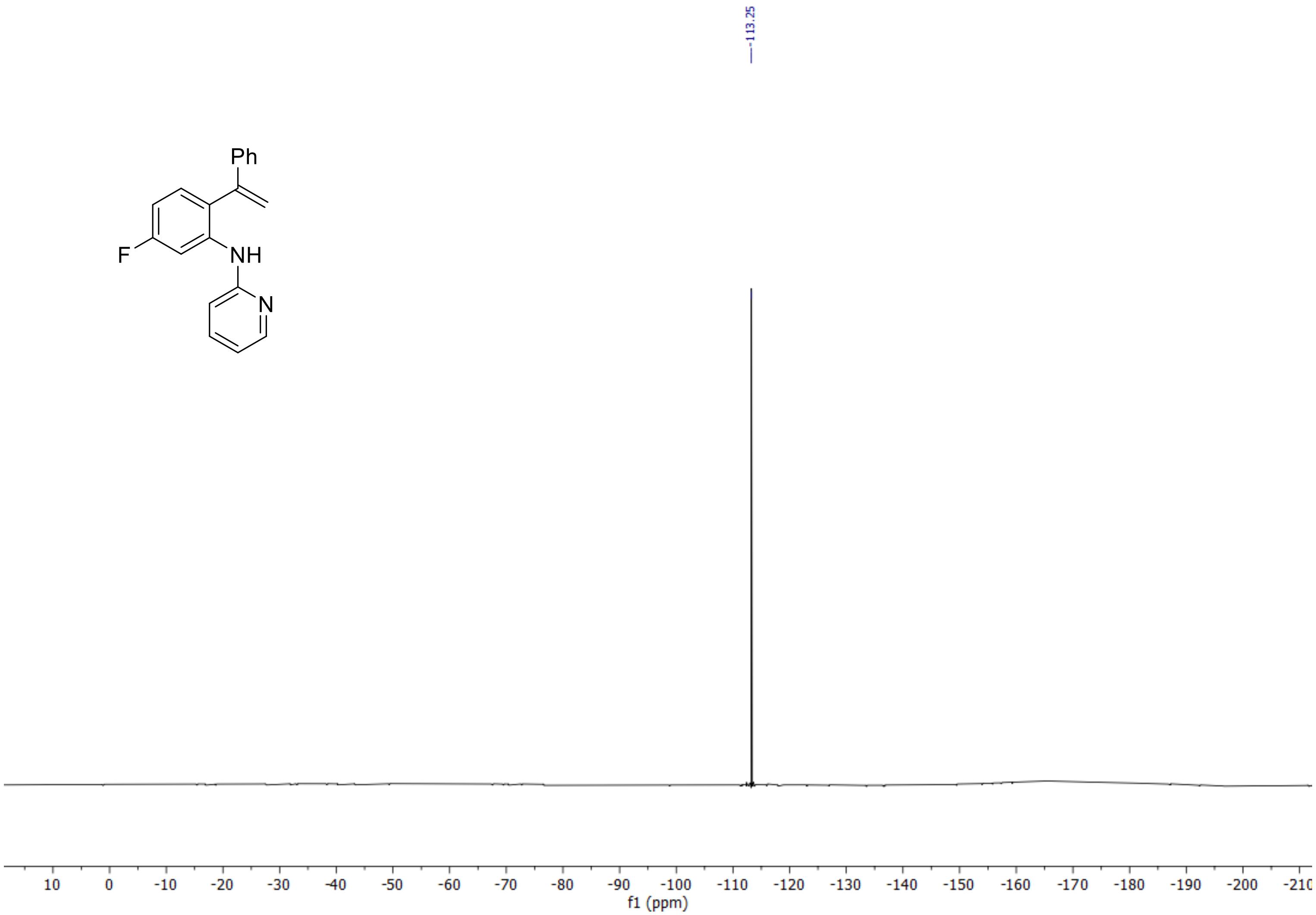

^{1}H NMR (400 MHz, $CDCl_3$) of **1o**

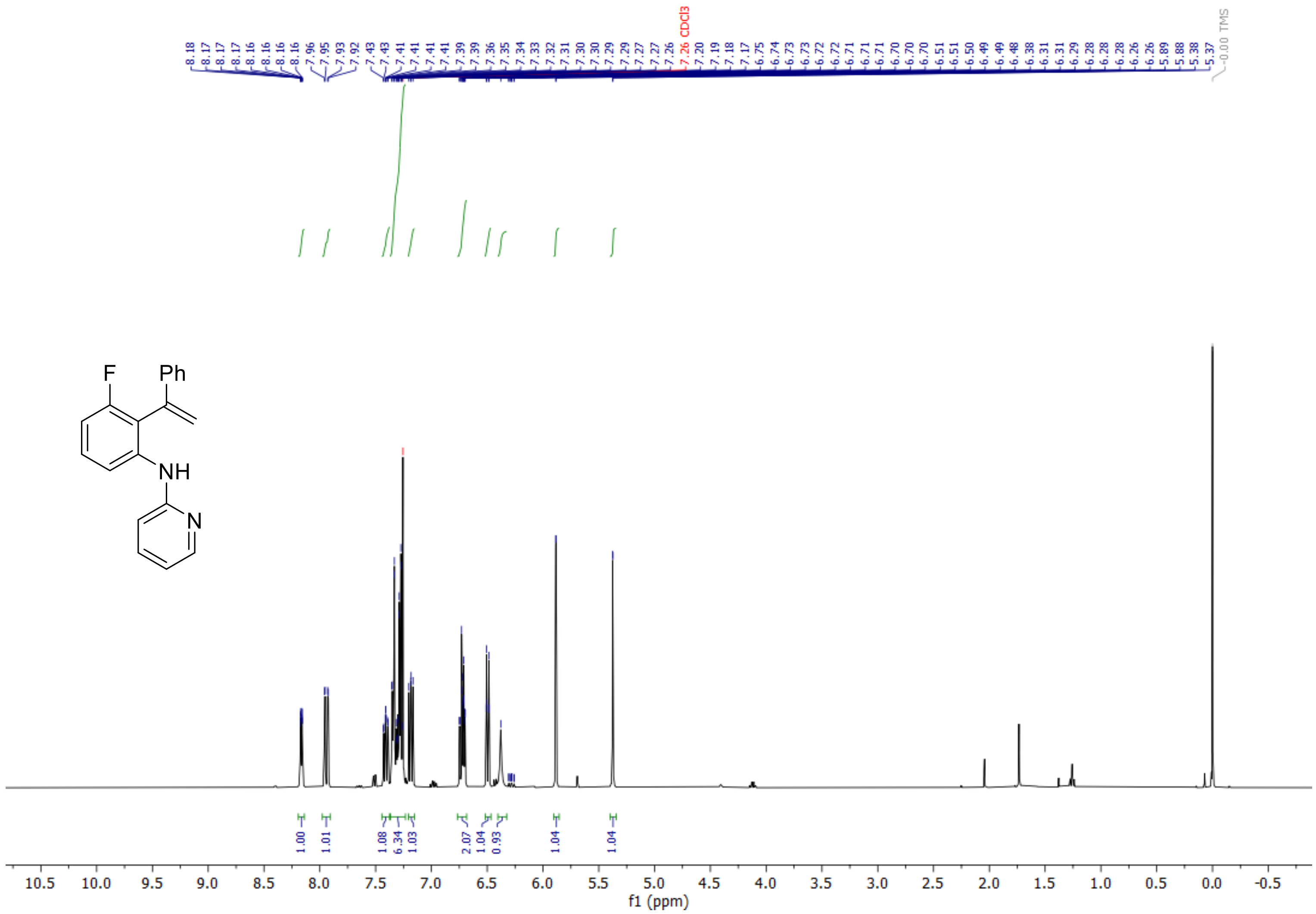

^{13}C NMR (101 MHz, $CDCl_3$) of **1o**

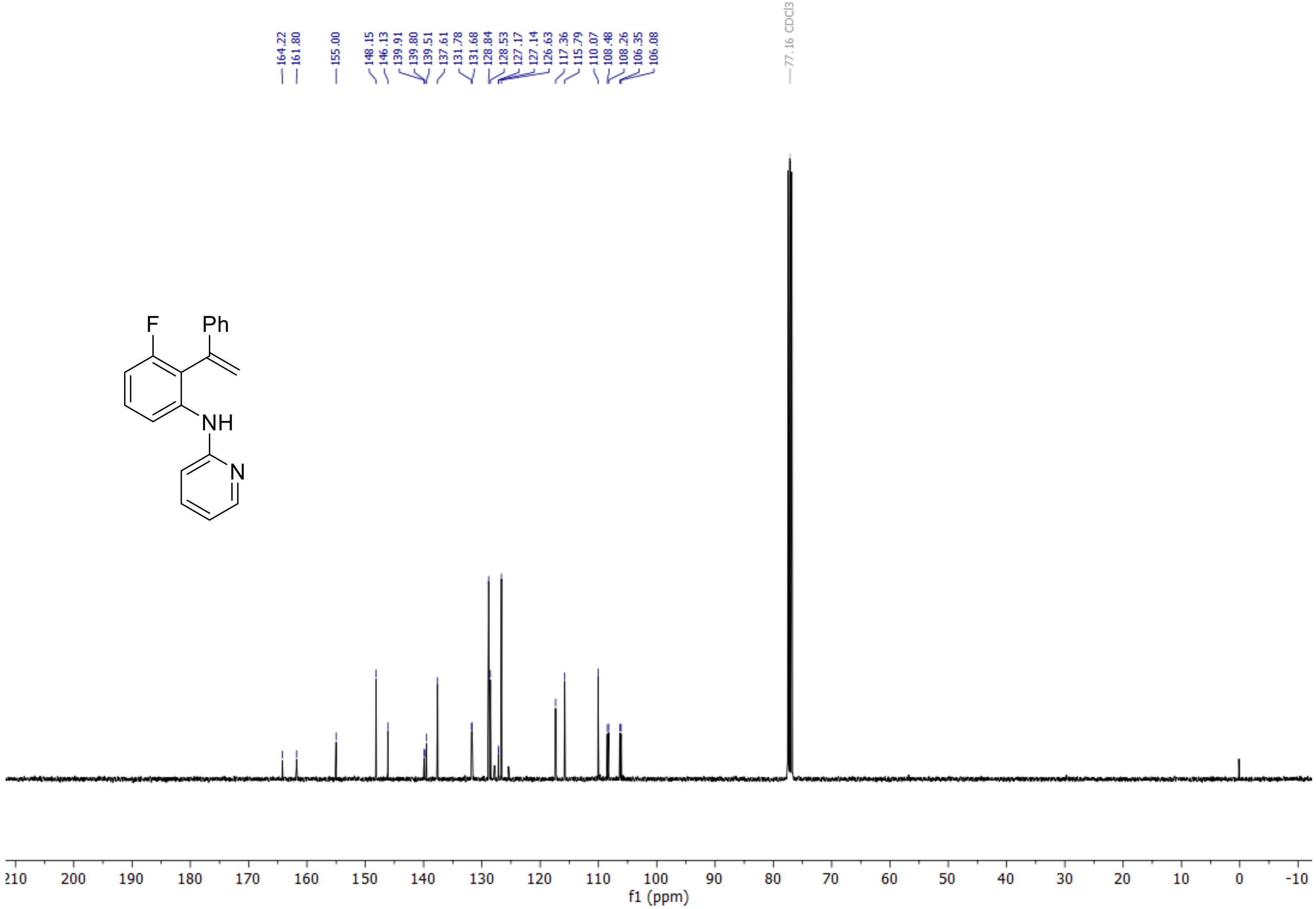

^1H–^{13}C HSQC-DEPT NMR (400 MHz, $CDCl_3$) of **1o**

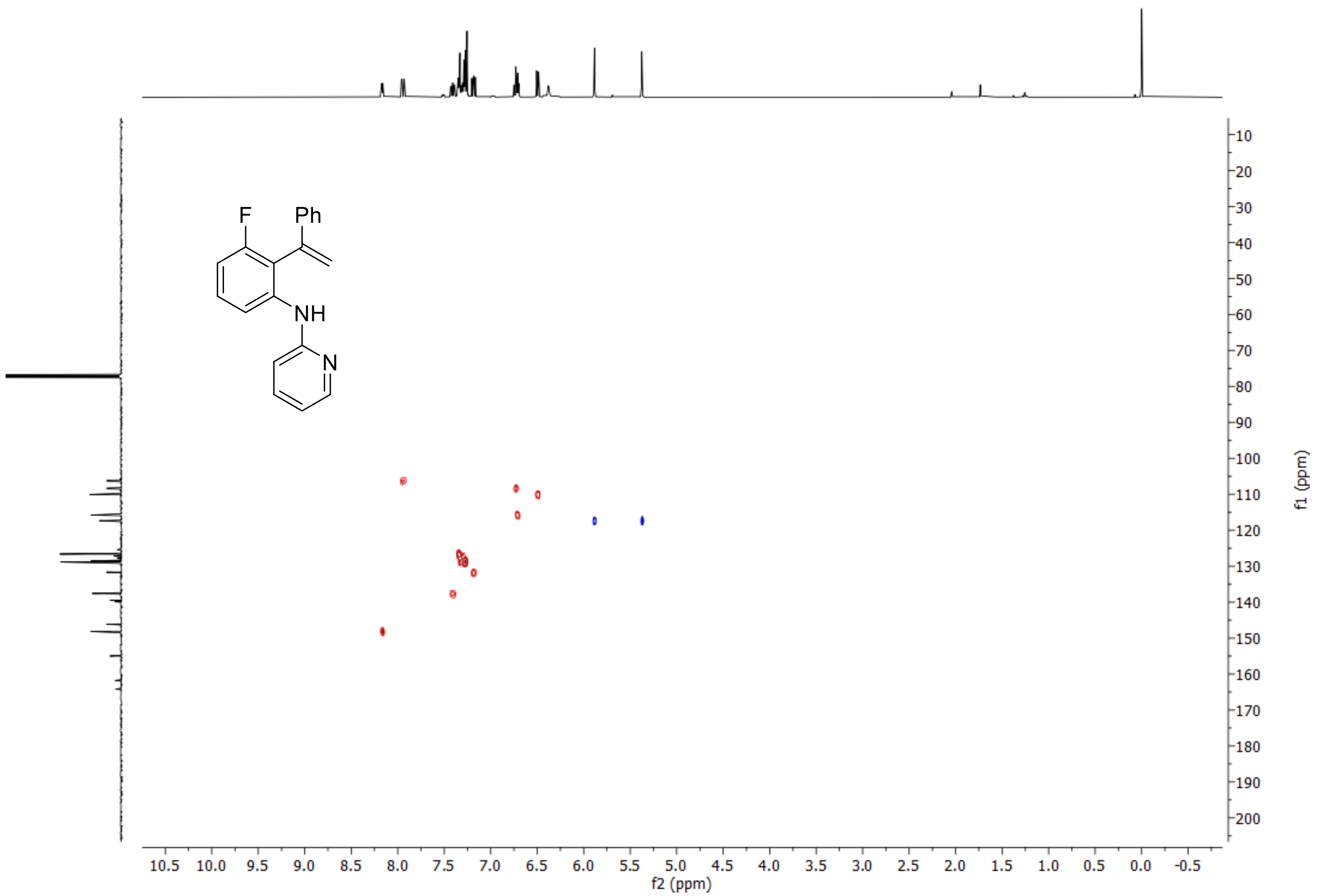

^{19}F NMR (376 MHz, $CDCl_3$) of **1o**

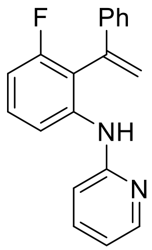

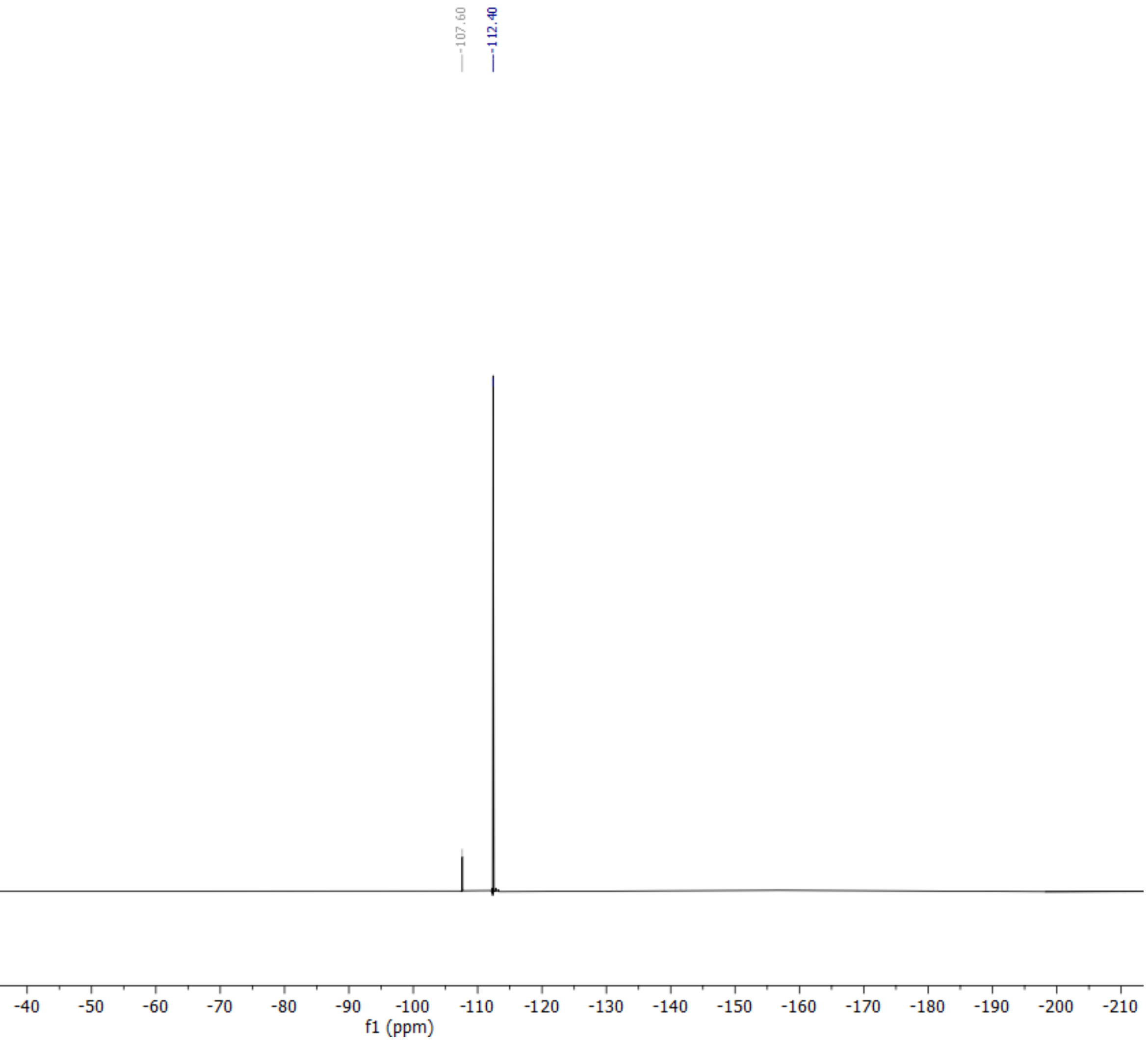

^{1}H NMR (400 MHz, $CDCl_3$) of **1p**

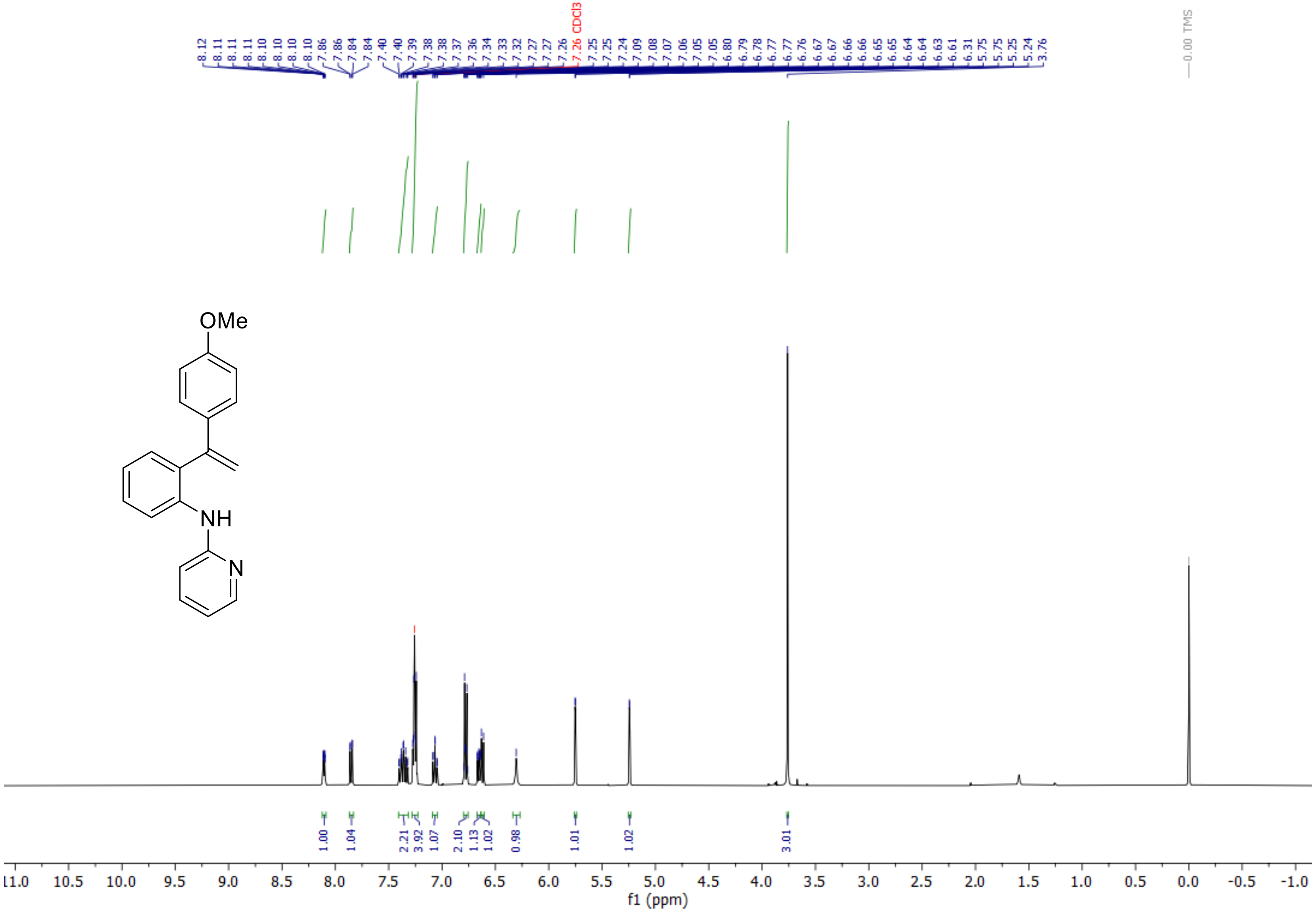

^{13}C NMR (101 MHz, $CDCl_3$) of **1p**

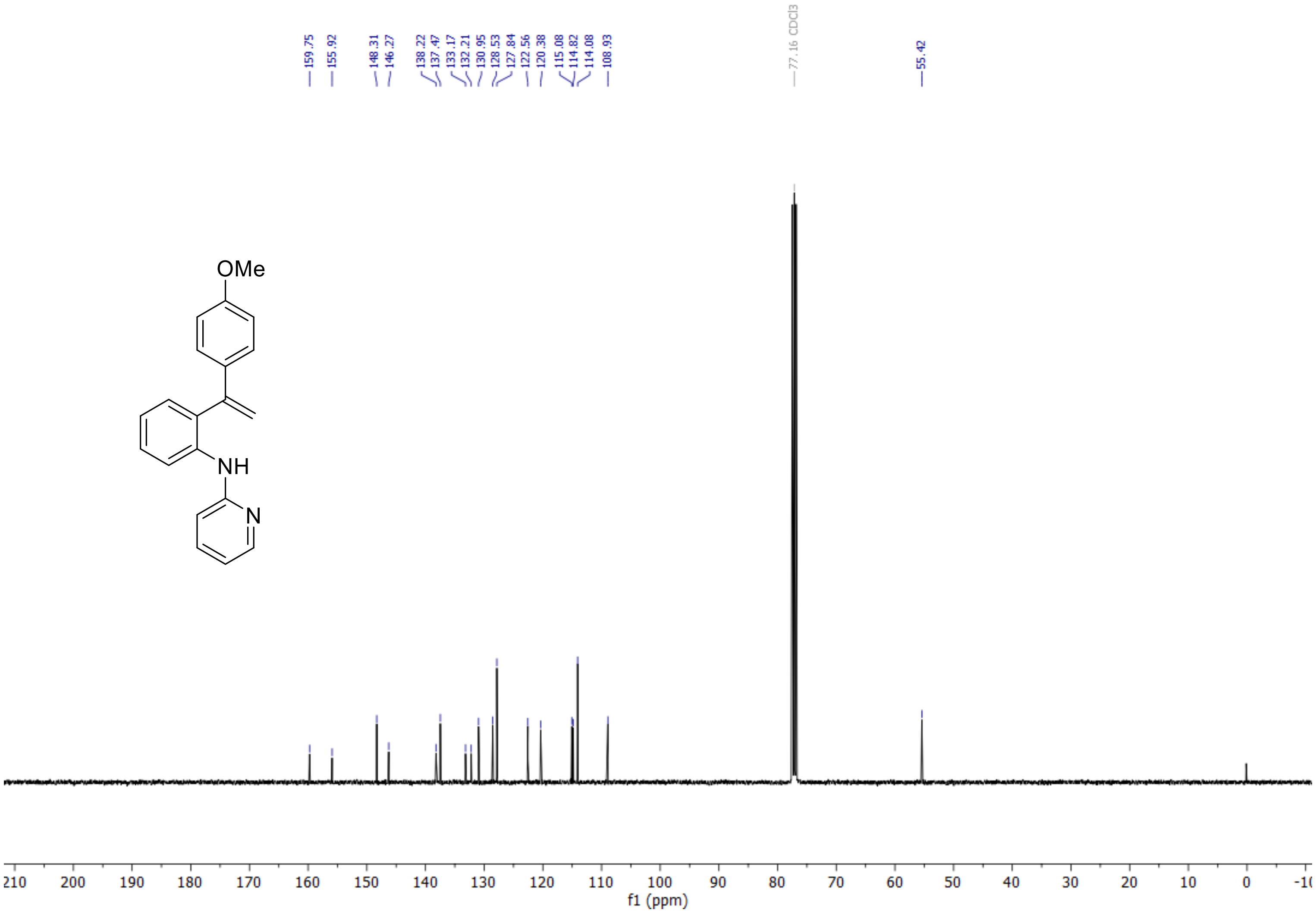

^1H–^{13}C HSQC-DEPT NMR (400 MHz, $CDCl_3$) of **1p**

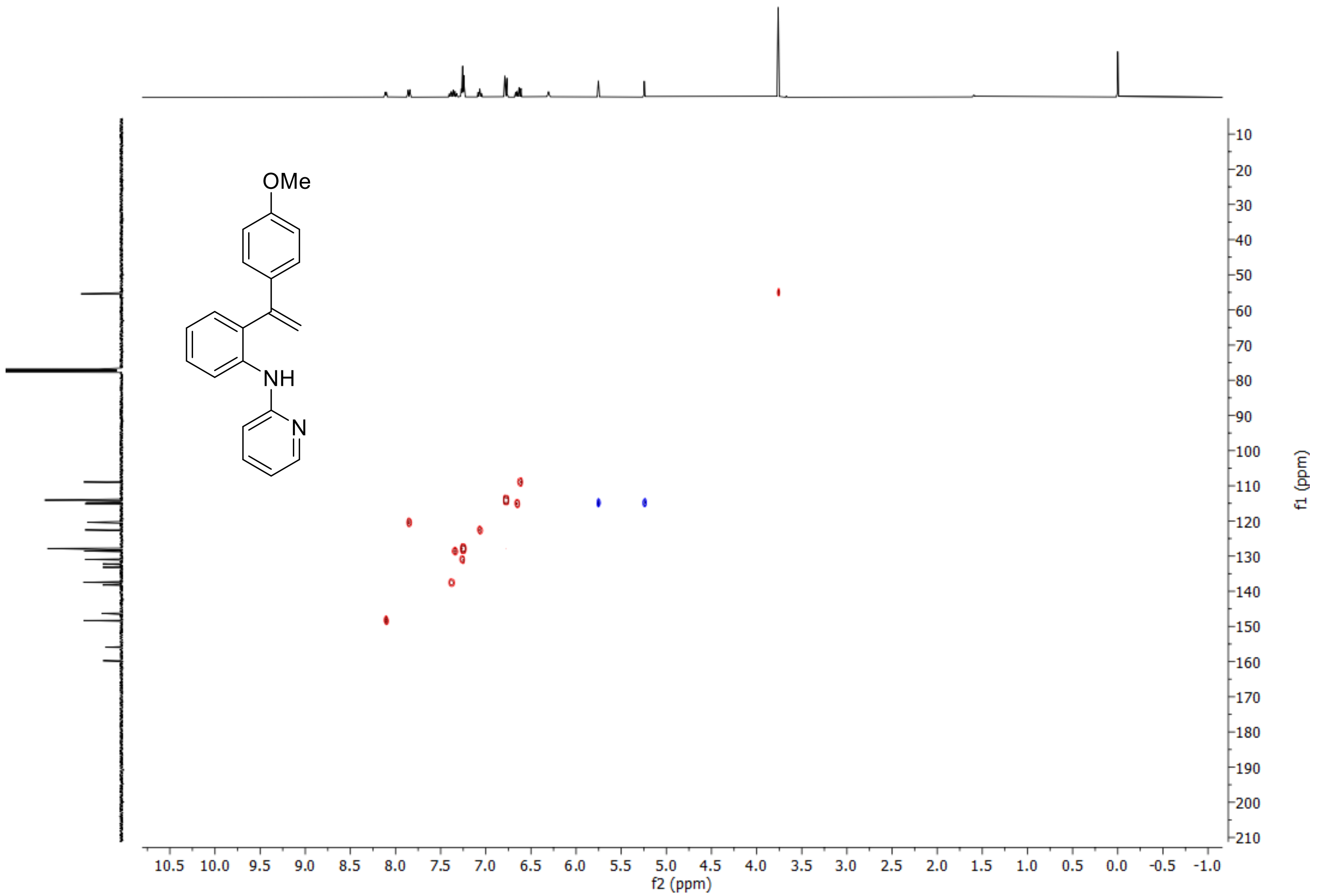

^{1}H NMR (400 MHz, $CDCl_3$) of **1q**

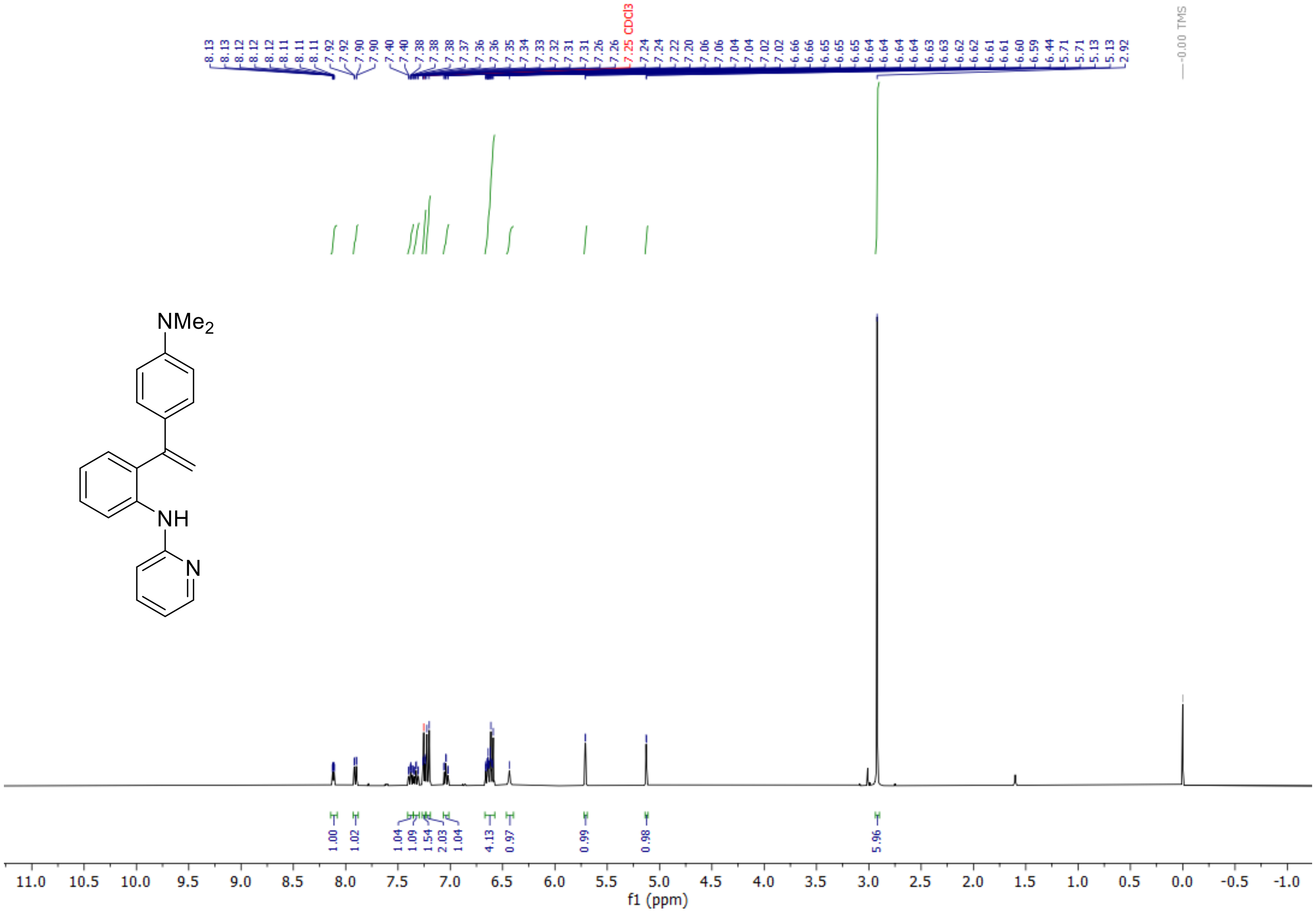

^{13}C NMR (101 MHz, $CDCl_3$) of **1q**

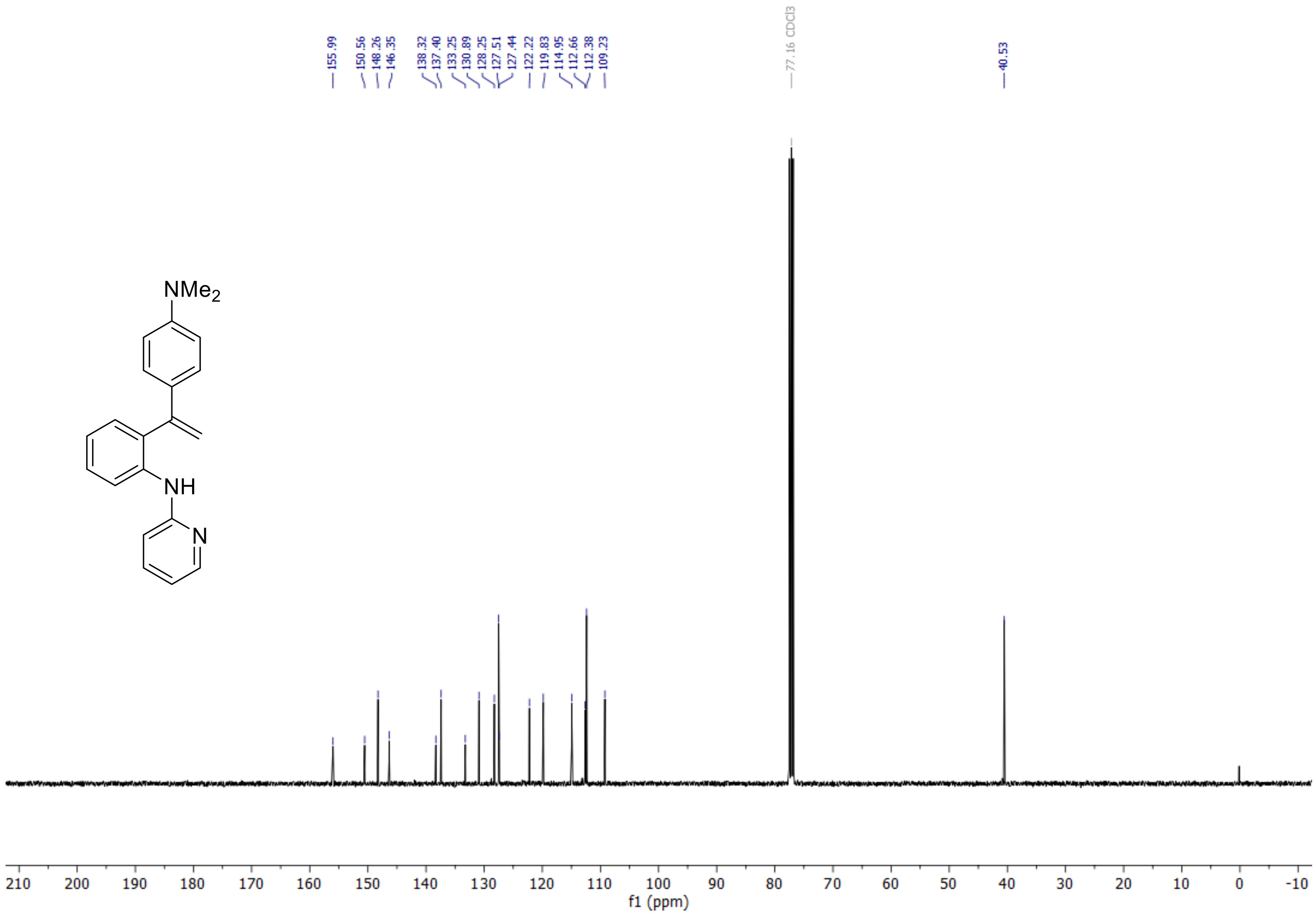

^{1}H–^{13}C HSQC-DEPT NMR (400 MHz, $CDCl_3$) of **1q**

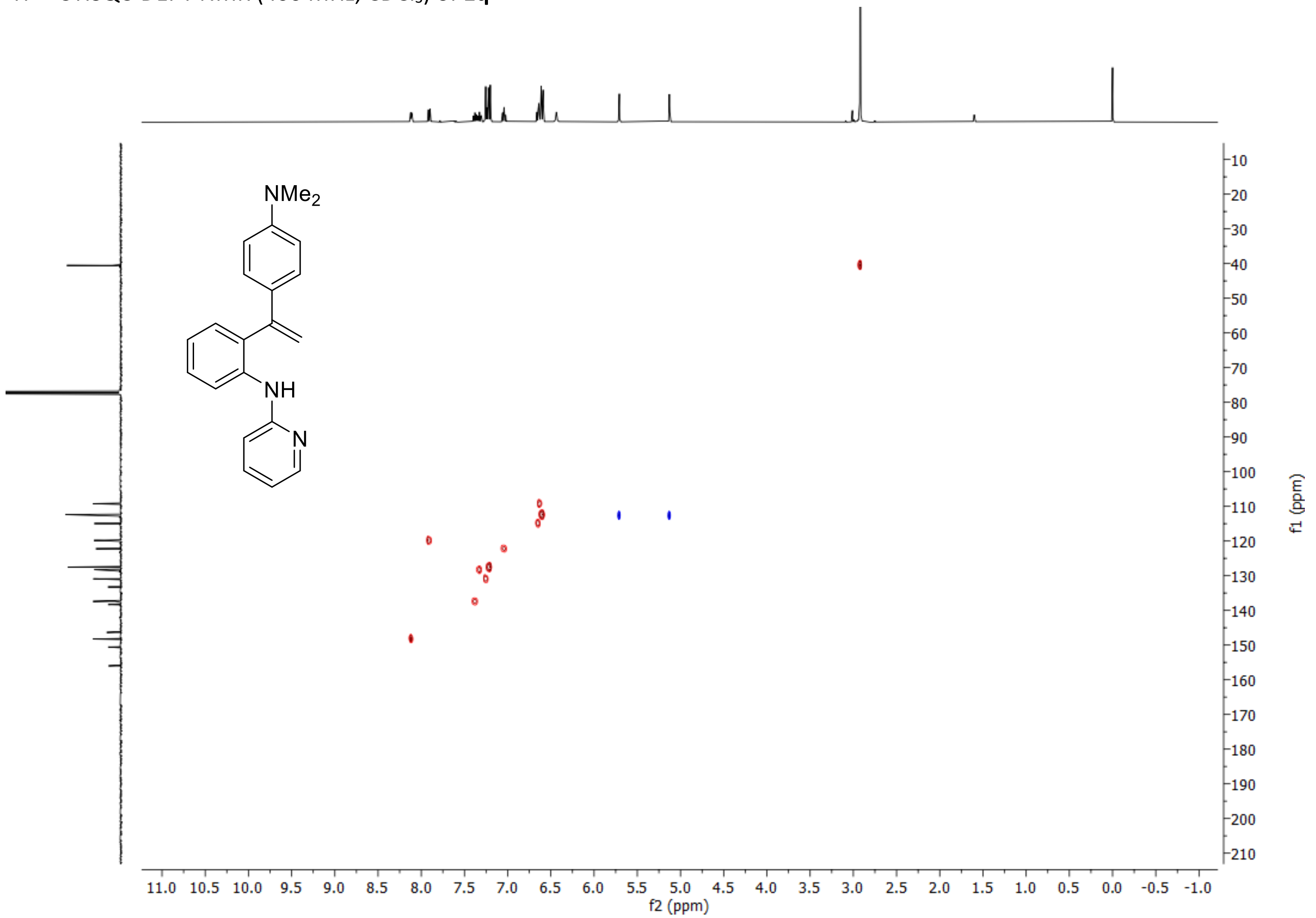

^{1}H NMR (400 MHz, $CDCl_3$) of **1r**

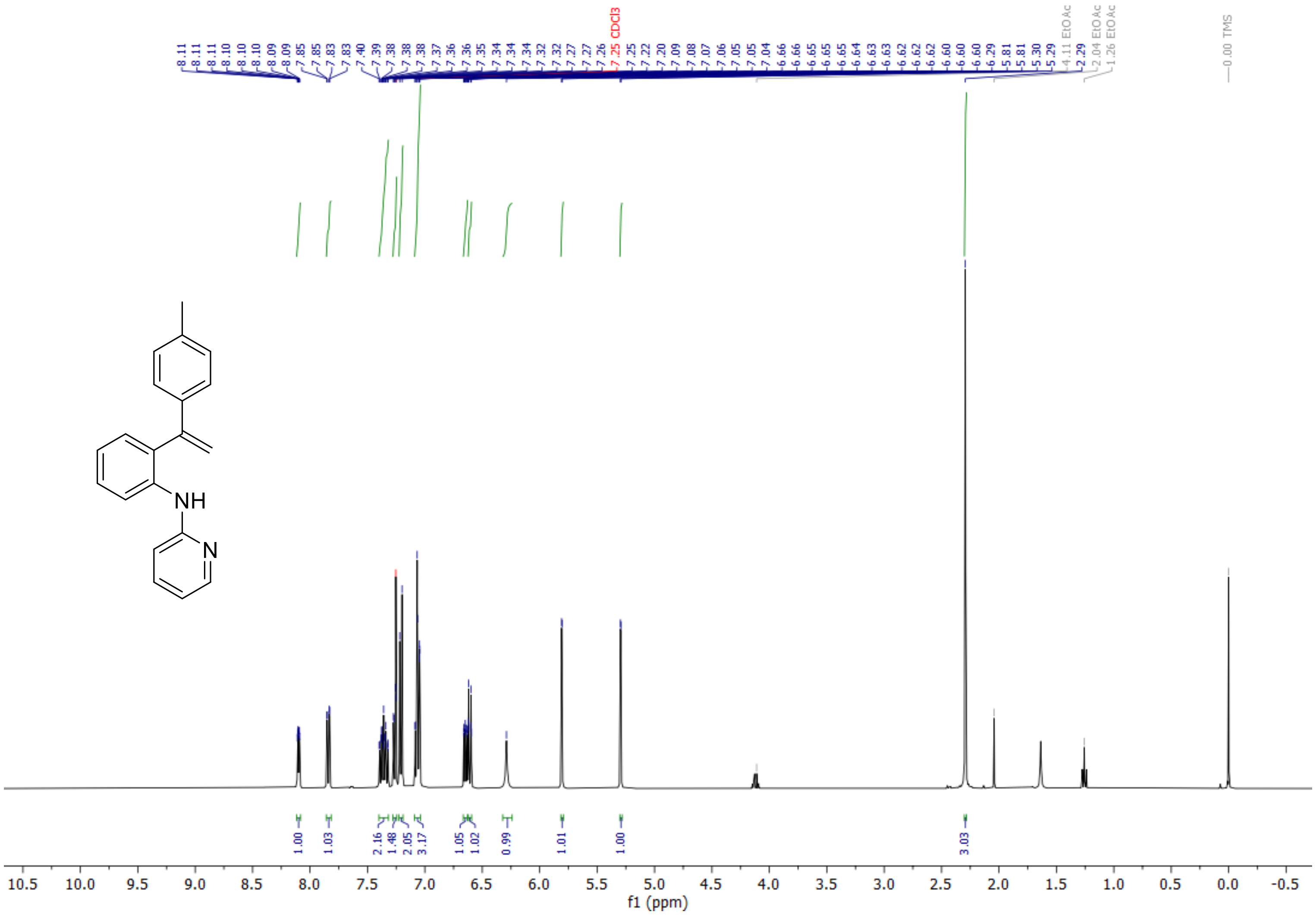

^{13}C NMR (101 MHz, $CDCl_3$) of **1r**

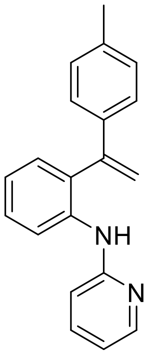

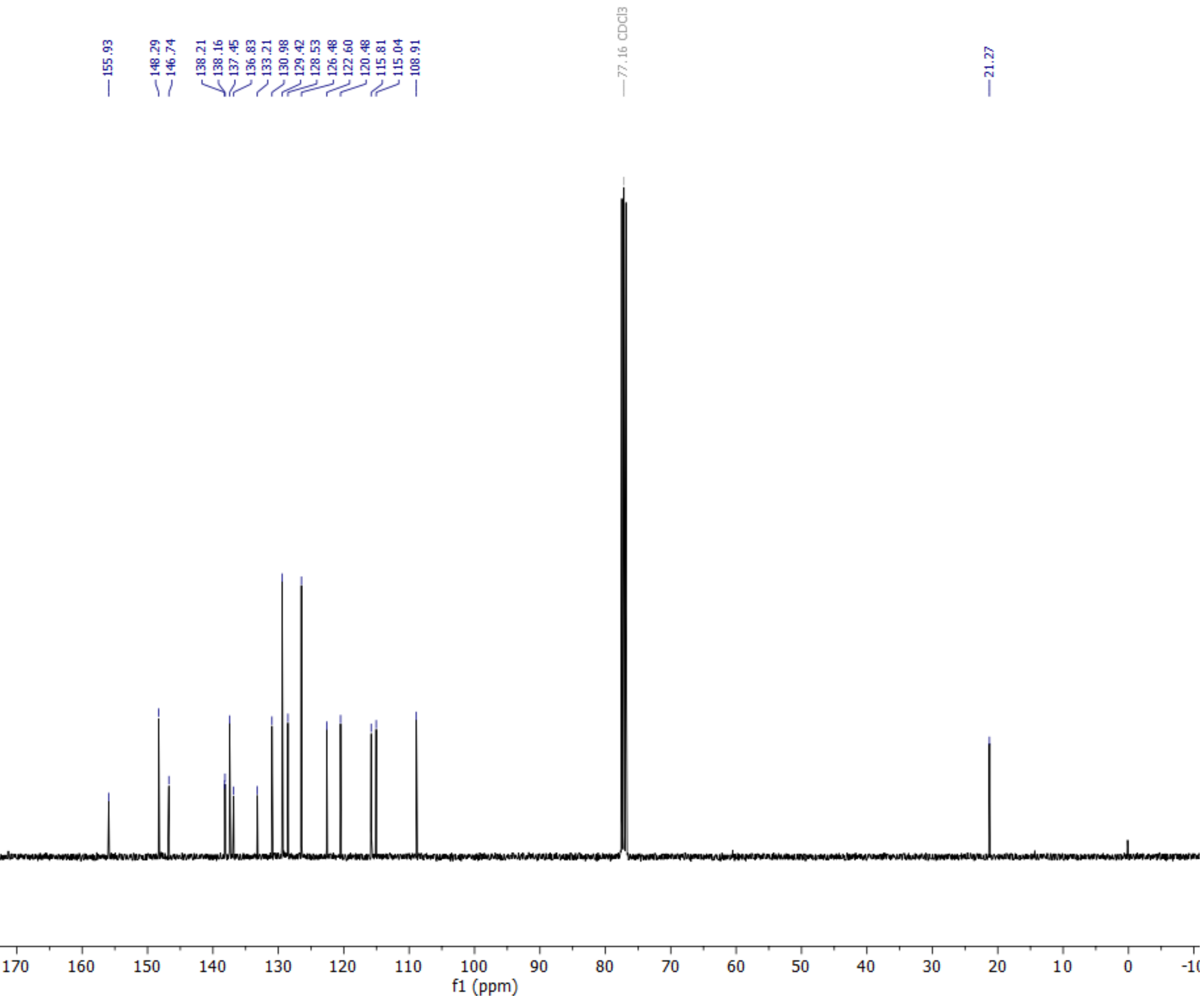

$^{1}H–^{13}C$ HSQC-DEPT NMR (400 MHz, $CDCl_3$) of **1r**

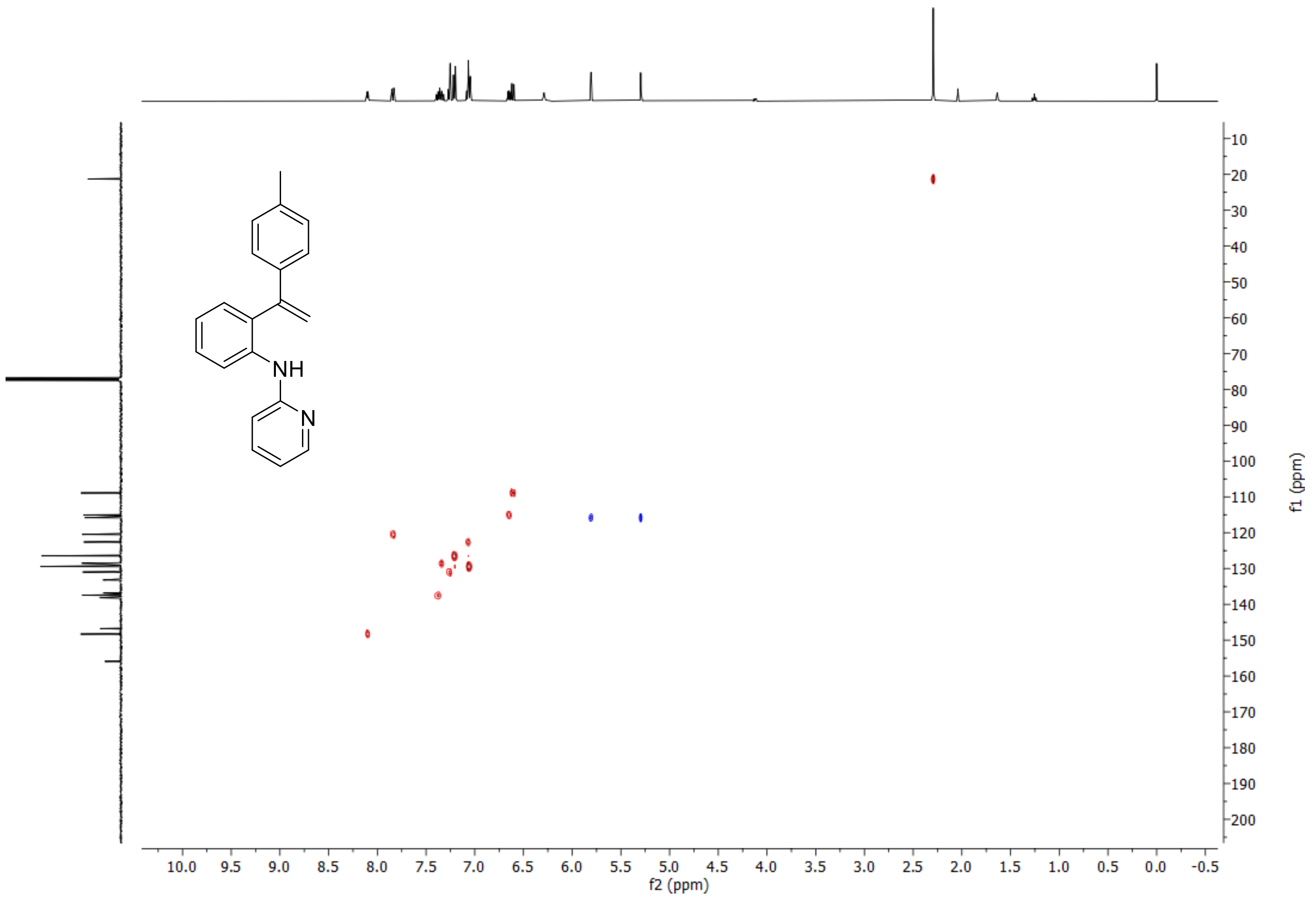

^{1}H NMR (400 MHz, $CDCl_3$) of **1s**

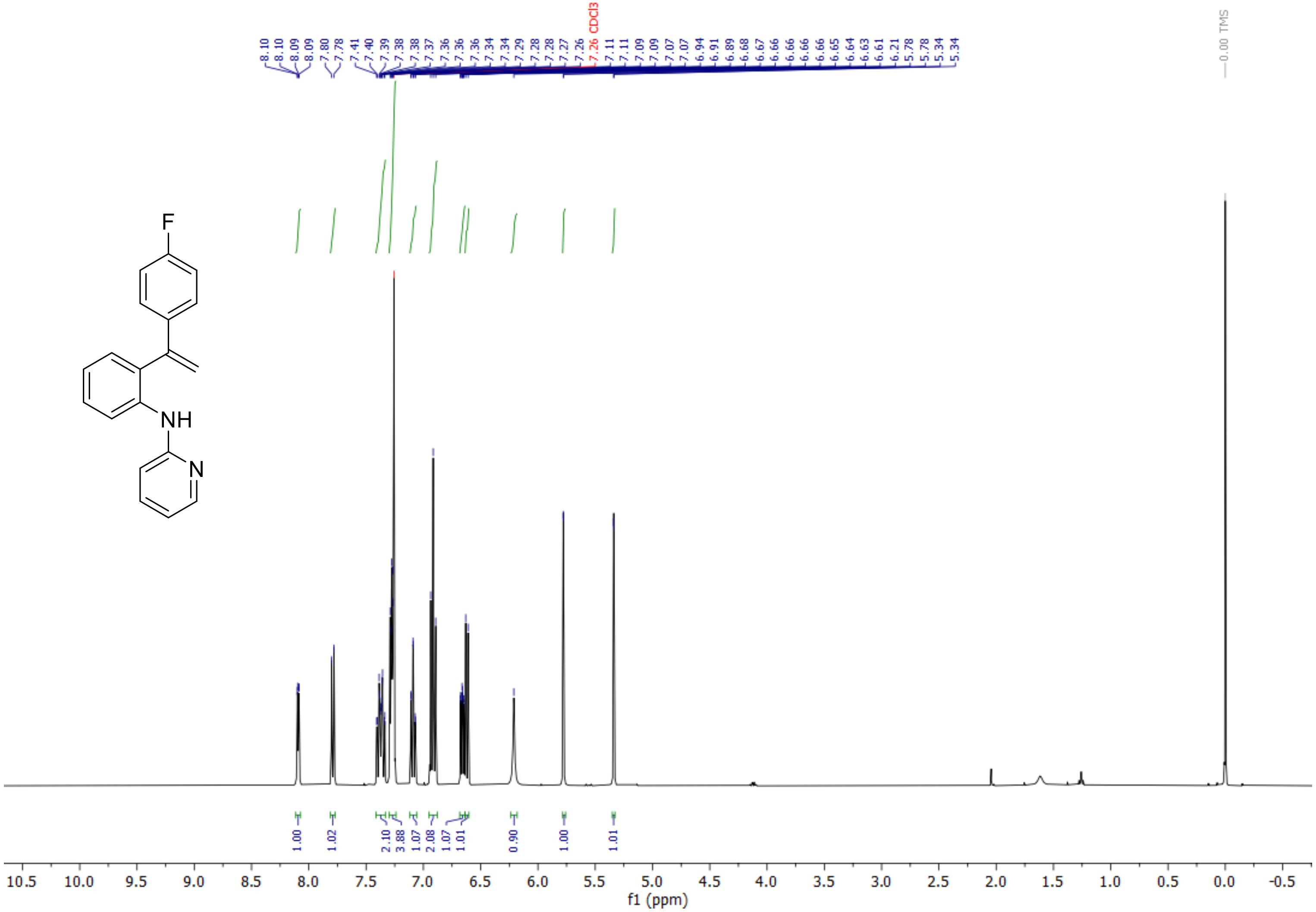

^{13}C NMR (101 MHz, $CDCl_3$) of **1s**

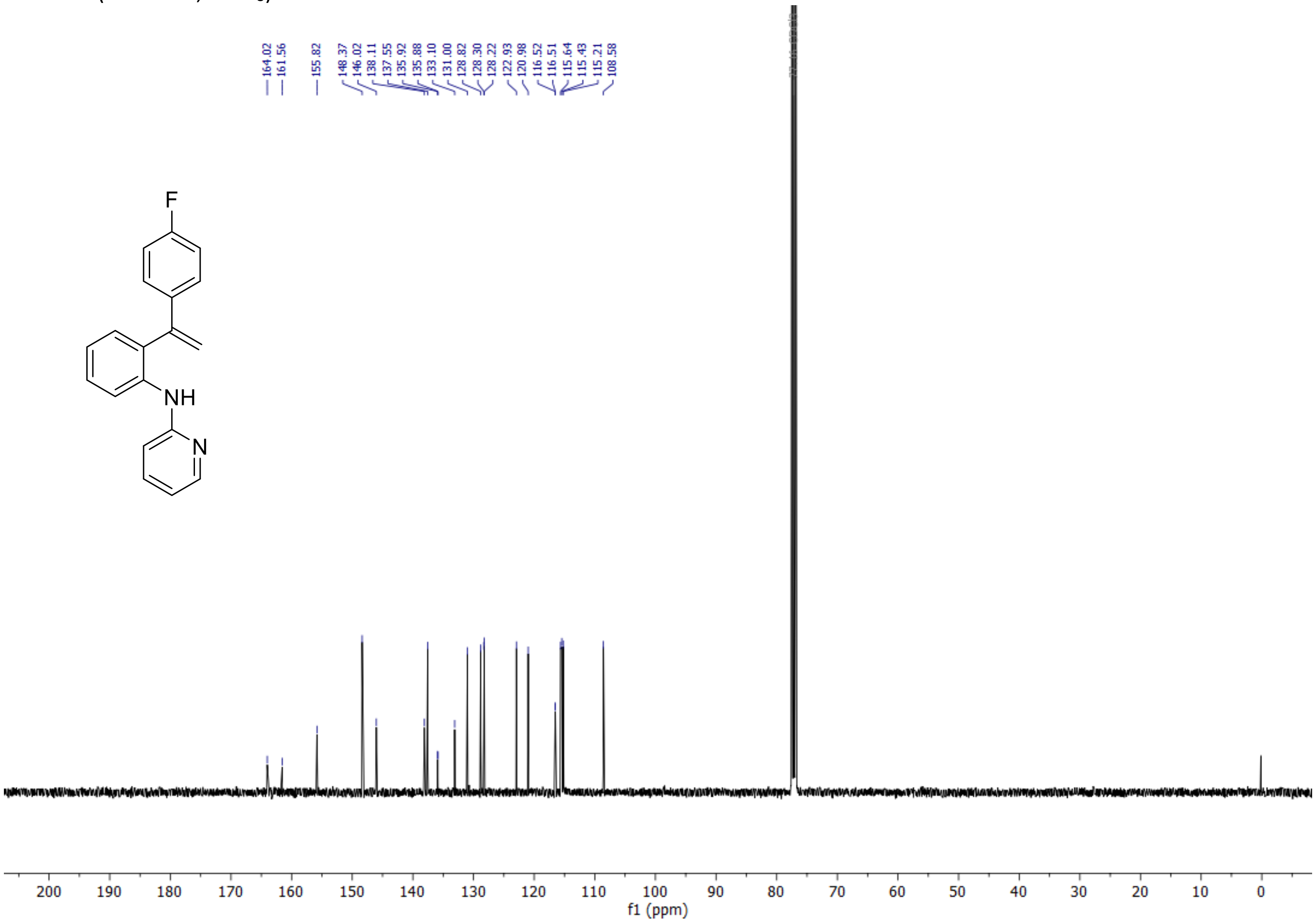

^{1}H–^{13}C HSQC-DEPT NMR (400 MHz, $CDCl_3$) of **1s**

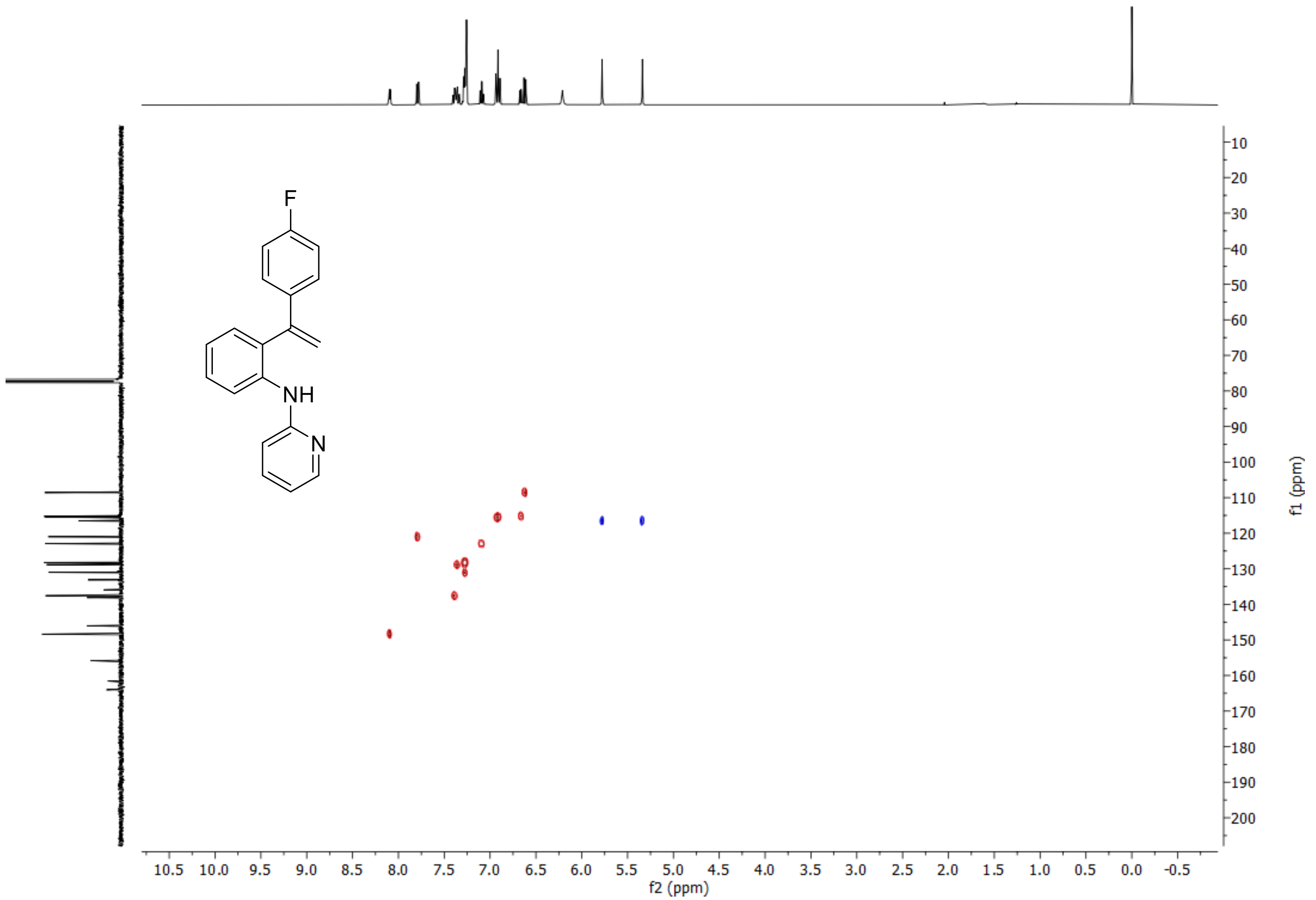

^{19}F NMR (376 MHz, $CDCl_3$) of **1s**

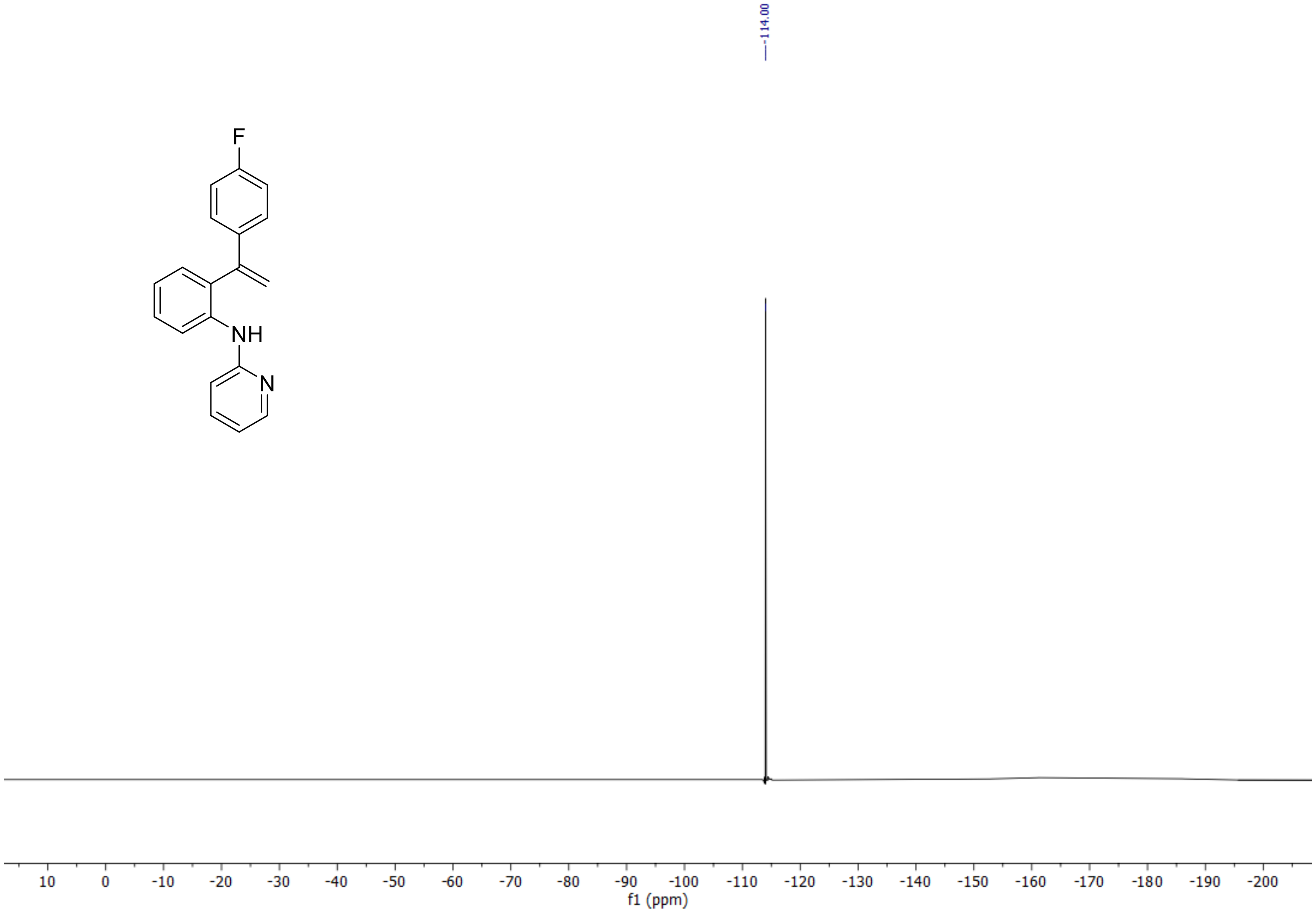

^1H NMR (400 MHz, $CDCl_3$) of **1t**

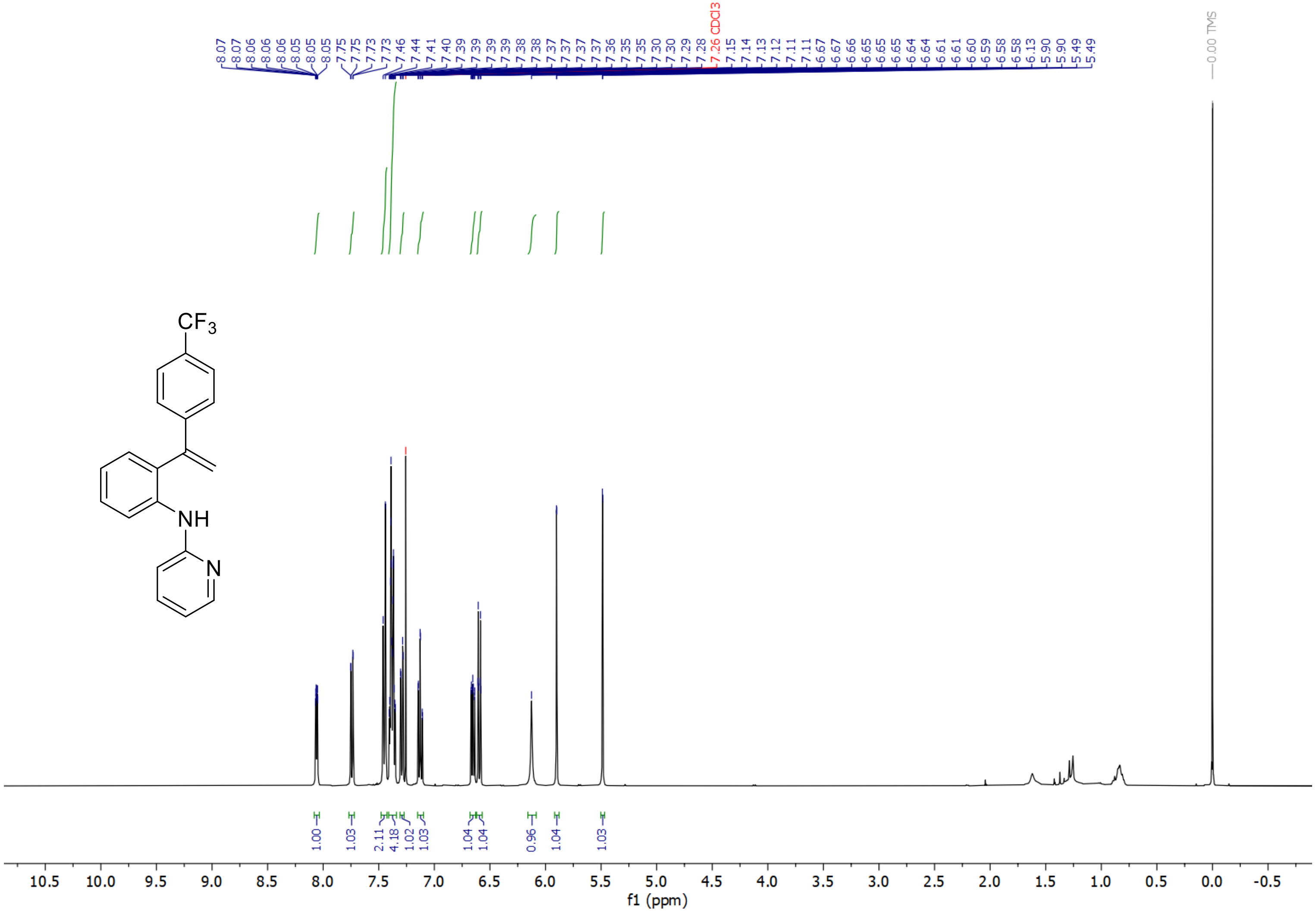

^{13}C NMR (101 MHz, $CDCl_3$) of **1t**

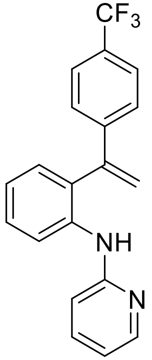

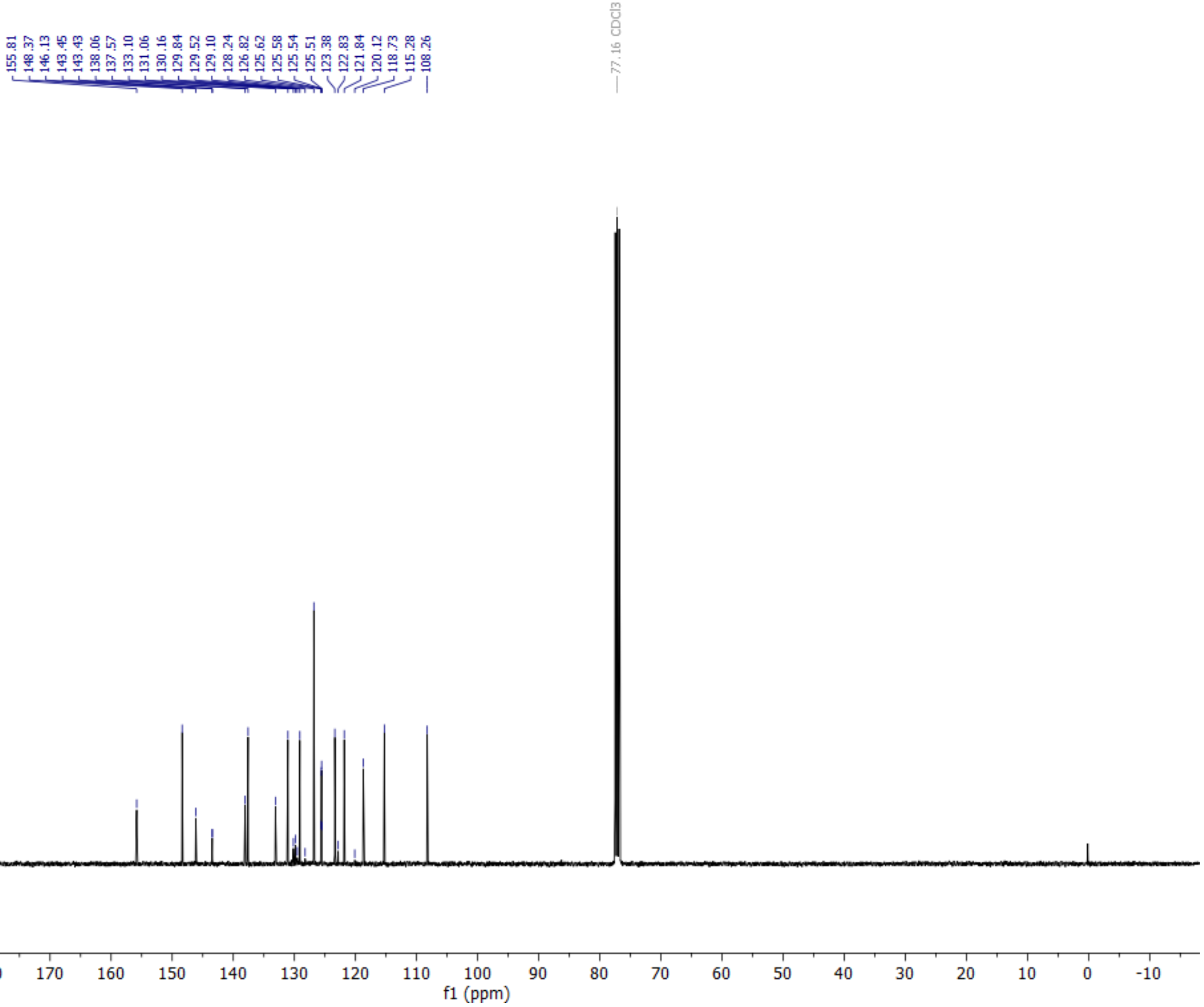

$^1H–^{13}C$ HSQC-DEPT NMR (400 MHz, $CDCl_3$) of **1t**

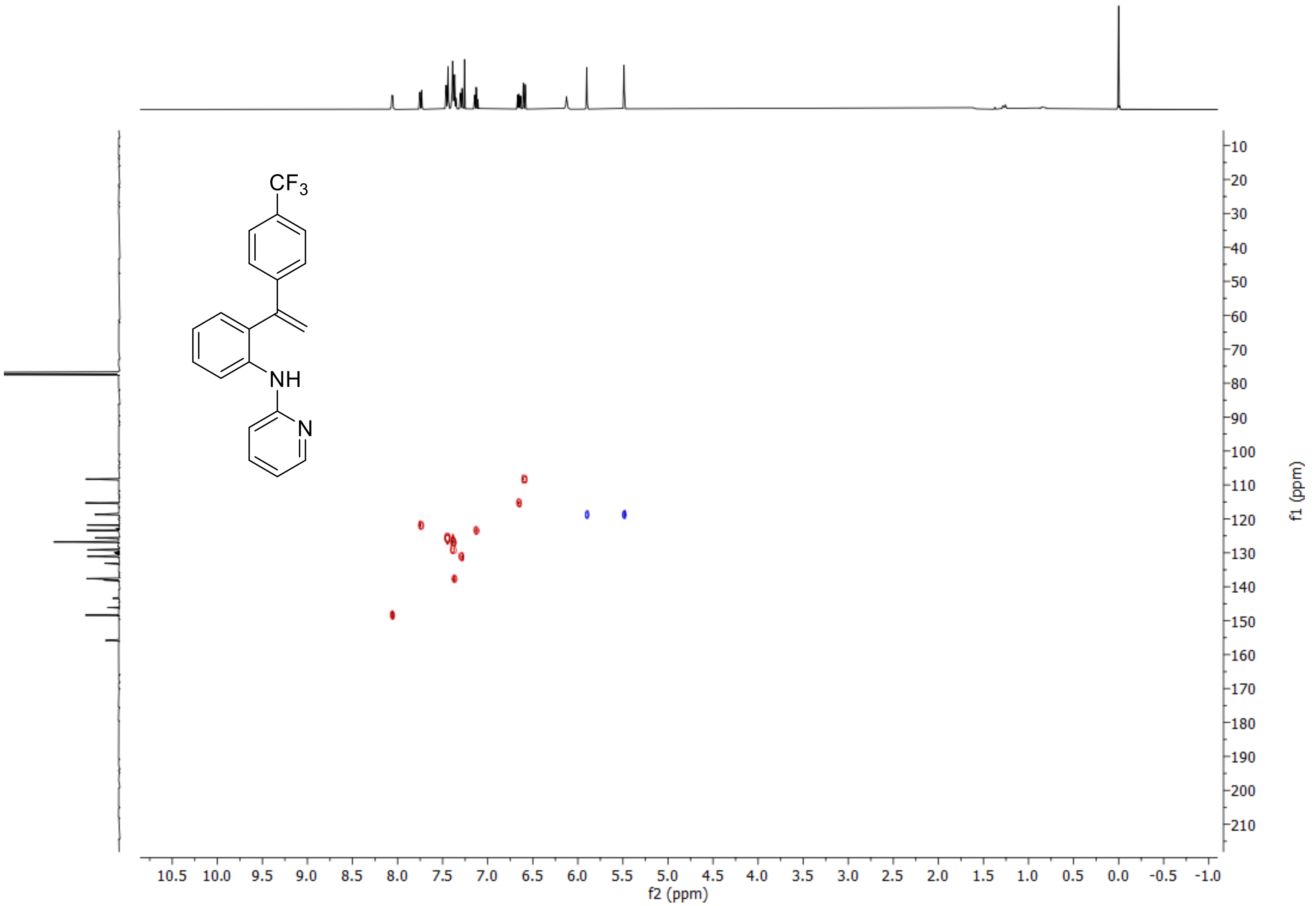

^{19}F NMR (376 MHz, $CDCl_3$) of **1t**

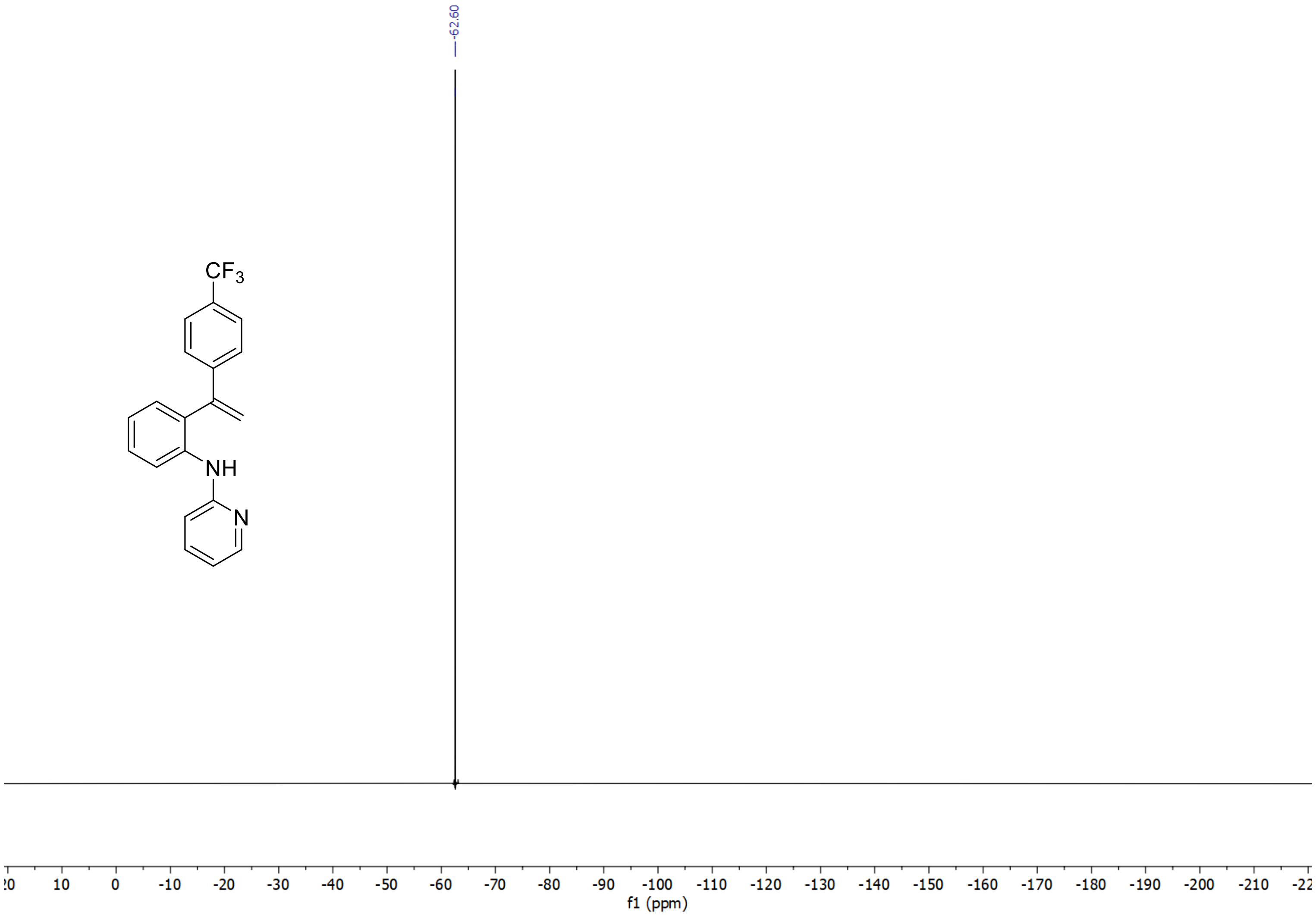

^{1}H NMR (400 MHz, $CDCl_3$) of **1u**

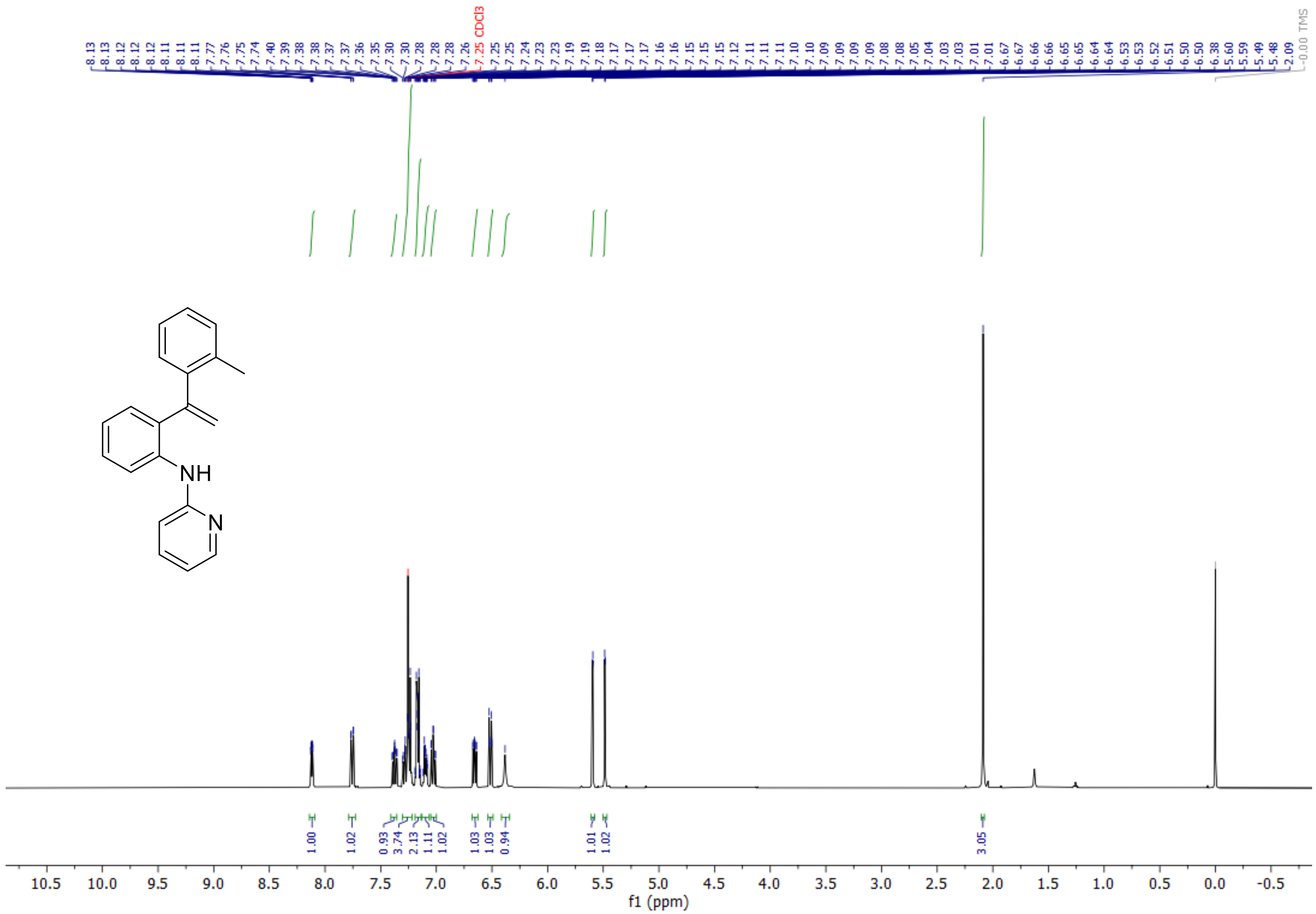

^{13}C NMR (101 MHz, $CDCl_3$) of **1u**

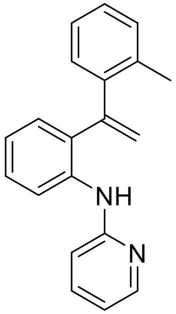

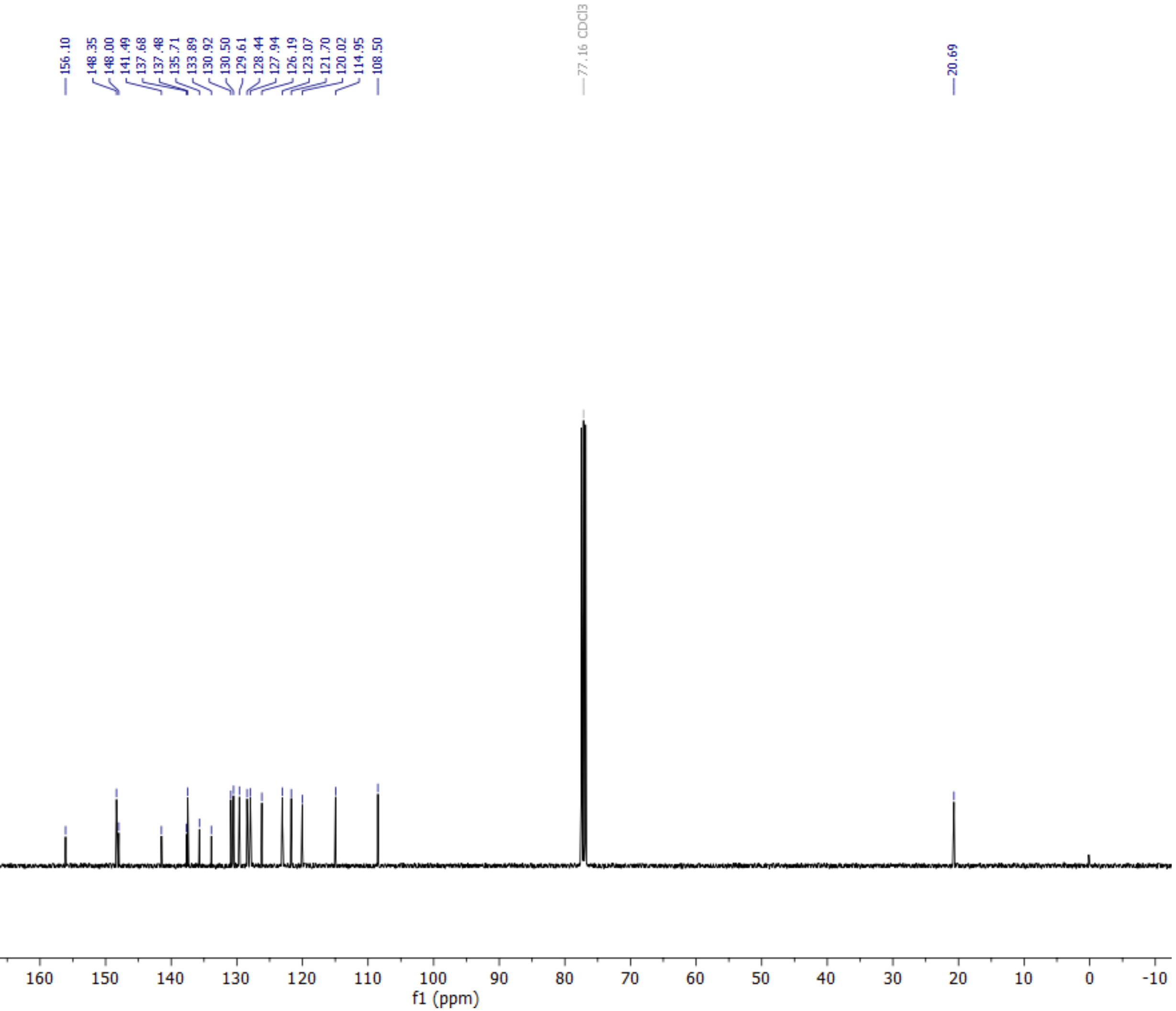

$^1H–^{13}C$ HSQC-DEPT NMR (400 MHz, $CDCl_3$) of **1u**

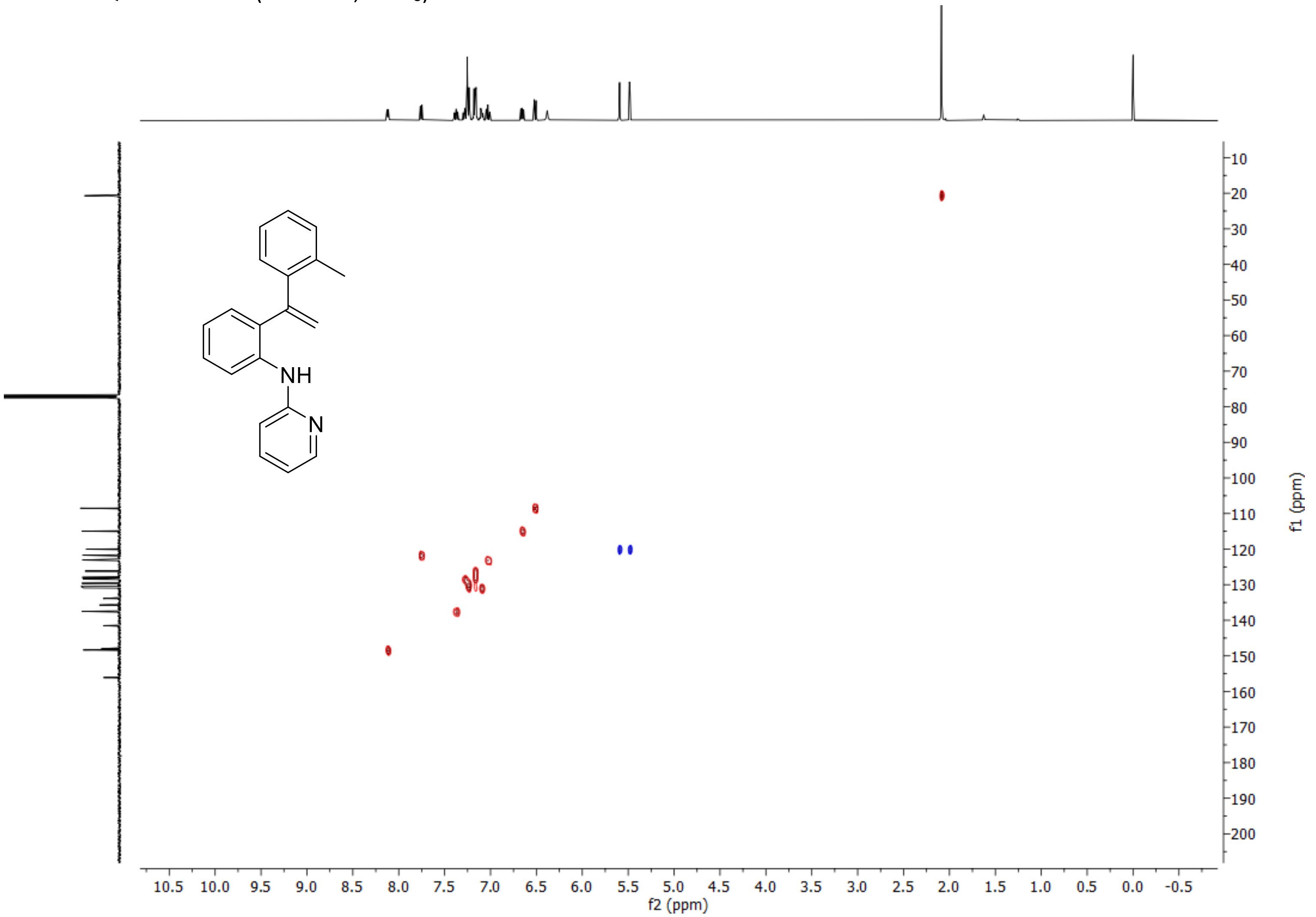

^{1}H NMR (400 MHz, $CDCl_3$) of **1v**

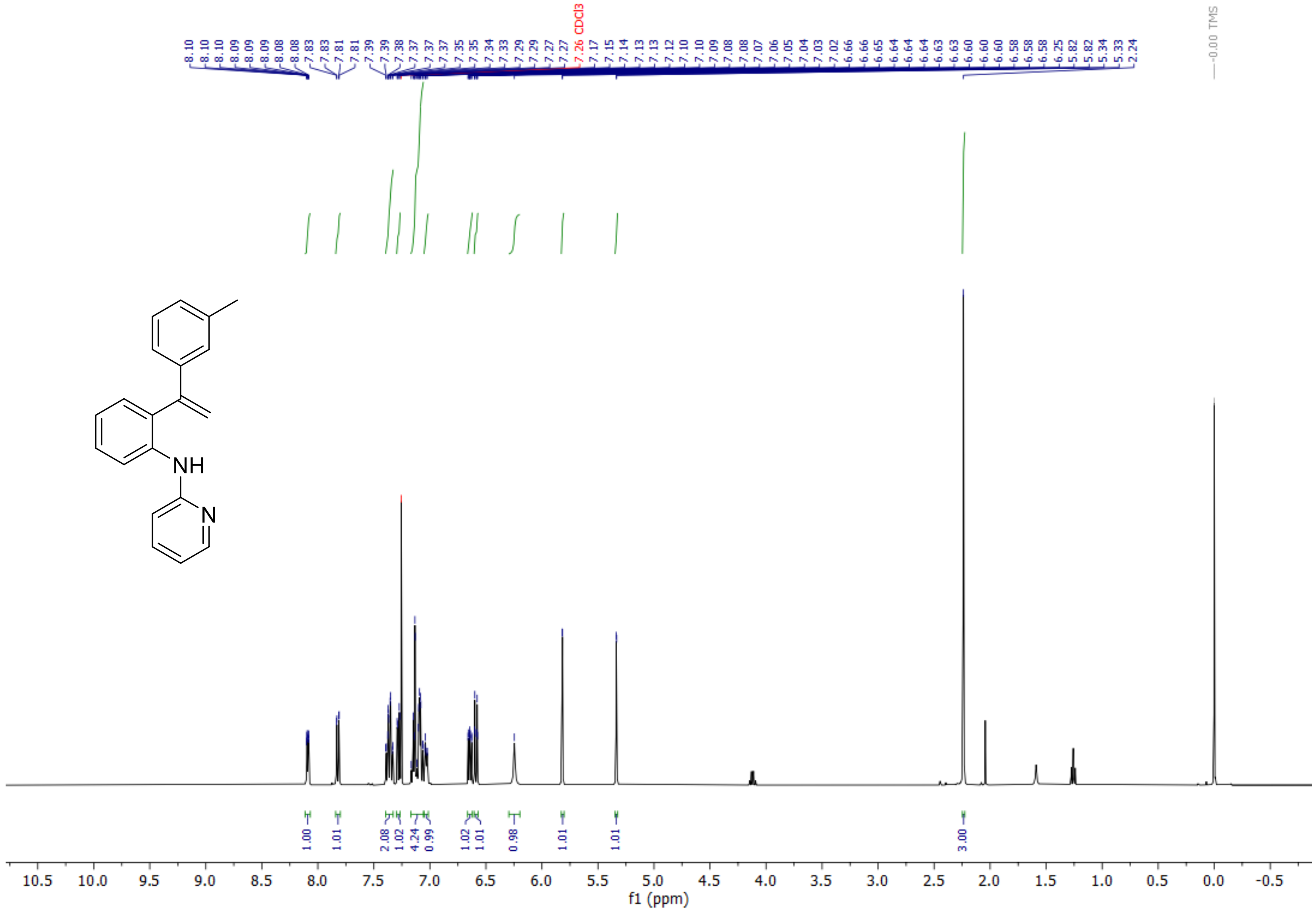

^{13}C NMR (126 MHz, $CDCl_3$) of **1v**

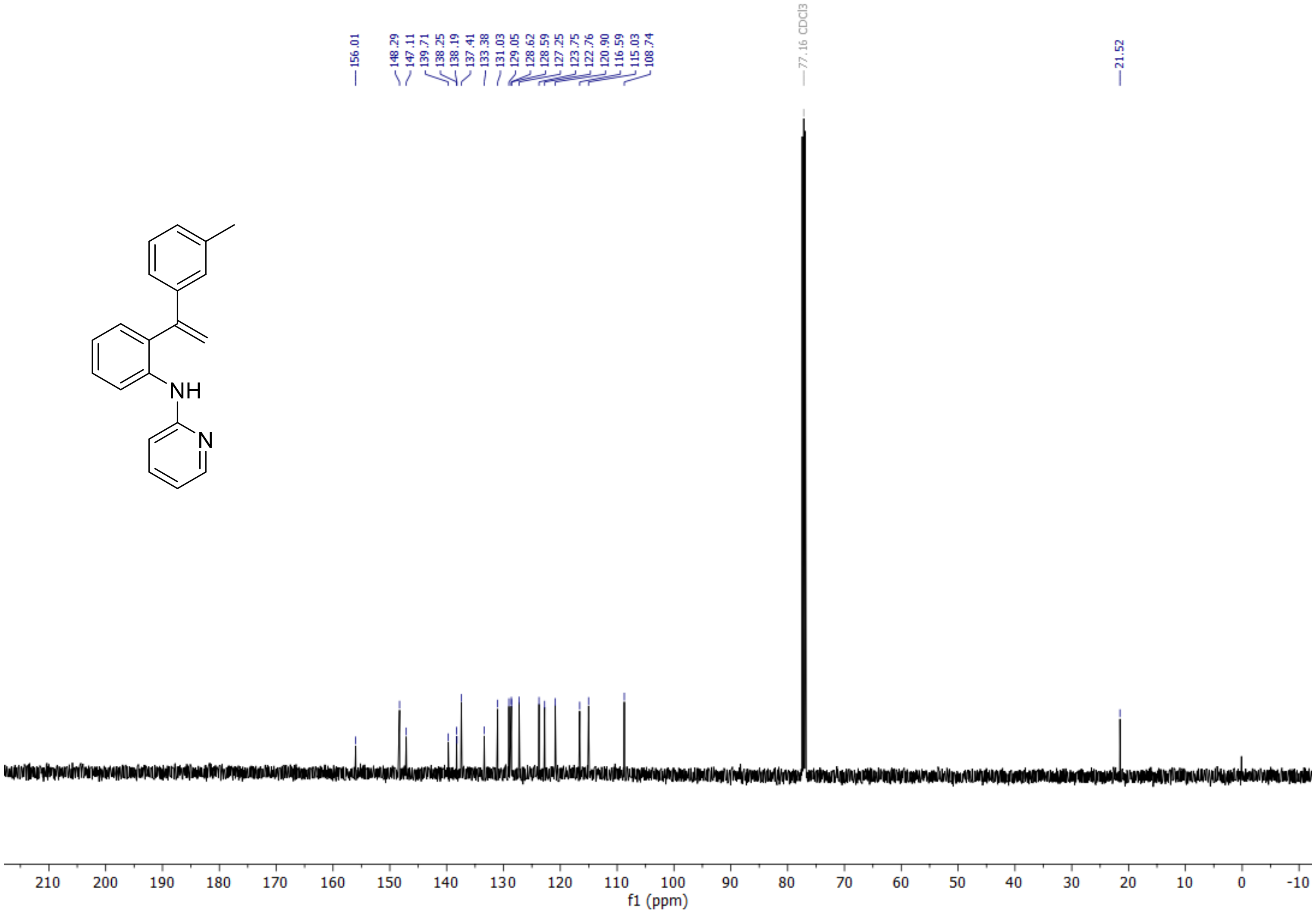

^{1}H–^{13}C HSQC-DEPT NMR (400 MHz, $CDCl_3$) of **1v**

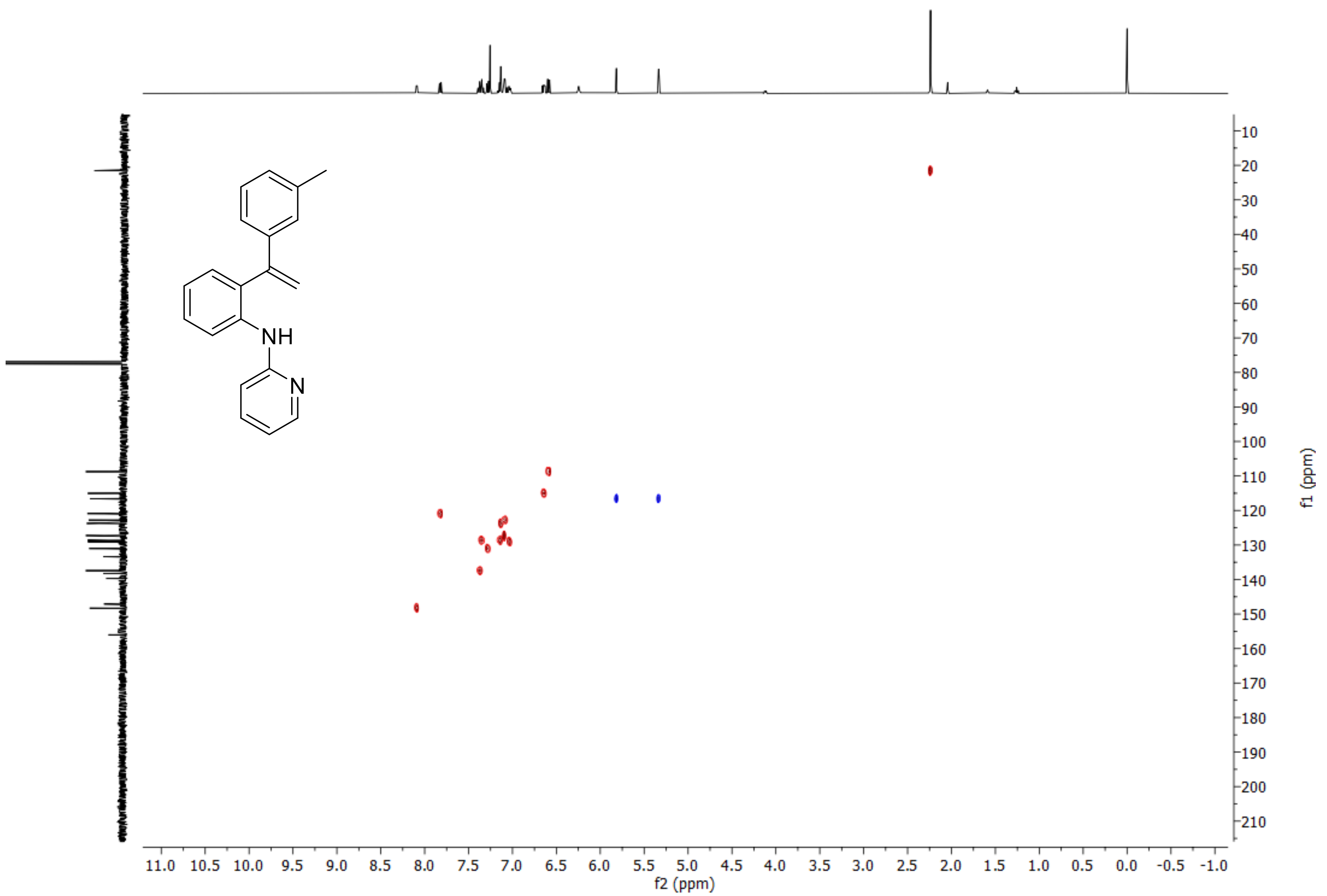

^{1}H NMR (400 MHz, $CDCl_3$) of ***cis*-1x**

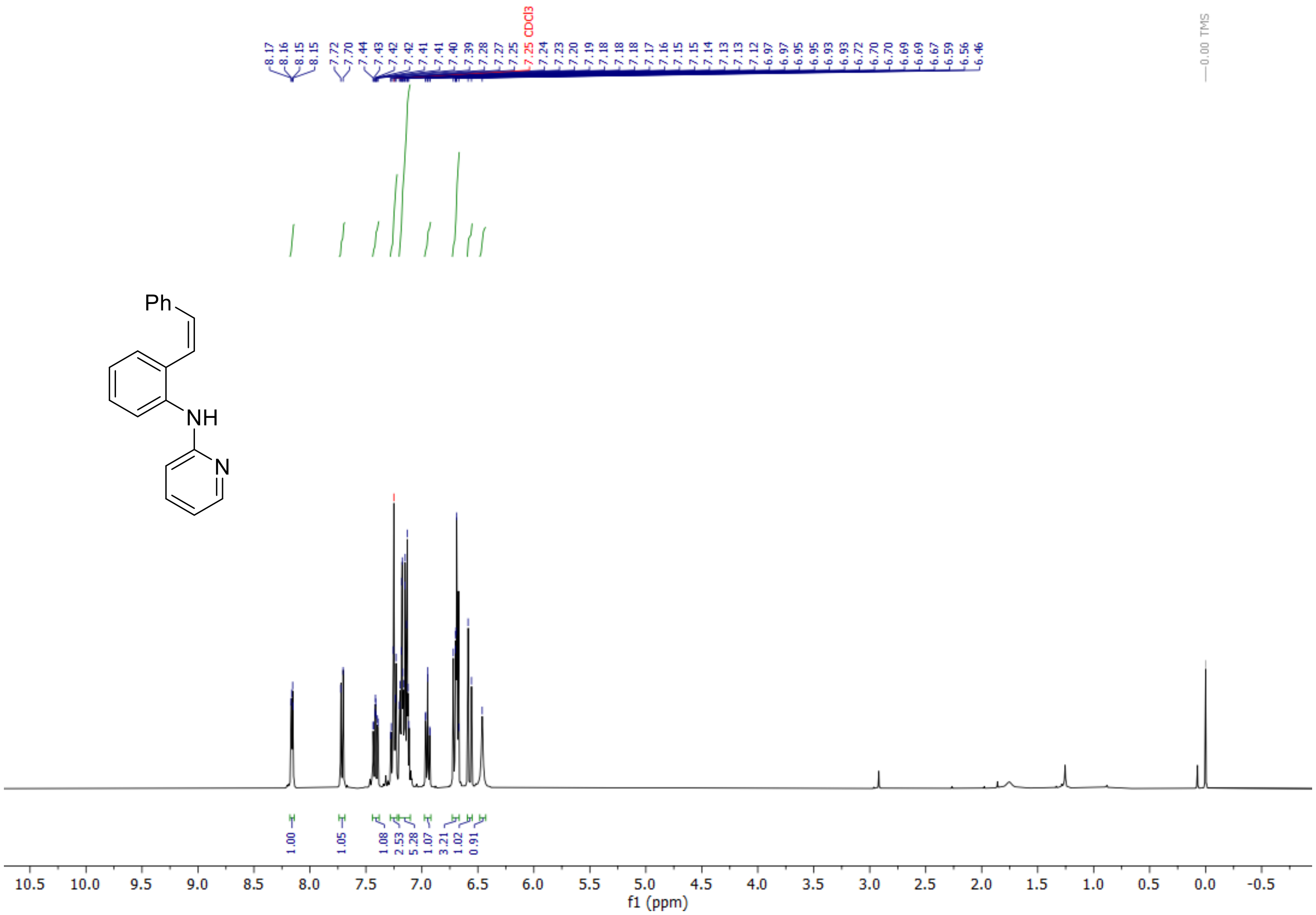

^{13}C NMR (101 MHz, $CDCl_3$) of ***cis*-1x**

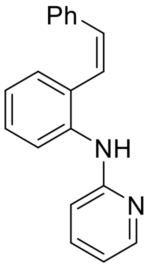

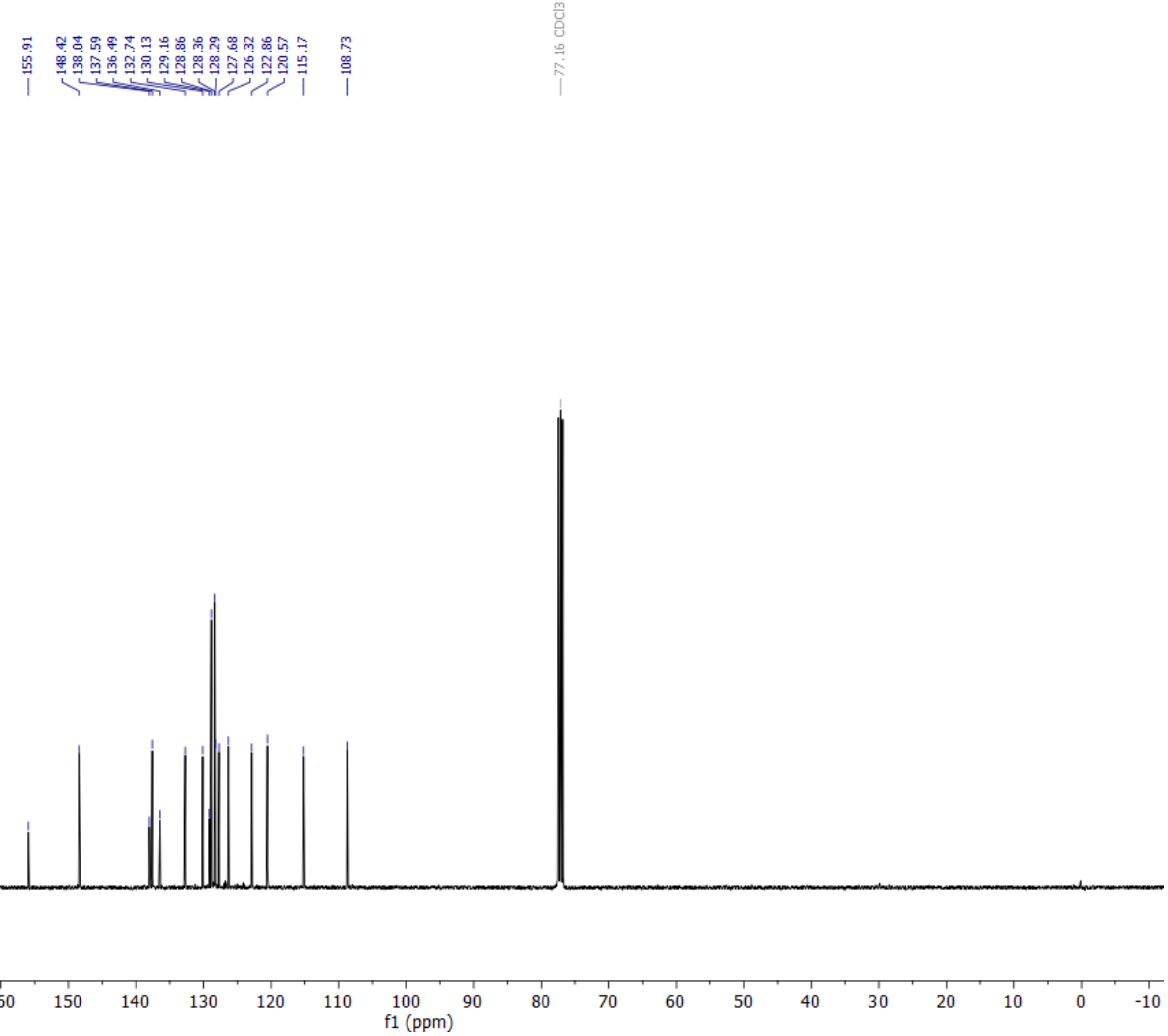

^{1}H–^{13}C HSQC-DEPT NMR (400 MHz, $CDCl_3$) of ***cis*-1x**

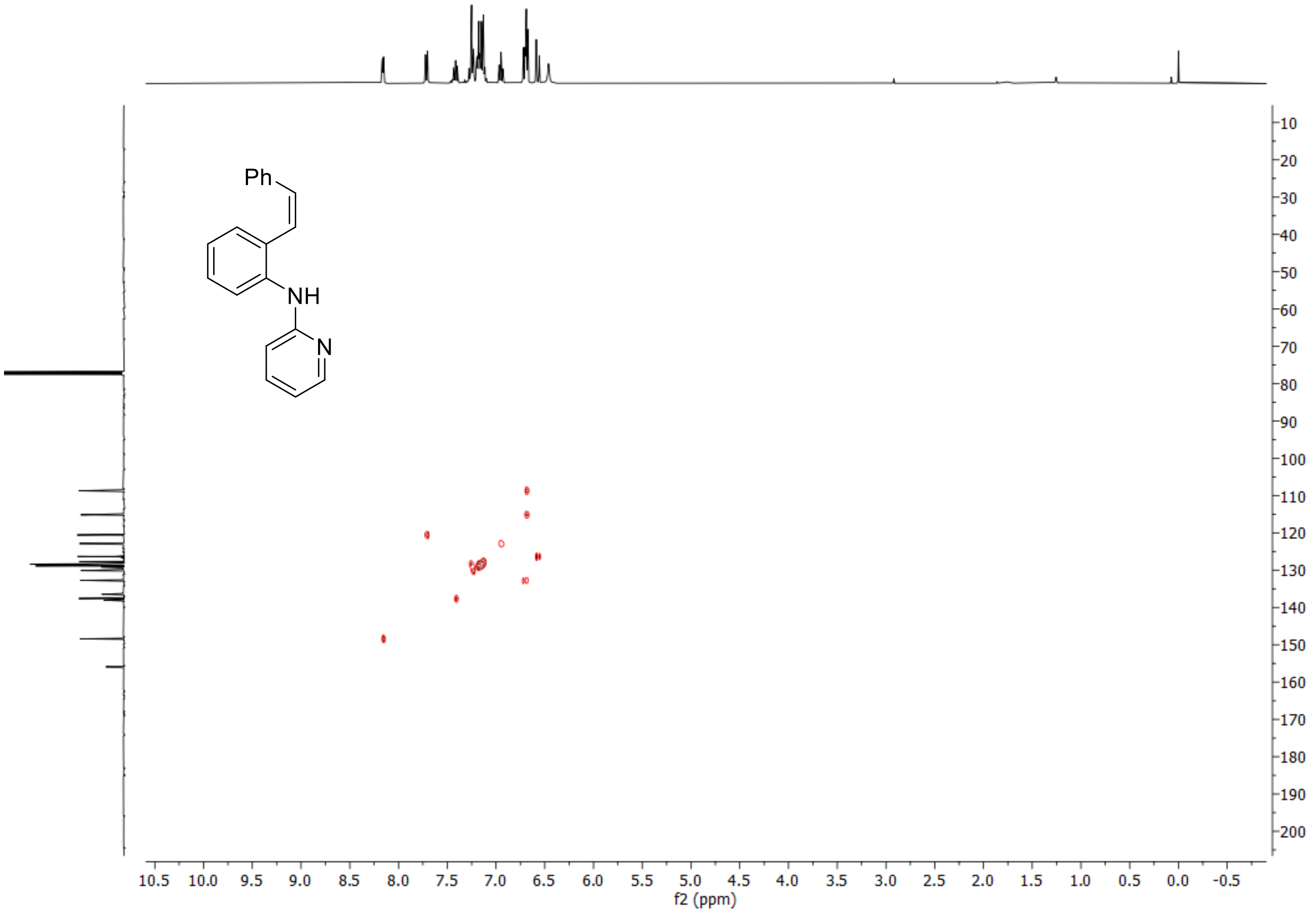

^{1}H NMR (400 MHz, $CDCl_3$) of ***trans*-1y**

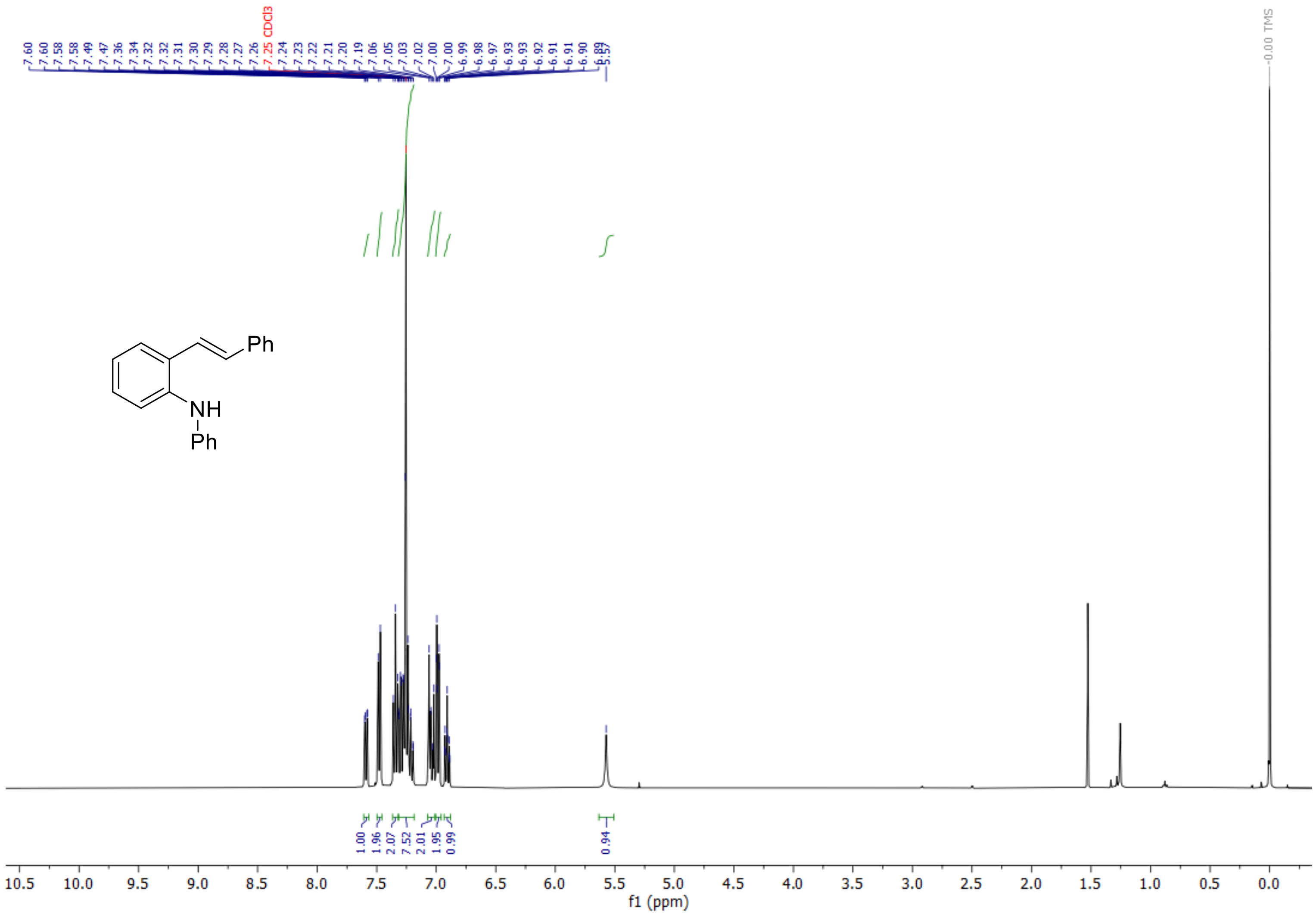

^{13}C NMR (101 MHz, $CDCl_3$) of ***trans*-1y**

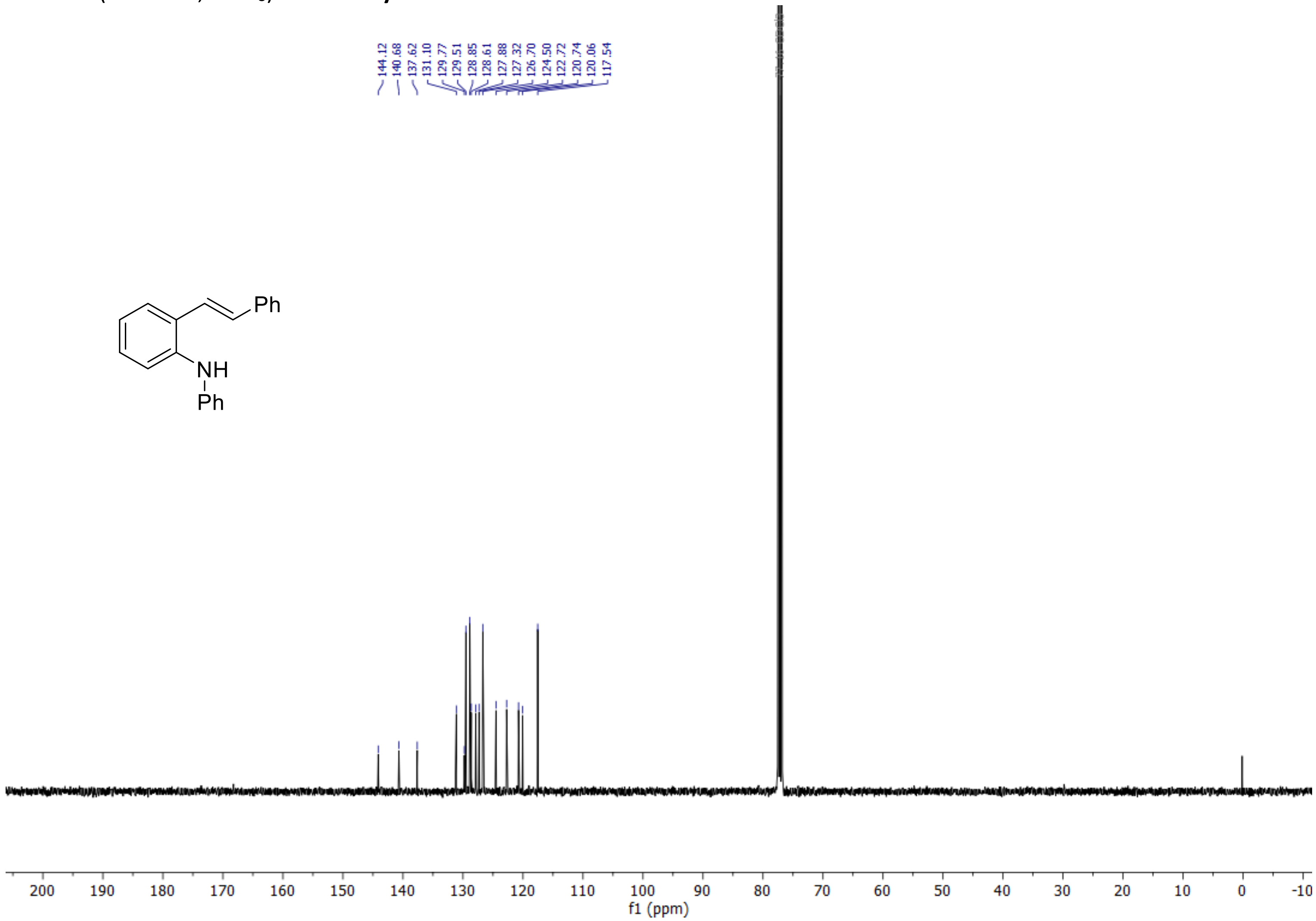

^{1}H–^{13}C HSQC-DEPT NMR (400 MHz, $CDCl_3$) of ***trans*-1y**

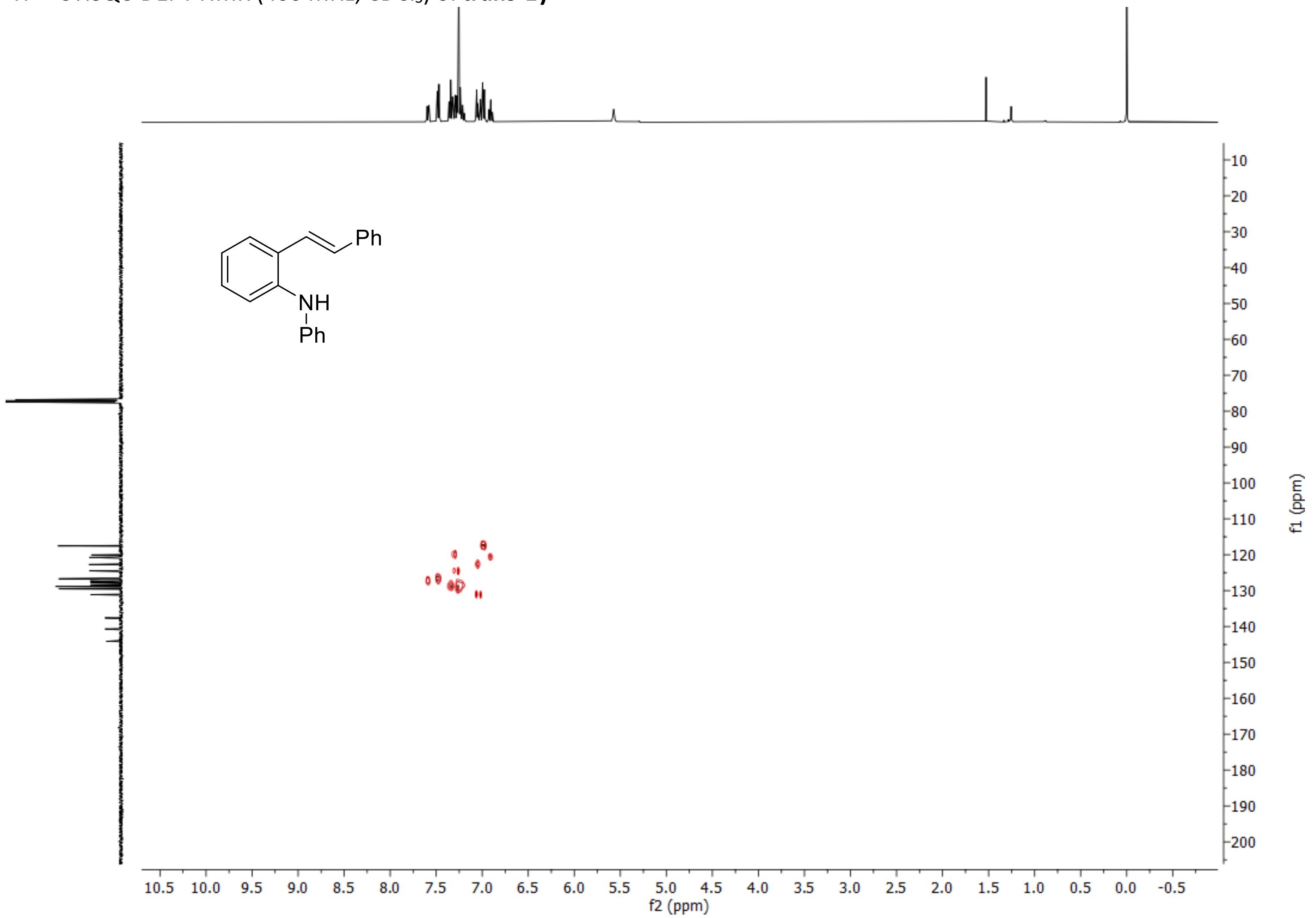

^{1}H NMR (400 MHz, $CDCl_3$) of ***cis*-1y**

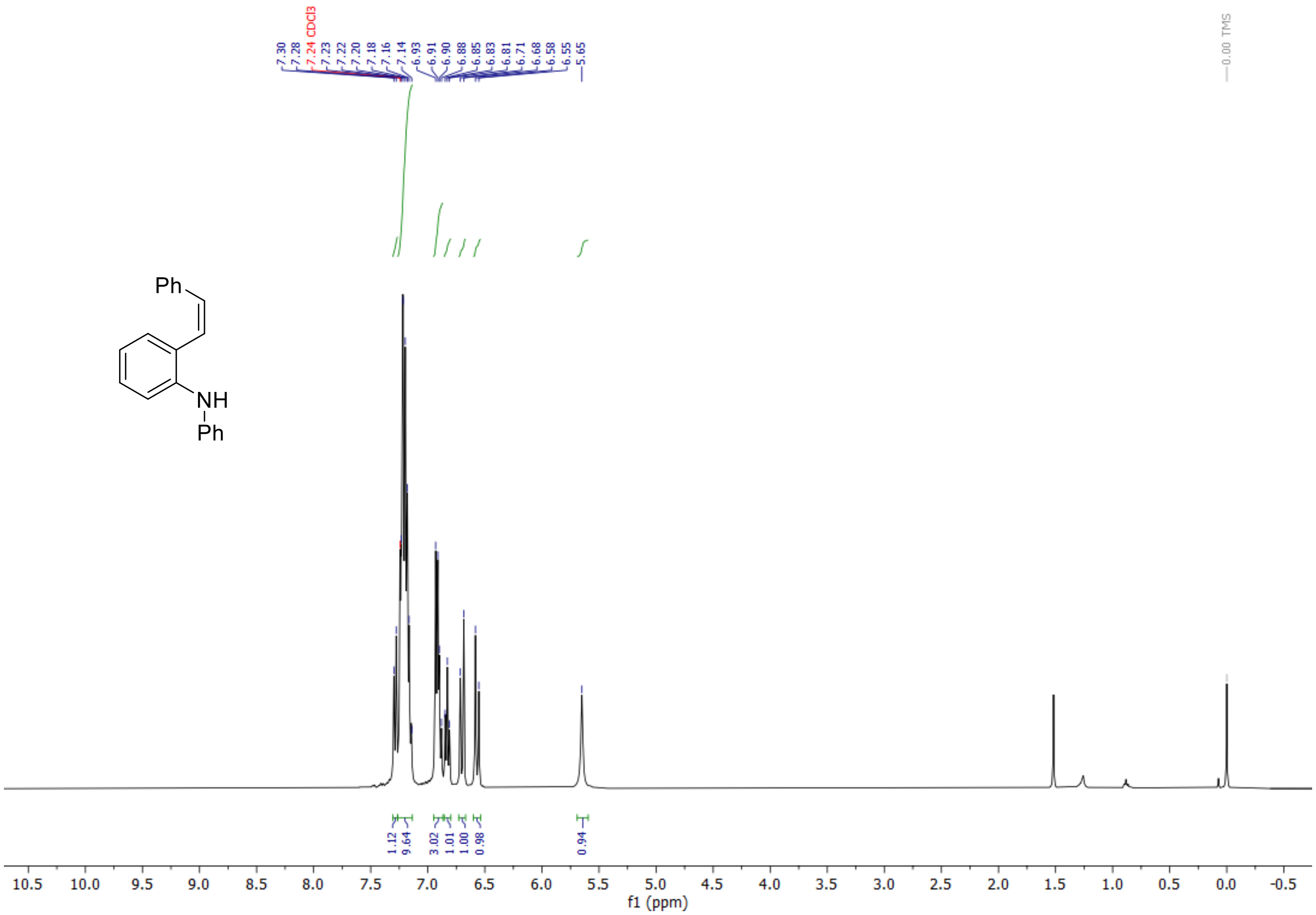

^{13}C NMR (101 MHz, $CDCl_3$) of ***cis*-1y**

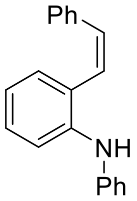

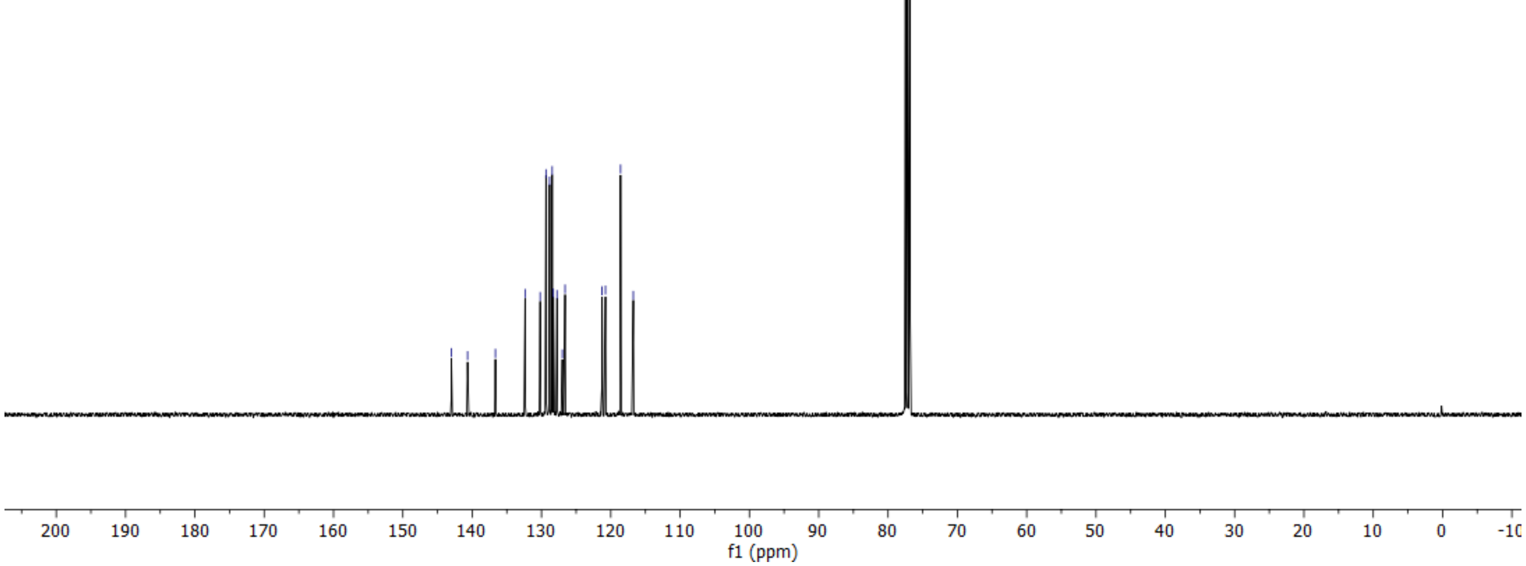

^{1}H–^{13}C HSQC-DEPT NMR (400 MHz, $CDCl_3$) of ***cis*-1y**

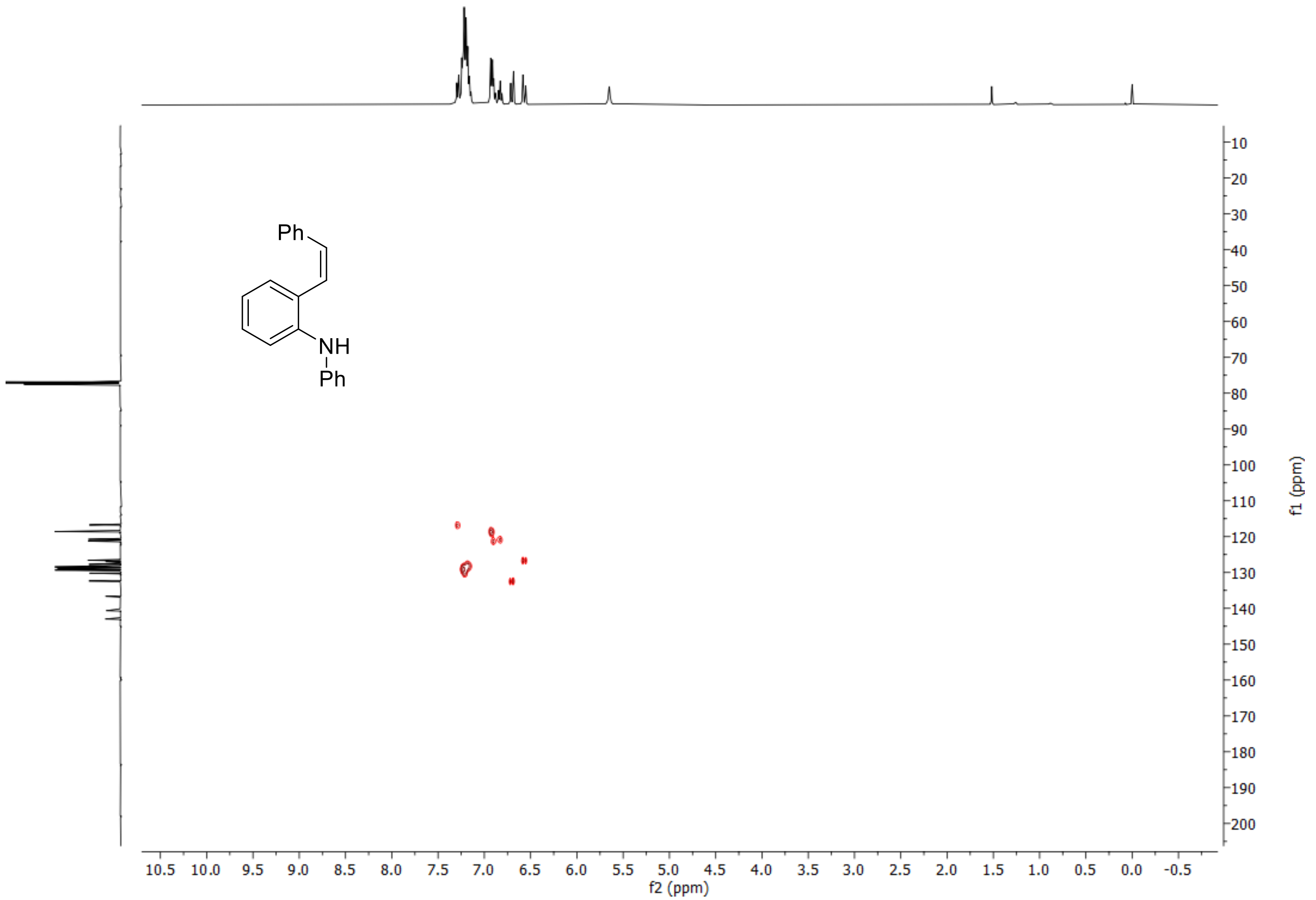

^1H NMR (400 MHz, $CDCl_3$) of **2a**

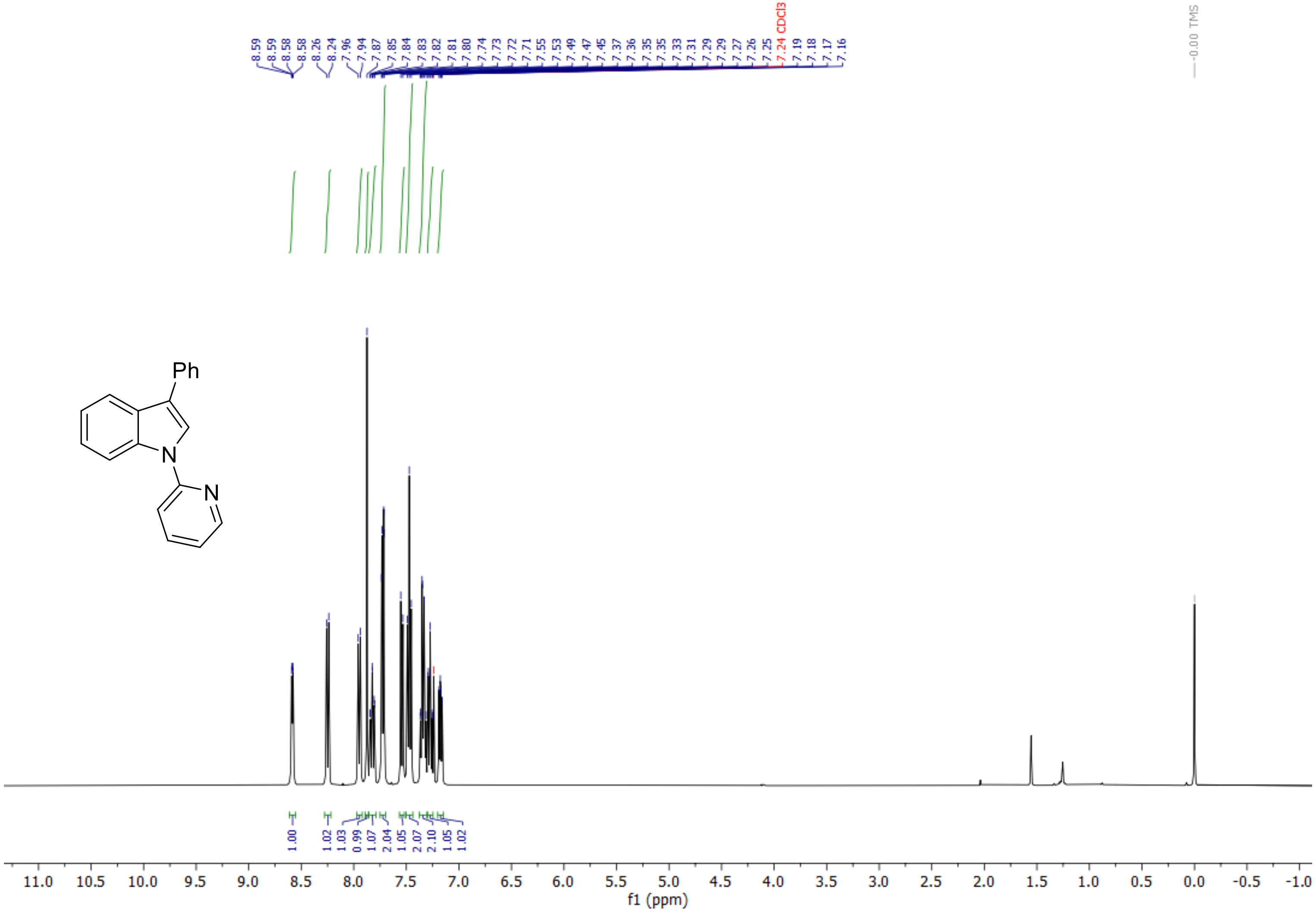

^{1}H NMR (400 MHz, $CDCl_3$) of **2b**

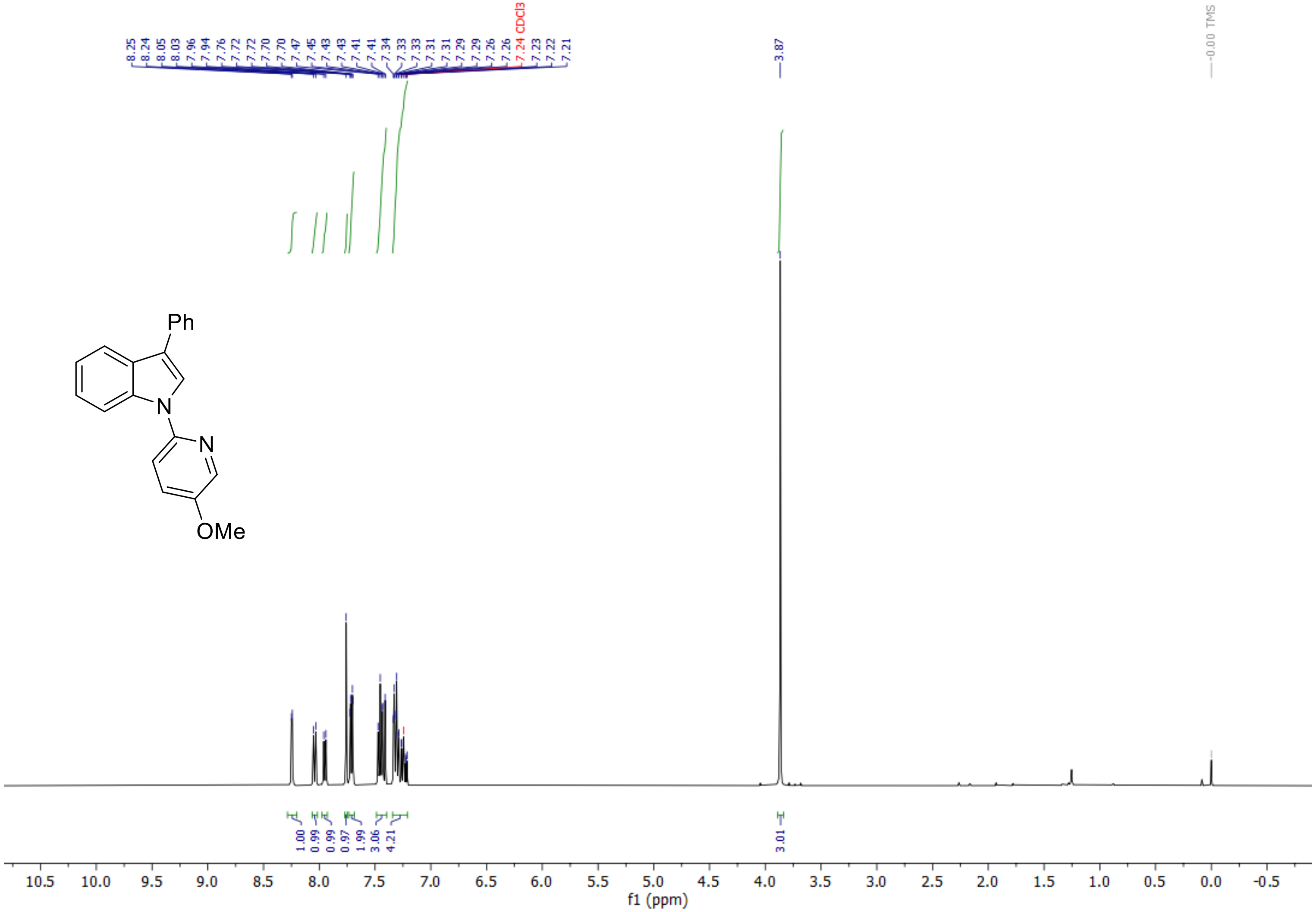

^{13}C NMR (101 MHz, $CDCl_3$) of **2b**

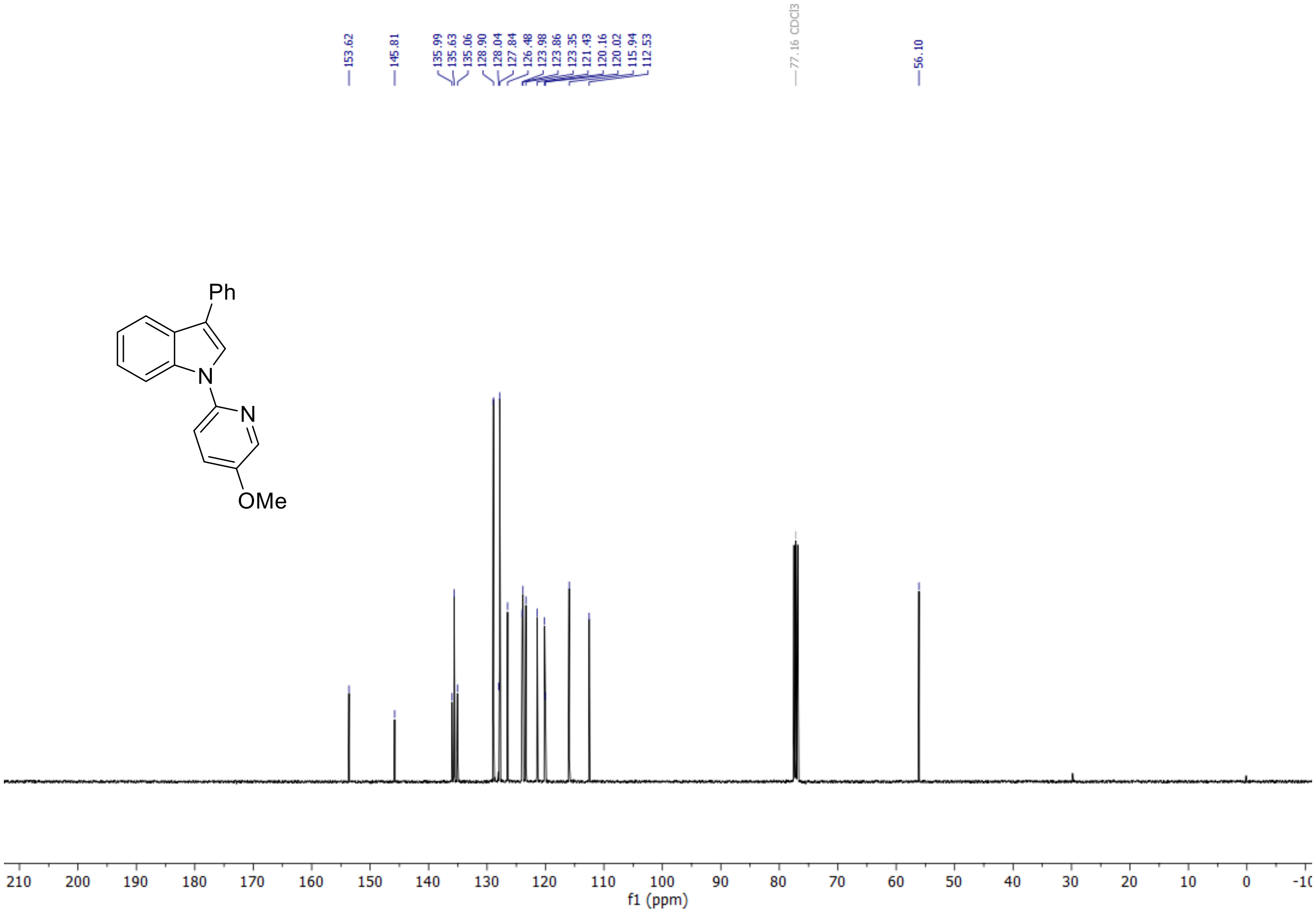

^1H–^{13}C HSQC-DEPT NMR (400 MHz, $CDCl_3$) of **2b**

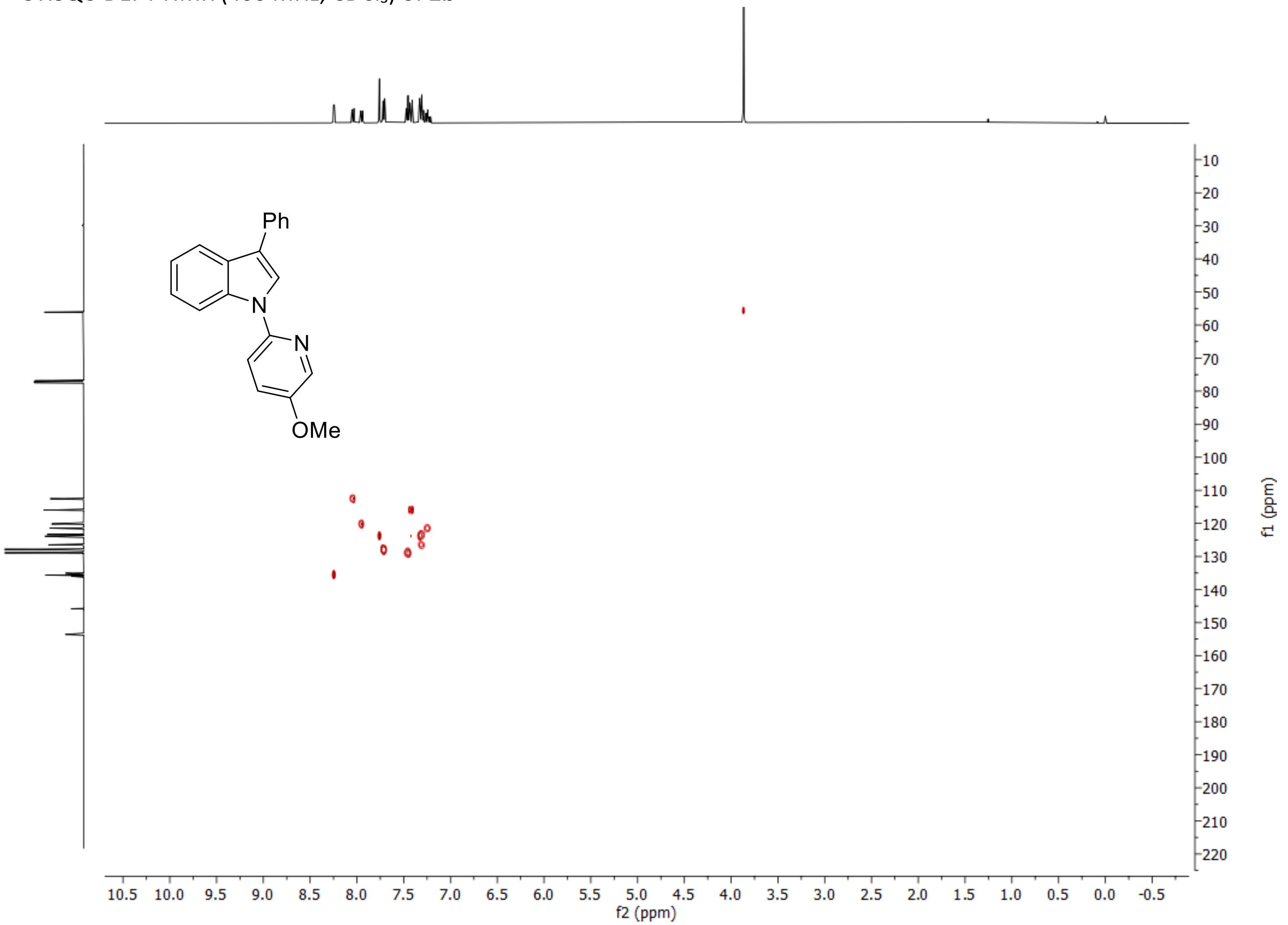

^1H NMR (400 MHz, $CDCl_3$) of **2c**

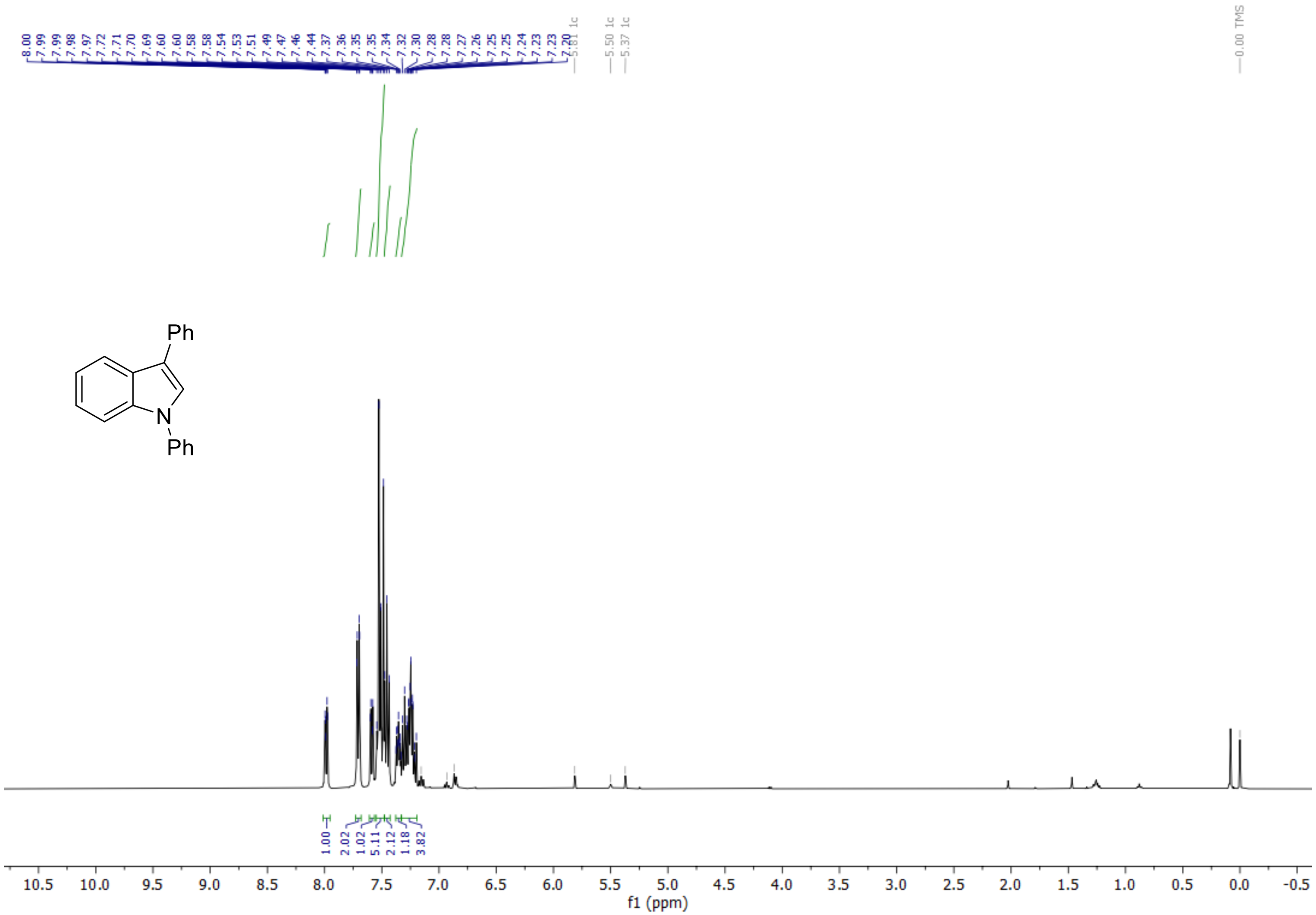

^{1}H NMR (400 MHz, $CDCl_3$) of **2d**

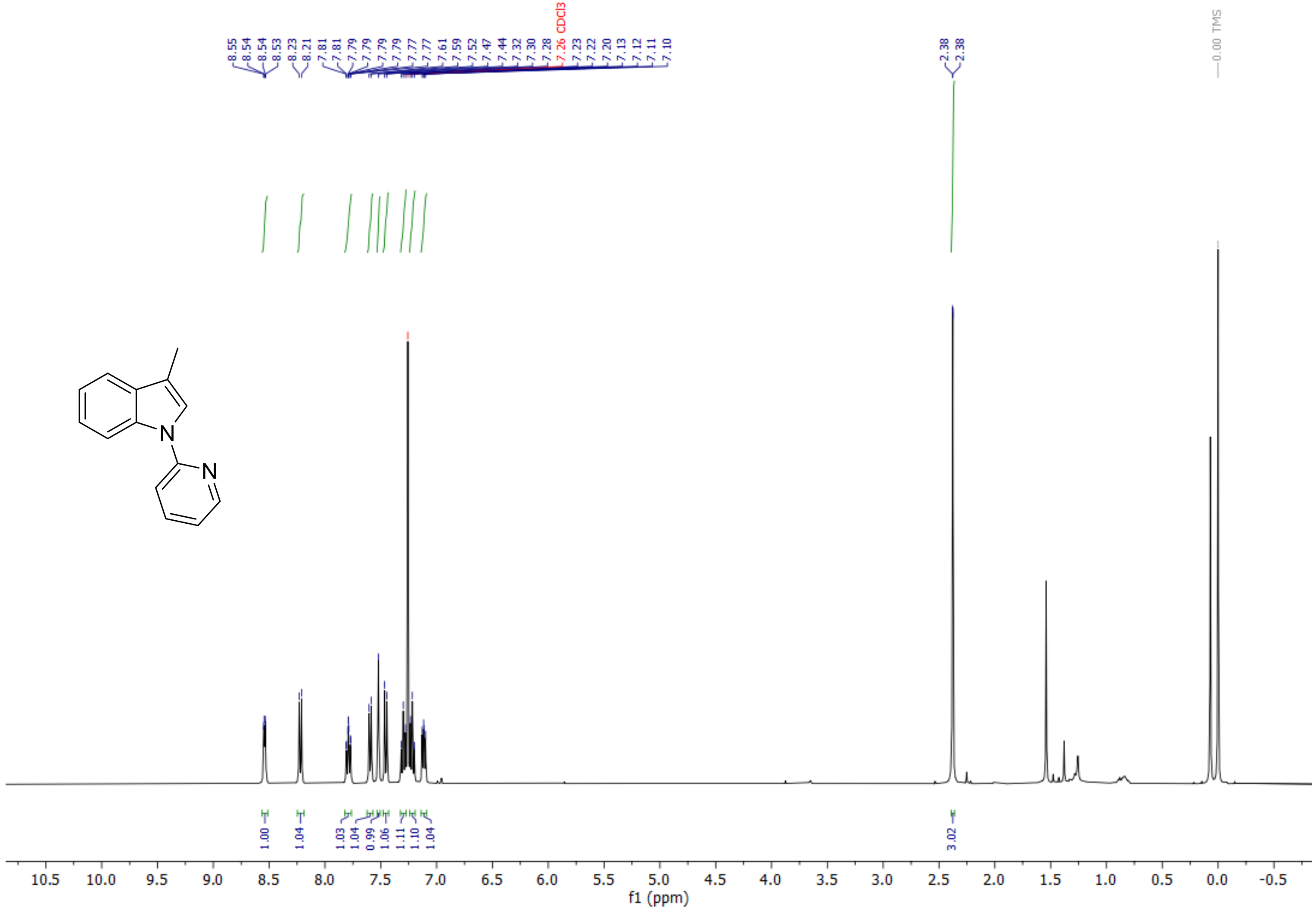

^{1}H NMR (400 MHz, $CDCl_3$) of **2e**

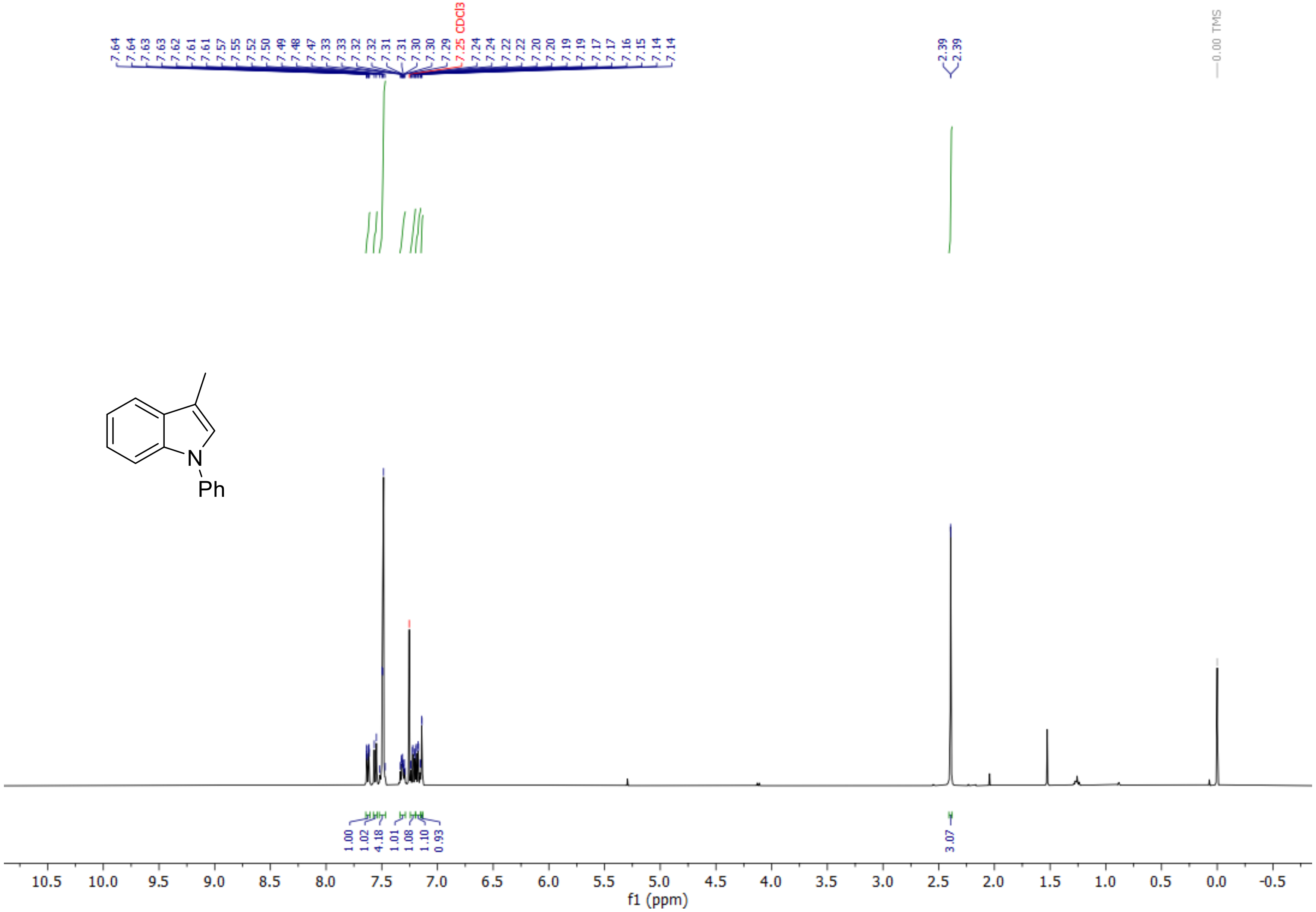

^{1}H NMR (400 MHz, $CDCl_3$) of **2f**

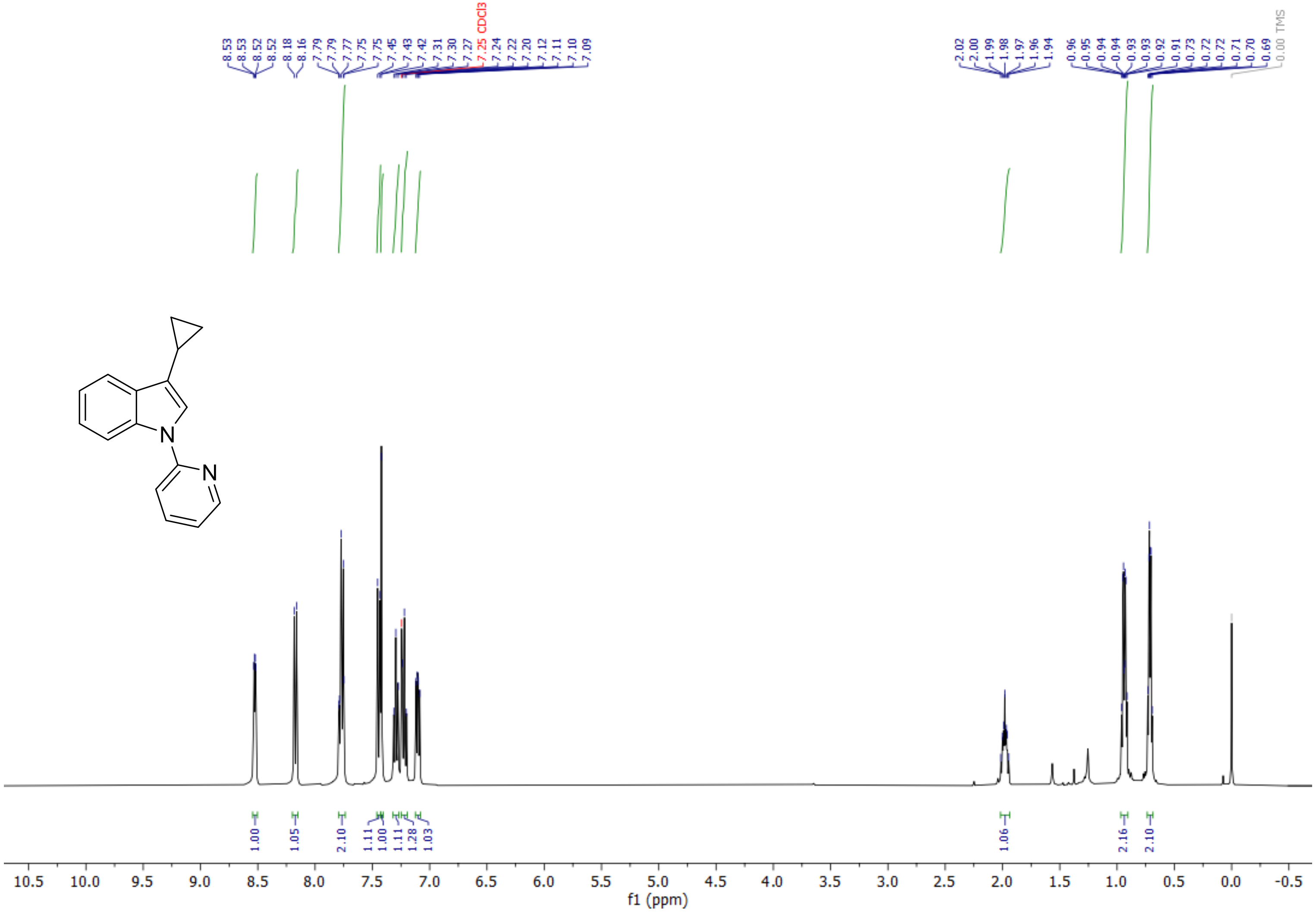

^{13}C NMR (101 MHz, $CDCl_3$) of **2f**

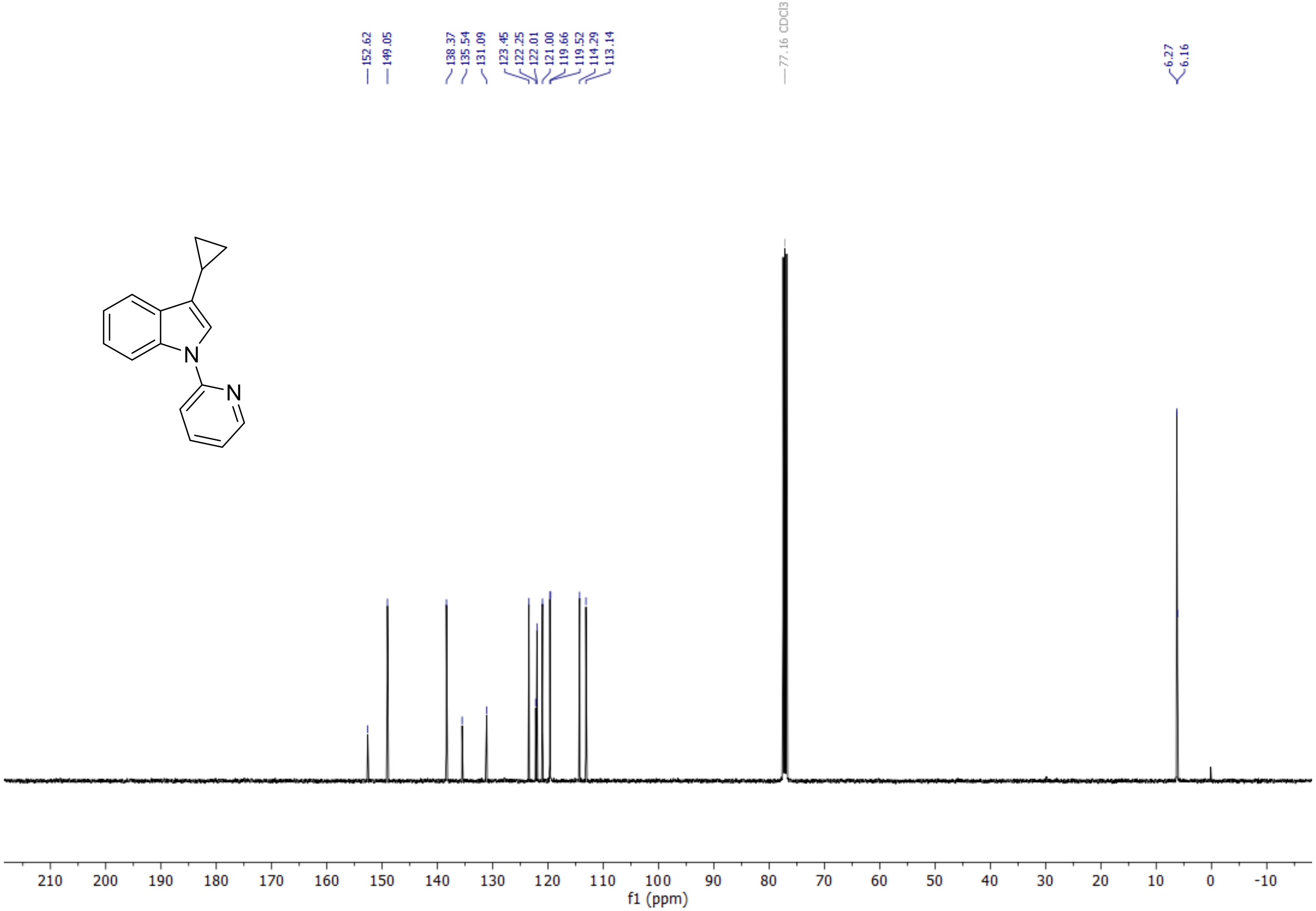

^1H–^{13}C HSQC-DEPT NMR (400 MHz, $CDCl_3$) of **2f**

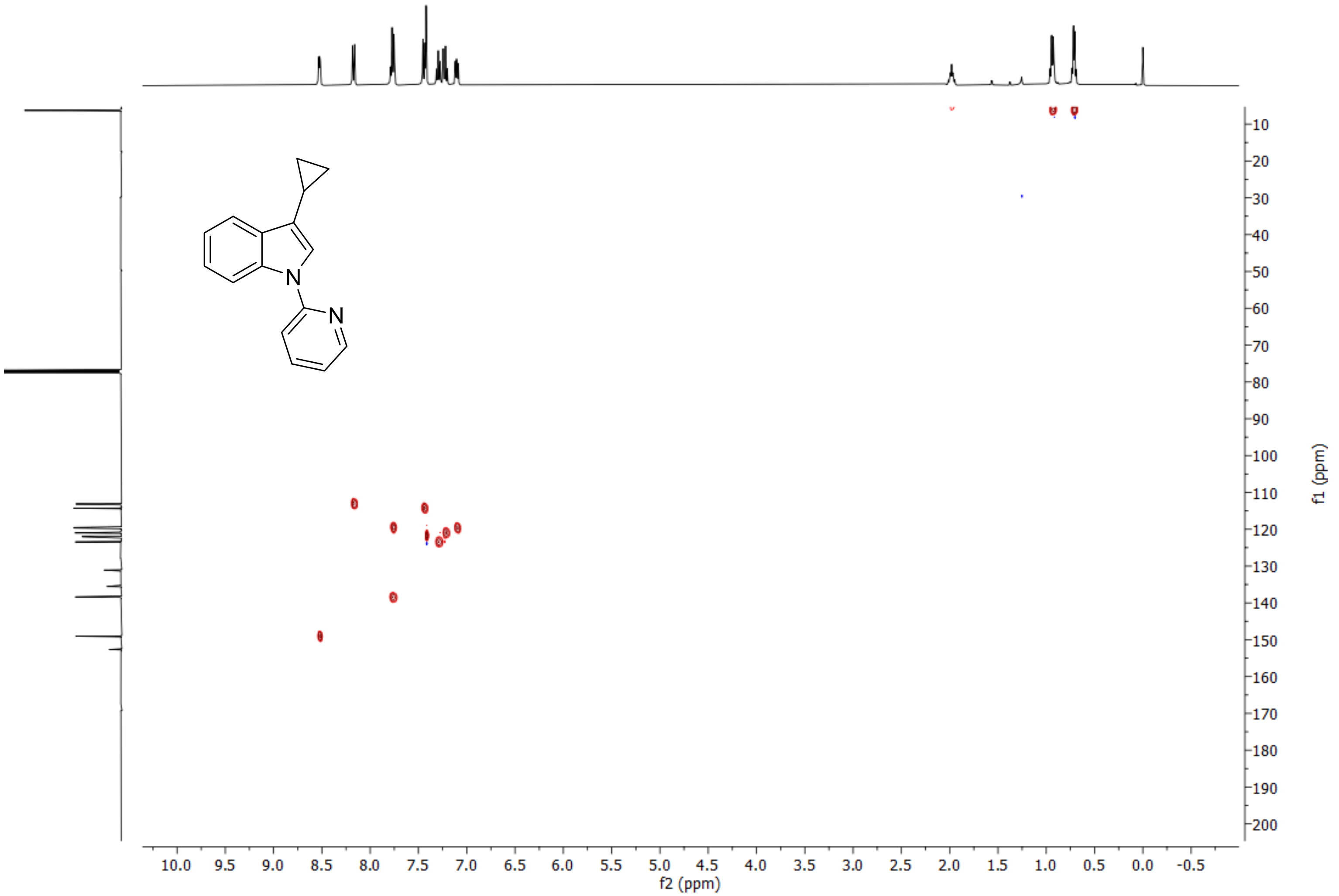

^{1}H NMR (400 MHz, $CDCl_3$) of **2g**

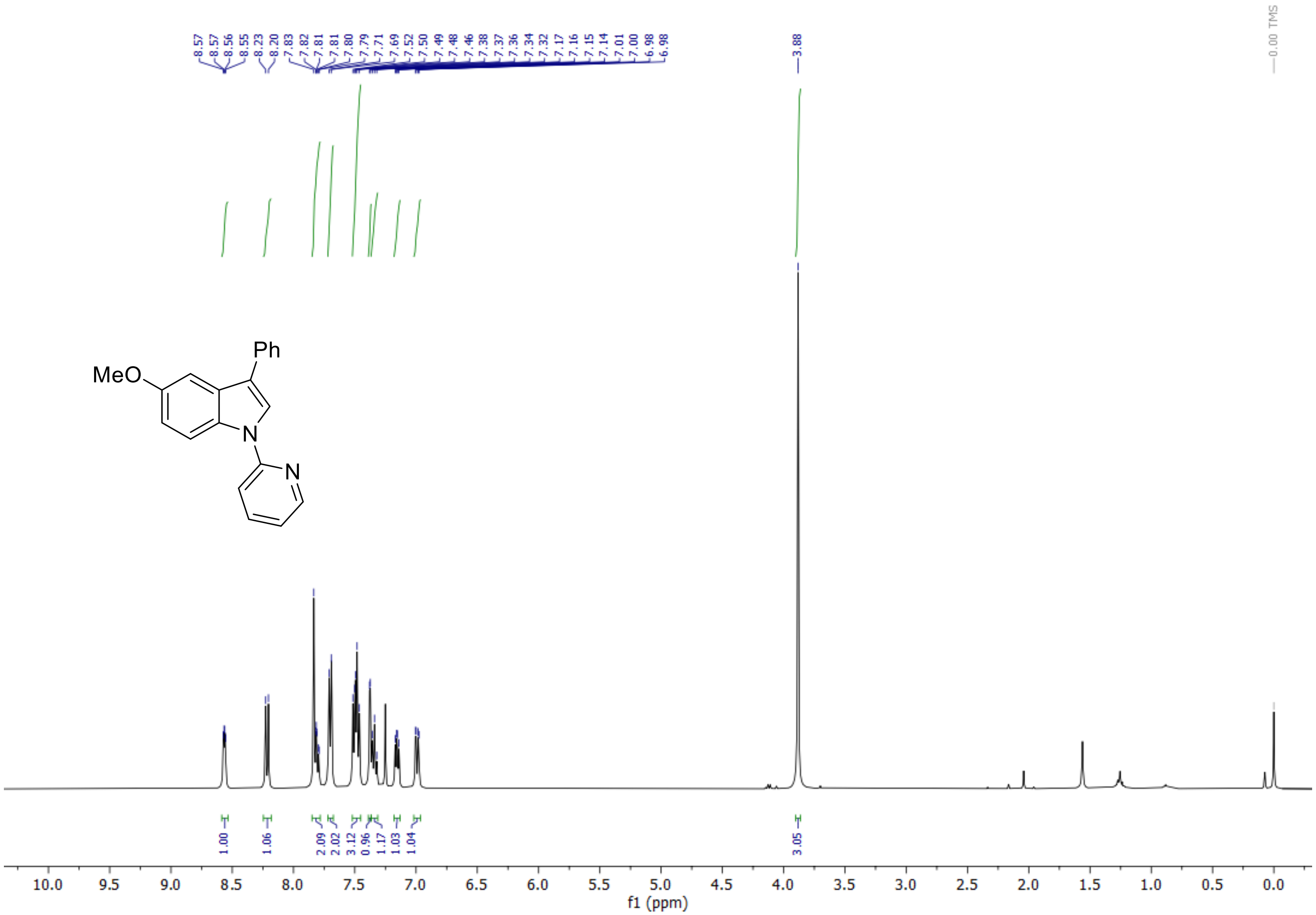

^{13}C NMR (101 MHz, CDCl₃) of **2g**

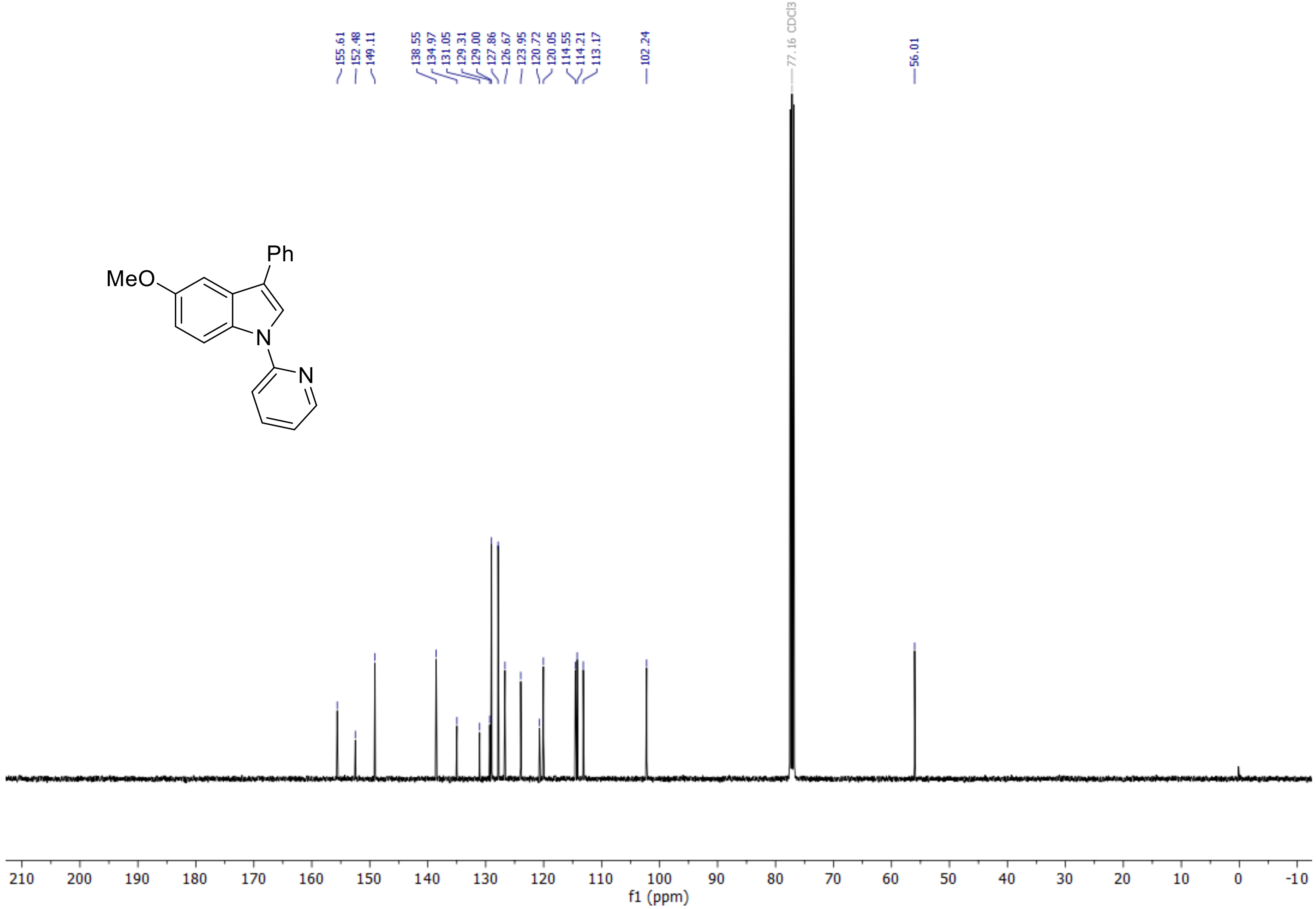

^{1}H–^{13}C HSQC-DEPT NMR (400 MHz, $CDCl_3$) of **2g**

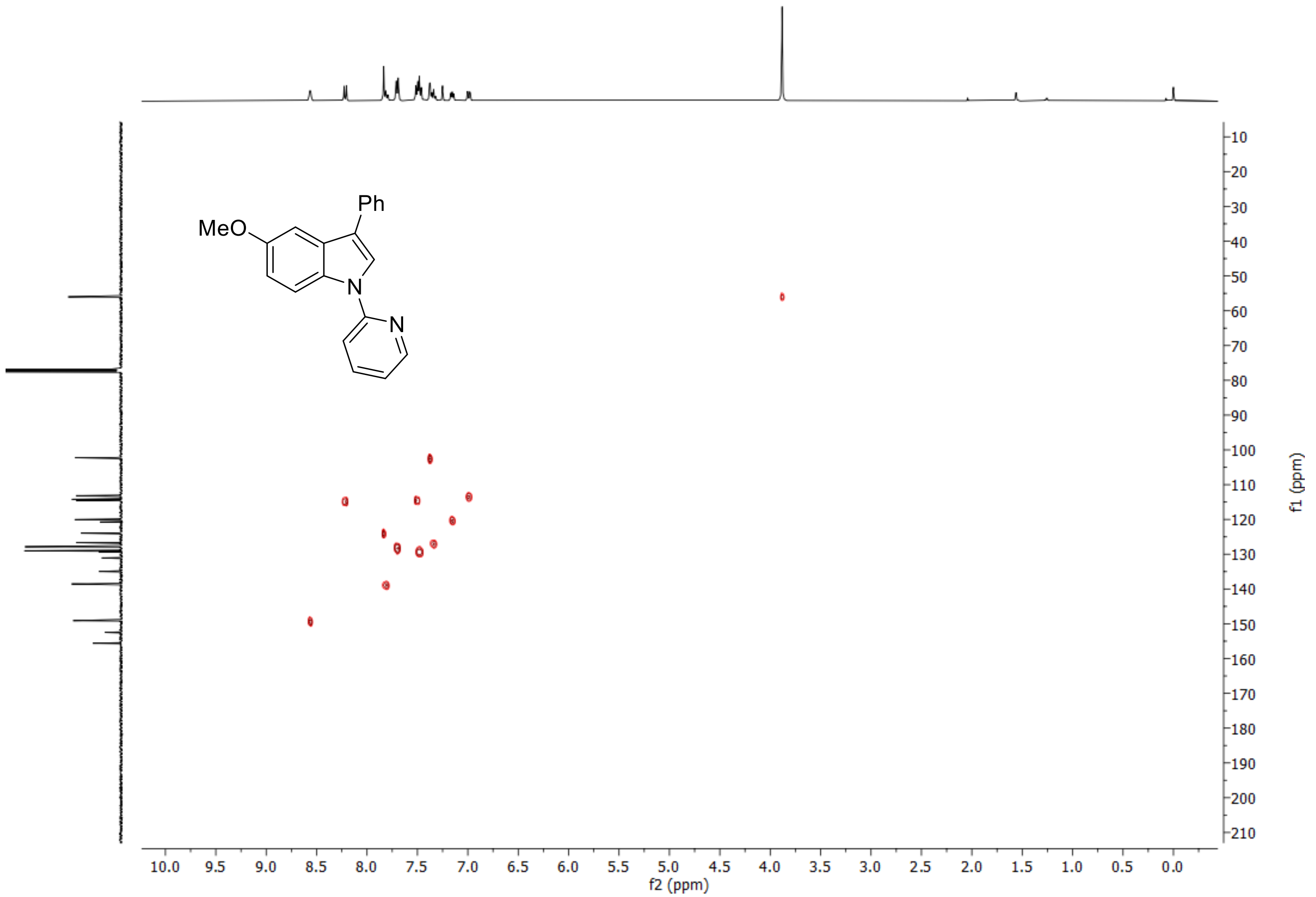

^{1}H NMR (400 MHz, $CDCl_3$) of **2h**

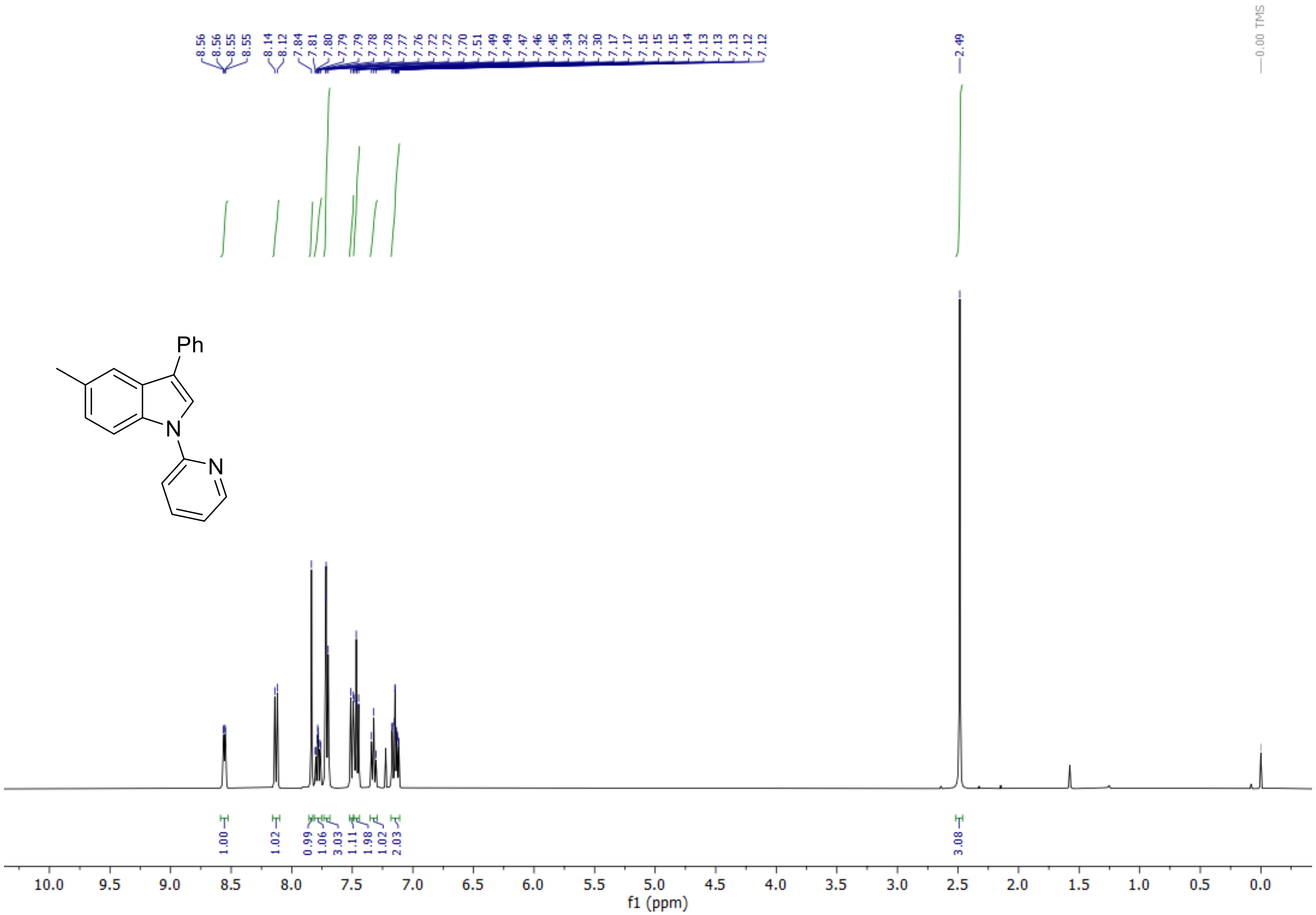

^{13}C NMR (101 MHz, $CDCl_3$) of **2h**

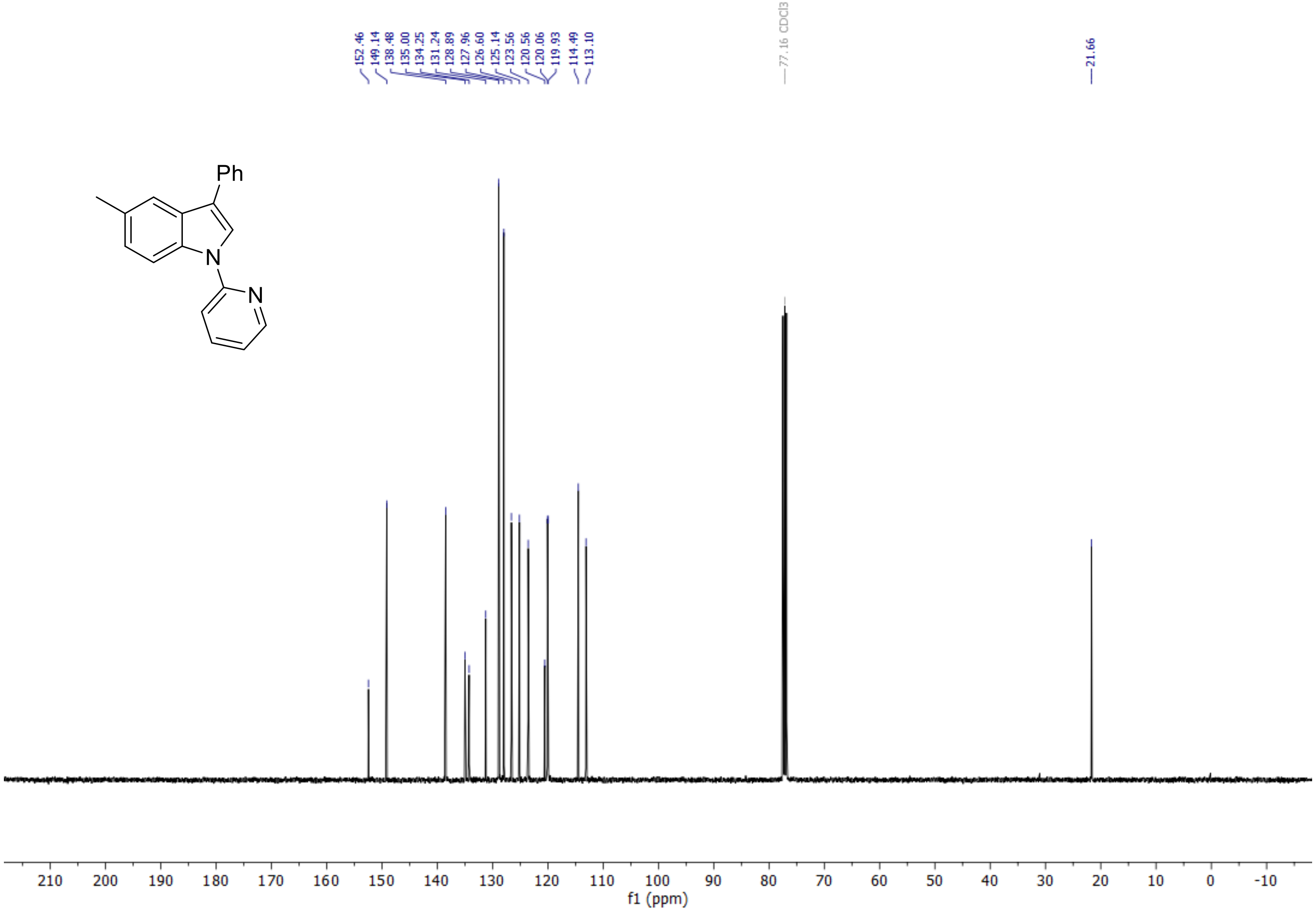

^{1}H–^{13}C HSQC-DEPT NMR (400 MHz, $CDCl_3$) of **2h**

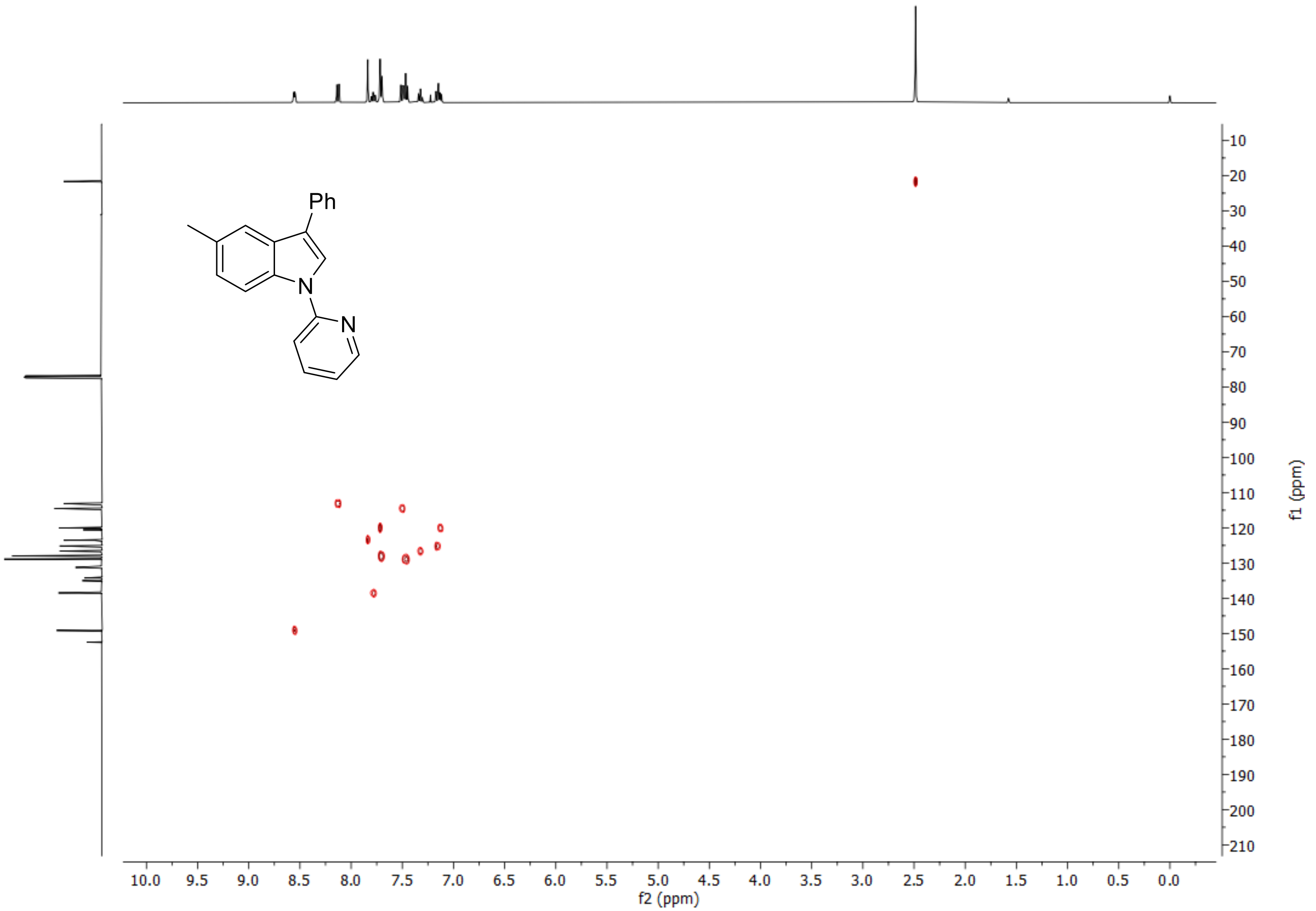

^{1}H NMR (400 MHz, $CDCl_3$) of **2i**

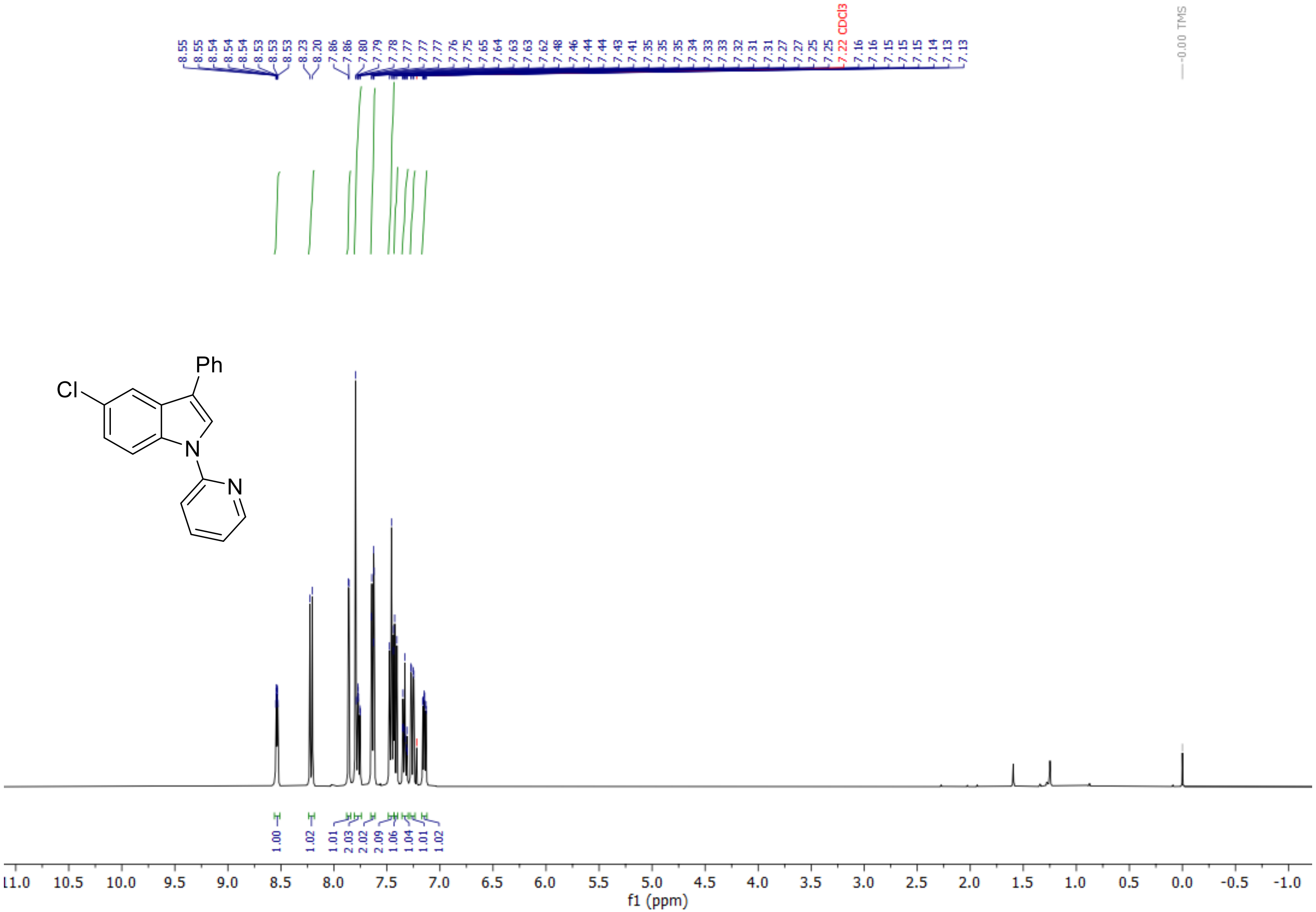

^{13}C NMR (101 MHz, $CDCl_3$) of **2i**

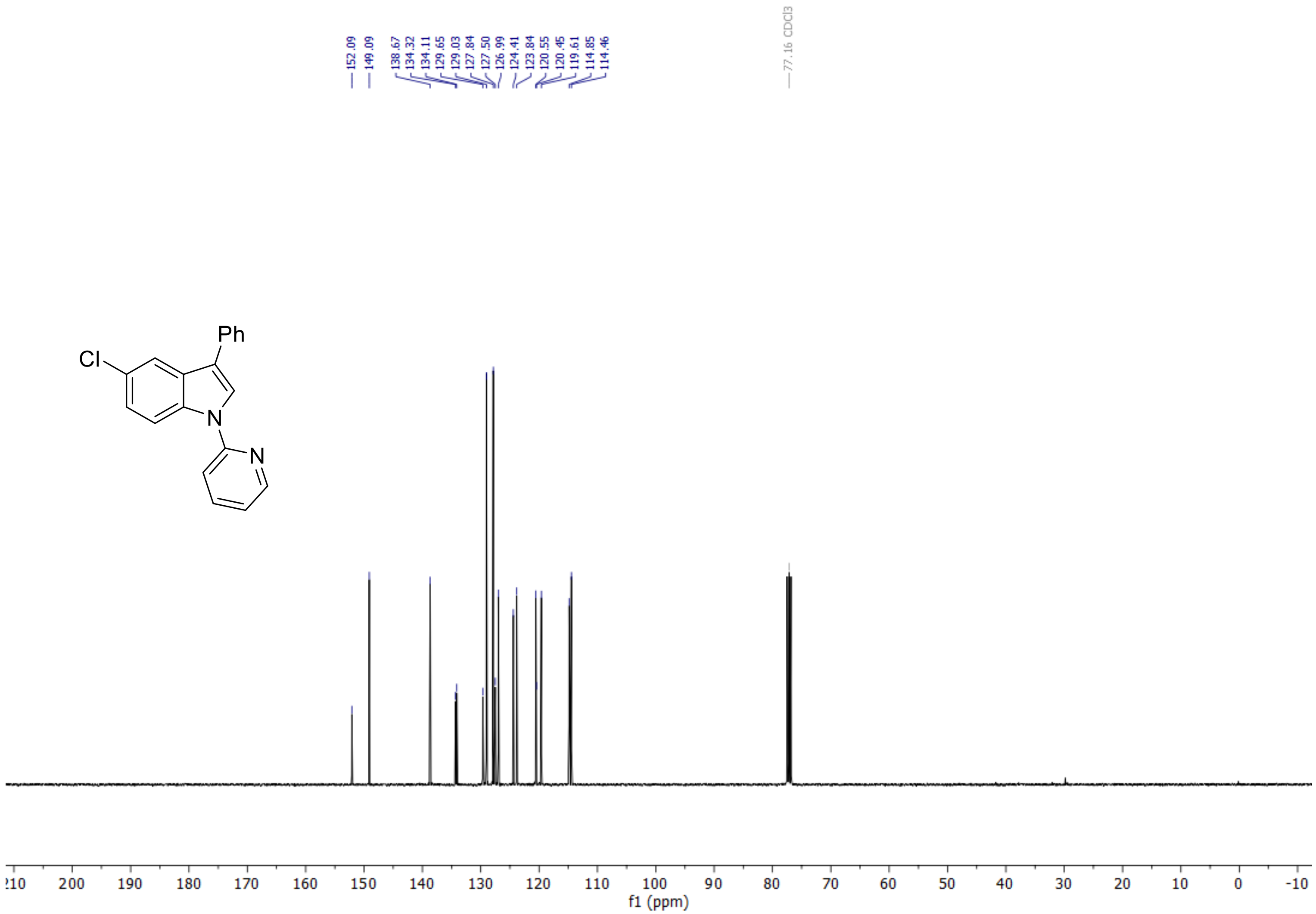

$^1H–^{13}C$ HSQC-DEPT NMR (400 MHz, $CDCl_3$) of **2i**

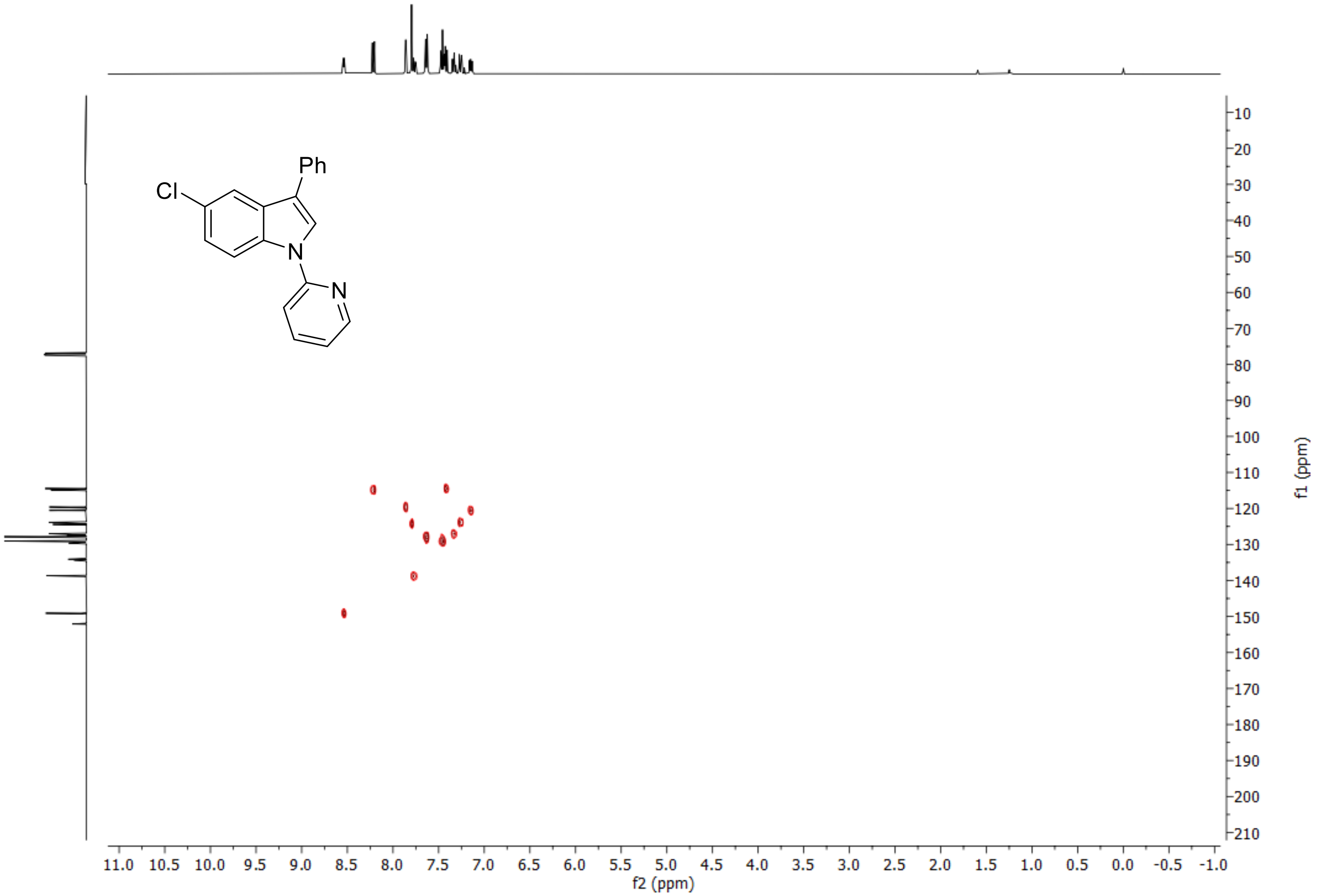

^{1}H NMR (400 MHz, $CDCl_3$) of **2j**

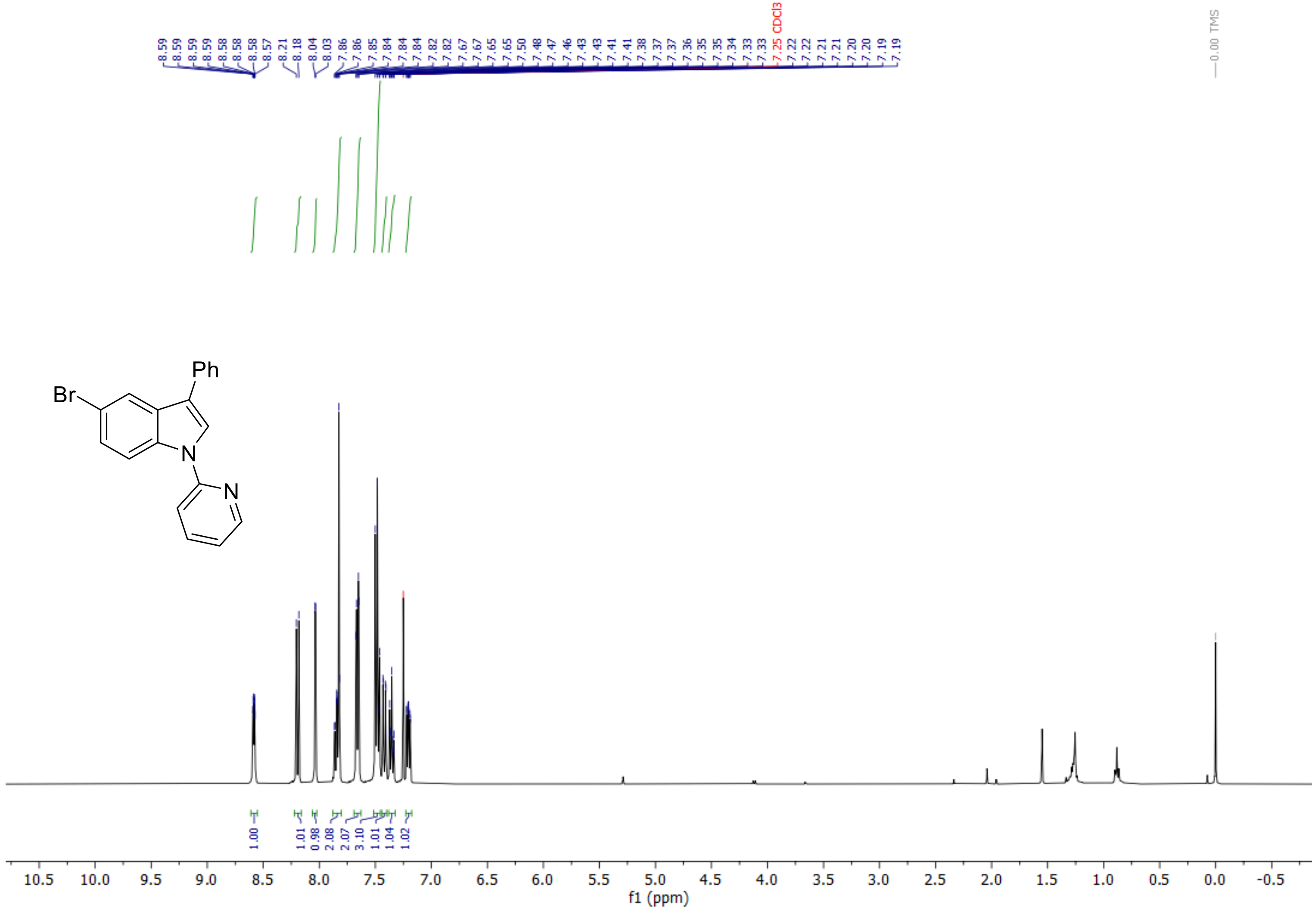

^{13}C NMR (126 MHz, $CDCl_3$) of **2j**

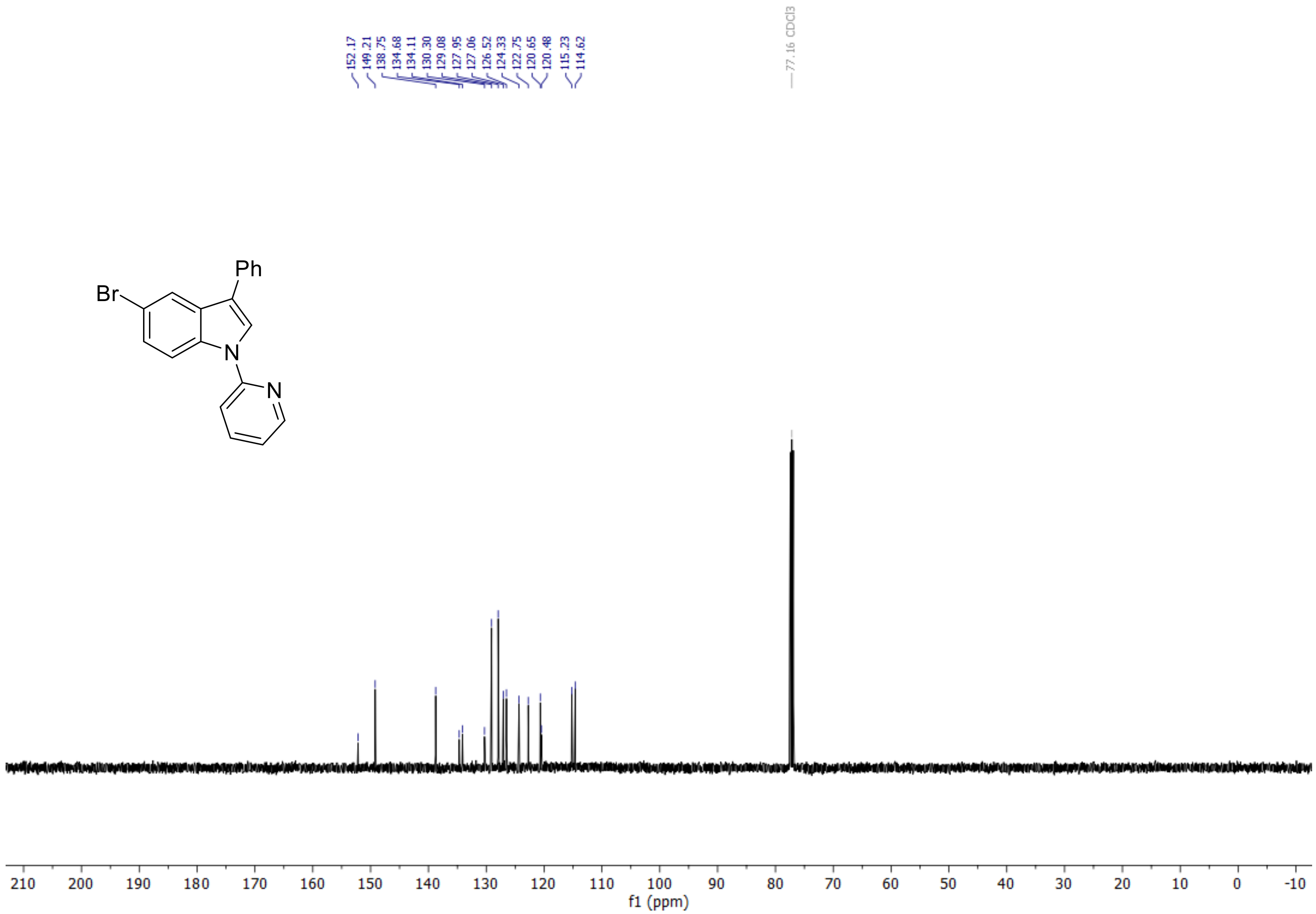

^{1}H–^{13}C HSQC-DEPT NMR (400 MHz, $CDCl_3$) of **2j**

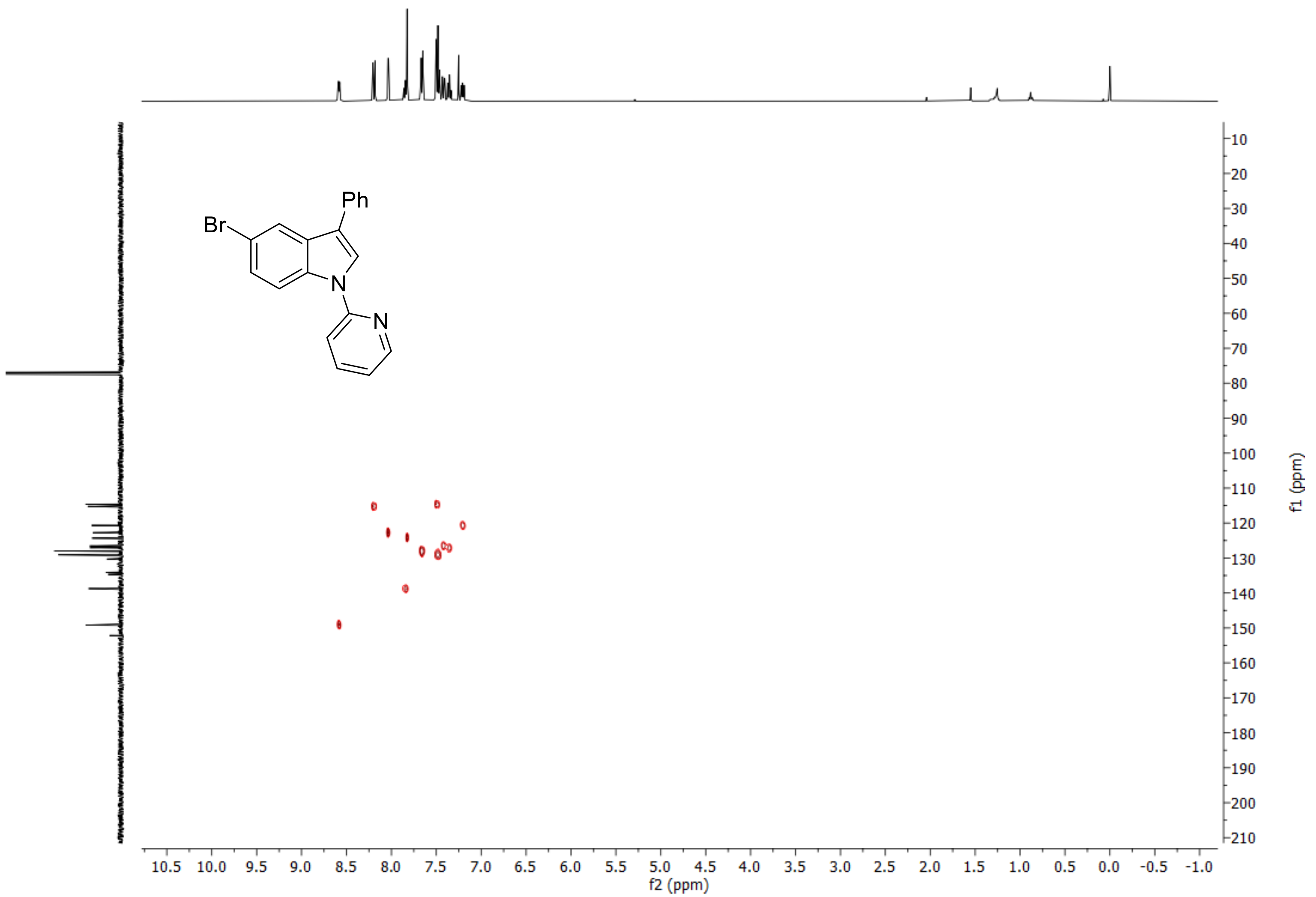

^{1}H NMR (400 MHz, $CDCl_3$) of **2k**

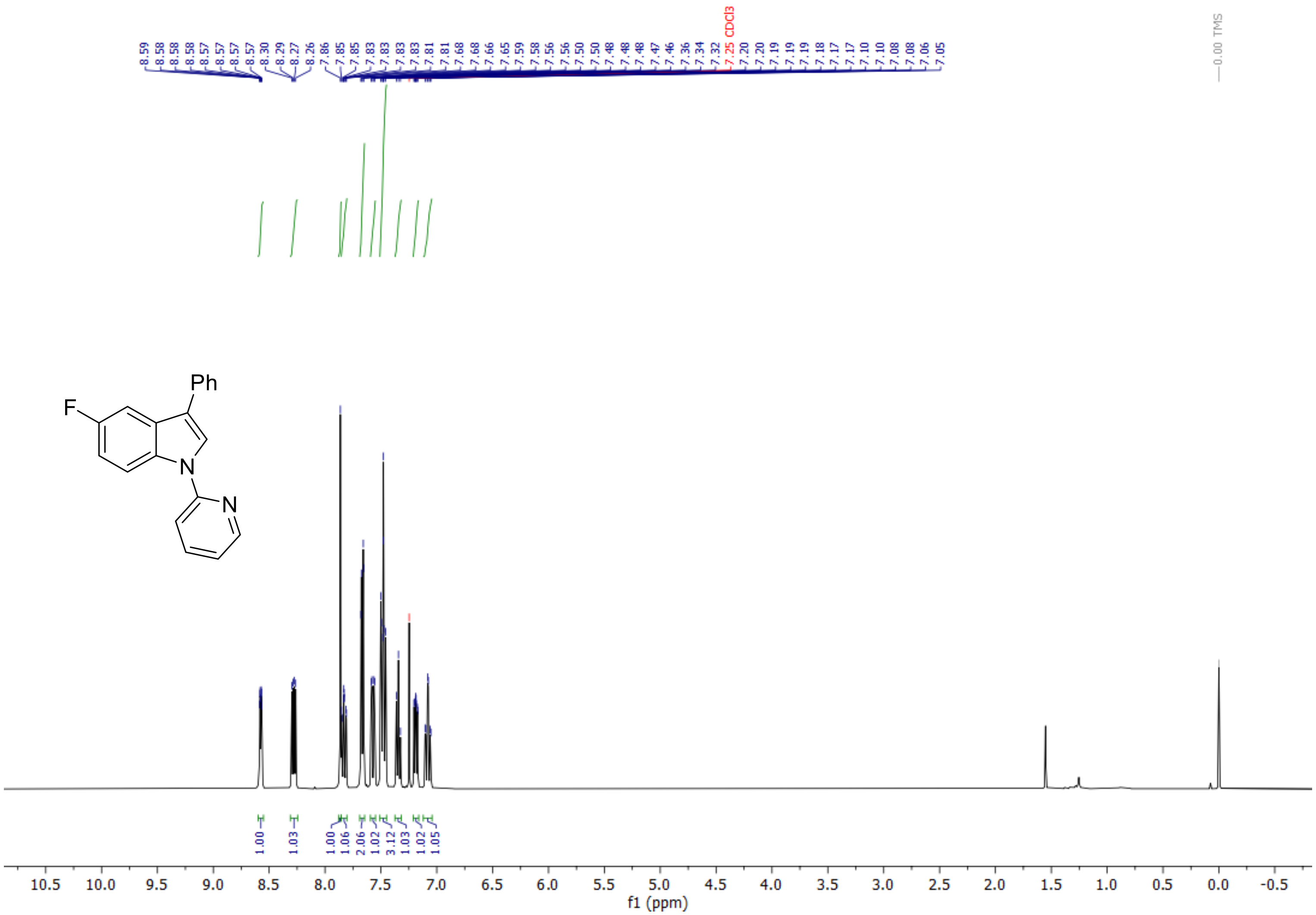

^{13}C NMR (126 MHz, $CDCl_3$) of **2k**

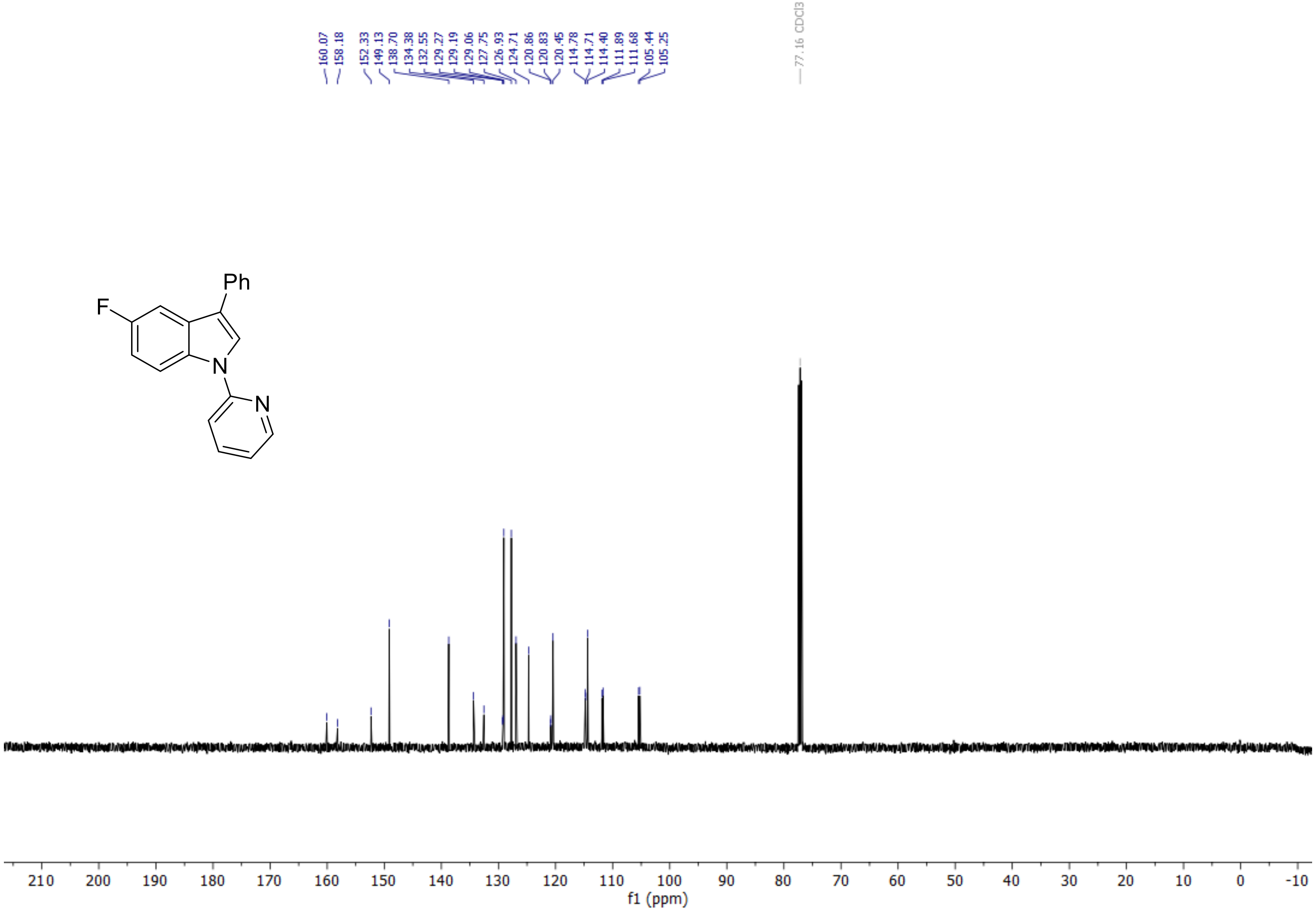

^{1}H–^{13}C HSQC-DEPT NMR (400 MHz, $CDCl_3$) of **2k**

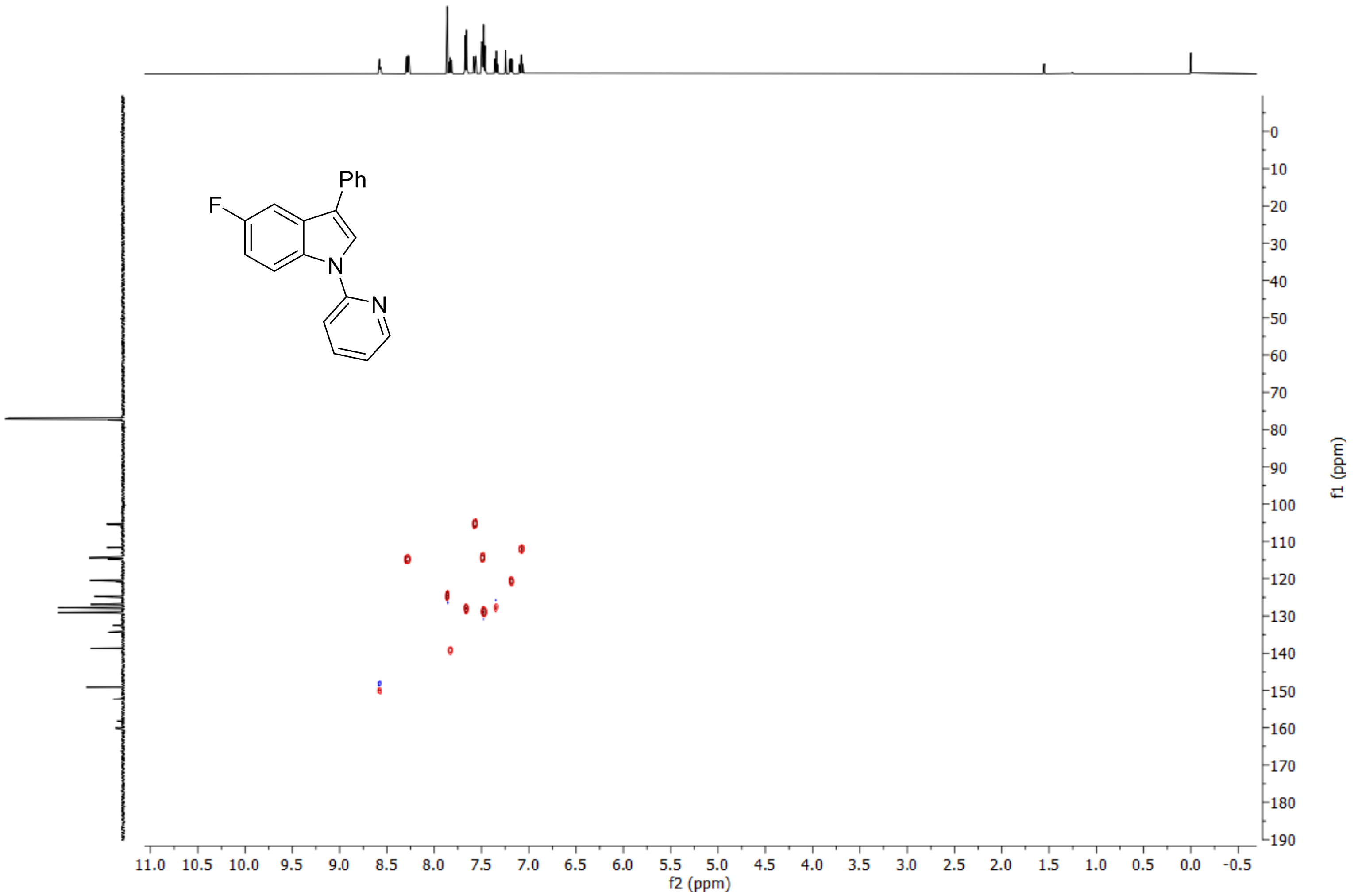

^{19}F NMR (376 MHz, $CDCl_3$) of **2k**

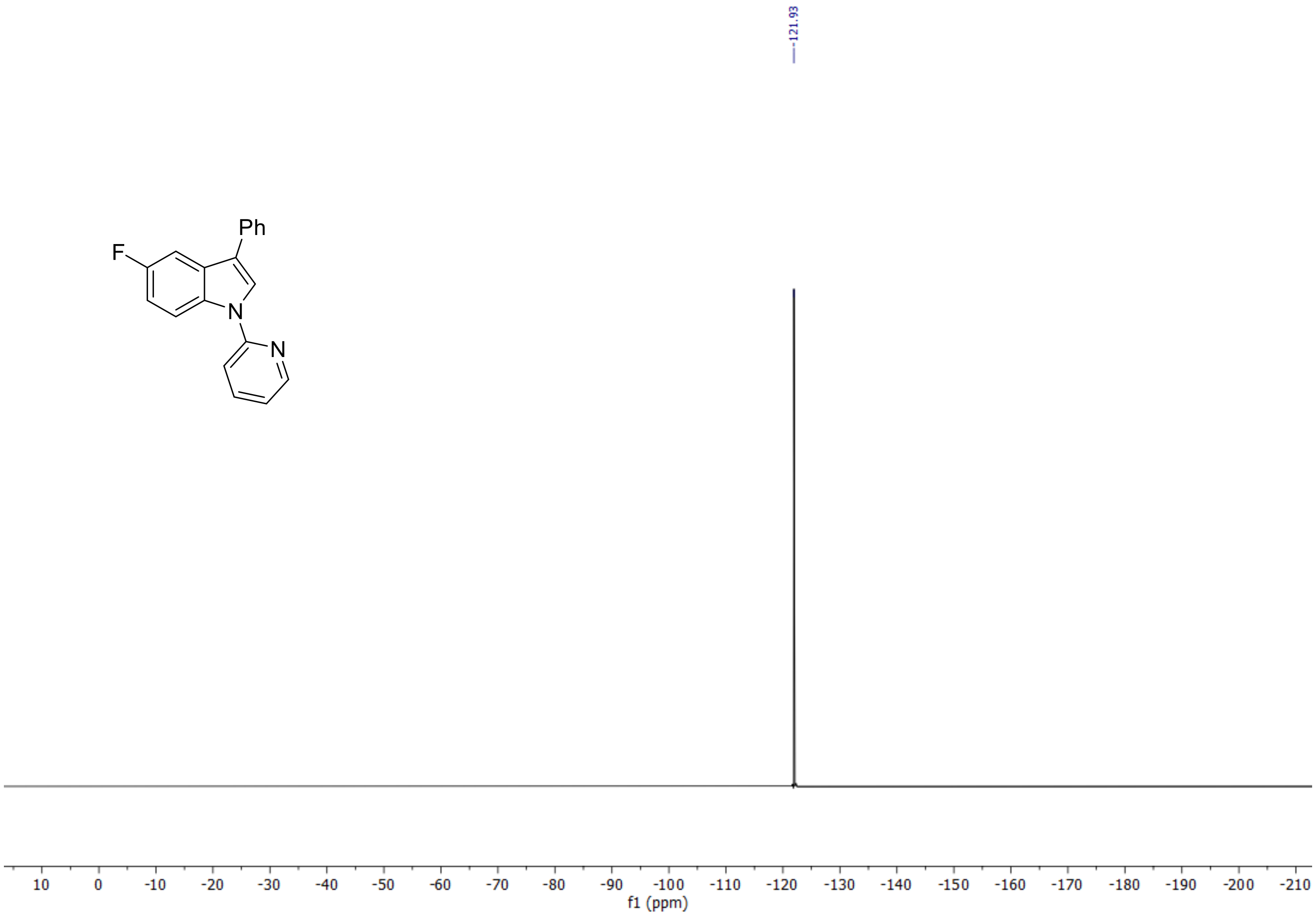

^{1}H NMR (400 MHz, $CDCl_3$) of **2l**

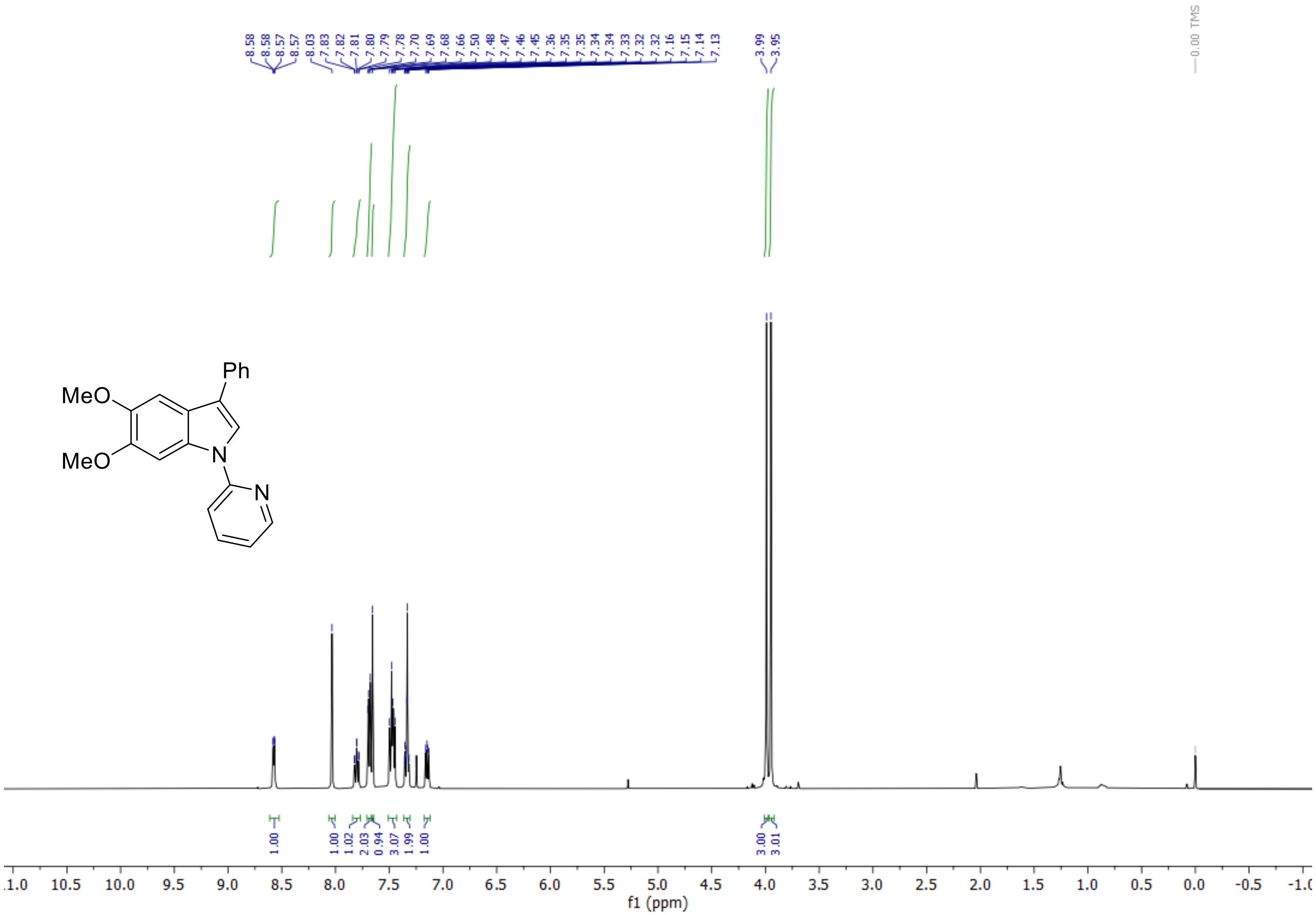

^{13}C NMR (101 MHz, $CDCl_3$) of **2l**

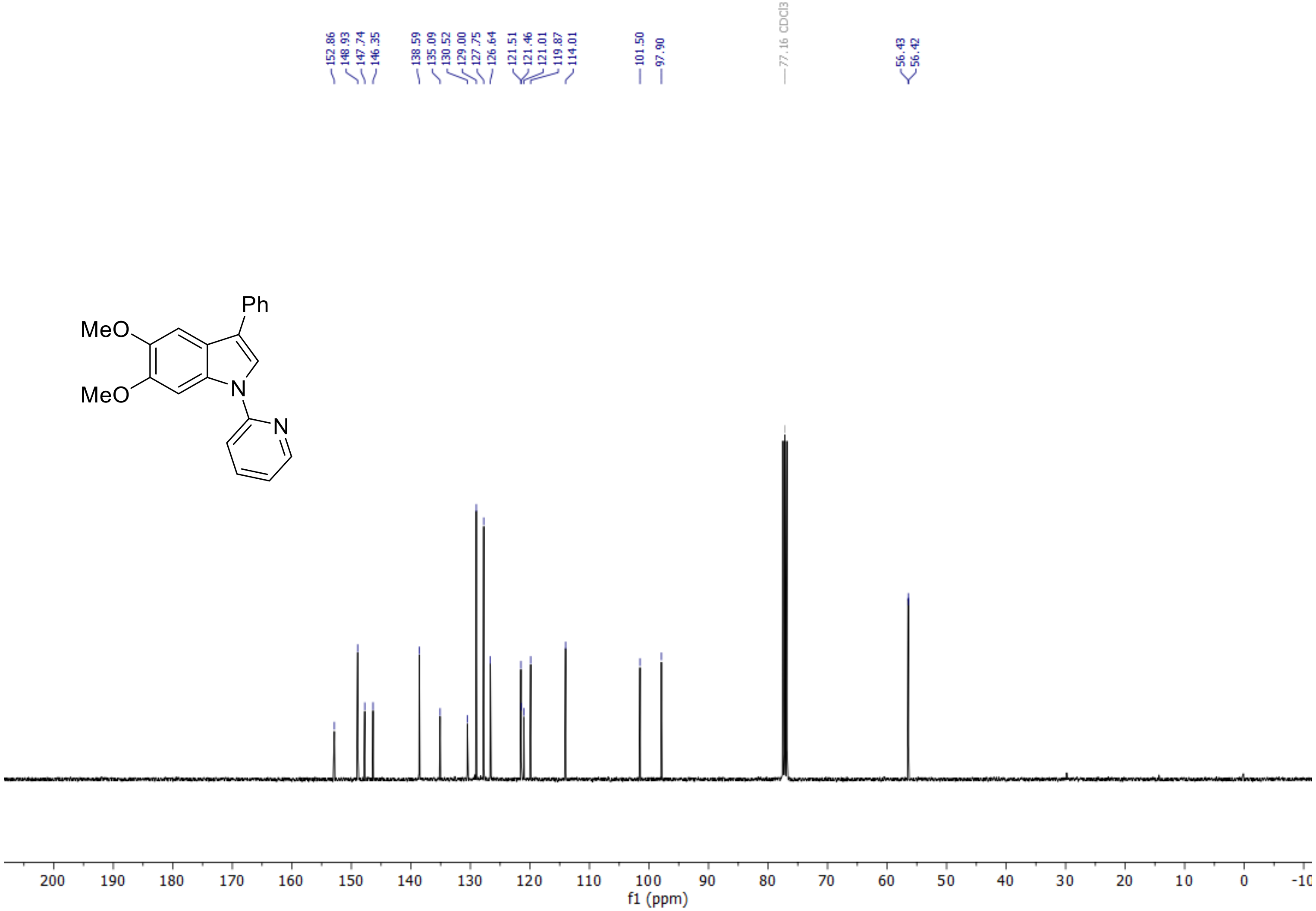

^{1}H–^{13}C HSQC-DEPT NMR (400 MHz, $CDCl_3$) of **2l**

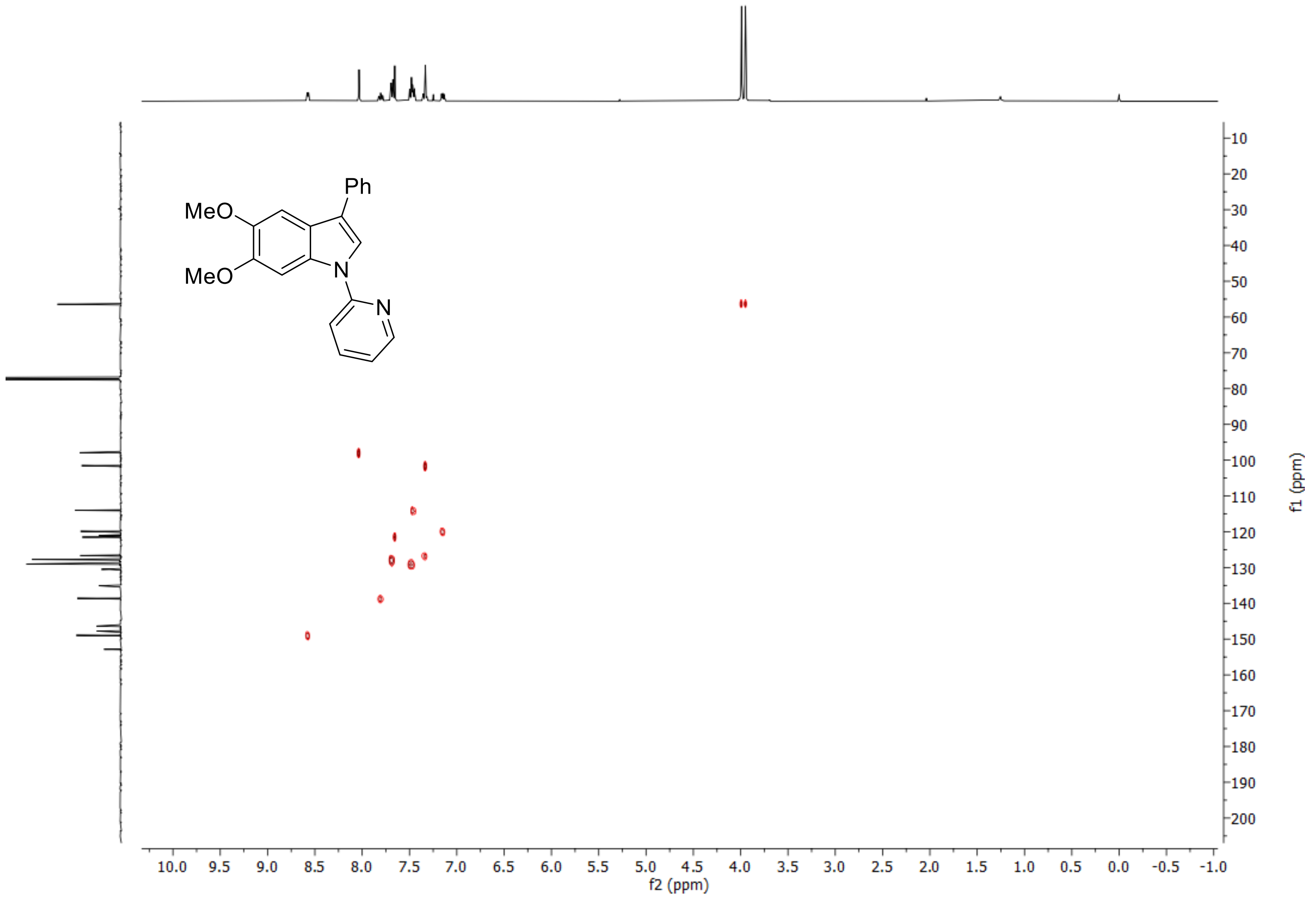

^{1}H NMR (400 MHz, $CDCl_3$) of **2m-1**

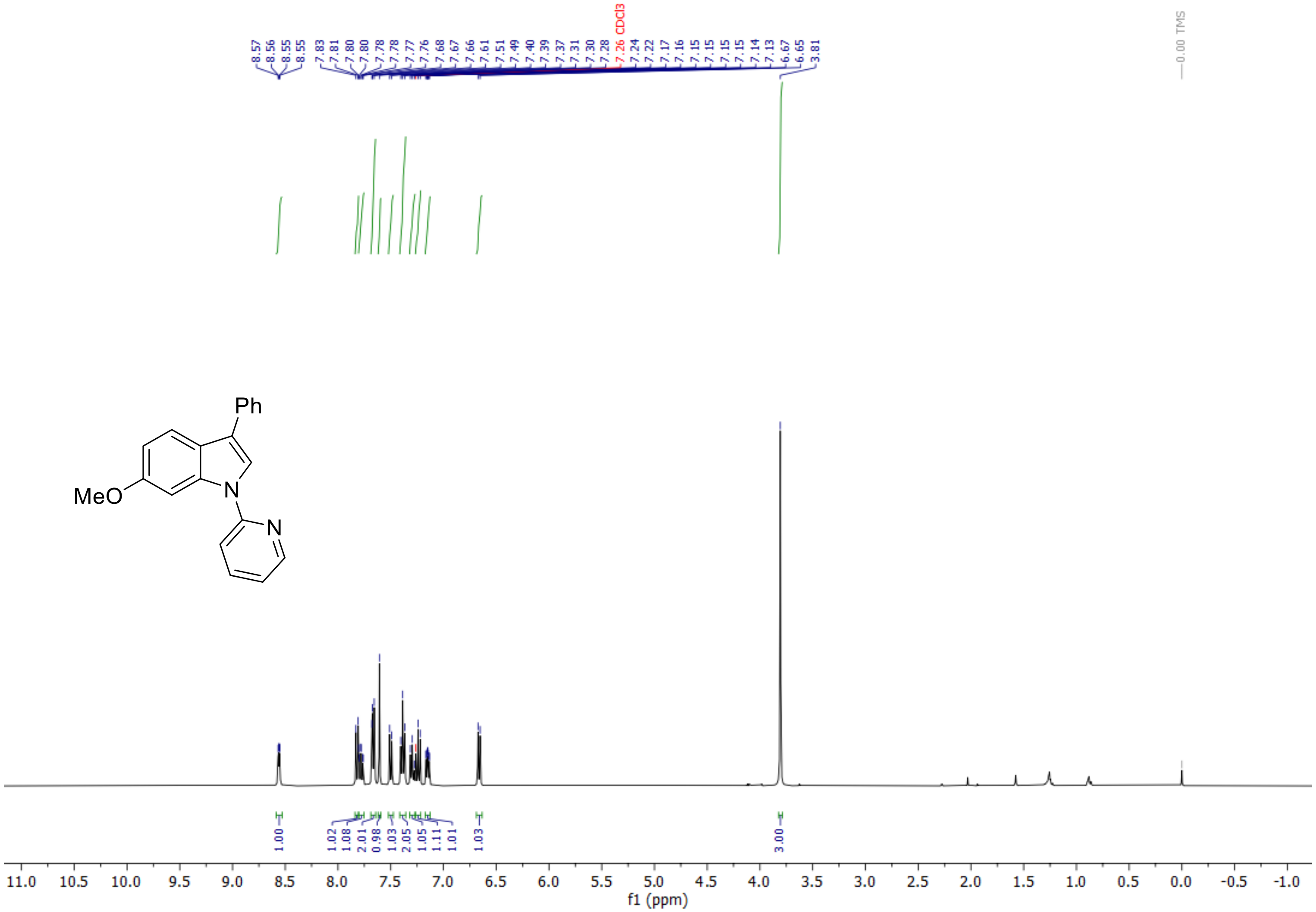

^{13}C NMR (101 MHz, $CDCl_3$) of **2m-1**

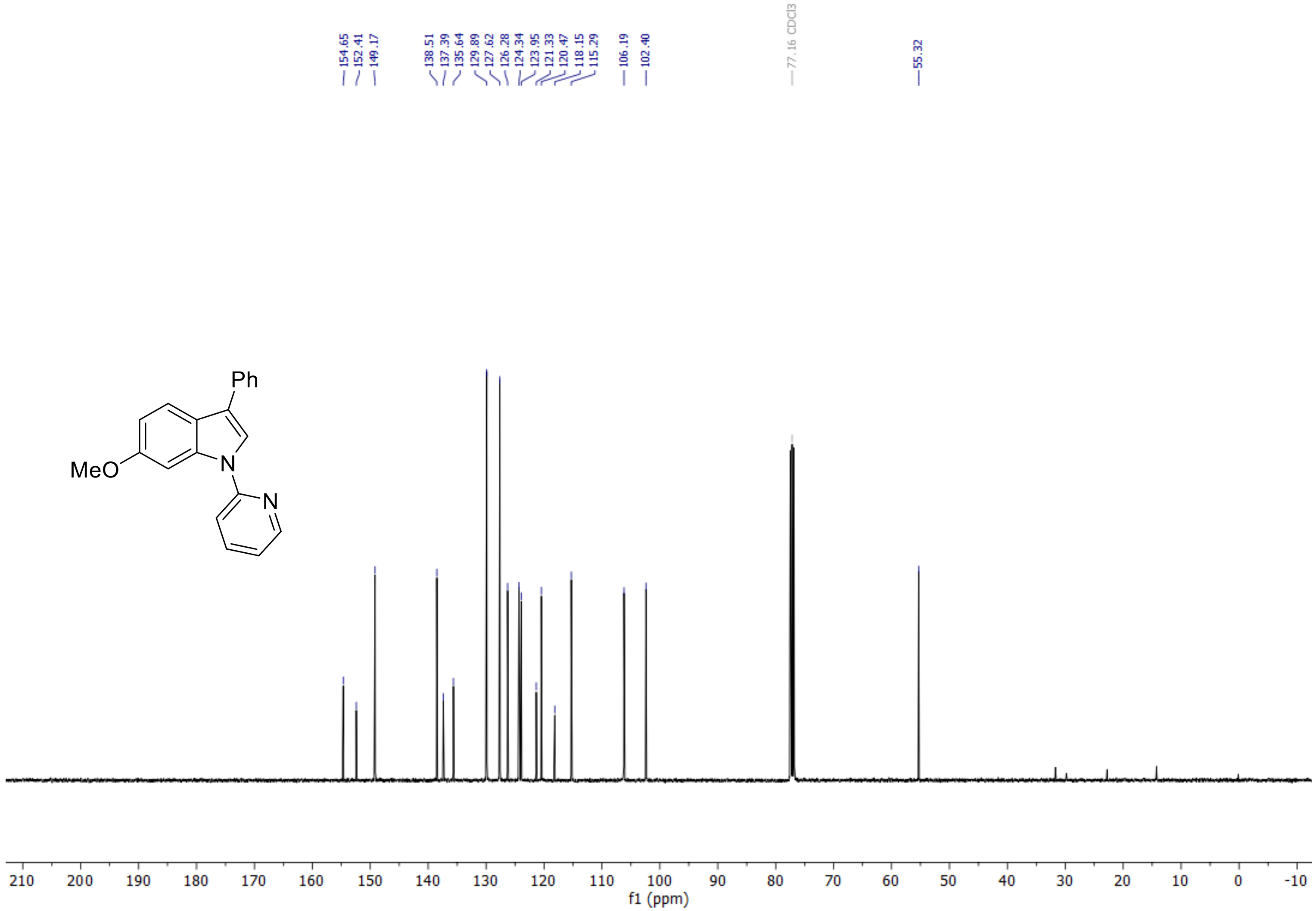

^{1}H–^{13}C HSQC-DEPT NMR (400 MHz, $CDCl_3$) of **2m-1**

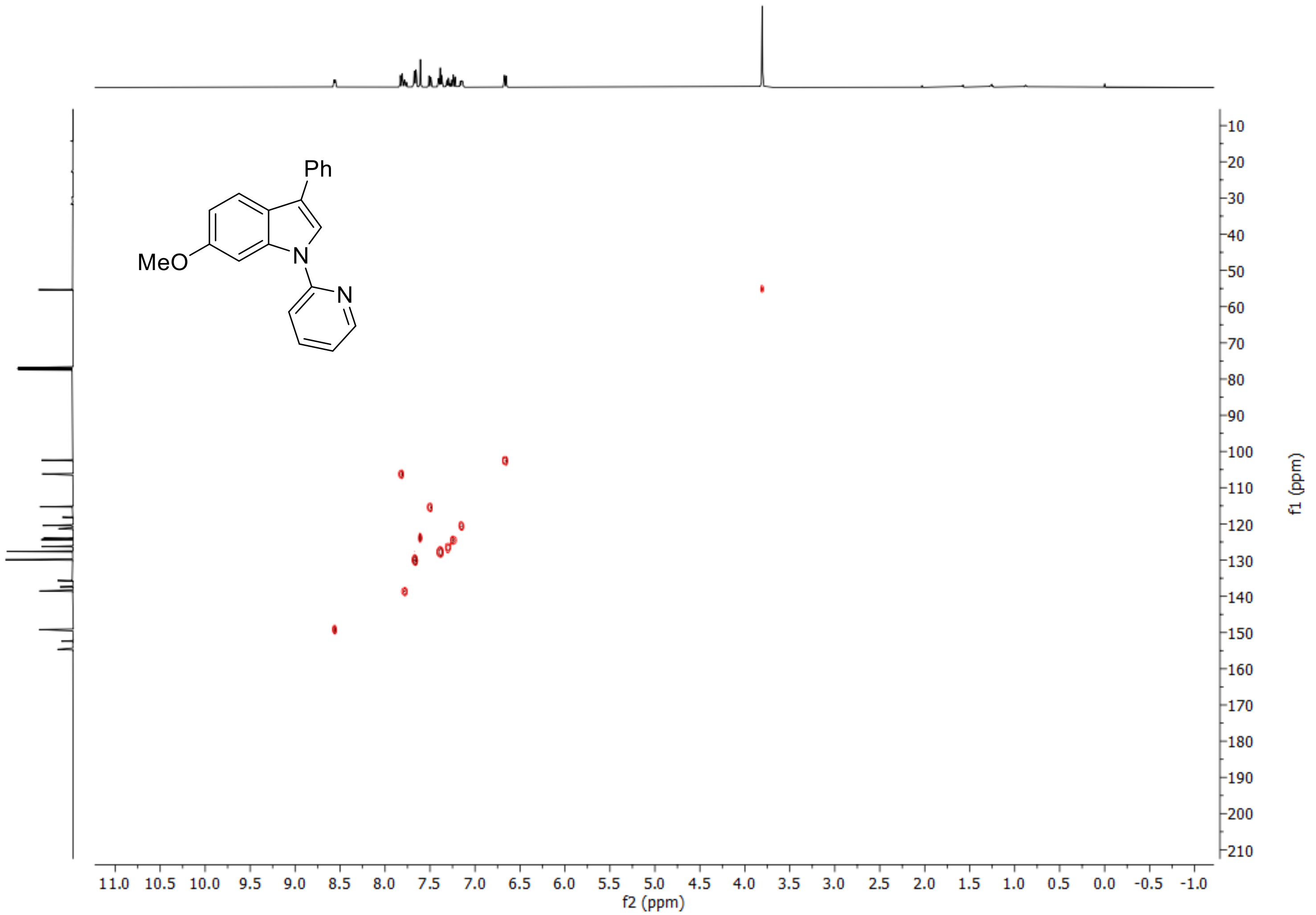

^{1}H NMR (400 MHz, $CDCl_3$) of **2m-2**

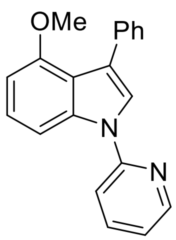

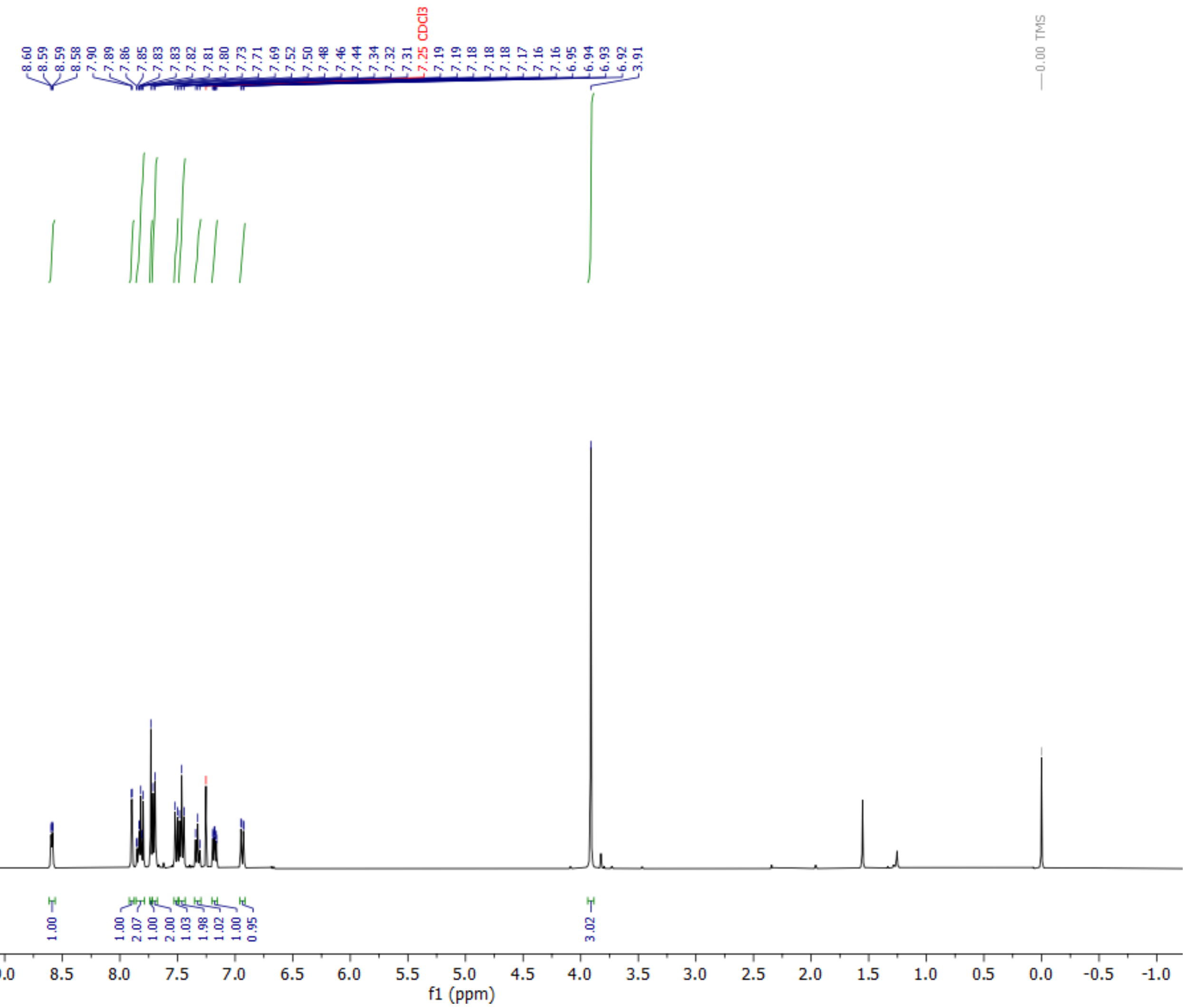

^{13}C NMR (101 MHz, $CDCl_3$) of **2m-2**

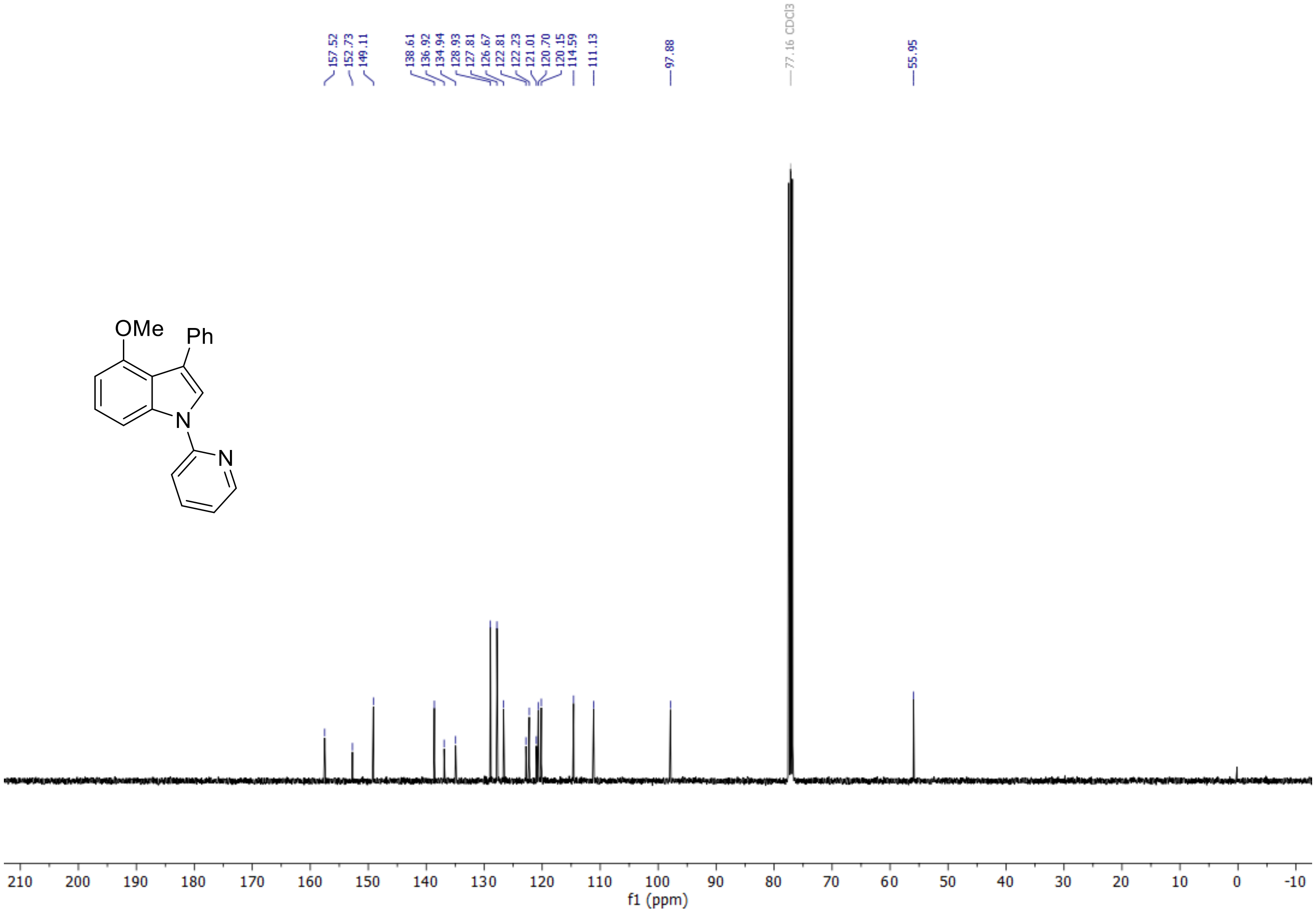

^{1}H–^{13}C HSQC-DEPT NMR (400 MHz, $CDCl_3$) of **2m-2**

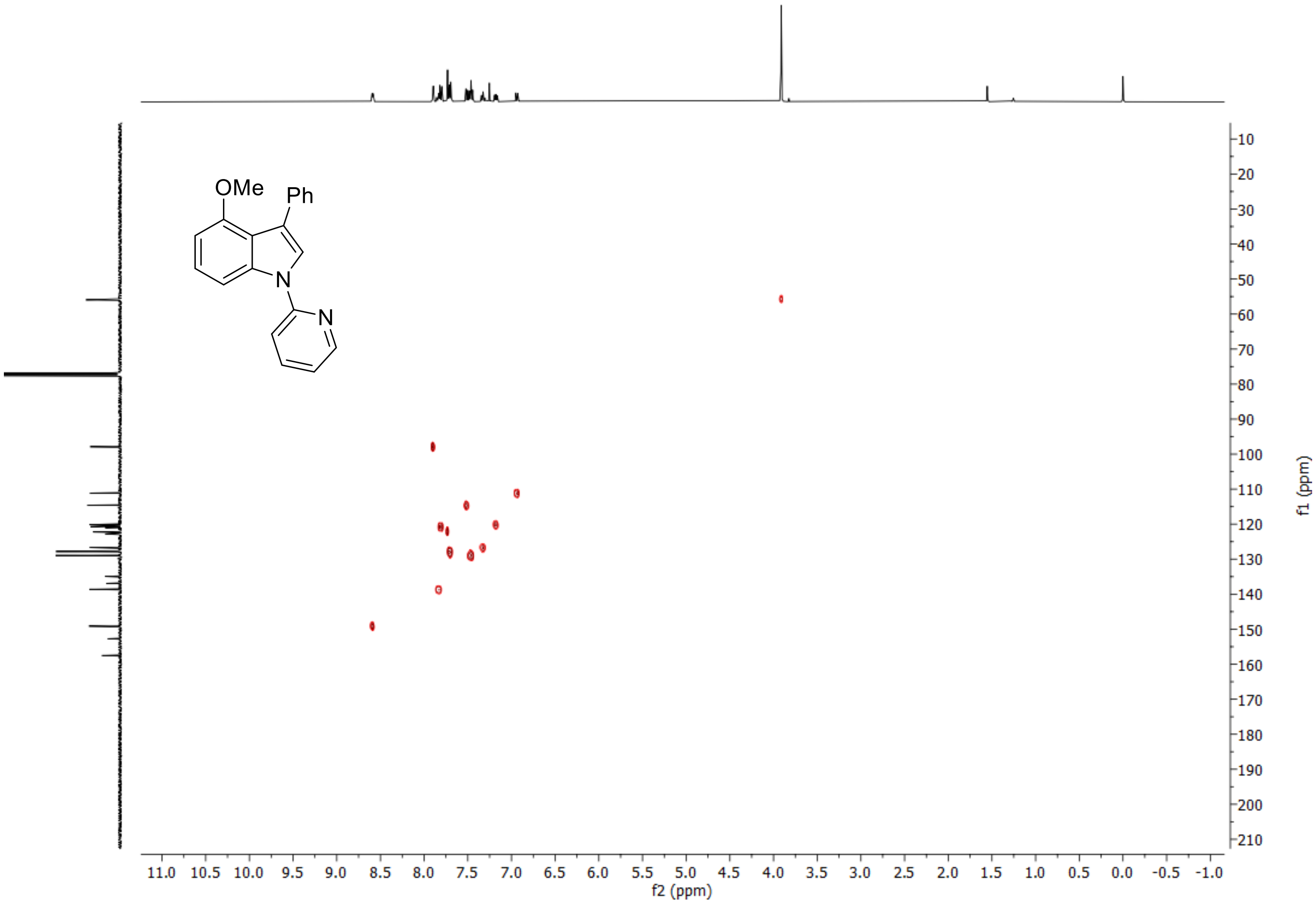

^{1}H NMR (400 MHz, $CDCl_3$) of **2n**

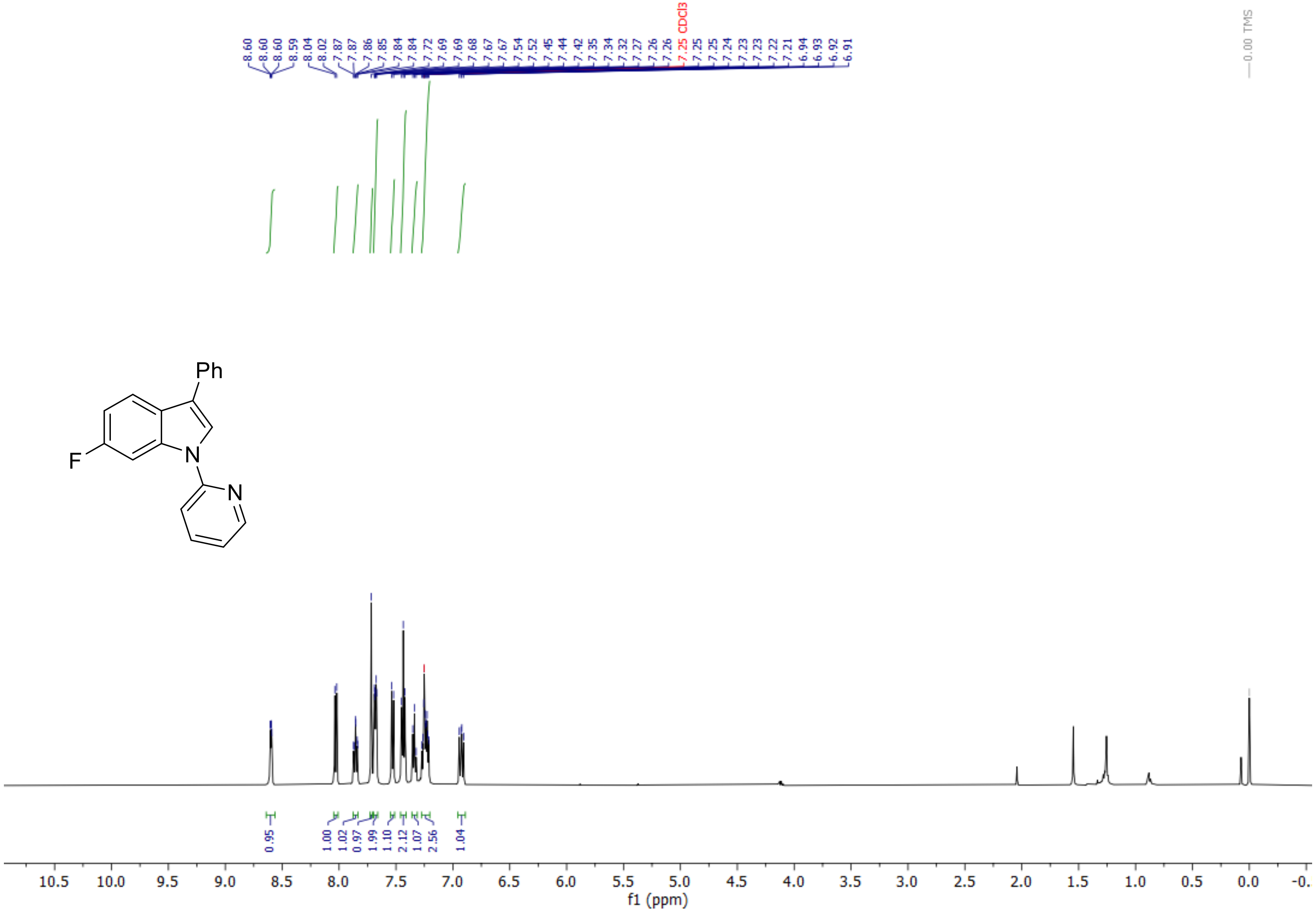

^{13}C NMR (101 MHz, $CDCl_3$) of **2n**

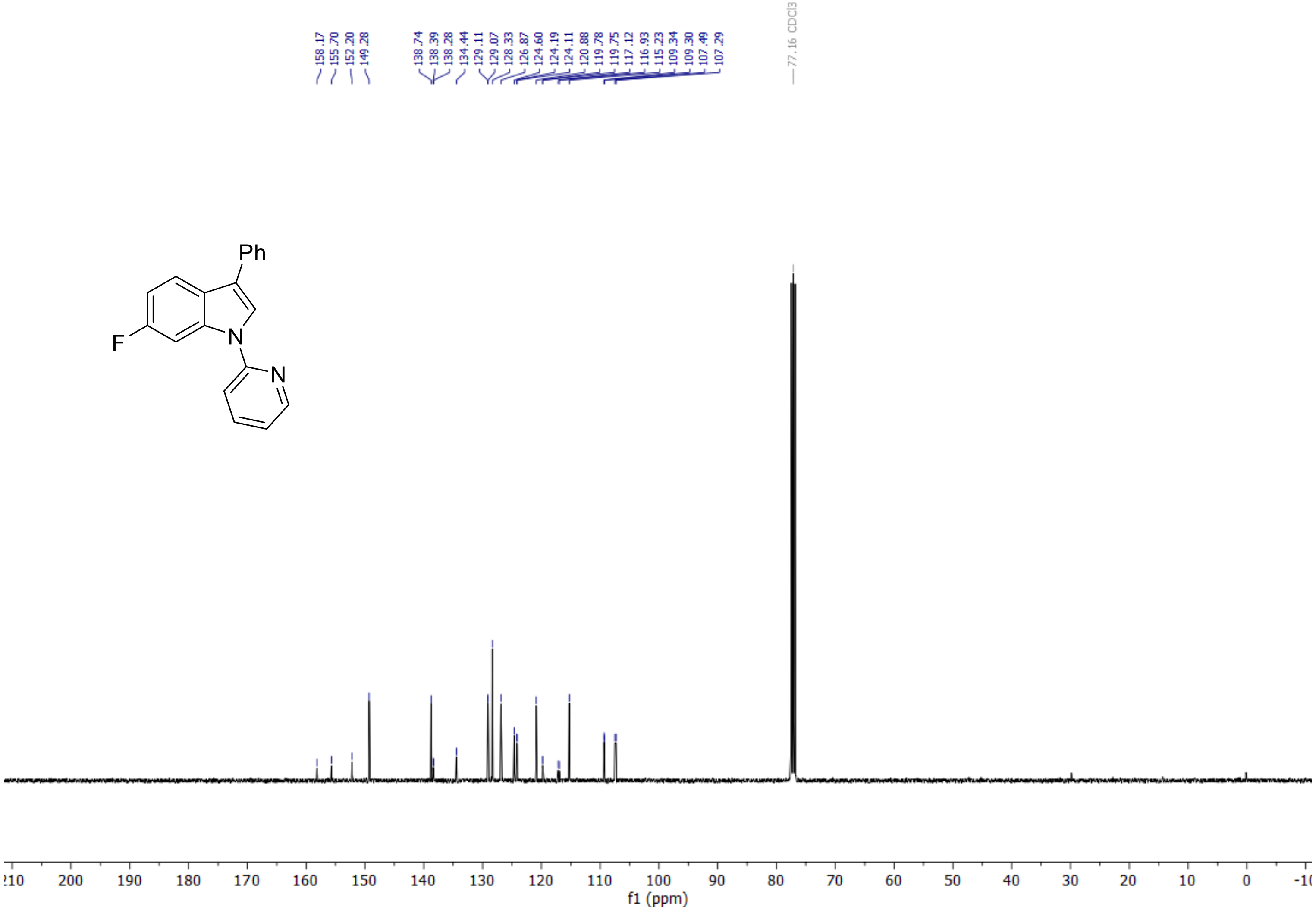

^{1}H–^{13}C HSQC-DEPT NMR (400 MHz, $CDCl_3$) of **2n**

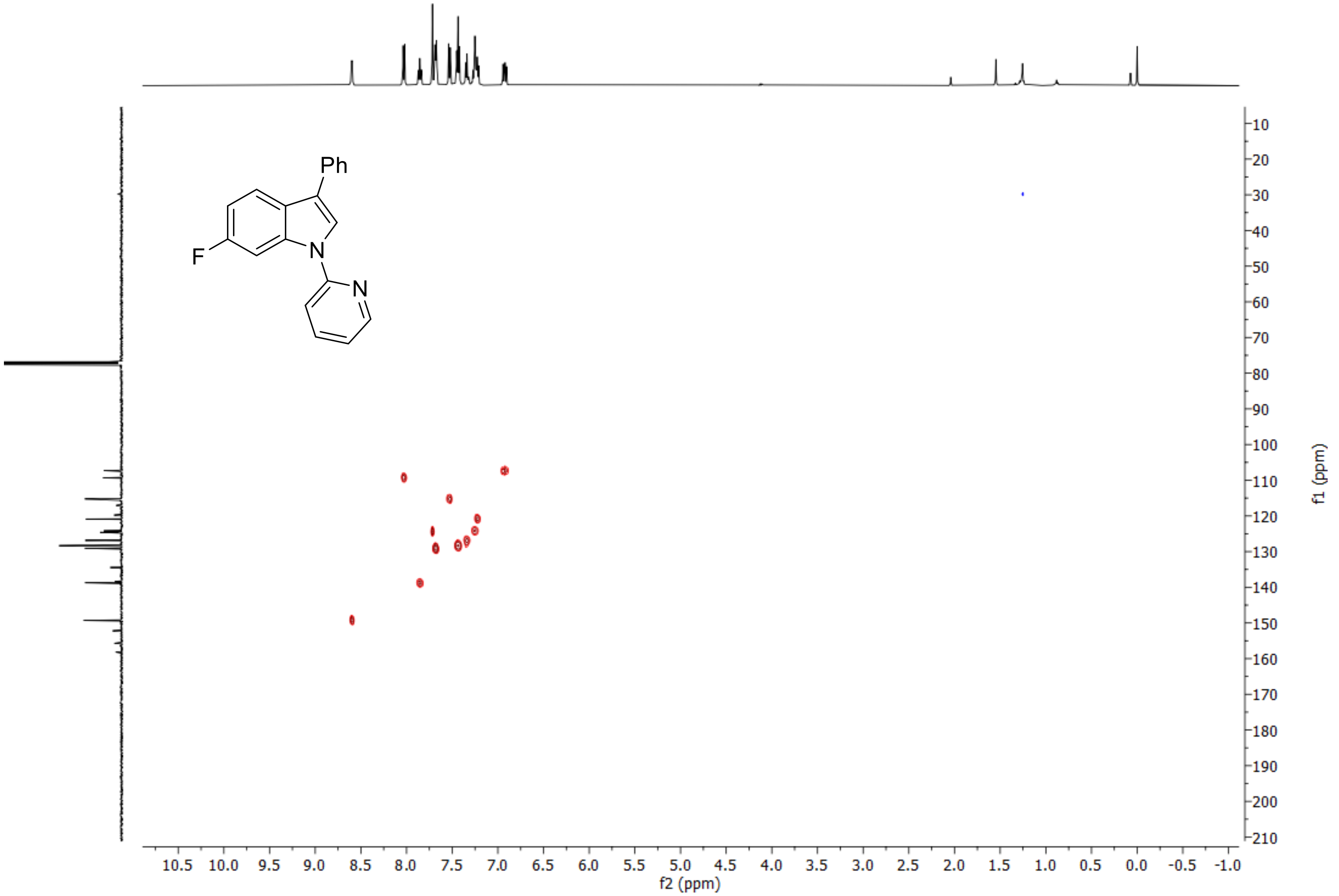

^{19}F NMR (376 MHz, $CDCl_3$) of **2n**

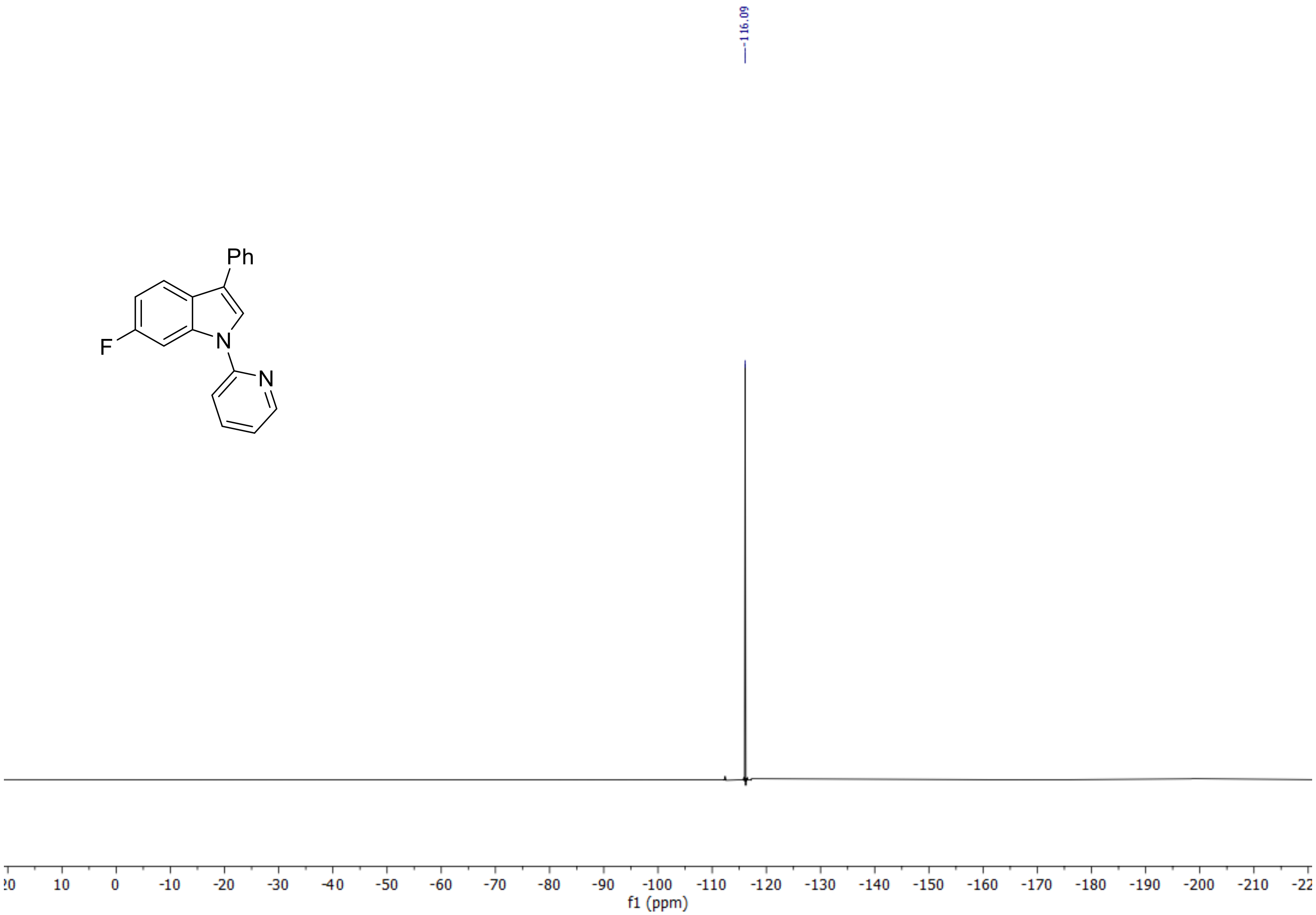

^{1}H NMR (400 MHz, $CDCl_3$) of **2o**

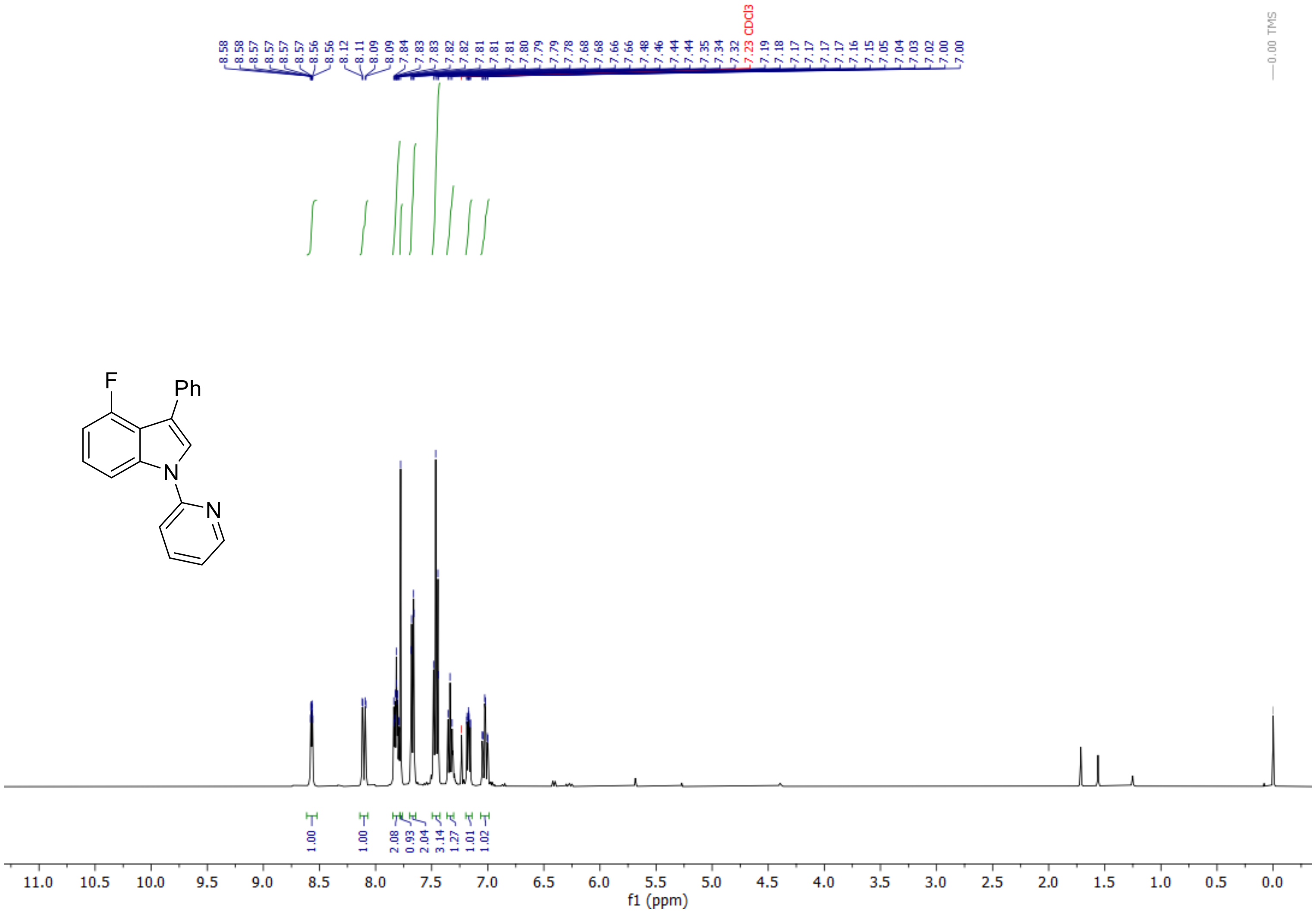

^{13}C NMR (101 MHz, $CDCl_3$) of **2o**

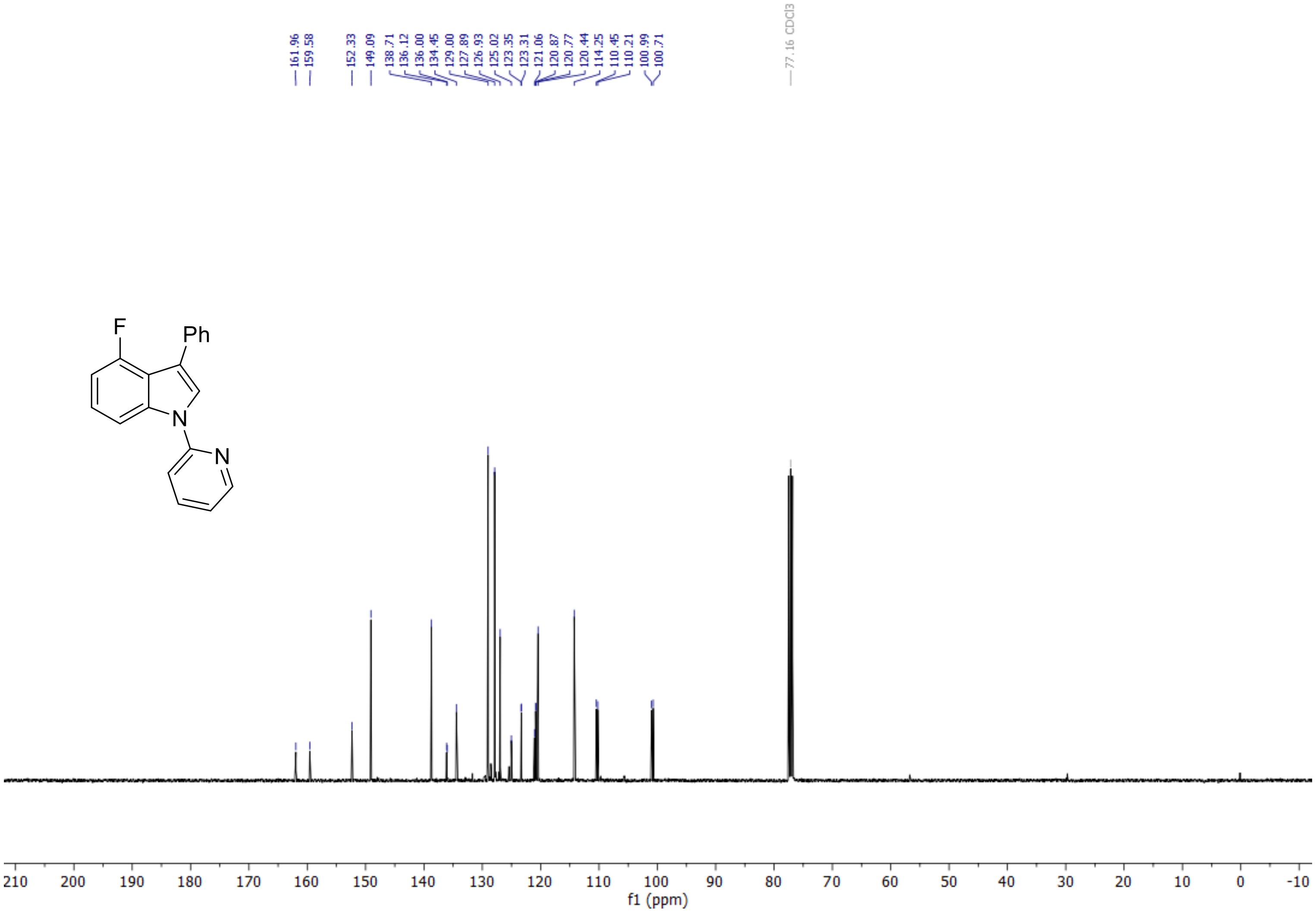

^{1}H–^{13}C HSQC-DEPT NMR (400 MHz, $CDCl_3$) of **2o**

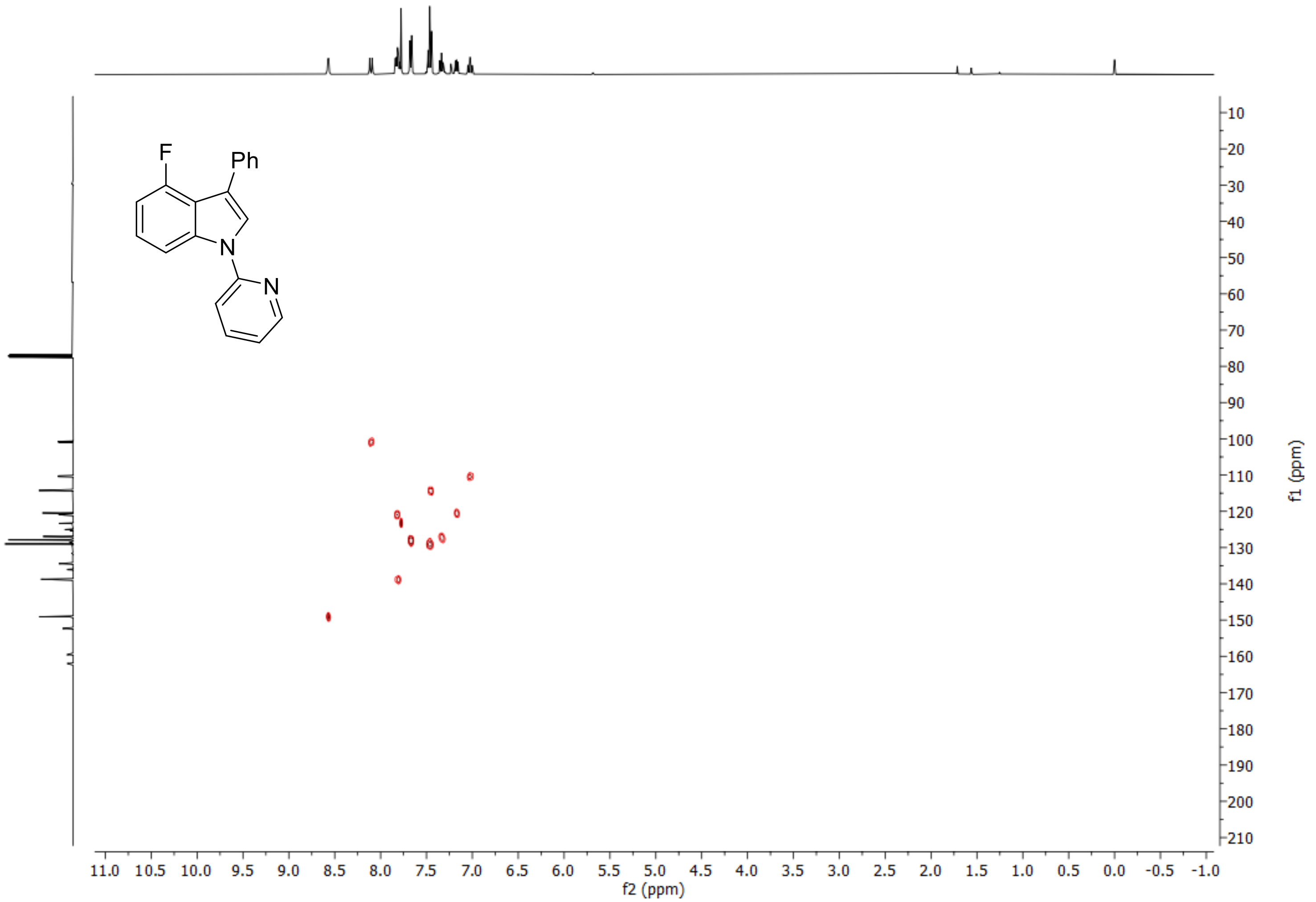

^{19}F NMR (376 MHz, $CDCl_3$) of **2o**

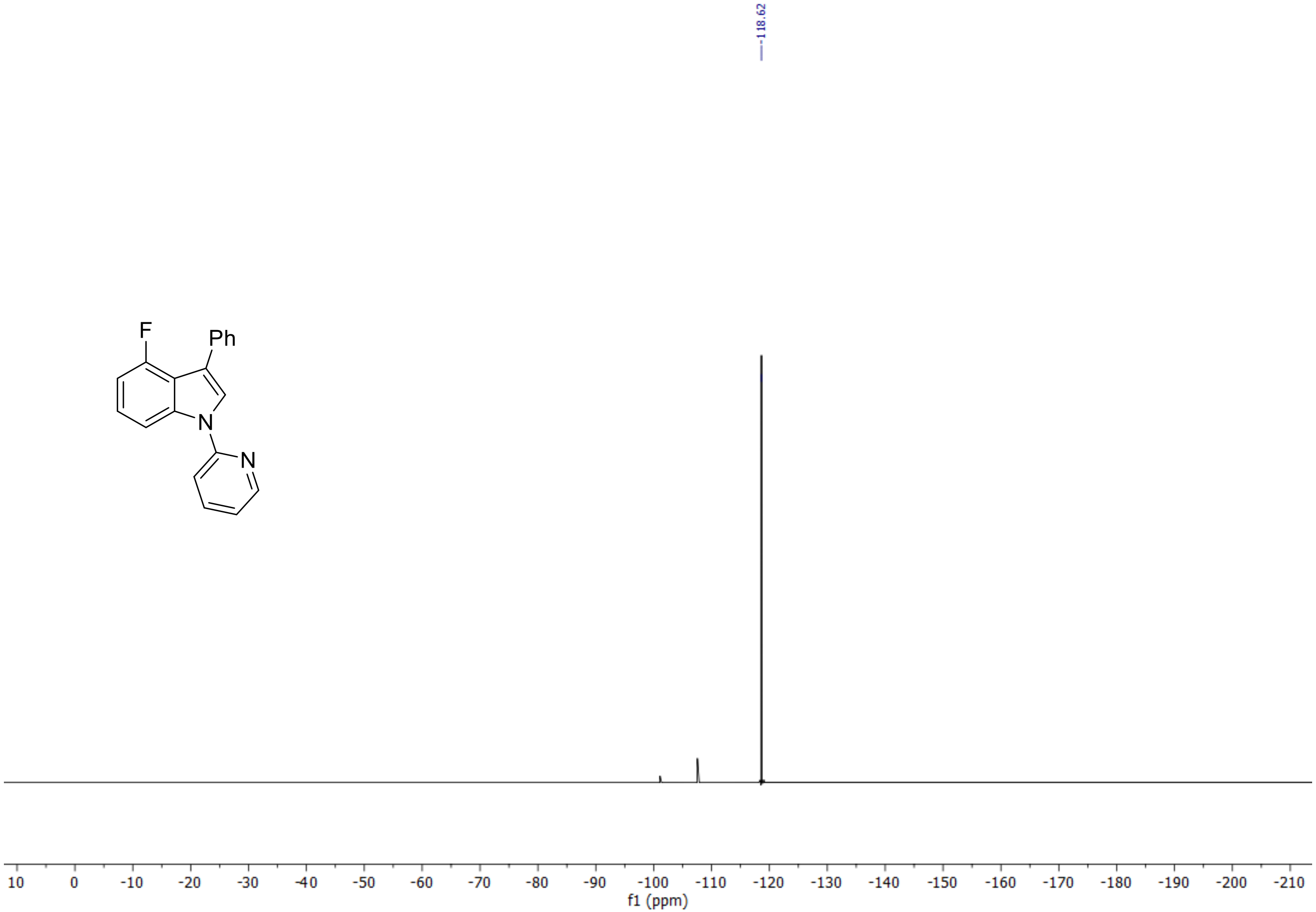

^{1}H NMR (400 MHz, $CDCl_3$) of **2p**

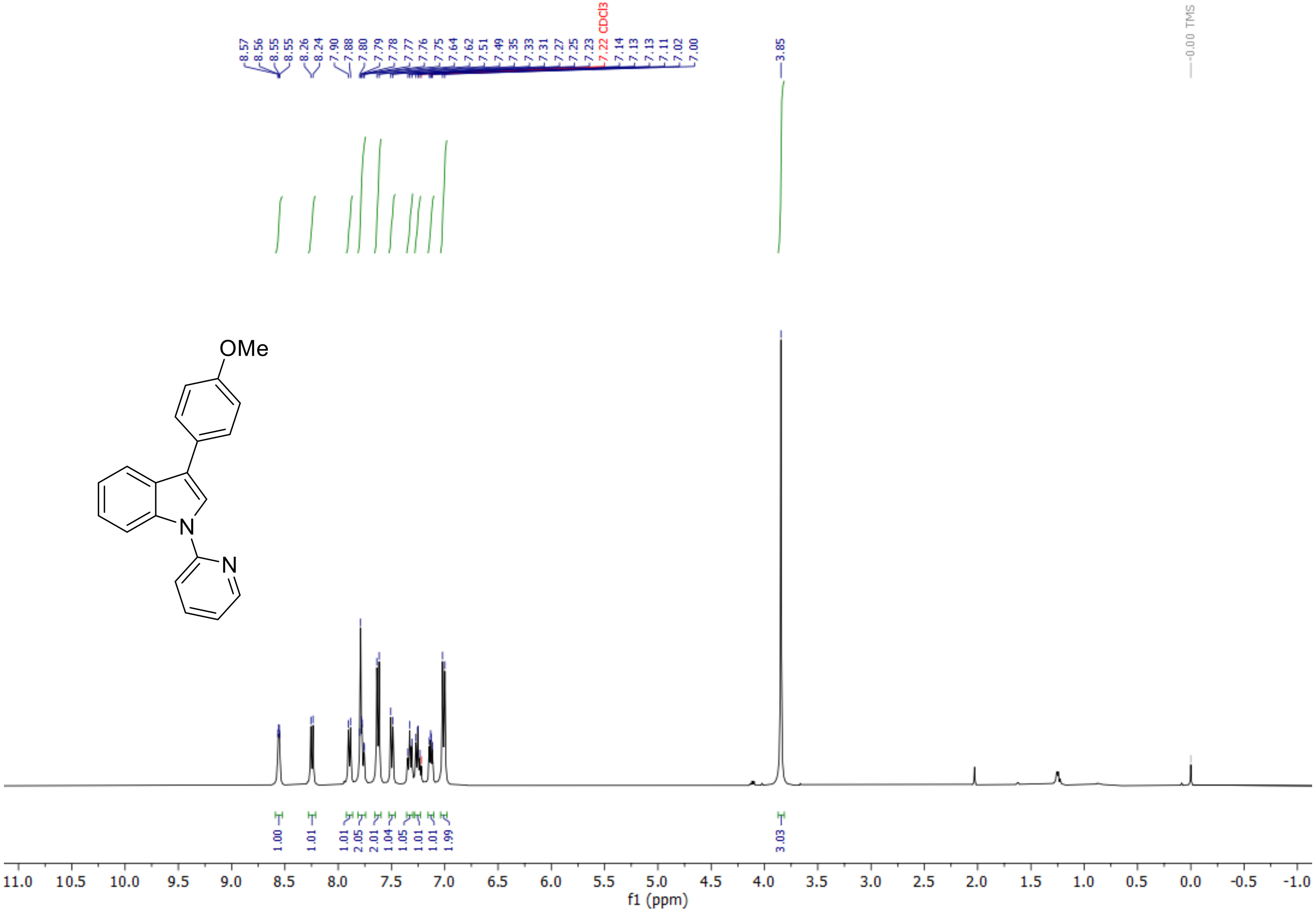

^{13}C NMR (101 MHz, $CDCl_3$) of **2p**

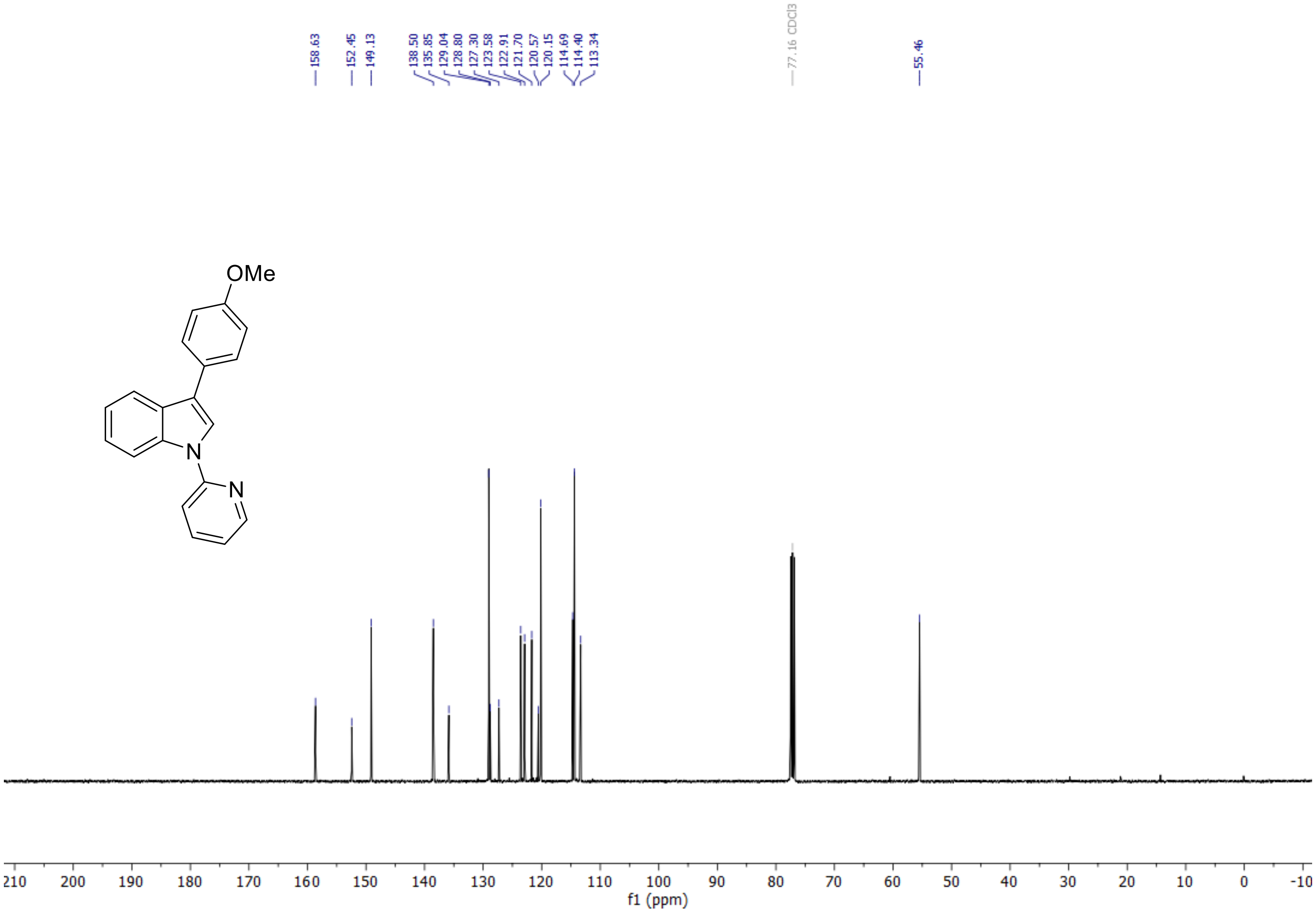

^{1}H–^{13}C HSQC-DEPT NMR (400 MHz, $CDCl_3$) of **2p**

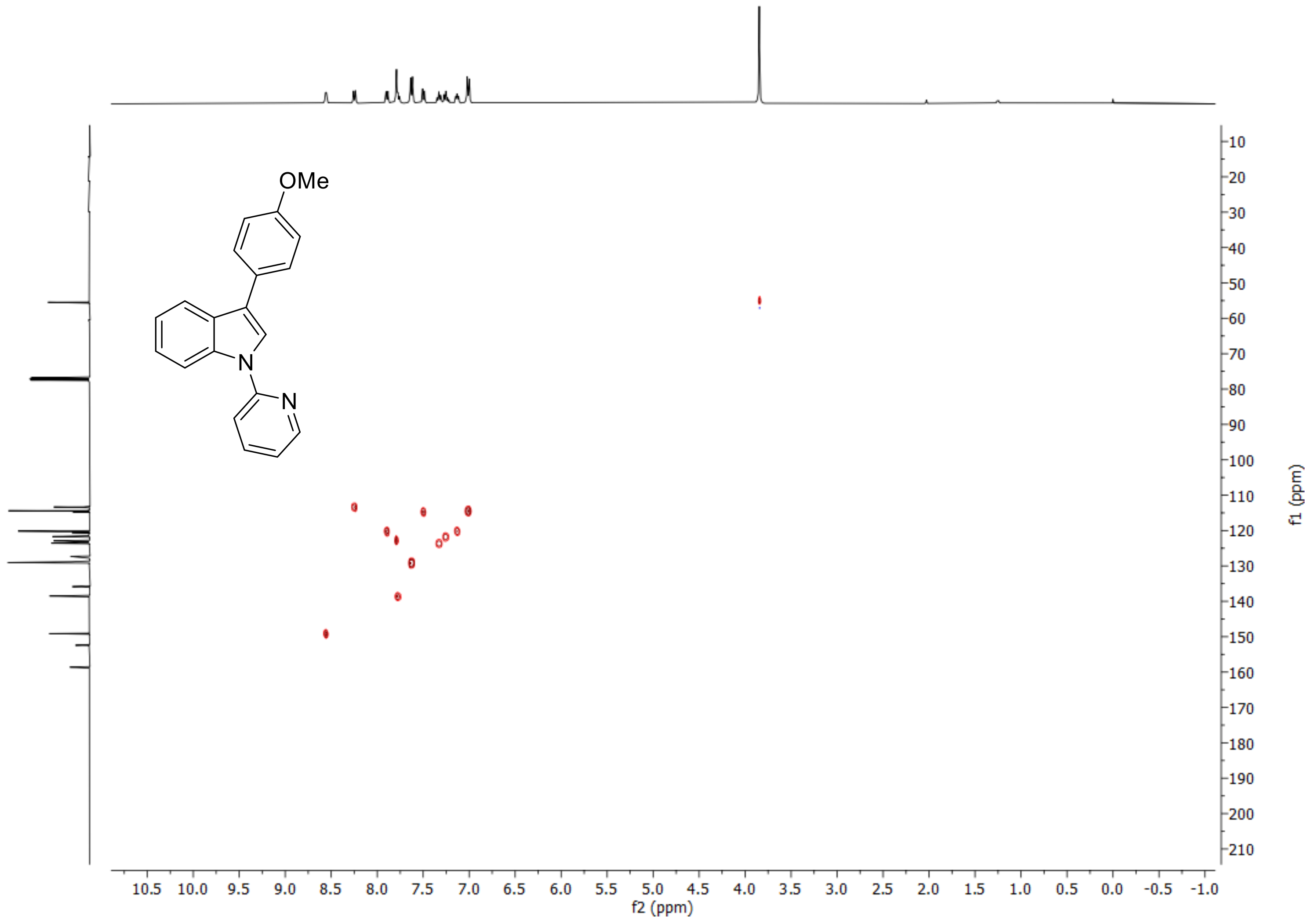

^{1}H NMR (400 MHz, $CDCl_3$) of **2q**

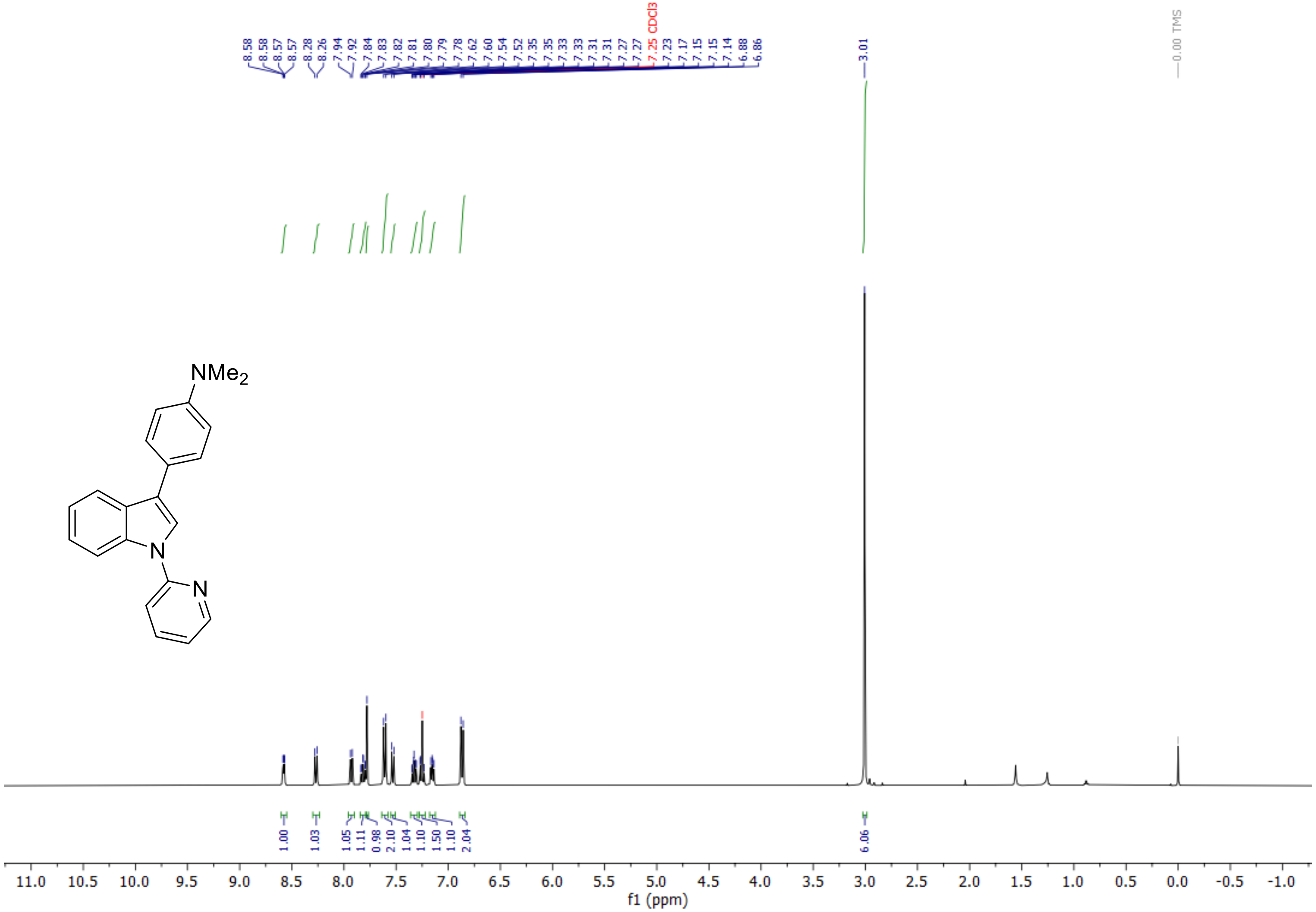

^{13}C NMR (101 MHz, $CDCl_3$) of **2q**

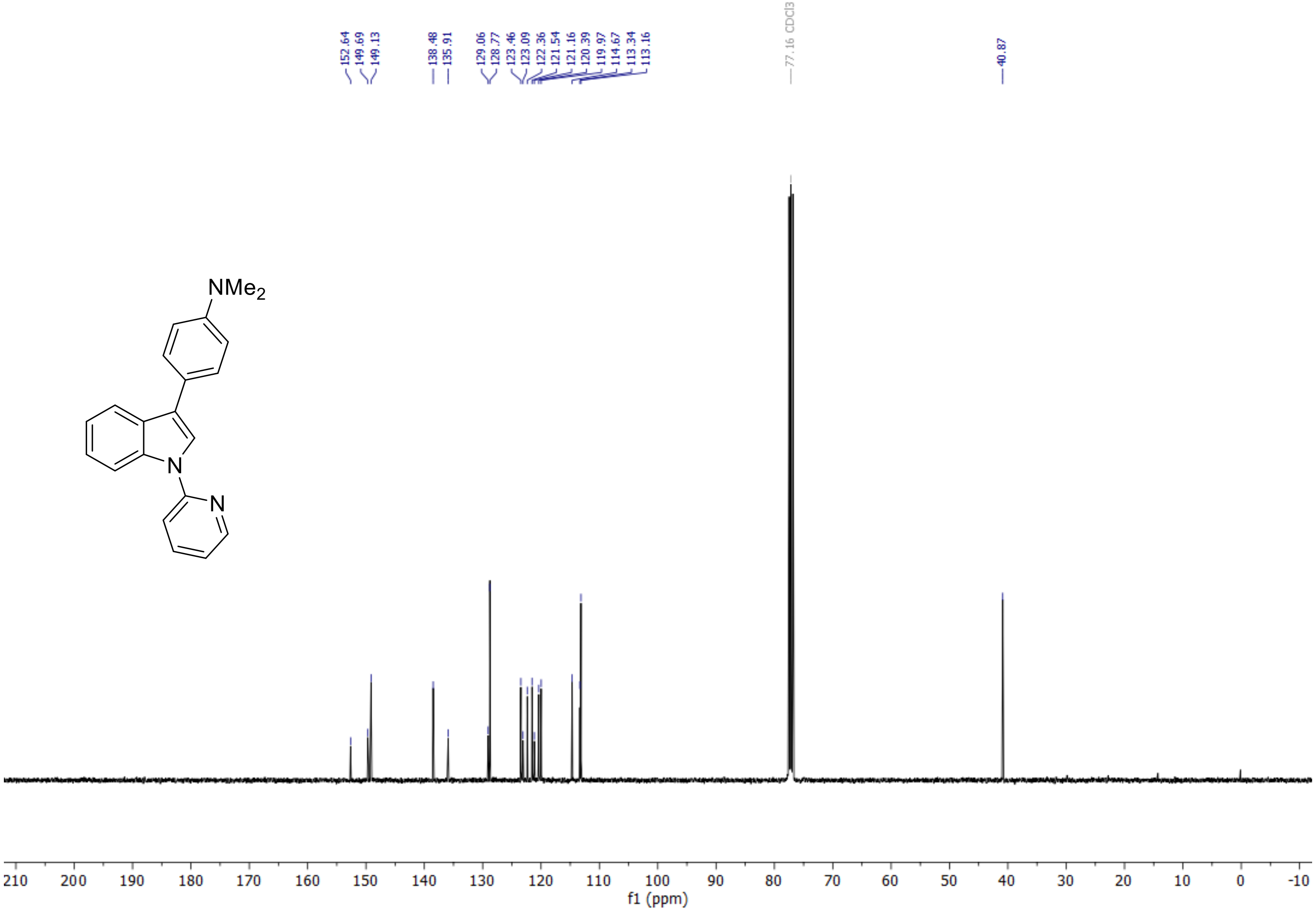

^{1}H–^{13}C HSQC-DEPT NMR (400 MHz, $CDCl_3$) of **2q**

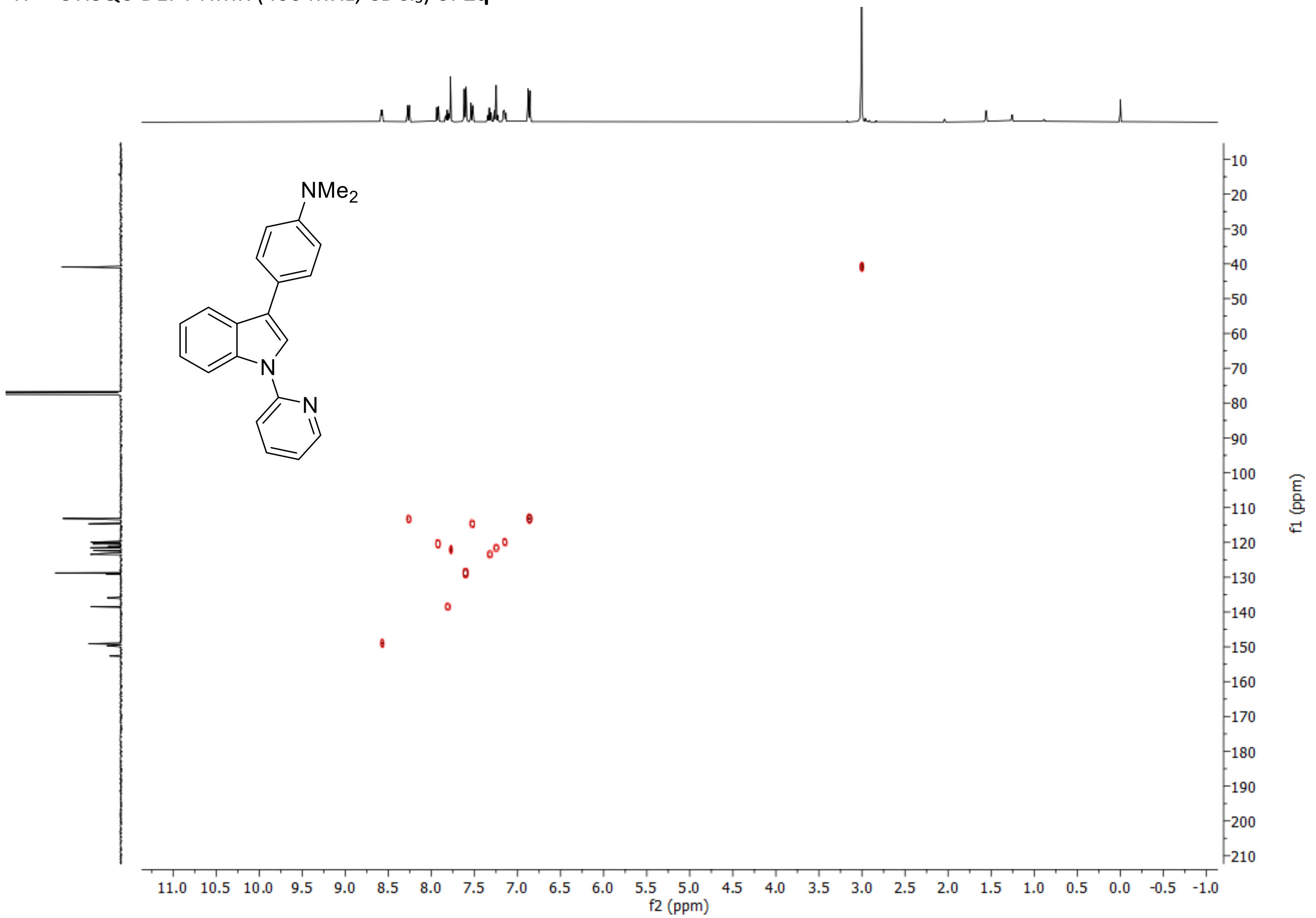

^{1}H NMR (400 MHz, $CDCl_3$) of **2r**

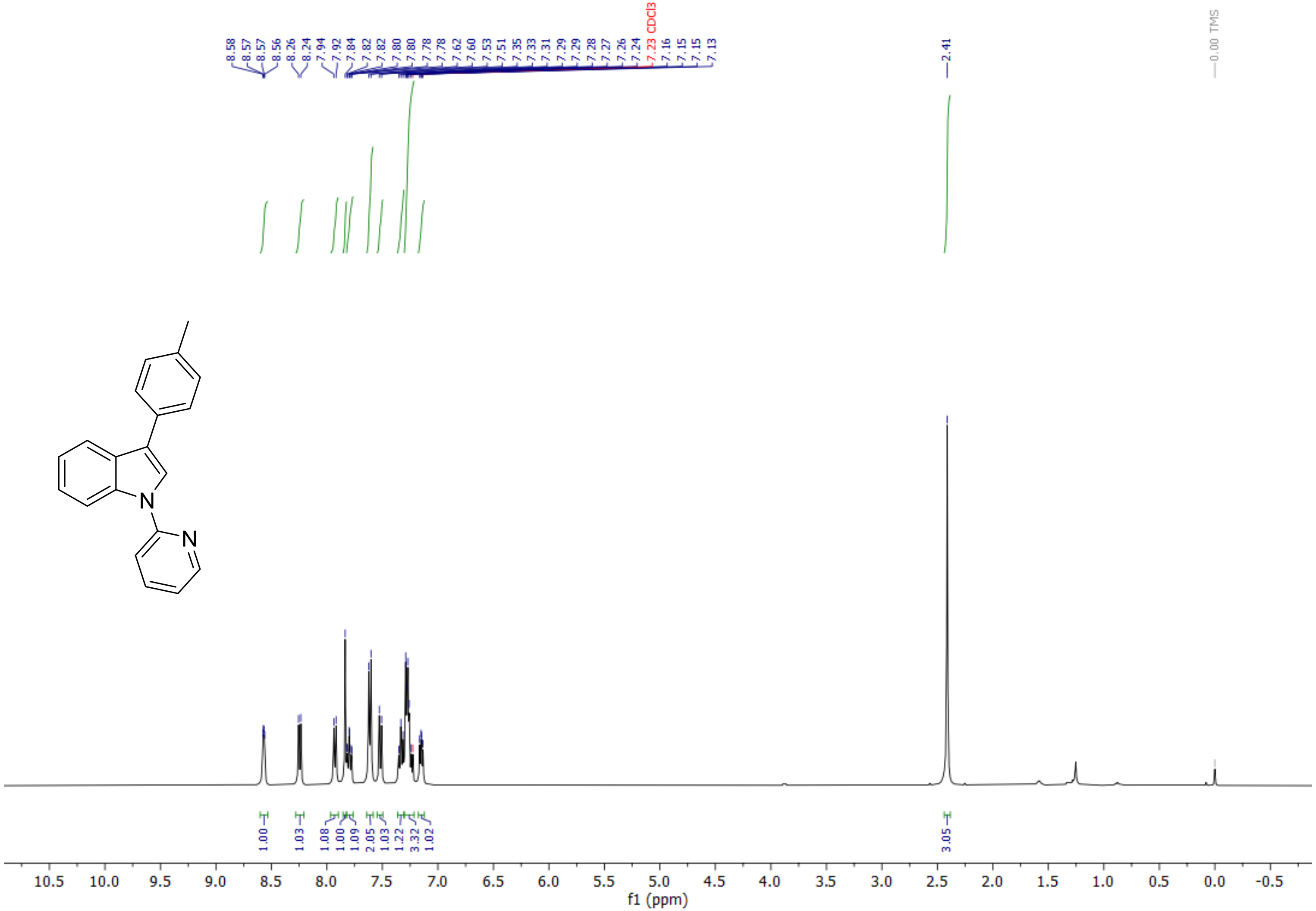

^{13}C NMR (101 MHz, $CDCl_3$) of **2r**

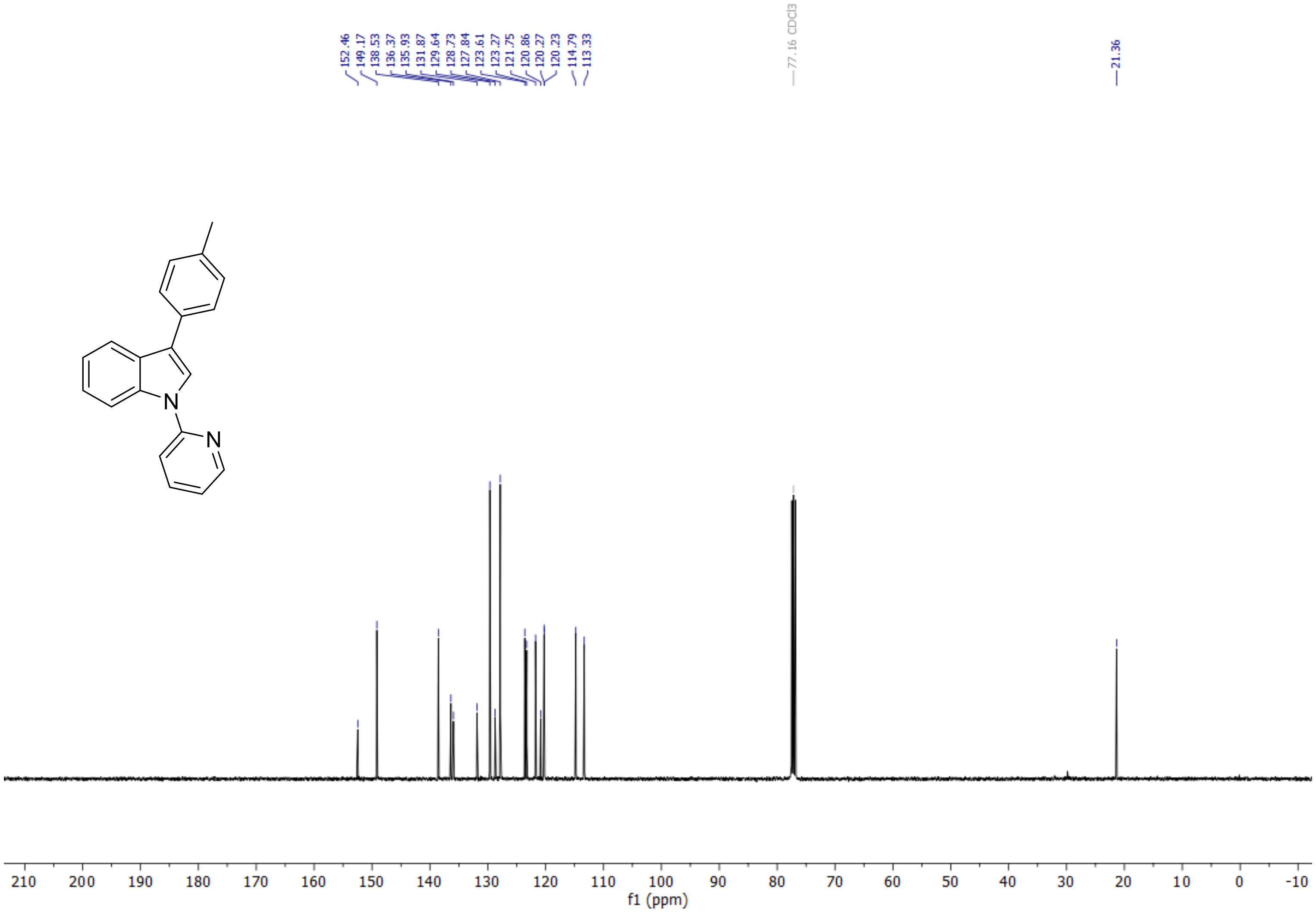

$^{1}H–^{13}C$ HSQC-DEPT NMR (400 MHz, $CDCl_3$) of **2r**

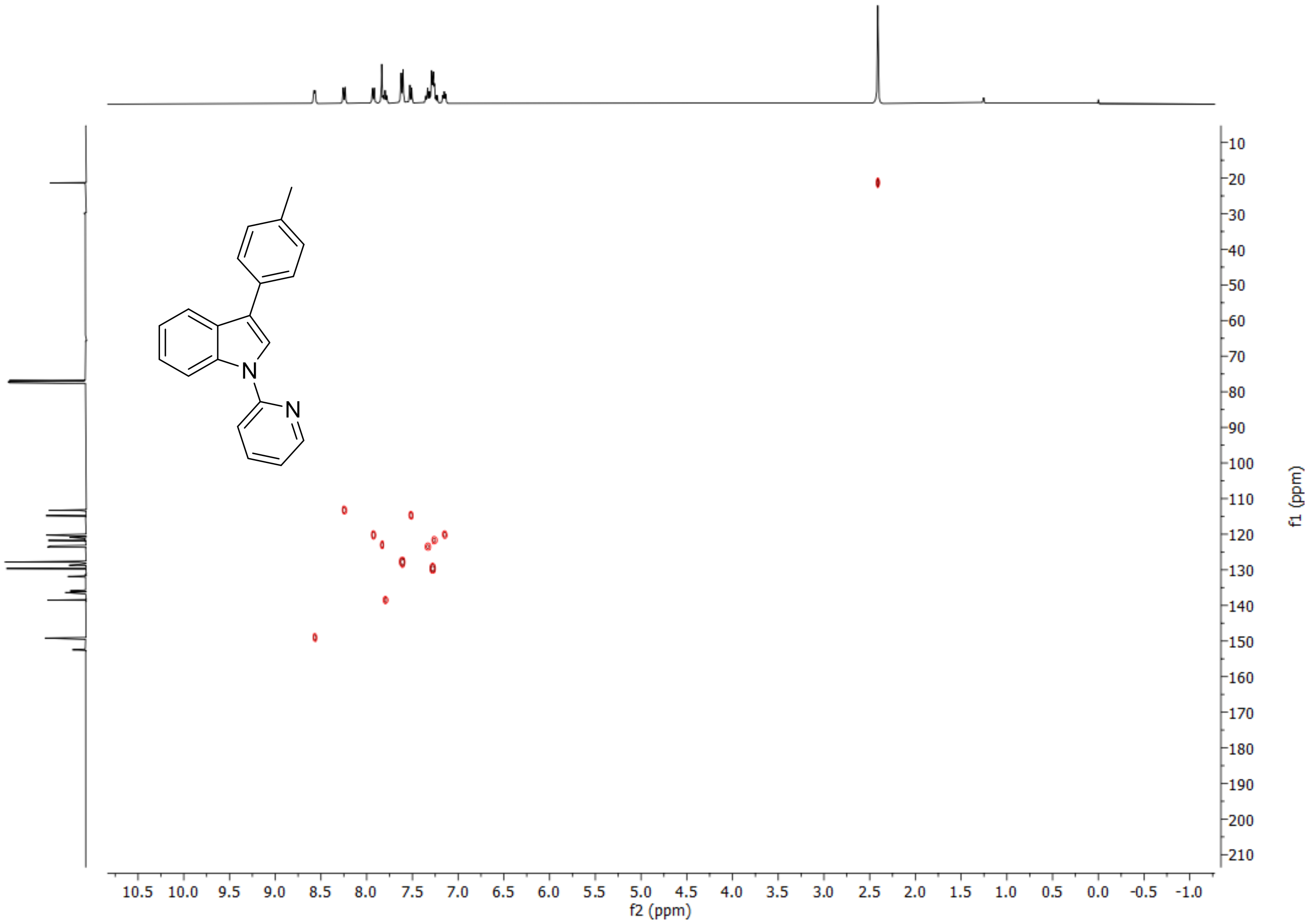

^{1}H NMR (400 MHz, $CDCl_3$) of **2s**

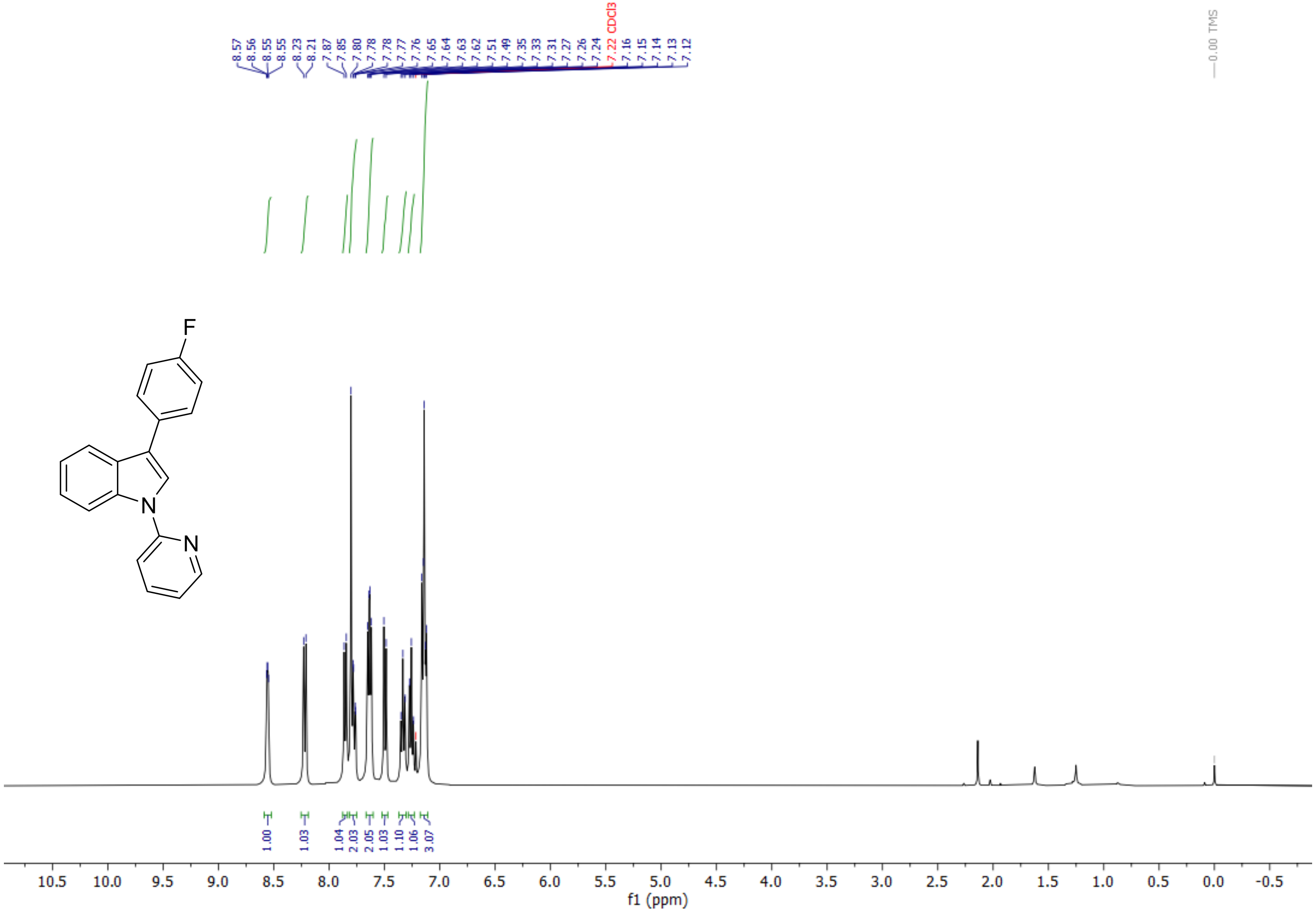

^{13}C NMR (101 MHz, $CDCl_3$) of **2s**

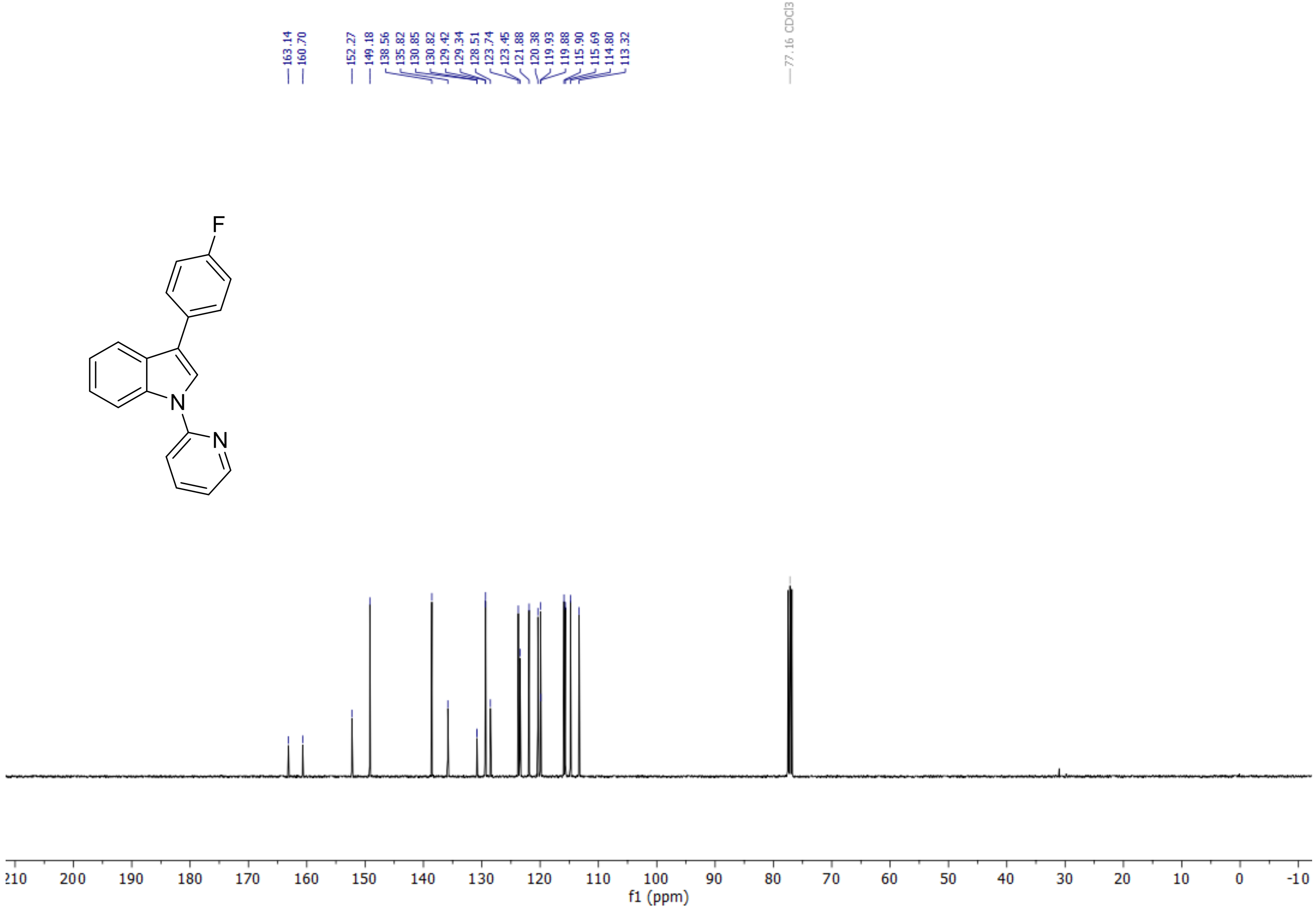

^{1}H–^{13}C HSQC-DEPT NMR (400 MHz, $CDCl_3$) of **2s**

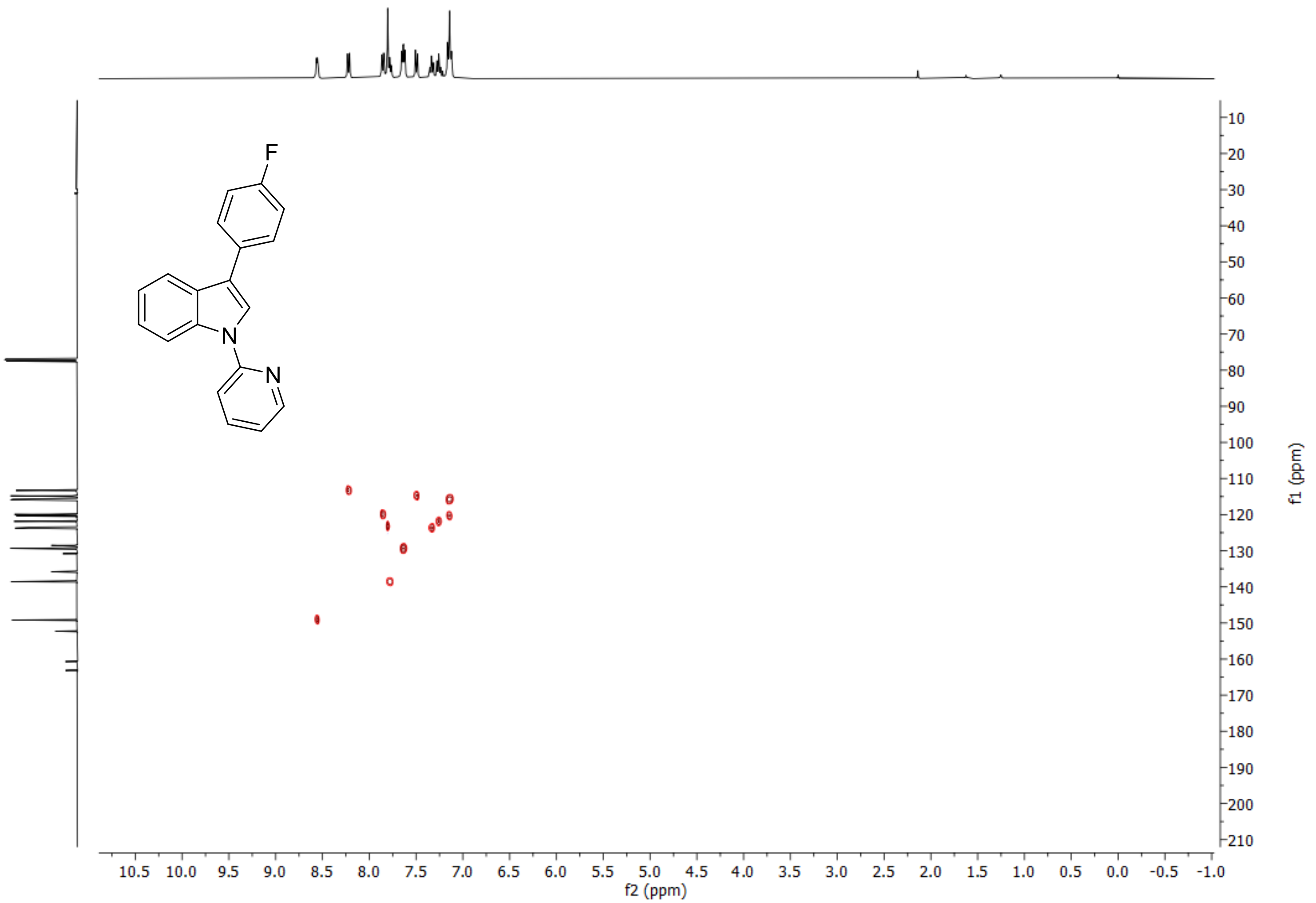

^{19}F NMR (376 MHz, $CDCl_3$) of **2s**

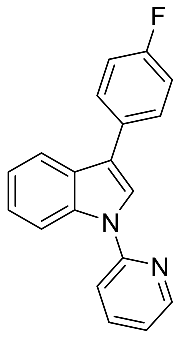

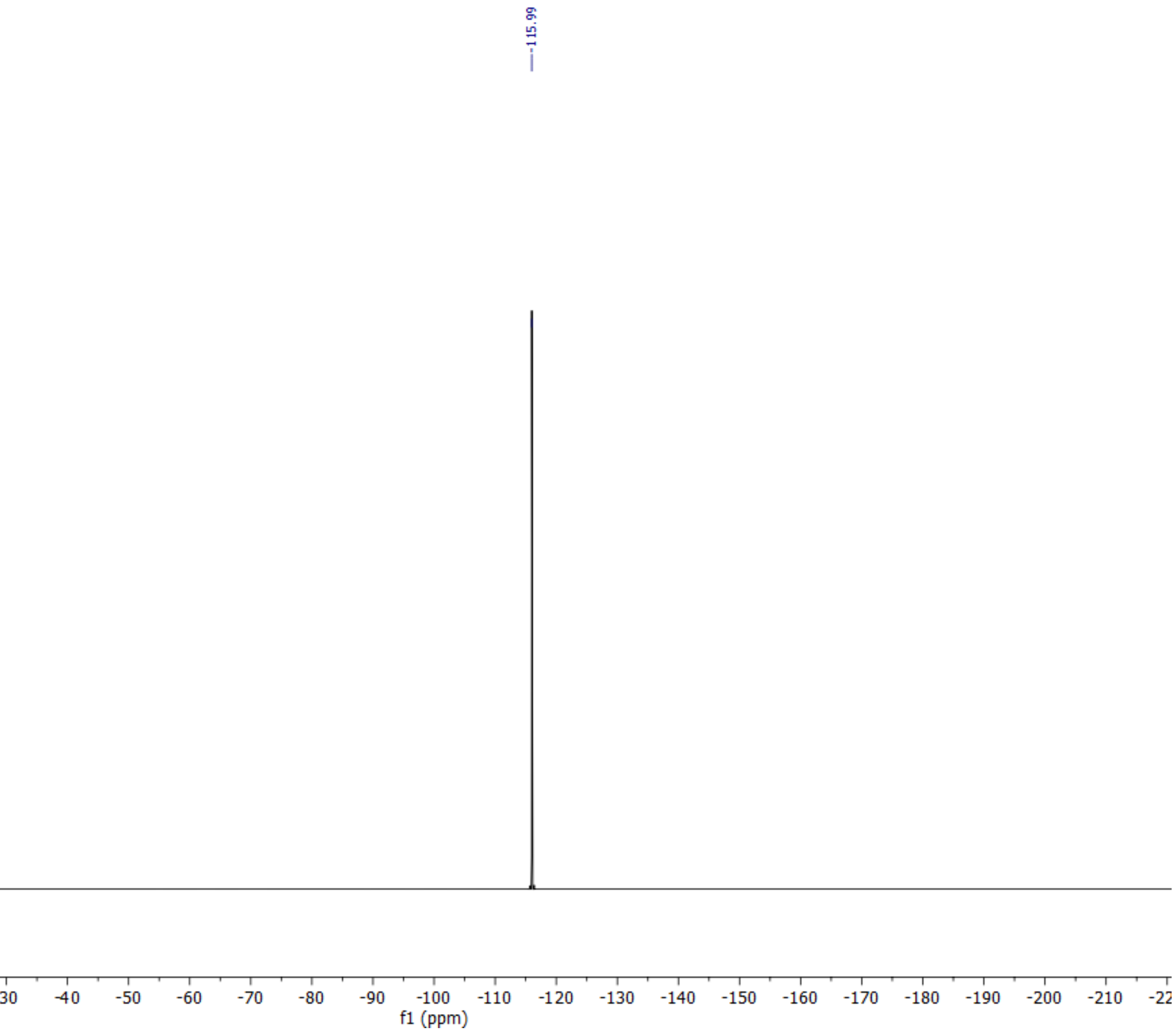

^{1}H NMR (400 MHz, $CDCl_3$) of **2t**

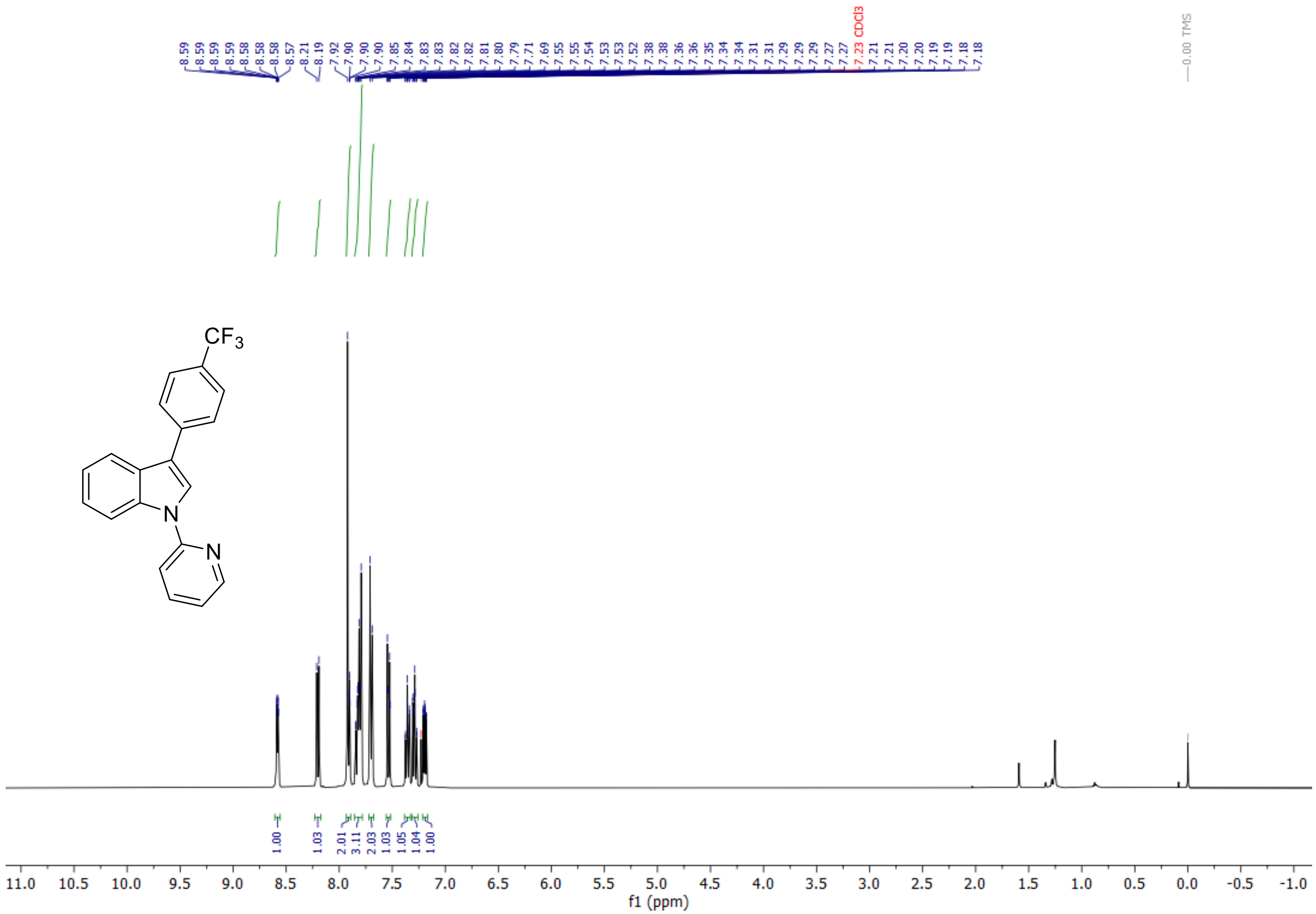

^{13}C NMR (101 MHz, $CDCl_3$) of **2t**

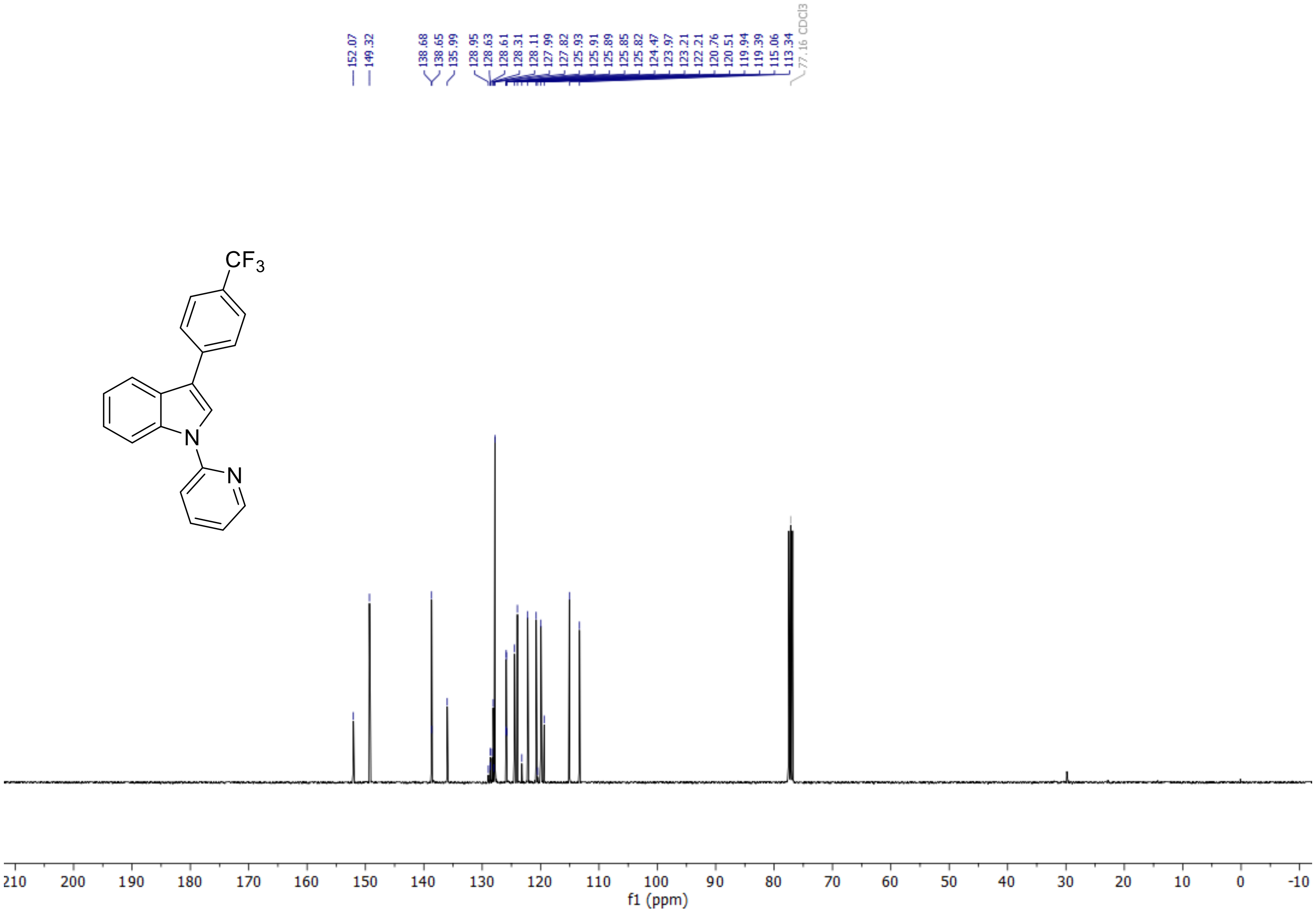

^{1}H–^{13}C HSQC-DEPT NMR (400 MHz, $CDCl_3$) of **2t**

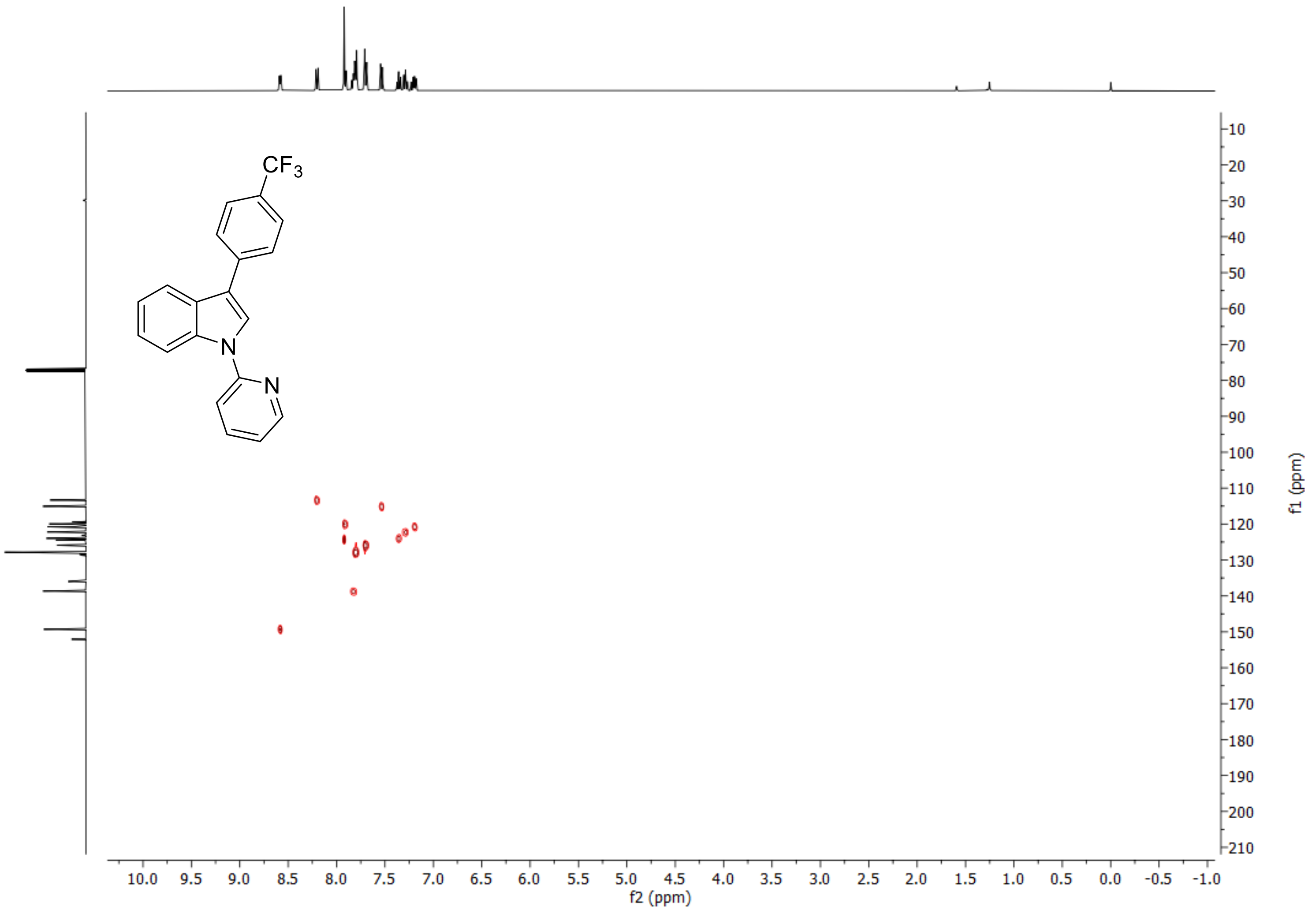

^{19}F NMR (376 MHz, $CDCl_3$) of **2t**

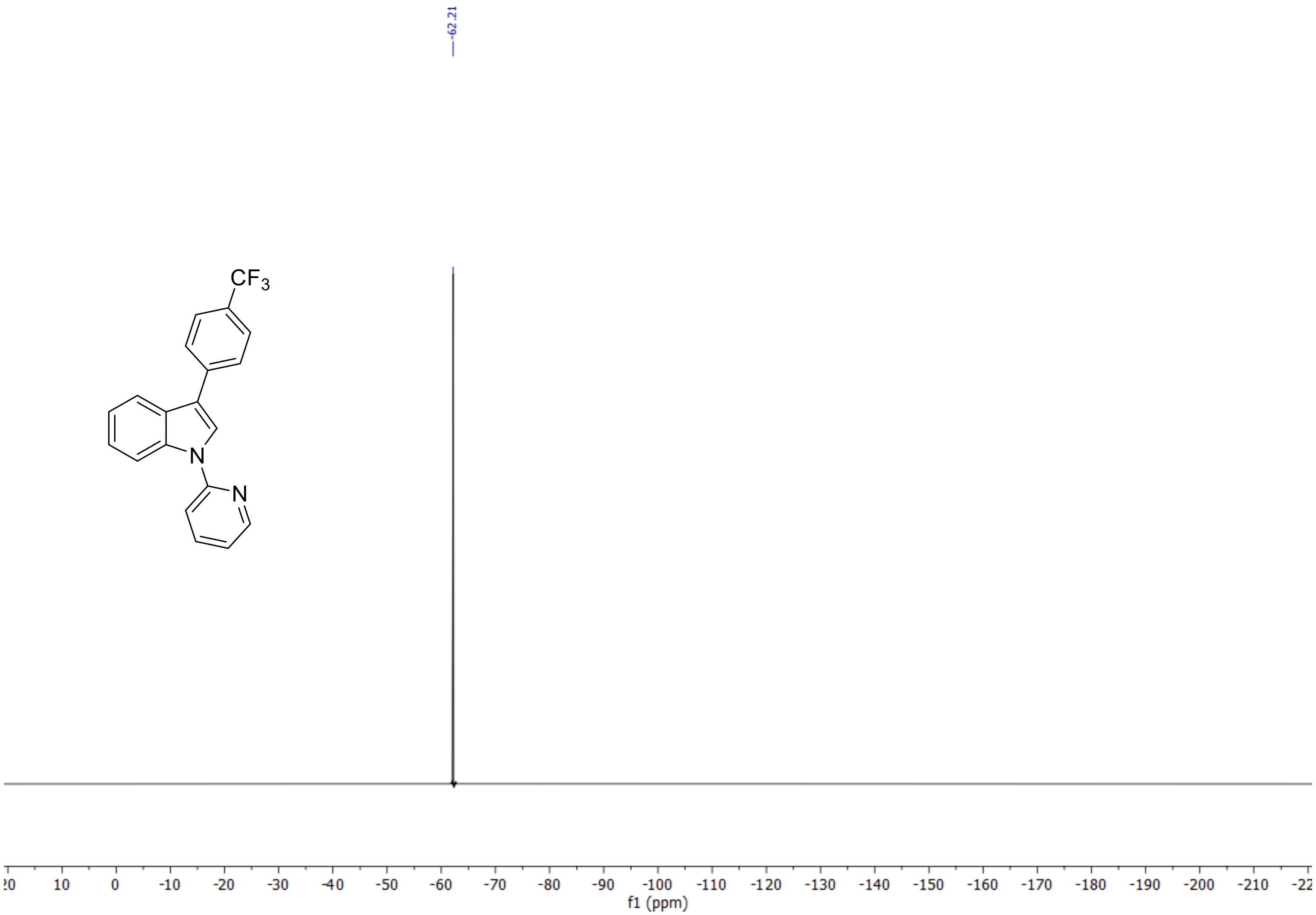

^{1}H NMR (400 MHz, $CDCl_3$) of **2u**

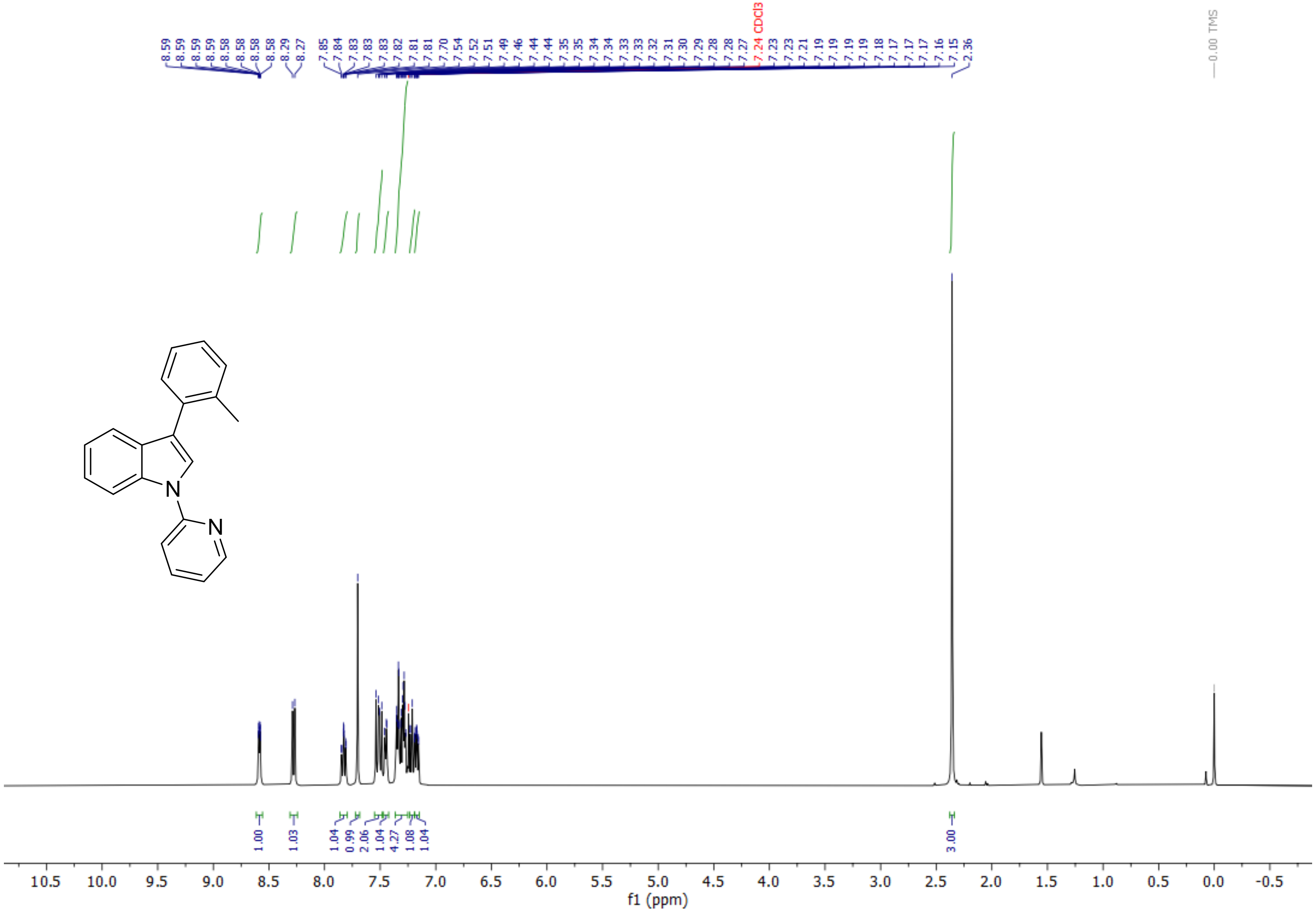

^{13}C NMR (101 MHz, $CDCl_3$) of **2u**

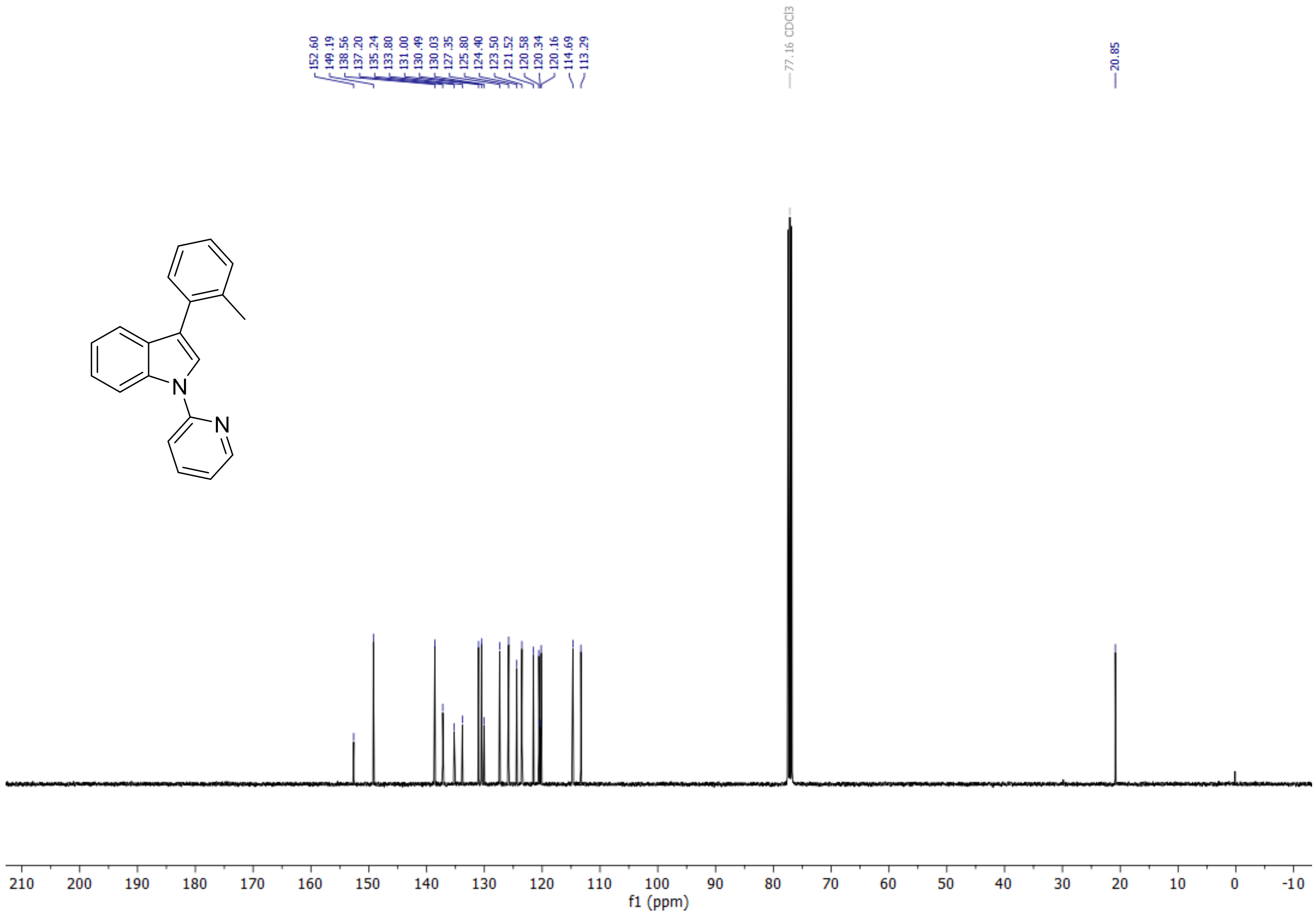

^{1}H–^{13}C HSQC-DEPT NMR (400 MHz, $CDCl_3$) of **2u**

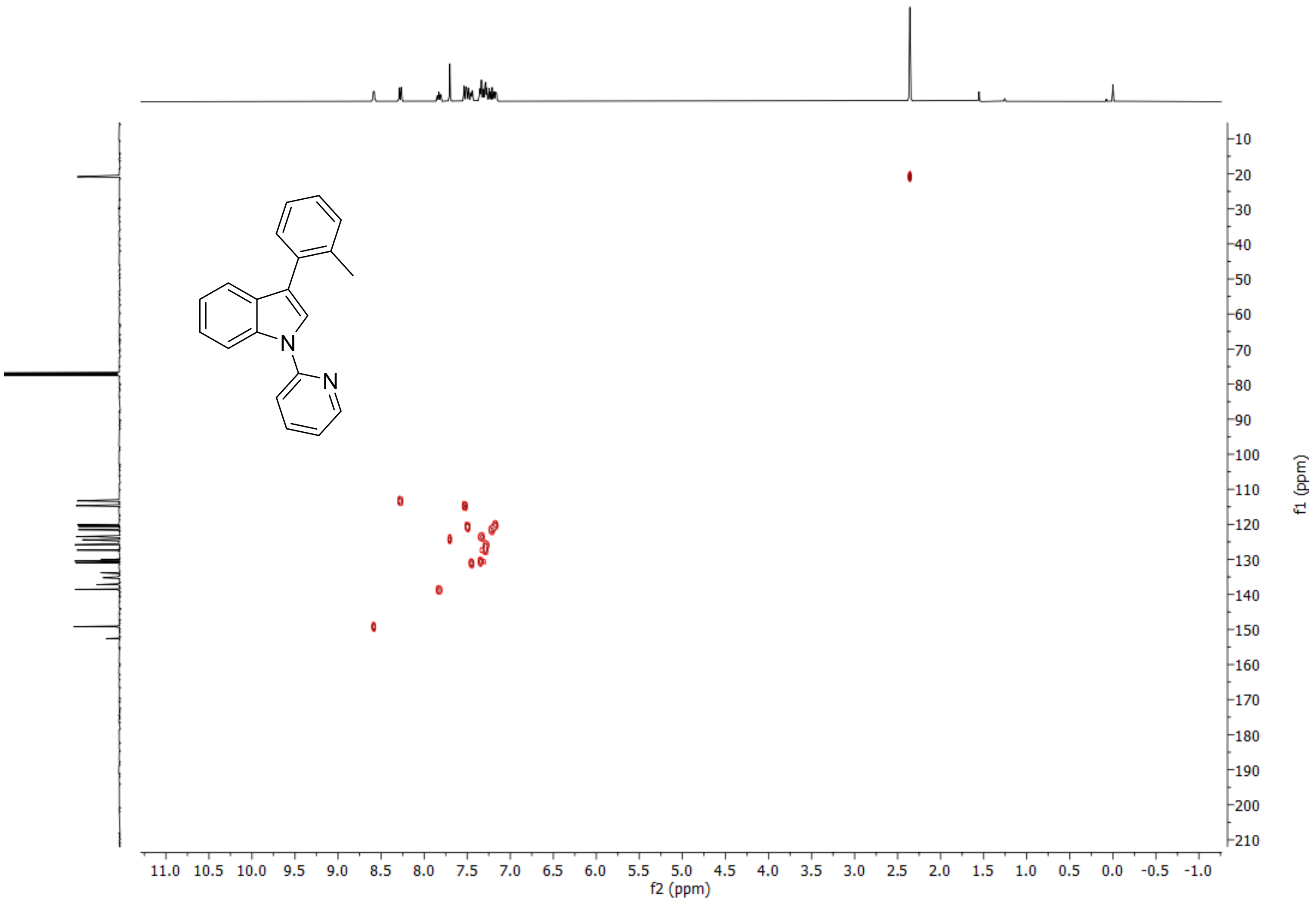

^{1}H NMR (400 MHz, $CDCl_3$) of **2v**

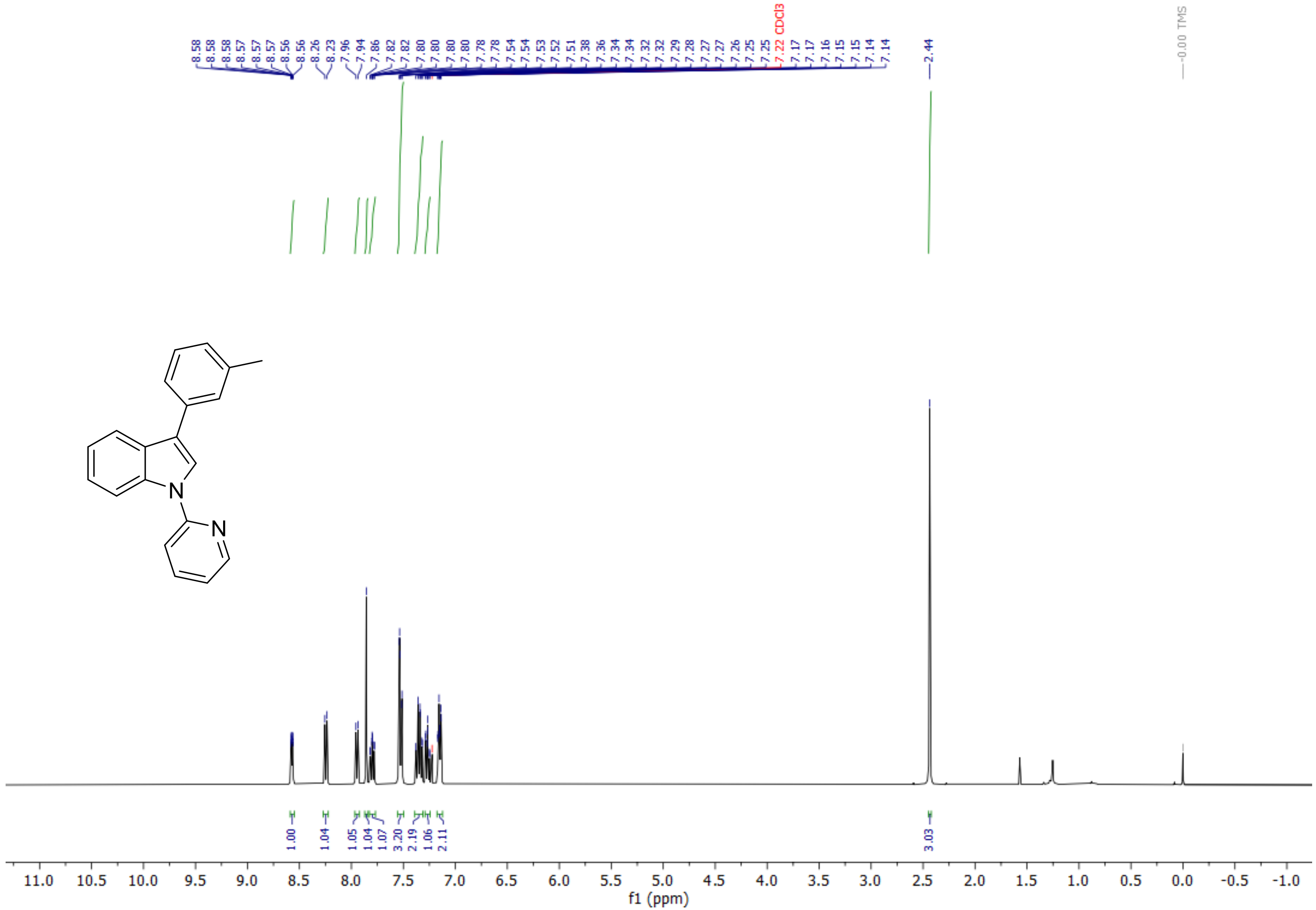

^{13}C NMR (101 MHz, $CDCl_3$) of **2v**

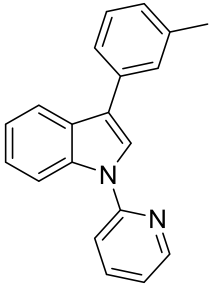

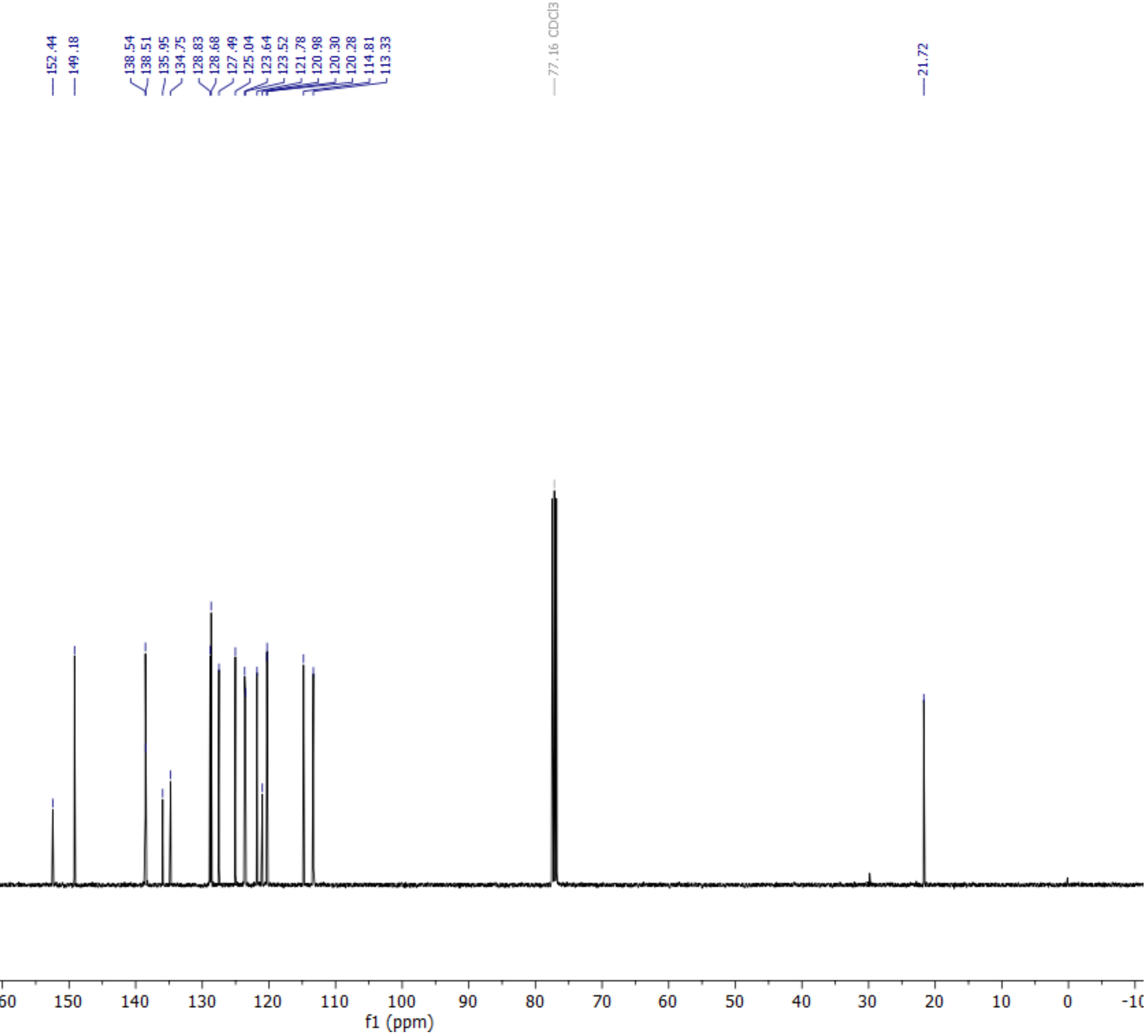

$^{1}H–^{13}C$ HSQC-DEPT NMR (400 MHz, $CDCl_3$) of **2v**

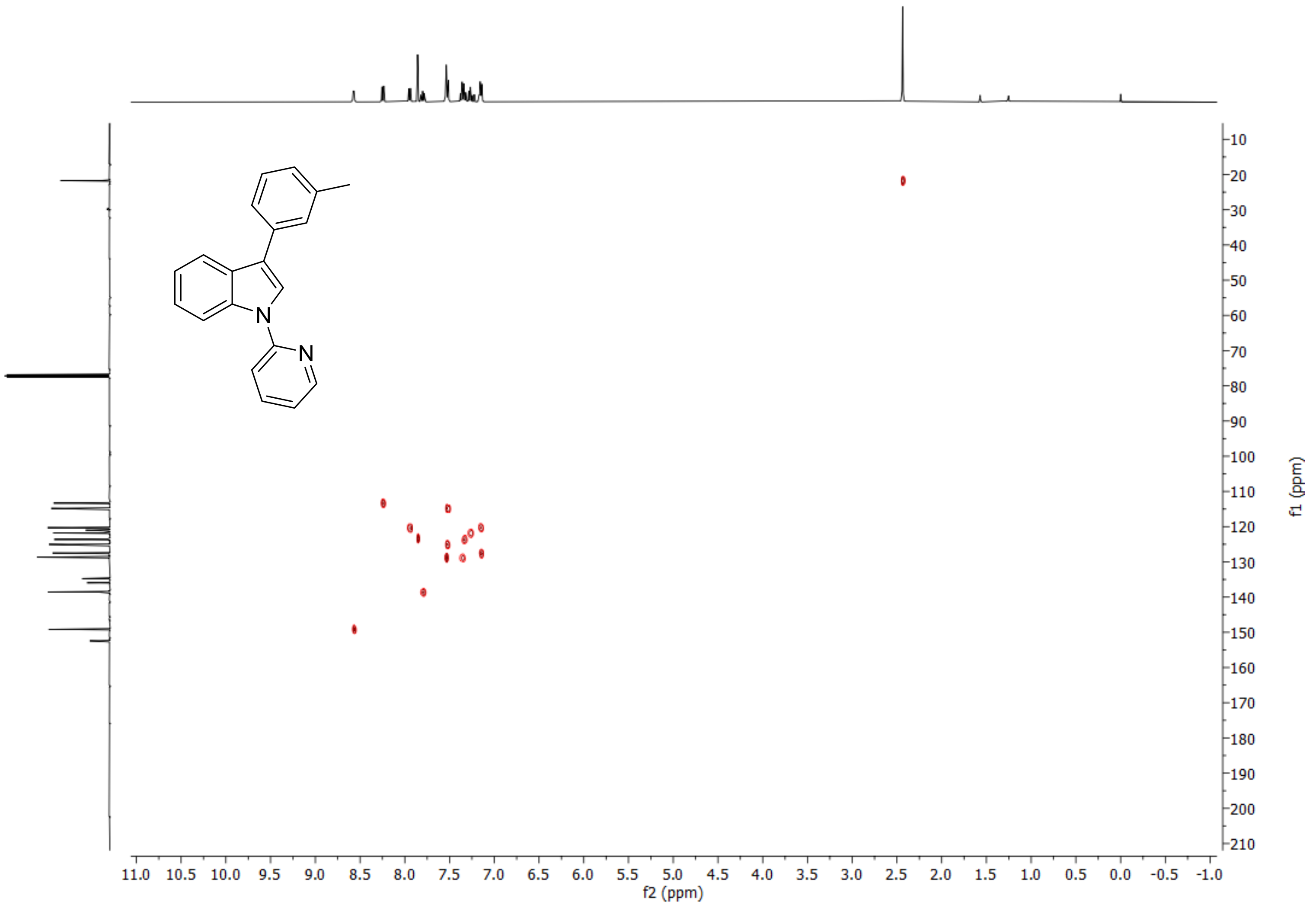

^{1}H NMR (400 MHz, $CDCl_3$) of **3a**

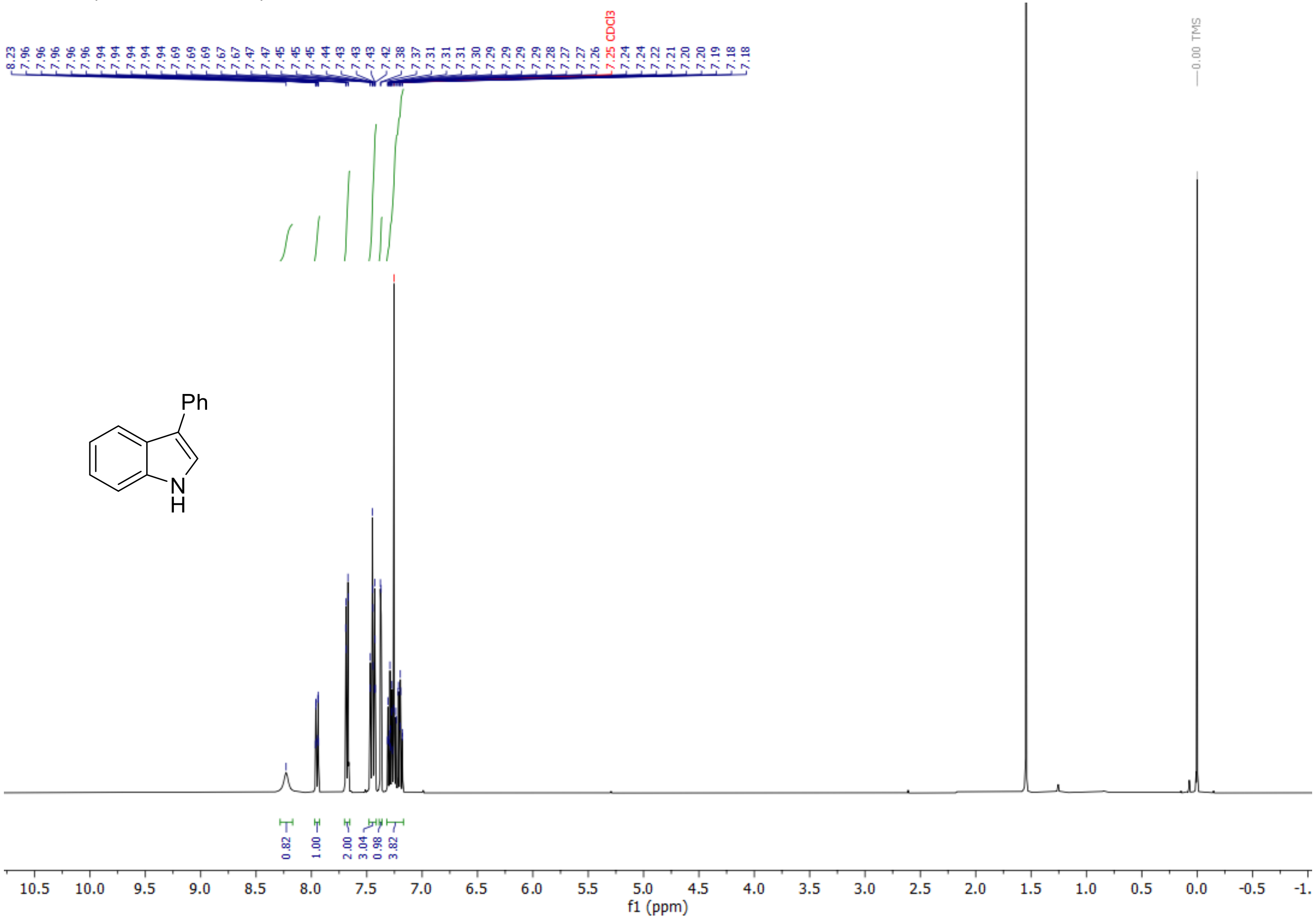

^{1}H NMR (400 MHz, $CDCl_3$) of **S1**

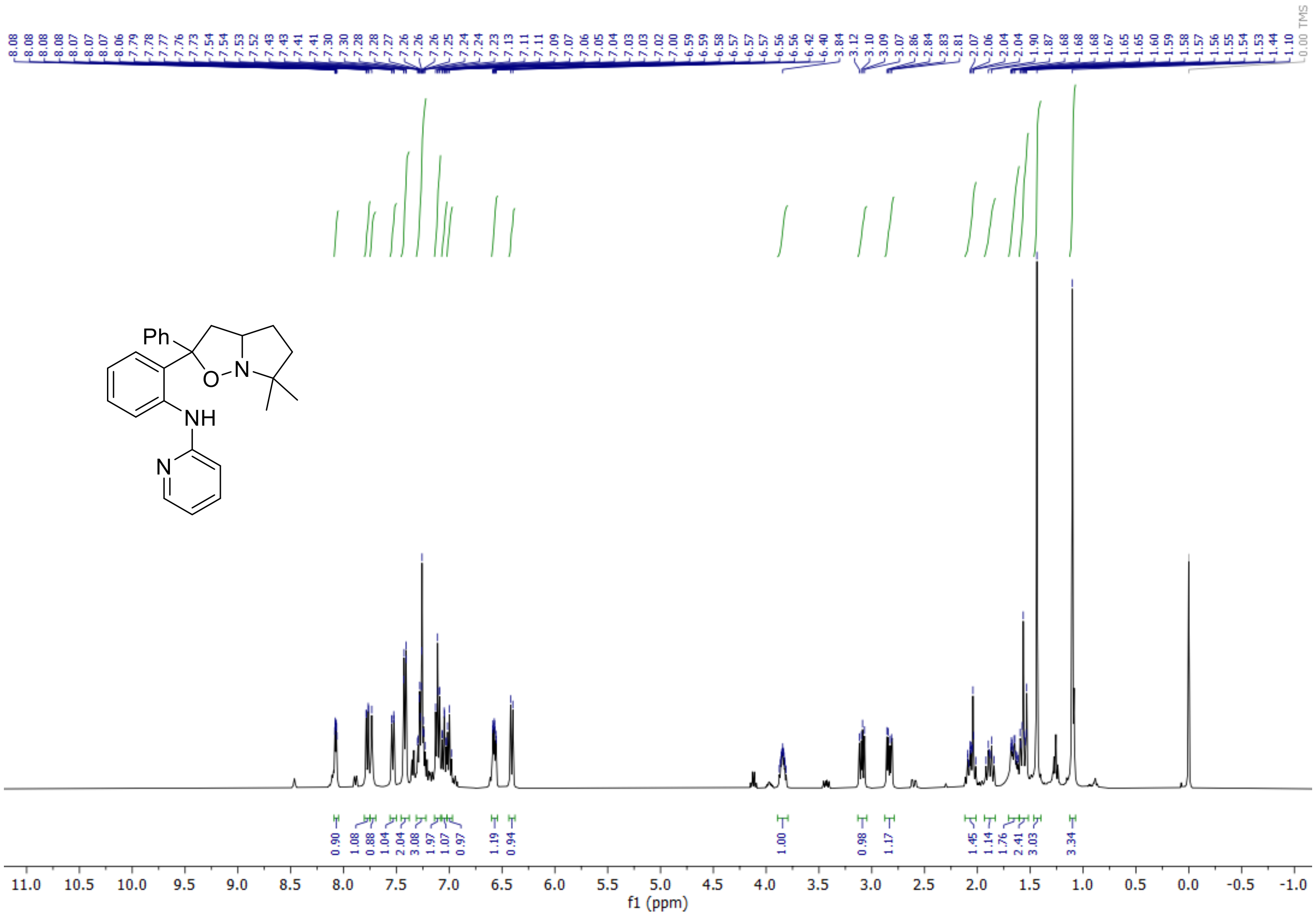

^{13}C NMR (101 MHz, $CDCl_3$) of **S1**

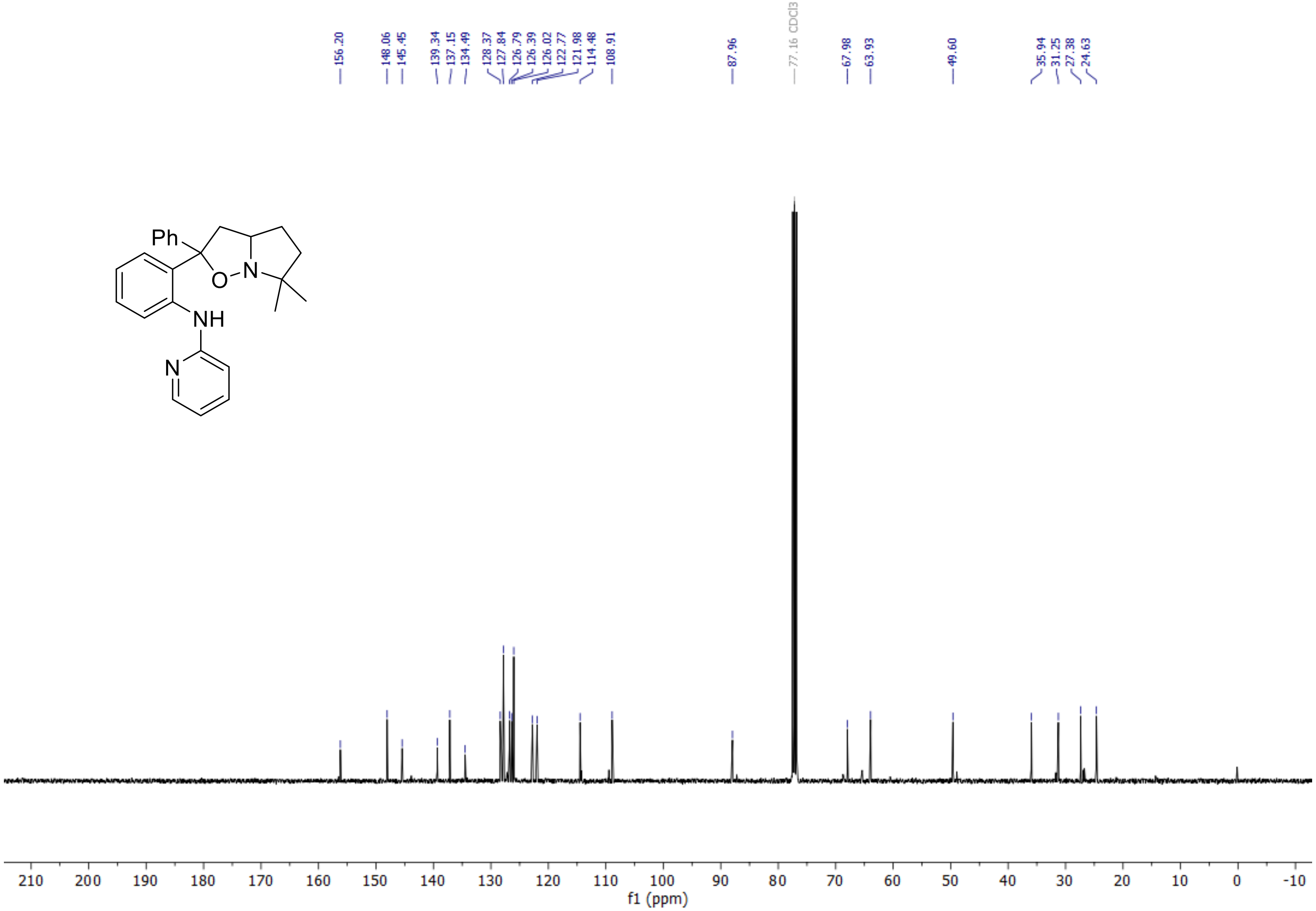

^{1}H–^{13}C HSQC-DEPT NMR (400 MHz, $CDCl_3$) of **S1**

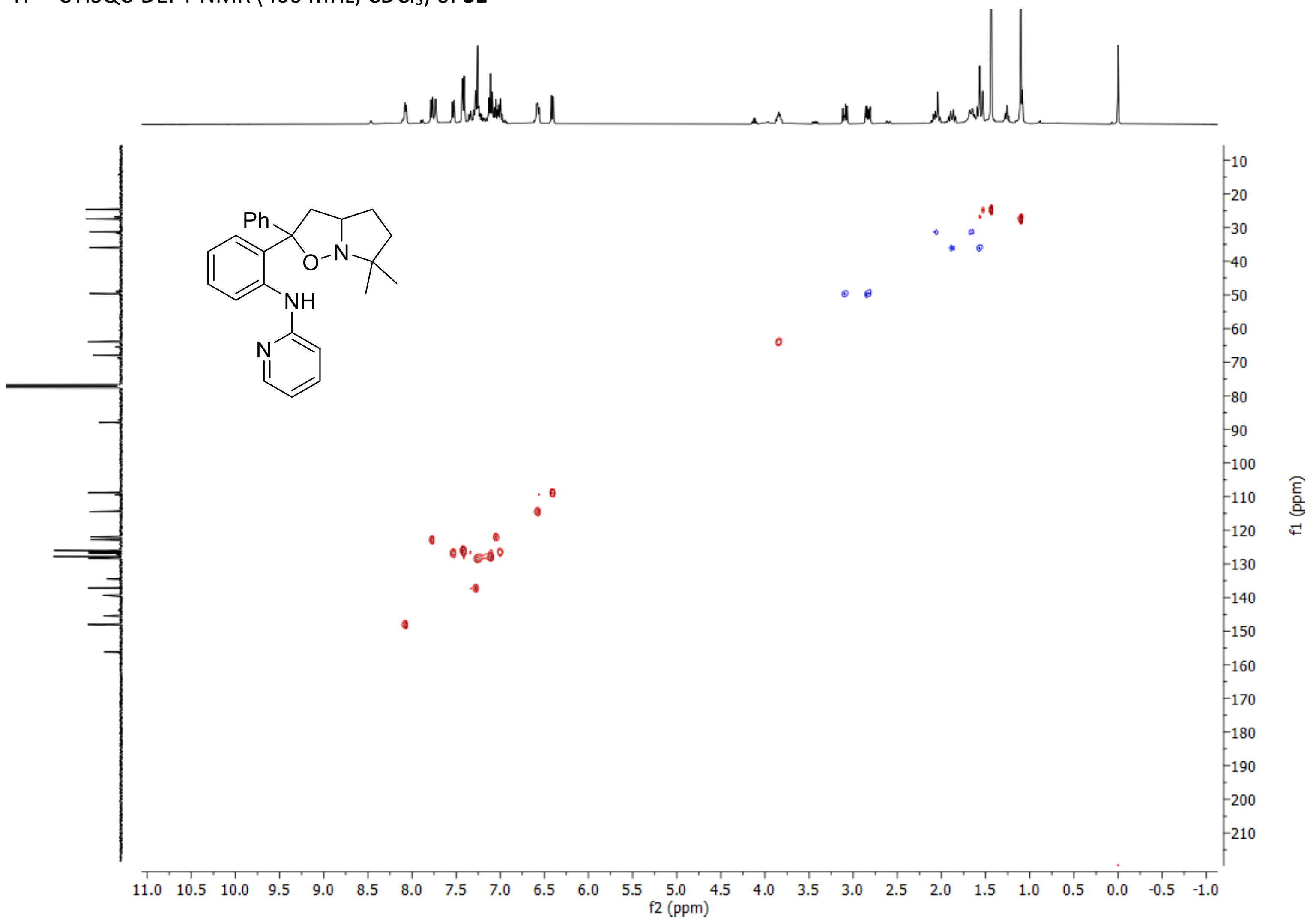

^{1}H–^{15}N HSQC NMR (500 MHz, $CDCl_3$) of **S1**

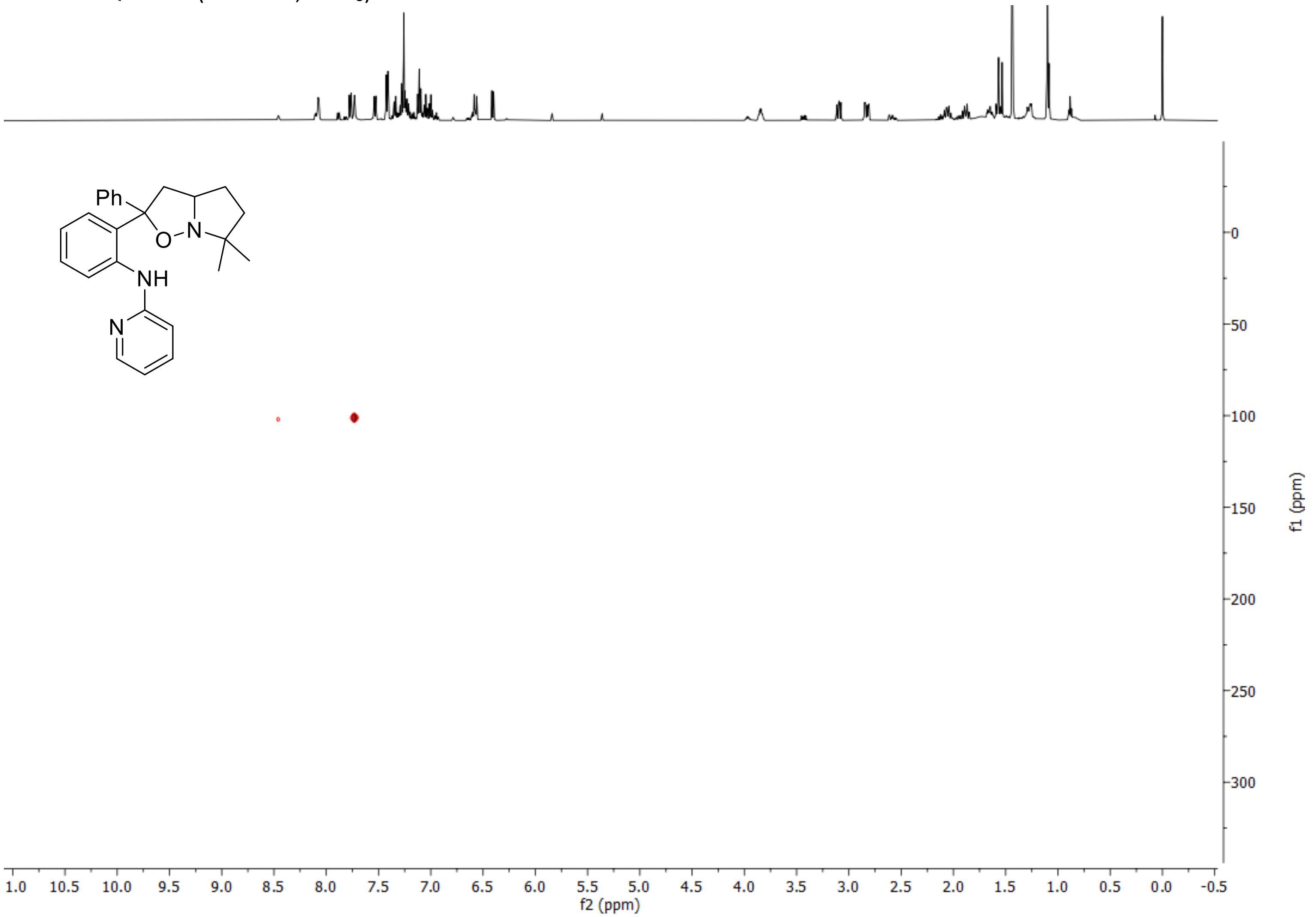

Supplement: Supplementary file 1 [file ol5c03410_si_001.pdf]
